# Comparative metabolite analysis of *Piper sarmentosum* organs approached by LC–MS-based metabolic profiling

**DOI:** 10.1007/s13659-024-00453-z

**Published:** 2024-05-14

**Authors:** Ismail Ware, Katrin Franke, Andrej Frolov, Kseniia Bureiko, Elana Kysil, Maizatulakmal Yahayu, Hesham Ali El Enshasy, Ludger A. Wessjohann

**Affiliations:** 1 Department of Bioorganic Chemistry, Leibniz Institute of Plant Biochemistry, 06120 Halle (Saale), Germany; 2 Biotechnology Research Institute, Universiti Malaysia Sabah, Jalan UMS, Kota Kinabalu 88400, Sabah, Malaysia; 3 Institute of Bioproduct Development, Universiti Teknologi Malaysia (UTM), 81310 Johor Bahru, Johor, Malaysia; 4 Institute of Biology/Geobotany and Botanical Garden, Martin Luther University Halle-Wittenberg, 06108 Halle (Saale), Germany; 5 German Centre for Integrative Biodiversity Research (iDiv) Halle-Jena-Leipzig, 04103 Leipzig, Germany; 6 City of Scientific Research and Technology Applications, New Borg Al Arab, Alexandria, 21934 Egypt

**Keywords:** *Piper sarmentosum*, LC–MS/MS, Multivariate analysis, Metabolite profiling, Secondary metabolites, Alkaloids

## Abstract

*Piper sarmentosum* Roxb. (Piperaceae) is a traditional medicinal and food plant widely distributed in the tropical and subtropical regions of Asia, offering both health and culinary benefits. In this study the secondary metabolites in different organs of *P. sarmentosum* were identified and their relative abundances were characterized. The metabolic profiles of leaves, roots, stems and fruits were comprehensively investigated by liquid chromatography high-resolution mass spectrometry (LC-HR-MS) and the data subsequently analyzed using multivariate statistical methods. Manual interpretation of the tandem mass spectrometric (MS/MS) fragmentation patterns revealed the presence of 154 tentatively identified metabolites, mostly represented by alkaloids and flavonoids. Principle component analysis and hierarchical clustering indicated the predominant occurrence of flavonoids, lignans and phenyl propanoids in leaves, aporphines in stems, piperamides in fruits and lignan-amides in roots. Overall, this study provides extensive data on the metabolite composition of *P. sarmentosum*, supplying useful information for bioactive compounds discovery and patterns of their preferential biosynthesis or storage in specific organs. This can be used to optimize production and harvesting as well as to maximize the plant’s economic value as herbal medicine or in food applications.

**Graphical Abstract:**

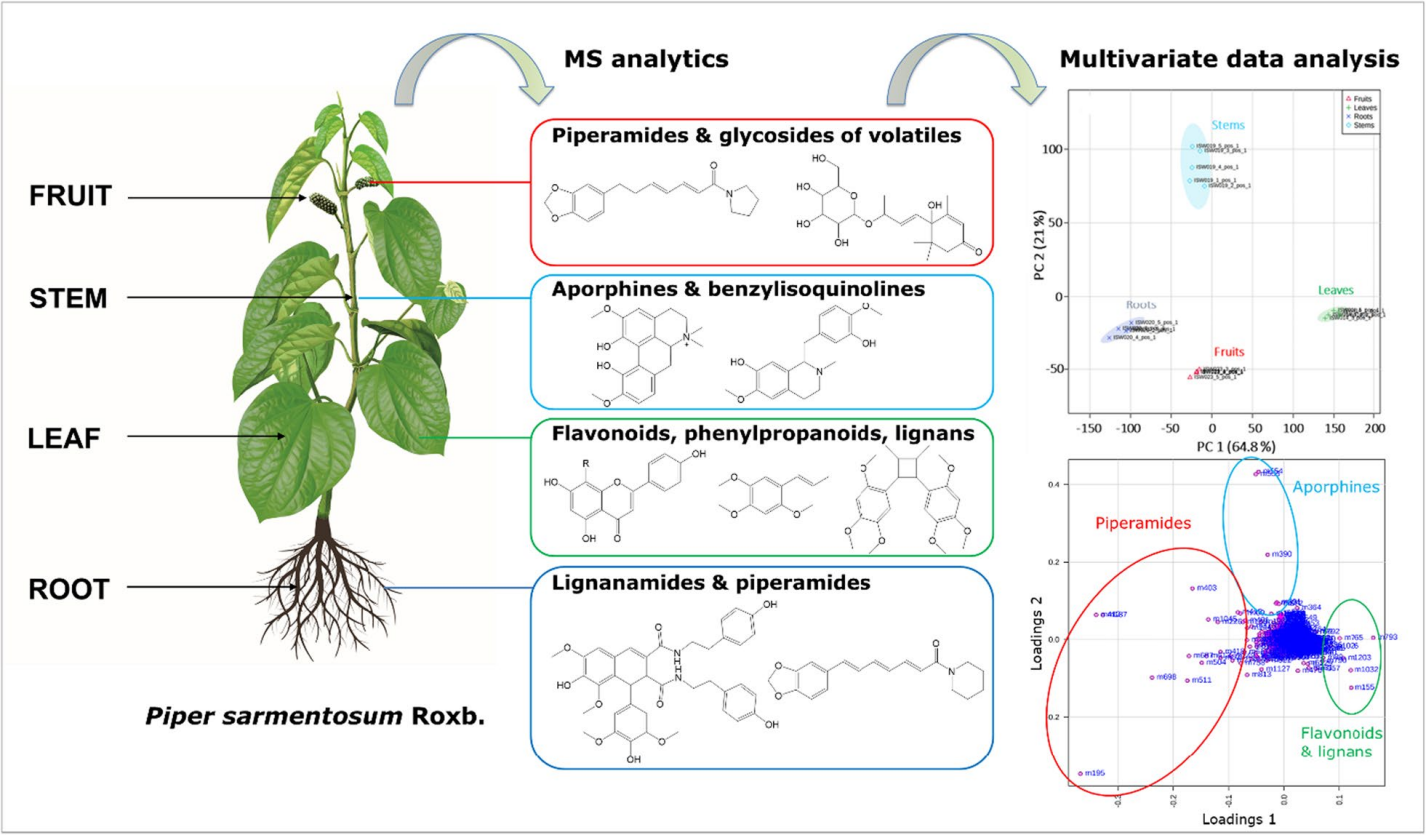

**Supplementary Information:**

The online version contains supplementary material available at 10.1007/s13659-024-00453-z.

## Introduction

*Piper* is one of the most diverse genera in the Piperaceae family [1], comprising approximately 2000 species, followed by *Peperomia* with 1700 species [2, 3]. The *Piper* species are widely distributed in tropical regions, having its center of origin in Peninsular Malaysia, but are also common in the American tropics [4]. About 600 species of *Piper* are reported from Asia, most of them are distributed in the Malesia region [5]. Many of these *Piper* species have long been used as food and in traditional medicine. Some of such applications were pharmacologically proven [6]. The most famous representative of this genus is *Piper nigrum* L., the well-known black pepper (The "pepper") used as spice and seasoning [7, 8].

*Piper sarmentosum* Roxb. is common in several Asian countries. The whole plant, leaves, fruits, stems and roots have been used as medicines for decades and proved to have significant nutritional and medicinal value [9]. The tender leaves of *P. sarmentosum* are consumed as vegetables and are traditionally used in Malaysia and Indonesia to treat malaria, cough and rheumatic pain [10, 11]. The population of Thailand commonly employ the whole plant to treat muscle pain and hyperglycemia, while the leaf is consumed as an indigenous vegetable [12, 13]. In Chinese folk medicine, the whole plant, including leaves, stems, roots and fruits are used to treat a variety of diseases such as uterine bleeding, rheumatic bone pain, traumatic injuries, insect and snake bites, toothache, fever, and stomach pain. In addition, some communities in the southern part of China often eat roots, fruits and leaves as traditional edible vegetables [14, 15].

Several previous studies on *P. sarmentosum* focused mainly on the constituents and pharmacological activities of the leaves [6, 15–17]. Pharmacological studies recently conducted in Malaysia showed that the extract of *P. sarmentosum* leaves has vascular protective, neuroprotective, anti-obesity, and anti-hyperlipidemia activity [18–20]. However, little is known about the metabolite composition and medicinal value of other organs. Moreover, the metabolite profiles and inter-organ chemical differences have not been systematically elucidated in *P. sarmentosum* by LC-HR-MS-based metabolomics so far.

LC–MS-based metabolite profiling is the method of choice to address the complex patterns of biologically active natural products in herbal plants [21]. In its state-of-the-art implementation, this technique relies on the ultra-high performance liquid chromatography (UHPLC) coupled on-line to electrospray ionisation-quadrupole time-of-flight mass spectrometry (ESI-QqTOF-MS). Currently, UHPLC is the best method of liquid chromatography in terms of resolution, sensitivity, and throughput, while QqTOF-MS is one of the most sensitive, accurate, fast and processive detectors, which is ideal for identification and quantification of a wide range of natural products in the most complex and challenging samples. Due to its powerful strategic combination of high resolution and sensitivity, the coupling of UHPLC to ESI-QqTOF-MS has been successfully used to analyze complex samples [22]. The high throughput of this platform allows to discriminate different plant varieties [22–24], plant organs [25], plant developmental stages [26, 27], the geographical origins of plants [28–30], and to determine the quality of traditional medicinal plants and processed products [31–34].

Therefore, in the present study, the metabolic profiles of four different organs of *P. sarmentosum,* namely the leaf, stem, root and fruit, were comprehensively characterized by UHPLC coupled on-line to ESI-QqTOF-MS. The variability of metabolite contents was measured, and potential biomarkers of each organ could be identified by multivariate analysis techniques, i.e. principal component analysis (PCA) with loading plots, orthogonal partial least squares-discriminant analysis (OPLS-DA), and hierarchical clustering. These statistical methods provide a comprehensive and intuitive description of the chemical constituents in the four organs of *P. sarmentosum* which can serve as valuable resources for further functional studies on the species.

## Results and discussion

### Analysis and comparison of LC–MS fingerprints

The present study employed the high-resolution RP-UHPLC-ESI-QqTOF-MS to determine the intra-organ variation and relative abundances of metabolites extracted from leaf, stem, root and fruit of *P. sarmentosum*. The aim was to achieve high chromatographic resolution to detect as many individual chemical species in a single batch analysis as possible. First, to determine the appropriate ion detection mode, the SWATH experiments were performed in both positive and negative ionization modes (See Figure S1 for TIC of each organ in negative ion mode). According to the results of these experiments, LC–MS analysis in positive ion mode revealed essentially more peaks, better sensitivity and more rich and explicit structural information. This can be explained by the presence of numerous metabolites in the test samples more likely to be ionized in the positive ion mode than in negative one, as e.g. alkaloids. Major peaks in the negative ion mode represent flavonoids (Figure S1), however, this compound class also could be detected and analyzed in the positive ion mode. Therefore, only the positive ionization mode was used for all further series of MS analyses.

To monitor the reproducibility and instrument performance, aliquots of a quality control (QC) sample (i.e. a pooled extract prepared from the samples of all organs) were systematically measured between sample sequences. Figure S2 shows the close clustering of QC samples in a principal component analysis score plot. The stability of retention times, UHPLC performance and especially the high-resolution mass accuracy was also examined by the presence of orcinol (C_7_H_8_O_2_) and kinetin (C_10_H_9_N_5_O) as internal standards (IS). Both cost-efficient and routinely used ISs eluted at specific retention times, (4.9 min for orcinol and 5.3 min for kinetin), showed good ionization properties and do not natively appear in any organ of *P. sarmentosum*. Ideally, internal standards should cover a wider polarity range of the chromatogram, however, the appearance of many compound peaks in the chromatogram region between 10 and 20 min did not allow the application of an internal standard eluting in this retention time range without overlap.

**Fig. 1:**
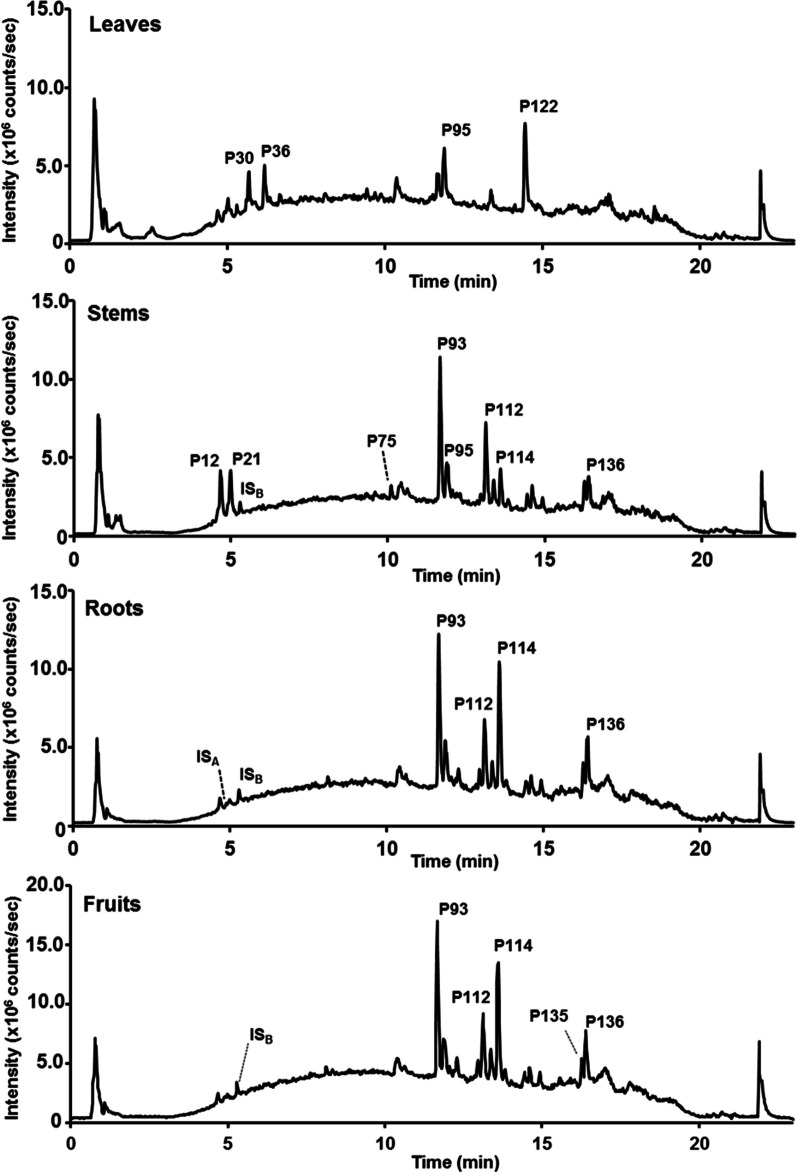
Comparison of the total ion chromatograms (TICs) acquired from methanolic extracts of different *P. sarmentosum* organs (leaves, stems, roots and fruits) in the positive ion mode: *IS*_*A*_  internal standard orcinol, *IS*_*B*_  internal standard kinetin, and *P* peak number

The representative total ion chromatograms (TICs) acquired from the extracts of the different organs are shown in Fig. 1. The efficient RP-UHPLC separation of *P. sarmentosum* metabolites was successfully achieved in 23 min. Most major peaks from different organs exhibit clear qualitative and quantitative (intensity) differences. For example, the TICs of fruit and root extracts showed a lower intensity of peaks eluting between 5 and 10 min compared to the extracts prepared from other organs (Fig. 1). On the other hand, the peaks eluting between 11 and 17 min demonstrated clearly higher intensity in these extracts. In contrast, the leaf extracts were featured with an opposite pattern of relative metabolite abundances, and stems were intermediate. Thus, our results clearly demonstrate that the quantitative patterns of characteristic *P. sarmentosum* metabolites depend on the plant organ. (For qualitative differences see below).

**Fig. 2:**
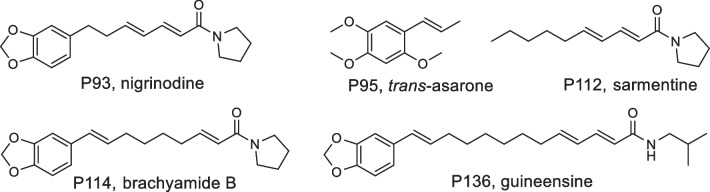
The structures of selected compounds representing the most prominent peaks in the TICs of extracts from different organs of *P. sarmentosum*

In this study, these patterns were analyzed by electrospray (ESI) high-resolution-mass spectrometry (HR-MS) to enable the determination of the elemental composition. Based on these data, complemented with mass spectral fragmentation patterns obtained in MS/MS experiments, comparison with databases and co-elution experiments with authentic standard reference compounds (see chapter 2.4 for details) the most prominent peaks found in the t_R_ range of 11–17 min (Fig. 1) could be assigned to the groups of piperamides and phenylpropanoids. For example, peaks P93, P95, P112, P114 and P136 were putatively identified as nigrinodine, *trans*-asarone, sarmentine, brachyamide B and guineensine, respectively (Fig. 2).

### Characterization of the inter-organ differences by untargeted metabolite profiling

Due to its high mass accuracy, sensitivity and resolution, the HR-QqTOF-MS is the most efficient analytical platform for online structural elucidation of multiple components in plants [35]. The inter-sample alignment of the individual TICs corresponding to all features in the dataset resulted in a consensus peak table (data matrix comprising individual features as defined pairs of t_R_-*m/z* values) containing 1272 entries. These matched features comprised protonated molecular ions and other adducts, as well as in-source fragmentation products associated with characteristic neutral losses (matrix data not shown). The data matrix was subjected to multivariate statistics analysis with MetaboAnalyst software.

**Fig. 3:**
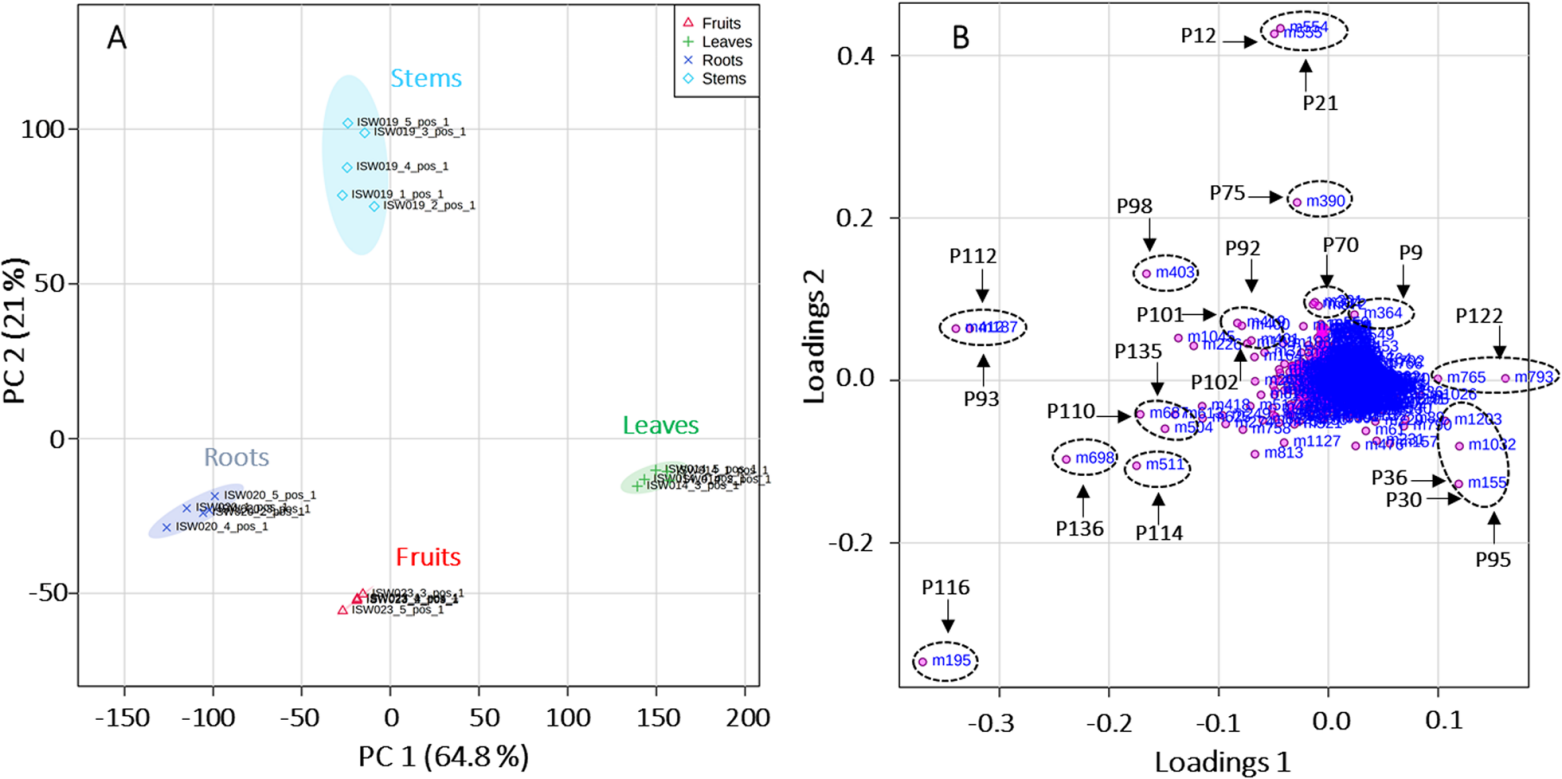
Principle component analysis (PCA) with scores (**A**) and loadings (**B**) plots accomplished with 1272 features detected in the RP-UHPLC-QqTOF-MS data of leaf, stem, root and fruit extracts from *P. sarmentosum*

Principal component analysis (PCA) is recognized to be an efficient tool for reducing complex data sets and providing important insight into the variation within and between experimental groups [24]. The results of the PCA analysis accomplished with our dataset (score and loadings plots) are shown in Fig. 3. As can be seen from the score plot, approximately 86% of the total variance was accounted for by the first two principal components. The first component (PC1) was responsible for 64.8% of the total variance, whereas the second component (PC2) explained 21.0% of the total variance.

**Fig. 4:**
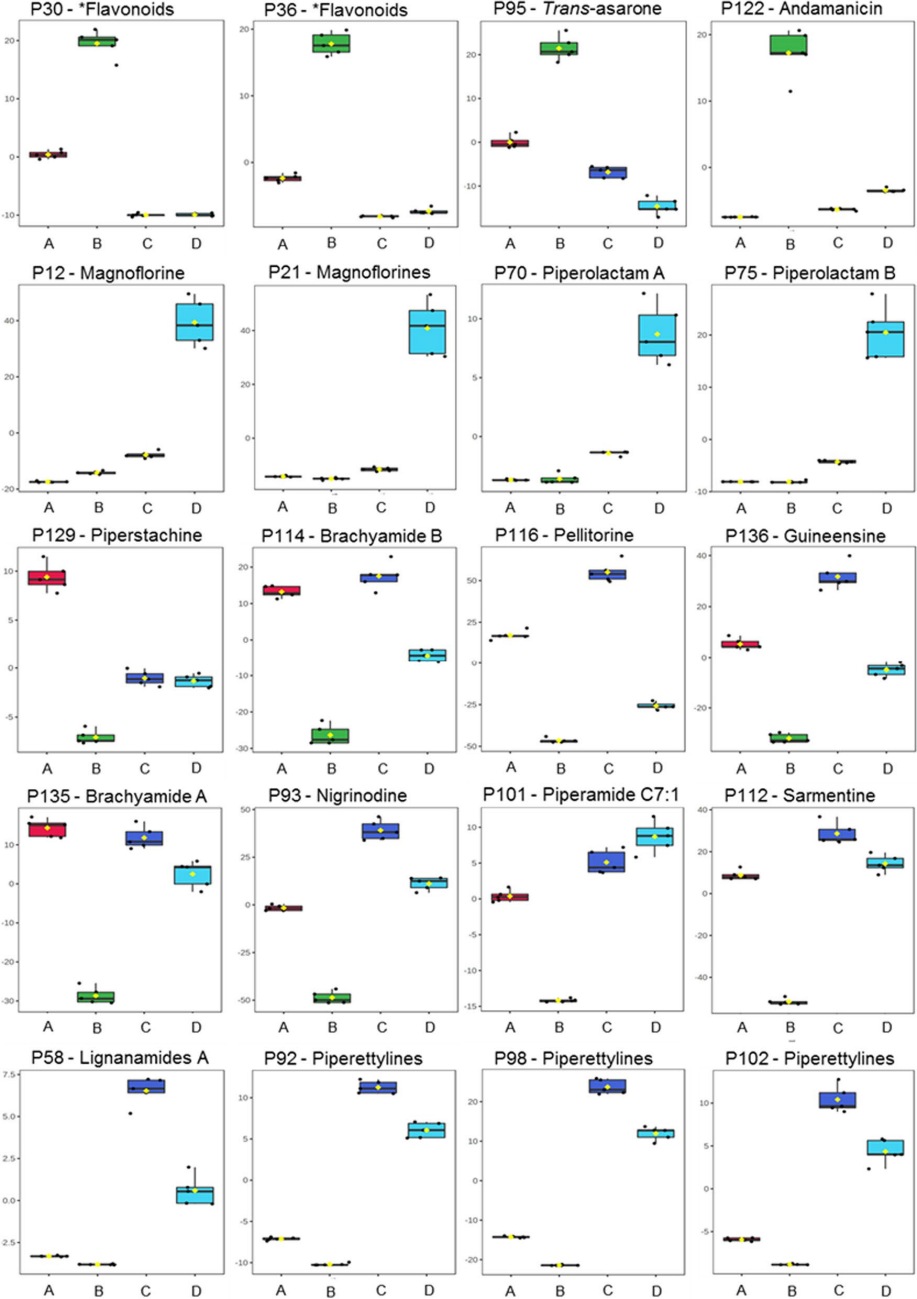
Boxplots illustrating the relative abundances of the individual secondary metabolites contributing to PCA separation demonstrate significantly different intensities of the corresponding chromatographic peaks, in fruits (red, **A**), leaves (green, **B**), roots (blue, **C**) and stems (cyan, **D**) of *P. sarmentosum*. Corresponding t_R_ and *m/z* values of metabolites are summarized in Table 1 and Table S1. *The flavonoids P30 and P36 represent Vitexin 2ʺ-*O*-galactoside and Vitexin 4ʺ-(3-hydroxy-3-methylglutaroyl)-2ʺ-*O*-*β*-D-glucopyranoside, respectively

The leaves were separated from the other organs in PC1, whereas PC2 indicates that stems appeared to be significantly different from other organs, here especially fruits, in terms of their metabolite patterns. The loadings plot gives access to the features contributing to the separation of organs, which is primarily due to differences in their abundance within the various test samples and specific *p* value (Fig. 3B). The presence of highly intense chromatographic signals corresponding to the neolignane andamanicin (P122), flavonoids (P30, P36) and the phenyl propanoid *trans*-asarone (P95) led to the positioning of leaves in a single group in PCA. Thereby, P122 was represented by two different features, namely its protonated molecular ion [M + H]^+^ (feature m765, t_R_ 14.5 min, *m/z* 417.2256) and the corresponding ammonium adduct [M + NH_4_]^+^ (feature m793, t_R_ 14.5 min, *m/z* 434.2530). Additionally, the existence of the unique aporphine-type compounds in stems, like magnoflorines (P12, P21), piperolactams A (P70) and B (P75), had a powerful discriminating effect for their separation from the other groups in the score plots. Finally, as can be seen from the TICs in Fig. 1, the metabolite profiles of fruits and roots are quite similar. Therefore, the corresponding individual samples are located in the same quadrant of the PCA score plot, though well separated. This fact can be explained by higher contents (manifested in the LC–MS dataset by higher relative intensities of corresponding molecular ionic species) of piperamides in these two organs, specifically pellitorine (P116), brachyamide B (P114) and guineensine (P136) (Fig. 3). Interestingly, peaks P93 (nigrinodine) and P112 (sarmentine), which are both derived from piperamide-type compounds, represent strong negative loadings located in the left side of PC1 compared to other common features (Fig. 3B). This indicates the relative high content of both P93 and P112 in all organs except the leaves. The relative abundances of individual metabolites, corresponding to the most significant loadings (i.e. the most significant ones for the separation between the plant organs) are illustrated by boxplots as displayed in Fig. 4. The observed results are strongly supported and further detailed by PCA and OPLS-DA pairwise comparisons of metabolite profiles from different organs (See Fig. S3). This was performed for all organs resulting in 6 analyses (leaves/stems, leaves/roots, leaves/fruits, stems/roots, stems/fruits and roots/fruits). Beside the already described metabolites, variation within the groups is partly caused by unidentified features and artifacts (e.g. feature m226, t_R_ 10.4 min; *m/z* 230.2467 calculated for C_14_H_32_NO^+^).

Our results clearly indicate that production and accumulation of secondary metabolites is organ-specific [25, 36]. Additionally, as demonstrated by the differential responses of plant organs to environmental stressors, the stress adaptation responses are organ-specific [37]. This results in the existence of well-defined organ-specific patterns of metabolite accumulation, which facilitates interaction with the environment and helps to establish defense mechanisms with minimum expenditure of energy and valuable nitrogen containing compounds [38, 39]. Obviously, this factor affects the nutritional and medicinal value of the plant organs.

### Hierarchical clustering analysis

**Fig. 5:**
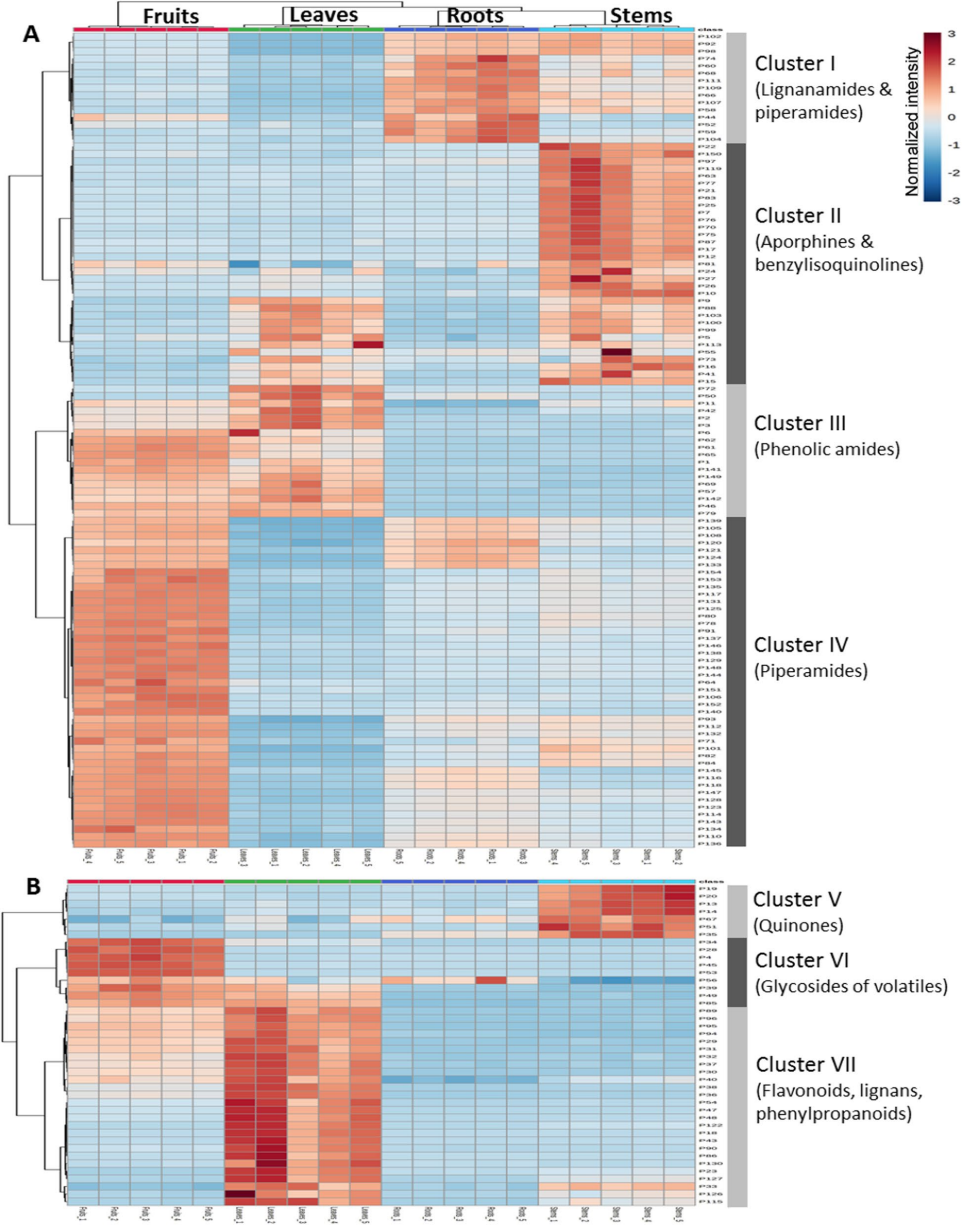
Hierarchical clustering with heatmap representation illustrating the relative inter-organ distribution of alkaloids (**A**) and flavonoids along with other metabolites (**B**), identified in different organs. The heatmap was established using a hierarchical clustering algorithm based on the similarities in abundances of aligned annotated metabolites. The red and blue color in the heatmap represent an increase and a decrease of metabolite levels compared to the average, respectively

Hierarchical clustering with heat map representation proved to be an excellent technique to address the distribution and relative abundances of the identified individual phytochemicals in different organs of *P. sarmentosum* (Fig. 5). The heatmap was constructed using a hierarchical clustering algorithm based on the inter-sample similarities in the relative abundances of all annotated metabolites (Fig. 5). Altogether, the hierarchical clustering was performed based on 154 annotated compounds with assigned structure from different organs belonging to the two major groups, alkaloids (**A**) and flavonoids including other metabolites (**B**). The individual replicates obtained from the same organ cluster nicely together indicating good reproducibility of results. The heatmap clearly shows the organ-specific accumulation of individual metabolites in leaves, stems, roots and fruits, indicating essential metabolic or storage differences between the *P. sarmentosum* organs which will influence their medicinal properties. Based on this dataset, seven clusters could be assigned including Cluster I-IV for alkaloids and Cluster V-VII for flavonoids and other metabolites (Fig. 5).

Cluster I is primarily composed of lignanamides (P58, P60, P68, P74) and specific piperamides such as piperettylines (P92, P98, P102) and piperettines (P109, P111) containing a benzodioxole group (type A and E). The accumulation of these compounds was found in the roots where they may serve as defense against feeding and pathogens. Significant levels of similar piperamides were recently also detected in cells distributed in the cortex of black pepper roots [8]. Several plants, including *Piper* species such as *P. puberulum* and *P. flaviflorum*, have been reported to contain lignanamides [40, 41]. However, no comparative analysis of lignanamides among plant organs has been reported. The general biosynthesis pathway of lignanamides in another plant species, *Cannabis sativa* L., has been discussed in the past. However, detailed mechanisms and molecular events throughout the biosynthesis of lignanamides remain inconclusive [42]. Lignanamides are natural products which are formed via an oxidative coupling mechanism involving hydroxycinnamic acid amides (HCAAs) as intermediates [43]. The formation of HCAAs is catalyzed by hydroxycinnamoyl-CoA:tyramine *N*-(hydroxycinnamoyl) transferease (THT), an enzyme characteristic for several plant species, including potato and tobacco [44]. The function of HCAAs in strengthening plant defense, as a response to fungal and pathogen challenge, has been described in potato tubers, onion roots and tomatoes [45].

Aporphines and benzylisoquinolines belong to the large group of isoquinoline alkaloids mainly present in Cluster II. Isoquinoline alkaloids such as magnoflorine (P12, P21), cepharadione A (P81), aristolactam BIII (P97), coclaurines (P9, P26), and reticuline (P16) are among the metabolites responsible for the formation of this cluster. These compounds had elevated levels in stems. Several studies found that most of these phytochemicals are abundant in specialized cell sites [46, 47]. For example, the analysis of isoquinoline alkaloids in the stems of *Sinomenium acutum* showed a differential distribution of alkaloids, especially within the outer cortical regions, phloem and xylem [46]. In opium poppy (*Papaver somniferum*), sieve elements and specialized laticifers of the phloem produce and accumulate isoquinolines alkaloids, respectively [48]. The co-localization to vascular tissue might explain the predominant occurrence in stems. Basically, these compounds are synthesized based on phenylpropanoids and amino acids, and the primary specific part of their biosynthesis is common to all these plants i.e., from norcoclaurine to reticuline. However, occasionally some mixed pathways may also occur to provide structural divergence [49]. In general, isoquinoline alkaloids play a role in defense against pathogens, and their ecological functions also appear to include plant-animal interactions [50–52].

Cluster III is featured with a high number of phenolic amides. This distinct class of metabolites accumulated predominantly in leaves and fruits. Some specific components, such as P50 (paprazine) and P72 were most abundant in leaves. Paprazine represents a tyramine derivative that contributes to active plant defense responses in infected leaf material. Biosynthesis of paprazine was induced in tomato leaves in response to wounding [53], and it was found to be associated with the resistance reactions of pepper leaves infected with *Xanthomonas campestris* [54]. According to Newman et al. [54], the up-regulation of phenolic amides (e.g. paprazine) in plant tissues was preceded by an increase in the extractable activity of tyrosine decarboxylase in parallel to the enhancement in the transcription of genes encoding phenylalanine ammonia-lyase and tyramine hydroxycinnamoyl transferase. This gene–gene interaction is accompanied by cell death, which is connected to a necrotizing reaction or hypersensitive response of the plant. Zacares et al. reported that large amounts of hydroxycinnamic acid amides such as paprazine and *trans*-*N*-feruloyltyramine were detected in tomato leaves after bacterial infection. This suggested that phenolic amides could play a key role in plant resistance vs. pathogens [55].

The most promising group of phytochemicals for medicinal and food applications in *Piper* species are piperamides, including piperine (P91), pellitorine (P116), retrofractamide B (P128) and guineensine (P136), which are classified into Cluster IV. Multiple metabolites representing this compound class (which actually contributes to the pungent taste of pepper) were detected in fruits, but not in the other organs. Similar occurrences of these constituents were also reported in fruits of other *Piper* species such as *P. nigrum* L. and *P. longum* L. [22, 56]. The observations of these constituents in *Piper* fruits were supported by the study of Schnabel and co-workers dealing with piperine synthase from *P. nigrum*. The authors propose that the accumulation of piperine and piperamides is related to the activity of piperine synthase (piperoyl-CoA: piperidine piperoyl transferase) and other BAHD-type enzymes that are preferentially expressed in fruits [57]. These secondary metabolites have a significant ecological impact on fruit-frugivore interactions and plant reproductive success [58].

Not less importantly, piperamides possess numerous advantages for human health, food applications, and feature potential agricultural prospects. For example, pellitorine, the major metabolite in this organ, was reported to possess insecticidal, anticancer, and antiplatelet properties [59]. In addition, piperine, a well-known compound derived from this species, has a wide range of biological activities and a distinct sharp flavor [60, 61]. Therefore, the fruits from *Piper* are predominantly used as food complements. Thus, the results obtained here provide insights in the perspectives for the use of *P. sarmentosum* fruits as an alternative to other *Piper* species.

Cluster VI is formed mostly by glycosides of volatiles such as P4, P28, P45, and P53, which are primarily found in fruits. The *Piper* fruits are particularly appreciated for their characteristics flavor and aroma that develops as fruits ripen [62]. A significant proportion of potential flavor contributors have been reported to exist as volatile compounds. Volatile aroma compounds are typically discovered in plants in two forms: "free" and "bound" to a sugar unit. These compounds are not odoriferous when bound; however, upon glycoside hydrolysis, these metabolites are liberated and then can be volatilized [63]. Moreover, the hydrolysis of glycosides may occur during the fruit ripening (ageing, senescence), or chewing/mechanical disruption of storage organs (e.g. vacuoles or lipid bodies, then combined with glycosidases from other cell compartments or cells), followed by the subsequent release of the volatile compounds that can olfactorily enhance the fruit flavor. Previous studies, e.g. such performed with the model plant *Arabidopsis thaliana*, show that glycosides are the result of glycosyltransferase activity. These enzymes add an activated sugar unit to the aglycone in the cytosol of the plant cell [64–66]. Recently, the analysis of the 7-deoxyloganetin glucosyltransferase-like (GGT) gene during fruit development of various *Piper nigrum* L. varieties revealed that increased GGT expression in *Piper* fruits correlated with up-regulation of volatile and nonvolatile metabolites [67]. Interestingly, some of the glycosides detected in this study contain 3-hydroxy-3-methylglutaryl residues as also found in flavonoids in this species (see chapter 2.4.2.1.)

Finally, Cluster VII is mostly represented by flavonoids (apigenin derivatives), including flavone-*C*-glycosides, and other phenolic metabolites such as phenylpropanoids and specific lignans. The compounds present in this cluster were found in relatively high amounts in leaves and are congruent with the constituents isolated from leaves of *P. sarmentosum* in our previous study [17].

The nature of flavonoid accumulation in plants indicates their important biological role. For instance, flavones are involved in a broad range of physiological functions in plant development and defense, such as protection from pathogen attack and in plant resistance to unfavorable environmental conditions like oxidative stress, high-level UV radiation and water/drought stress [68]. In general, the precursors for flavonoid biosynthesis are delivered by the phenylpropanoid pathway. The last steps of flavonoid biosynthesis involve various chemical modifications on the flavonoid aglycons, such as methylation, hydroxylation, glycosylation, and acylation, which increase their diversity and contribute to the final chemical properties [69]. Most of the enzymes involved in flavonoid metabolism are found in leaf mesophyll [70, 71]. This explains the greater content of flavonoids detected in the leaves in comparison to other organs. Leaves are the plant parts from *P. sarmentosum* which are preferentially consumed in Malaysia and other Asian countries, and which were shown to possess vascular protective, neuroprotective, anti-obesity, and anti-hyperlipidemia activity [18–20]. These activities might be connected to the content of flavonoids and lignans which possess a high antioxidative potential [72]. Previously, Miean et al. reported that the total flavonoid content including apigenin (TFC) of *P. sarmentosum* leaves can reach 120.5 mg/kg of dry weight [73]. According to Ugusman et al. [74], there is a positive correlation between the presence of flavonoids in *P. sarmentosum* leaves and its protective effects against oxidative stress. Other, earlier published studies reported that plant leaf extracts exhibited anti-inflammatory, antibacterial and insecticidal properties [10, 75, 76]. Simple phenylpropanoid compounds as asarone and its derivatives have an inhibitory effect on mosquitos and other insects, particularly larvae by inhibiting the hatching rates of eggs [77, 78].

**Fig. 6:**
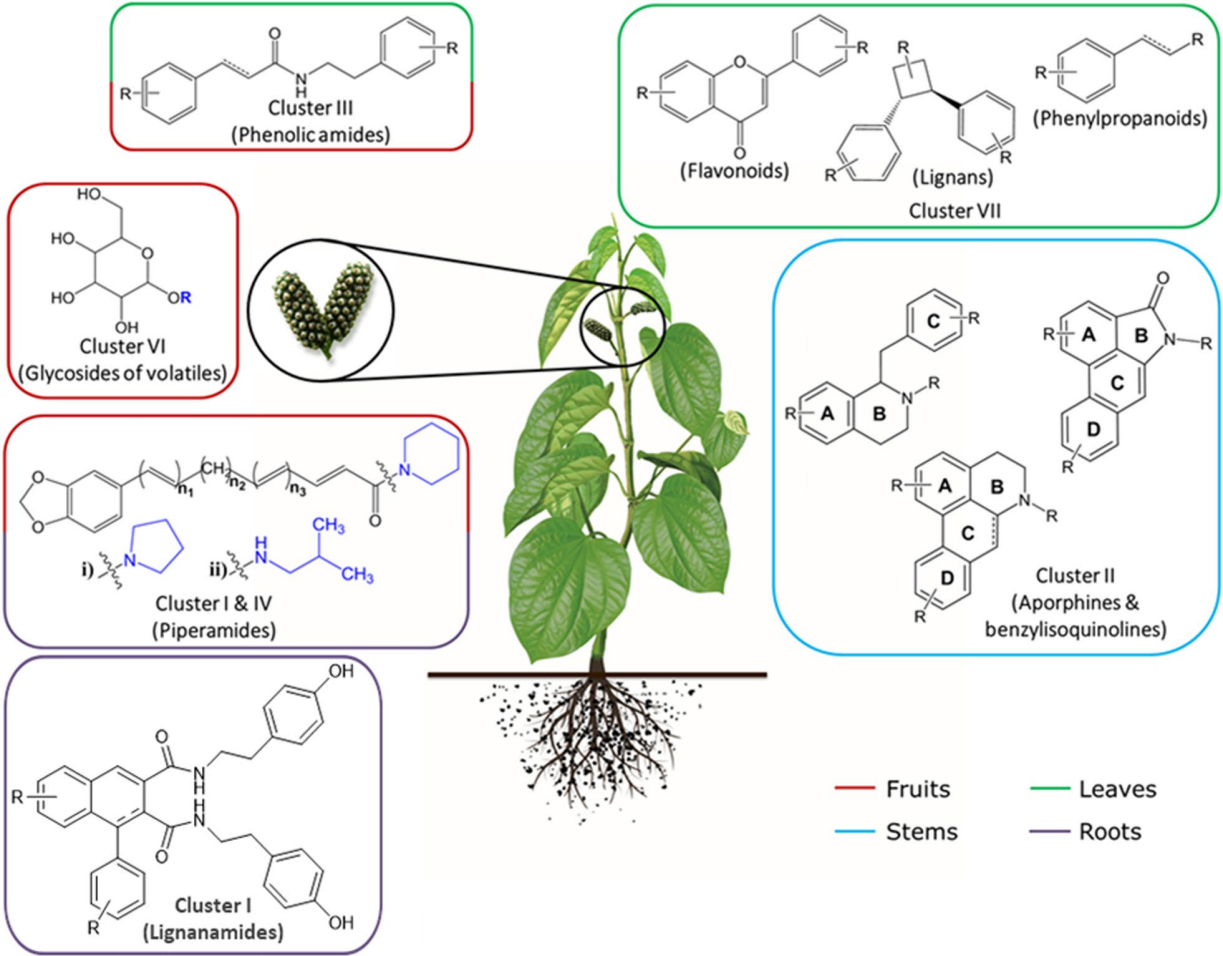
Predominant occurrence of compound classes in the organs of *P. sarmentosum*

Based on these data, the conclusion about the predominant occurrence of specific metabolite classes in different organs of *P. sarmentosum* is summarized in Fig. 6.

### Tentative identification of bioactives in *P.sarmentosum*

The untargeted metabolomic analysis which was used to characterize the metabolite composition of *P. sarmentosum* MeOH extracts revealed the differences between the plant organs. For annotation or identification of contributing constituents, their MS1 accurate monoisotopic ions were used to determine the likely elemental composition. As the mass accuracy of 5 ppm corresponded to the specification of the mass spectrometer used, this value was considered in the MS1 analysis. Moreover, a comparison of the fragmentation patterns with those reported in the literature and databases such as Reaxys and MassBank were performed to assign the compounds. The mass accuracy of fragment ions of 15 ppm was considered during the annotation process. This annotation strategy is well-established and is widely used [79–82]. In addition, isolated and fully elucidated compounds obtained from our previous work [17] were applied as reference compounds for the identification of individual metabolites by comparison of their *m/z* values, retention times and fragmentation patterns. Since structurally different compounds require different suitable collision voltages to obtain fragment information [83–85], a range of collision voltages between 15 and 60 V was used in the MS/MS analysis to identify metabolites.

As a result, a total of 154 compounds were annotated, either unambiguously identified by co-elution with authentic standards verified by NMR spectroscopy data or tentatively assigned by *m/z* values and MS/MS fragmentation patterns. The identified species were numbered according to the order of their elution time (Fig. 1 and Table 1 and S1). Alkaloids appeared to be the predominant metabolites, with 111 compounds annotated. In addition, 15 flavonoids and 28 other compounds were identified. Table 1 depicts the major organ-specific metabolites of *P. sarmentosum* characterized by both MS and MS/MS. The detailed list of all individual compounds annotated is summarized in Table S1.

**Table 1:** RP-UHPLC-QqTOF-MS/MS data of major and discriminating metabolites annotated in four organs of *P. sarmentosum* in positive ionization mode

Peak no	t_R_ (min)	Molecular formula	[M + H]^+^ [*m/z*]	Diff. (ppm)	MS^2^ *m/z* (key ions, rel. intensity [%]^a^)	CID (eV)	Annotation [ref.] (class)
P9	4.44	C_17_H_20_NO_3_^+^	286.1436	− 0.70	269.1179 (82), 237.0882 (5), 209.0960 (13), 175.0744 (m, 11), 145.0630 (4), 143.0473 (6), 137.0579 (3), 137.0579 (7), 107.0489 (k, 24)	20	Coclaurine [92] (Benzylisoquinoline)
P12	4.66	C_20_H_24_NO_4_^+^	342.1702	0.58	299.1289 (12), 297.1128 (**100**), 282.0888 (20), 265.0869 (75), 237.0904 (11), 58.0654 (38)	30	Magnoflorine [93] (Aporphine)
P21	5.00	C_20_H_24_NO_4_^+^	342.1705	1.46	297.1134 (**100**), 282.0895 (2), 265.0867 (78), 237.0907 (2), 58.0651 (3)	25	Magnoflorine isomer (Aporphine)
P30	5.69	C_27_H_31_O_15_^+^	595.1657	0.00	475.1249 (3), 433.1178 (89), 415.1050 (60), 397.0942 (37), 367.0829 (11), 337.0727 (20), 313.0748 (**100**), 283.0616 (12), 271.0617 (28)	40	Vitexin 2''-O-galactoside^b^ (Flavonoid)
P36	6.18	C_33_H_39_O_19_^+^	739.2074	− 0.81	577.1591 (51), 559.1480 (29), 475.1263 (57), 313.0730 (34), 271.0623 (9)	30	Vitexin 4''-glycoside^b^ (Flavonoid)
P70	9.60	C_16_H_12_NO_3_^+^	266.0813	0.38	2,510,580 (**100**), 223.0612 (9), 195.0679 (37), 168.0556 (7), 167.0728 (37)	40	Piperolactam A^b^ (Aporphine)
P75	10.11	C_17_H_14_NO_4_^+^	296.0922	1.69	281.0697 (44), 280.0514 (4), 266.0462 (6), 264.0670 (12), 263.0592 (74), 236.0707 (4), 235.0642 (**100**)	30	Piperolactam B (Aporphine)
P92	11.66	C_18_H_20_NO_3_^+^	298.1430	− 2.68	227.0715 (b, **100**), 199.0759 (d, 20), 197.0600 (19), 169.0657 (47), 161.0604 (g, 7), 159.0439 (3), 150.0910 (5), 141.0702 (19), 135.0429 (e, 1), 131.0490 (7), 98.0602 (c, 36), 72.0810 (a, 3), 70.0645 (1)	25	Piperettyline (Piperamides E)
P93	11.66	C_18_H_22_NO_3_^+^	300.1590	− 1.33	229.0956 (b, 4), 201.0904 (d, 10), 187.0747 (5), 171.0901 (1), 161.0596 (g, 73), 139.0982 (h, 7), 135.0433 (e, 8), 131.0488 (**100**), 124.0750 (7), 103.0537 (18), 98.0595 (c, 23), 72.0806 (a, 8)	25	Nigrinodine [94] (Piperamides E)
P95	11.87	C_12_H_17_O_3_^+^	209.1171	− 0.48	194.0950 (46), 181.0869 (24), 179.0726 (**100**), 178.0997 (26), 177.0914 (6), 163.0757 (18), 162.0678 (8), 151.0763 (73), 147.0790 (8), 136.0520 (17), 123.0447 (15), 121.0648 (20), 107.0495 (7), 91.0543 (15)	30	*trans*-Asarone^b^ (Phenylpropanoid)
P98	11.92	C_18_H_20_NO_3_^+^	298.1437	− 0.34	227.0728 (b, **100**), 199.0767 (d, 25), 197.0612 (25), 171.0934 (1), 169.0661 (53), 161.0633 (g, 8), 159.0444 (3), 150.0913 (8), 141.0705 (23), 135.0444 (e, 1), 131.0493 (10), 124.0760 (3), 115.0537 (3), 103.0542 (3), 98.0608 (c, 56), 72.0903 (a, 3), 70.0854 (1), 55.0547 (5)	25	Piperettyline (isomer-A) (Piperamide E)
P110	12.98	C_20_H_24_NO_3_^+^	326.1752	0.31	255.1031 (b, 4), 227.1069 (d, 5), 187.0754 (9), 165.1152 (h, **100**), 161.0594 (g, 32), 150.0905 (5), 135.0439 (e, 4), 131.0488 (29), 103.0538 (4), 98.0599 (c, 58), 72.0804 (a, 3), 70.0643 (1)	25	6,7-Dehydrobrachyamide B [95] (Piperamide E)
P112	13.14	C_14_H_24_NO^+^	222.1852	0.00	194.1906 (2), 168.1744 (1), 150.0918 (h, 15), 124.0761 (27), 110.0963 (15), 98.0604 (c, 71), 95.0492 (15), 91.0539 (3), 81.0342 (**100**), 72.0810 (a, 13), 70.0654 (14), 69.0698 (10), 67.0545 (29), 53.0390 (92), 55.0540(38)	40	Sarmentine (Pyrrolamide)
P114	13.39	C_20_H_26_NO_3_^+^	328.1898	− 2.74	229.1236 (d, 22), 199.1124 (7), 161.0600 (g, 11), 135.0449 (e, **100**), 131.0494 (7), 98.0602 (c, 22), 84.0804 (5), 72.0812 (a, 12)	30	Brachyamide B (Piperamide E)
P116	13.59	C_14_H_26_NO^+^	224.2006	− 1.34	168.1390 (j, 39), 151.1121 (b, 13), 123.1168 (d, 18), 112.0760 (13), 109.1010 (33), 98.0901 (46), 95.0496 (42), 93.0696 (12), 83.0855 (21), 81.0344 (61), 79.0538 (16), 74.0966 (a, 1), 69.0701 (54), 67.0544 (43), 57.0704 (i, **100**)	30	Pellitorine [56] (Piperamide C)
P122	14.45	C_24_H_33_O_6_^+^	417.2267	− 1.20	385.1992 (5), 249.1497 (**100**), 217.1229 (39), 209.1178 (29), 181.0860 (17)	15	Andamanicin^b^ (Lignan)
P135	16.29	C_24_H_32_NO_3_^+^	382.2363	− 3.66	311.1674 (b, 1), 283.1719 (d, 1), 213.0928 (1), 201.0920 (1), 187.0766 (4), 175.0760 (2), 161.0808 (g, 9), 135.0451 (e, **100**), 131.0500 (15), 103.0545 (3), 98.0606 (c, 26), 84.0812 (22), 81.0334 (2), 72.0812 (a, 5), 55.0545 (3)	35	Brachyamide A [22] (Piperamide E)
P136	16.40	C_24_H_34_NO_3_^+^	384.2525	− 2.08	311.1664 (b, 4), 283.1715 (d, 8), 187.0762 (4), 175.0758 (4), 161.0602 (g, 9), 135.0446 (e, **100**), 131.0491 (5), 123.0439 (4), 109.1010, 95.0851 (2), 86.0965 (18), 81.0994 (2), 74.0961 (a, 1), 57.0999 (3)	30	Guineensine [22] (Piperamide B)

^a^Relative intensity of parent ion is 100% if none of fragment ions stated. ^b^Confirmed by co-elution with authentic standards (isolated compounds with the structure confirmed by NMR spectroscopy) [17]

#### Alkaloids

**Fig. 7:**
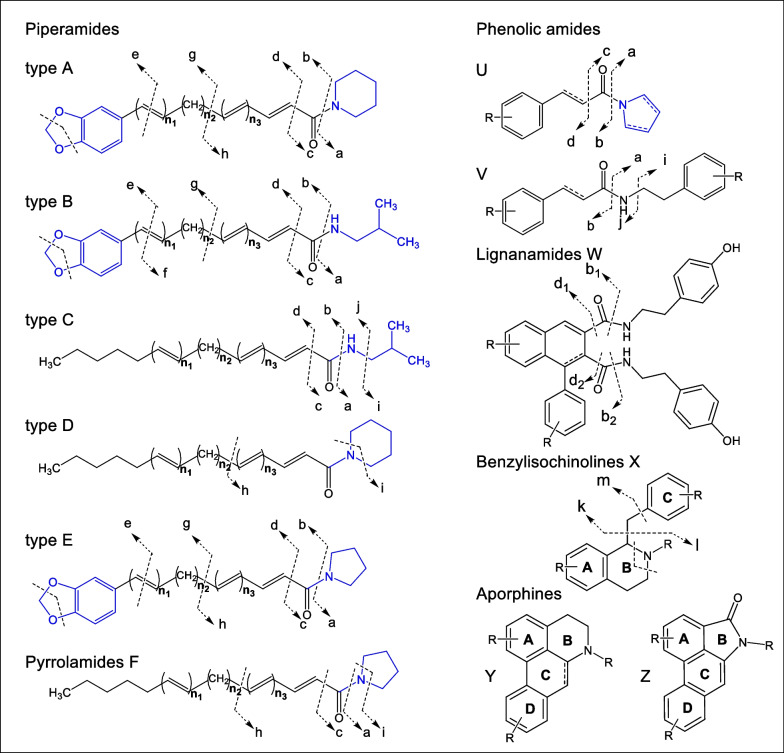
Classification of major alkaloid types found in *P. sarmentosum* and their characteristic fragmentation ions

*Piper* is a rich source of alkaloids, for which numerous biological activities have been reported [86, 87]. Furthermore, the quality of *Piper* species is usually assessed by their metabolite composition and the contents of some biologically active components [88]. In this study, a variety of alkaloids, mostly amides, were putatively identified in the organs of *P. sarmentosum.* According to their structure and MS breakdown pattern, piper alkaloids have been classified into types A-E in previous studies (Fig. 7) [22, 32, 89–91]. In addition to piperamides, other alkaloids such as phenolic amides (U and V), lignanamides (W), isoquinolines (X) and aporphines (Y, Z) were also classified, and the general characteristics of the fragmentation patterns were determined (Fig. 7). Structurally, piperamide types A, B and E have the same benzodioxol group at the terminal part of the acyl moiety of the amide bond, condensed with different amines such as piperidine, isobutylamine or pyrrolidine at the other terminus. In contrast, types C, D and pyrrolamides F have the benzodioxol group replaced by a short, saturated alkyl moiety.

In most of the cases, the analytes could be reliably assigned to the specific structural classes of alkaloids (mainly types A–E) by the signals of characteristic fragments in the MS/MS spectra. Indeed, all piperamides showed a typical cleavage of the N-CO bond resulting in the formation of an (aryl-) acylium cation (**b**) and the corresponding protonated amine part (**a**). This is often followed by further cleavage of CO leading to fragment **d**. Thereby, especially the specific neutral losses of the amine part give valuable structural information. Thus, the loss of piperidine (C_5_H_11_N, 85.0891 Da) and/or formylpiperidine (C_6_H_11_NO, 113.0841 Da) indicated the presence of type A or type D piperamides. The unique loss of isobutylamine (C_4_H_11_N, 73.0891 Da) and/or formylisobutylamine (C_5_H_11_NO, 101.0841 Da) referred to the presence of type B and type C piperamides. Similarly, the presence of a pyrrolidine unit was associated with the unique neutral losses of the amine moiety (C_4_H_9_N, 71.0735 Da) and/or formylpyrrolidine (C_5_H_9_NO, 99.0684 Da) (Fig. 7). The typical fragment ions, **g** at *m/z* 161 (C_10_H_9_O_2_^+^), **e** at *m/z* 135 (C_8_H_7_O_2_^+^), and **g** −CH_2_O at *m/z* 131, indicated the existence of a benzodioxole group. These features proved the structure of the terminal part of type A, B and E piperamides.

For instance, the pathways of the gas phase reactions under CID conditions for type A, B and E piperamides with molecular adduct ions [M + H]^+^ at *m/z* 314.1747 (P108), 302.1745 (P105) and 300.1590 (P93), respectively, were determined based on the MS/MS data as shown in Figure S4 in the supplementary material. The MS/MS data of these molecular ions show the same fragment ions at the *m/z* values of **b** (229), **d** (201), 187, **g** (161), **e** (135), **g** −CH_2_O (131) and 103 (**g** −CH_2_O −CO) in positive ion mode indicative for a benzodioxol group. The neutral losses are in accordance with the presence of a piperadine (P108), isobutylamine (P105) and pyrrolidine (P93) moiety. By matching the MS/MS spectra with those reported in the literature and databases, these compounds could be putatively identified as piperdardine (P108), chingchengenamide A (P105) and nigrinodine (P93), respectively [22, 56, 94]. Generally, the number of double bonds in all compounds could be calculated by the degree of unsaturation and their positions were estimated by the fragmentation losses and the literature data. Frequently detected isomers might result from *cis*/*trans* isomerization of the double bonds.

The detected phenolic amides, which included coumaroyl or feruloyl groups as well as various amino groups, generated fragment ions at *m/z* 147 and 177, respectively. Many of them contained a tyramine moiety (137 Da), accompanied with a loss of a NH_3_ moiety to form a vinylphenol ion **i** at *m/z* 121 [96]. Compound P50 displaying a [M + H]^+^ at *m/z* 284.1290 (C_17_H_18_NO_3_^+^, cal. 284.1281) in the MS^2^ spectrum was identified to be paprazine which produced fragment ions **b** at *m/z* 147 and **i** at *m/z* 121 (Fig. S5). The **b** ion at *m/z* 147 was yielded by the neutral loss of a tyramine unit. Whereas peak P55 displaying a [M + H]^+^ at *m/z* 314.1403 (C_18_H_20_NO_4_^+^, cal. 314.1387) was identified as *trans*-*N*-feruloyltyramine. The characteristics product ions at *m/z* 177 (**b**) and *m/z* 121(**i**) indicated the existence of feruloyl and tyramine moieties in the molecule (Fig. S5). The substances behind both peaks P50 and P55 were confirmed by isolated compounds.

Moreover, further alkaloid groups were detected and identified like aporphines and benzylisoquinolines. The aporphine group possesses a common isoquinoline skeleton in a highly conjugated tetracyclic structure and multiple methoxy, methylenedioxy, or hydroxyl substituents attached to this aromatic system conferring on the class a great structural diversity [97]. Most of the MS/MS fragmentation patterns of aporphine-type compounds contain characteristic neutral losses of the substituents on A and D rings and *N*-vicinal moieties through B-ring cleavage. Peak P12 with a molecular parent ion of *m/z* 342 [M + H]^+^ exhibited a major fragment ion at *m/z* 297 (C_18_H_17_O_4_^+^) which could be attributed to the elimination of (CH_3_)_2_NH, representing a key characteristic of the aporphines fragmentation pathway (Fig. S6). Subsequently, the fragment ions at *m/z* 282 (C_17_H_14_O_4_^●+^) and *m/z* 265 (C_17_H_13_O_3_^+^) were produced by the consecutive losses of ^●^CH_3_ and CH_3_OH, respectively, from the fragment ion at *m/z* 297. The subsequent removal of CO from the fragment ion at *m/z* 265 to *m/z* 237 (C_16_H_13_O_2_^+^) is described as the preferred fragmentation pathway of metabolites with methoxy and hydroxyl groups in ring A [92, 93, 98]. Thus, peak P12 with the molecular ion [M + H]^+^ *m/z* 342 was deduced as magnoflorine comparing with the reported data [93].

Figures 7 and S7 depict the MS fragmentation patterns of benzylisoquinoline-type skeletons, which strongly indicate the presence of key ions (**k**, **l**, **m**) and the loss of the NH group. The **k**-type ion represents the benzyl group and is generated on the MS/MS secondary to the formation of ion **l**. The **l** ion is produced under the MS/MS conditions after an “even electron”- type McLafferty rearrangement, involving the nitrogen proton and the aromatic ring of the benzyl conjugate [99]. The **m** ion occurs as a rearrangement after the elimination of the benzyl groups and amine moiety [92]. The *m/z* values of these key ions indicate the substituents present in the benzyl (ring C) and the isoquinoline part (ring A).

For example, the fragmentation of peak P16 (*m/z* 330) among the compounds listed in the group of benzylisoquinolines is shown in Figure S7. It was identified as a tertiary amine by the loss of 31 Da in the MS/MS spectrum resulting in an ion with *m/z* 299 ([M + H − CH_3_NH_2_]^+^). The ion **l** with *m/z* 192 indicates the vicinal OCH_3_ and OH group present at ring A. Furthermore, ion **k** with *m/z* 137 suggested the existence of OCH_3_ and OH on the benzyl moiety. This data was supported by the **m** ion of this compound, with *m/z* 175. Therefore, peak P16 was postulated as the well-known alkaloid reticuline in comparison with literature data [92].

#### Flavonoids

Flavonoids are referred to as a large group of polyphenol natural products with many members possessing various biochemical properties, such as antimicrobial, anti-inflammatory and anti-cancer activities. They serve as antioxidants, UV-protectants and play a pivotal role as coloring components of flowering plants [100]. MS/MS analysis can provide structural information about flavonoids including the structure of aglycones, glycosylation patterns and the presence of other substituents such as 3-hydroxy-3-methylglutaryl and other acyl residues [101]. According to previous studies of *P. sarmentosum*, the major identified flavonoid aglycone was apigenin (aglycon *m/z* 271) [102]. Several types of flavonoid substitutions have also been reported, such as *C*-glycosyl and* O*-glycosyl flavones with mono- and di-substituted glycosides [74, 103].

In the present study, a total of 15 compounds could be annotated as flavone derivatives, which were variously *C*-glycosylated and substituted with 3-hydroxy-3-methylglutaryl and other acyl residues. The annotation relied on comparison of their accurate monoisotopic masses and unique fragments with databases, published in literature reports and spectral data of isolated compounds. All compounds were assigned to possess an apigenin aglycone based on the corresponding fragment peaks appearing at *m/z* 313 (C_17_H_13_O_6_^+^) and *m/z* 271 (C_15_H_11_O_5_^+^). The most relevant flavonoids identified are summarized in Table 1 and Table S1.

**Fig. 8:**
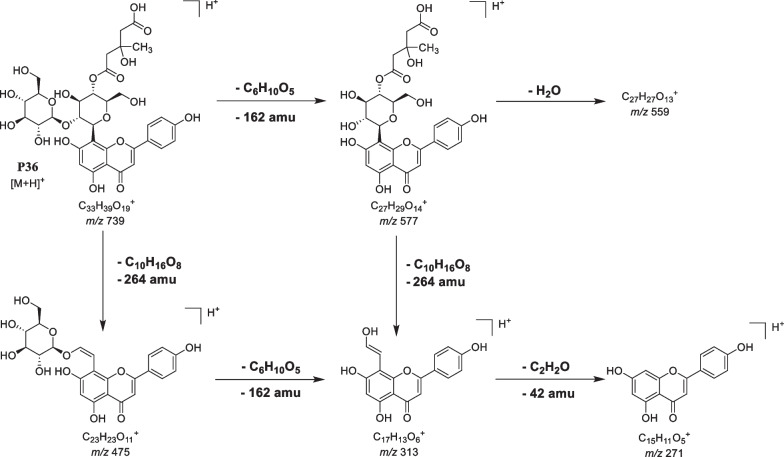
Fragmentation scheme of P36 (Vitexin 4ʺ-(3-hydroxy-3-methylglutaroyl)-2ʺ-O-*β*-D-glucopyranoside)

The fragmentation behavior of a 3-hydroxy-3-methylglutaryl residue (HMG) attached to a sugar moiety is in positive ion mode mainly characterized by diagnostic ions of [M + H–264]^+^, [M + H–162]^+^, and/or [M + H–102]^+^ [104, 105]. In contrast, in the negative ion mode, the separate neutral loss of the HMG moiety is visible as [M–H–144]^−^ (Figure S10_36b and 38b). Peaks P36, P38 and P39 were consistent with the presence of an HMG substituent on the saccharide residue. For example, the MS/MS spectra of the peak P36 (*m/z* 739.2130, C_33_H_39_O_19_^+^) showed fragment ions at *m/z* 577, 559, 475, 313 and 271 as explained in Fig. 8. The fragment ions are resulting from neutral losses of 264 and 162 Da indicating the simultaneous loss of HMG and glucose residues. The abundant ion at *m/z* 475 ([M + H − 264]^+^) is further decomposed by the loss of a second C_6_H_10_O_5_ unit leading to ions at *m/z* 313 and *m/z* 271. The ion at *m/z* 313 is indicative for flavonoid *C*-glycosides. The identity of peak P36 (Vitexin 4ʺ-(3-hydroxy-3-methylglutaroyl)-2ʺ-O-*β*-D-glucopyranoside) was confirmed by comparison with the t_R_ and fragment ions of this compound previously isolated for the first time from leaves of *P. sarmentosum* [17].

The most common acyl groups generally found as substituents of flavonoids are hydroxycinnamic acids such as caffeic, ferulic, and sinapic acid. The characteristic product ions of these were previously reported [105–107]. Based on the characteristic ion fragments and neutral losses observed for this type of flavonoid, four acyl containing compounds linked to glycosides (P43, P47, P48 and P54) were identified. Peak P48 and P54 (Fig. S8), for example, are isomers which both have a molecular weight of 914 Da. The characteristic ions at *m/z* 339 (C_16_H_19_O_8_^+^) and 177 (C_10_H_9_O_3_^+^) and the neutral losses of 264 and 162 suggest that both compounds contain feruloyl and HMG substituents. However, the relative intensity of the ion *m/z* 339 was higher for peak P48 (100%) than for P54 (19%), indicating for the former (P48) that the feruloyl moiety is linked to the other hexose unit in the diglycoside. In contrast, the feruloyl-glycoside of P54 might occur on the *C*-6 of flavone due to the lower relative intensity of the signal at *m/z* 339. These *C*-6 isomers are often eluted later than the corresponding 8-*C*-glycosides. To the best of our knowledge, acylated *O*-glycosyl flavones with two groups have not yet been described in the genus *Piper*.

#### Other metabolites

Another 28 compounds were classified as asarones, coumarins, quinones, lignans and their glucoside derivatives. As a typical representative, the MS/MS fragmentation of peak P95 was investigated as shown in Figure S9. Its protonated molecular ion was *m/z* 209.1182 in positive ion mode, and its main fragment appeared at *m/z* 179.0726 [M + H − 30]^+^ corresponding to the loss of C_2_H_6_. The radical fragment *m/z* 194.0950 [M + H − 15]^+^ was yielded through demethylation. The fragment ion at *m/z* 151.0763 [M + H − 28]^+^ was produced by decarboxylation of a fragment at *m/z* 179. Peak P95 represented the most abundant peak in *P. sarmentosum* which could be detected in all organs. The t_R_ and fragmentation pattern of P95 at *m/z* 209.1182 [M + H]^+^ were consistent with the isolated compound *trans*-asarone.

## Conclusions

In this study, RP-UHPLC-QqTOF-MS and MS/MS were used to characterize separately the metabolic profiles of leaves, stems, fruits and roots from the traditional medicinal plant *P. sarmentosum.* As a result, altogether 154 secondary metabolites were annotated. The assigned compounds were dominated by diverse alkaloids together with flavonoids and lignans. The individual MS fragmentation patterns allowed the distinct attribution to compound classes while specific substitution patterns or stereochemical information remained uncertain. In addition, the relative abundances of these metabolites in various organs were determined. Roots and fruits shared many common phytochemicals, especially piperamide types A-E, as evidenced by TICs, PCA score and loadings plots, and hierarchical clustering analysis. On the one hand, this finding indicates the similarity to other *Piper* fruits and the potential economic, medicinal, and nutritional value of *P. sarmentosum.* On the other hand, it supports the culinary application of roots by some communities in southern China. Stems generally contained the most significant amounts of aporphine alkaloids compared with other organs. In contrast, the leaves contained relatively high amounts of flavonoids. Several compounds could be identified as unique metabolites to distinguish different *P. sarmentosum* organs. The results obtained in this study provide valuable insight into the distribution of bioactive compounds in different *P. sarmentosum* organs and could also maximize the plant’s economic value as herbal medicine, for drug discovery or in food applications. As for the plant it is yet to be determined what the ecological role of the distribution of piperamides especially in root and fruit is. Since fruits (seeds) at some point hit the ground for germination, the constituents may serve the same purpose, e.g. as deterrent for feeding animals or invertebrates. Also, it remains yet unresolved to what extent they are produced independently in the two organs or transported. However, if in analogy to *P. nigrum* where special cells perform the piperamide synthesis, they likely are produced independently in both organs [8]. In any case, our finding shows, that separate harvesting and utilization of *P. sarmentosum* organs is relevant to the composition of any concoction thereof, be it for food or medicinal purposes.

## Experimental section

### Chemicals

All chemical standards were obtained from Merck (Darmstadt, Germany). Methanol and acetonitrile (both LC–MS CHROMASOLV™) were purchased from Riedel-de Haёn/Honeywell, (Seelze, Germany), ammonium formate (≥ 99%, MS eluent additive) was obtained from Sigma Aldrich (Steinheim, Germany), formic acid (LC–MS grade, 98–100%) from Fluka™/Honeywell (Seelze, Germany). Water was purified in-house on the water conditioning and purification system Millipore Milli-Q Gradient A10 (resistivity > 18 mΩ/cm, Merck Millipore, Darmstadt, Germany).

### Plant material and extraction

*Piper sarmentosum* Roxb. was collected in January 2019 from Negeri Sembilan, Malaysia. A voucher was authenticated (Number: MFI 0039/19) by Dr. Mohd Firdaus Ismail, a botanist at the Institute of Biosciences, Universiti Putra Malaysia. A duplicate of the Herbarium specimen is kept at the Department of Bioorganic Chemistry in the Leibniz Institute of Plant Biochemistry, Halle/Saale, Germany. Various plant organs such as leaves, stems, roots and fruits were collected from different plants. After the organs were sorted, samples were pooled, cleaned, and dried in the oven at 50 °C for 92 h. The dried samples were ground in a Mixer Mill MM 400 with a 20 mm stainless steel ball (Retsch, Haan, Germany) at a vibration frequency of 30 Hz for 60 s. Briefly, for sample preparation 4 mg dried plant organs were weighed in a 2 mL centrifuge tube. Then 1 mL of methanol containing 2 pmol/µL of kinetin and 50 pmol/µL orcinol as internal standards were added, the tubes were vortexed vigorously for 20 s, and extraction was performed by sonication in the ultrasound bath (40 °C) for 20 min. The extracted samples were centrifuged for 15 min at 14,000 × *g* and clear supernatants were transferred to LC–MS vials for analysis. Equivalent volumes of the extracts obtained from different organs were pooled. The aliquots (50 µL) of this pool were used as quality controls (QCs) to characterize the instrument and sample stability. Two QC samples were injected after every six injections of the randomized sample sequence during the RP-UHPLC- QqTOF MS analysis.

### LC–MS and MS/MS

Analysis of the extracts relied on reversed phase-ultra high-performance liquid chromatography (with 3 mmol/L aq. ammonium formate and acetonitrile as eluents A and B, respectively) coupled on-line to electrospray ionization quadrupole-time-of-flight mass spectrometry (RP-UHPLC-QqTOF-MS). For this, 5 µL of individual extracts were injected in a Waters ACQUITY I-Class UPLC System (Waters GmbH, Eschborn, Germany). After a two-min wash at 5% eluent B, the samples were separated at the flow rate of 400 µL/min on an EC 150/2 NUCLEOSHELL RP C18 column (150 × 2 mm, particle size 2.7 µm) thermostated at 40 °C in a linear gradient to 95% eluent B in 17 min. After a 2 min isocratic step at 95% eluent B, the column was re-equilibrated at 5% eluent B during 9 min. The column effluents were infused via an ESI part of DuoSpray™ Ion Source in a hybrid QqTOF mass spectrometer Sciex TripleTOF 6600 LC–MS System (AB Sciex, Darmstadt, Germany), operated in positive sequential window acquisition of all theoretical mass spectra (SWATH) mode and controlled by Analyst™ TF Software 1.8. The source was hold at 450 °C with nebulizer (GS1), drying (GS2) and curtain (CUR) gases set to 60, 70 and 55 psig, respectively, whereas the ion spray voltage was 5.5 kV. The SWATH experiments were performed with 1.11 s period time and comprised one TOF–MS scan and 48 product ion scans in the *m/z* range of 65 – 1250. The data were acquired at the pulser frequency of 19.1 Hz and the accumulation times of 100 and 20 ms for TOF–MS and product ion scans, respectively. The MS/MS data were obtained in 48 SWATH windows of 25 m*/z* each with collision potential (CE), collision energy spread (CES) and declustering potential (DP) set to 45, 35 and 35 V in positive mode. The targeted MS/MS data were acquired in positive ion mode at CEs of 15, 20, 30, 40, 50 and 60 V with CES set to 0 V and DP set to 35 V (total cycle time 0.9 s, 16 scans per cycle, scan accumulation time 50 ms, starting from *m/z* 50).

### Data processing and post-processing

The acquired chromatograms and spectra were examined in PeakView TM software (version 2.2) to accesses data quality and to adjust the settings for further processing. Deconvolution of the mass spectra, peak picking, retention time-based alignment, feature filtering and quantitation of individual signals in extracted ion chromatograms (XICs), i.e. integration of peak areas, were accomplished in MSDial software (version 4.12) after conversion of the raw data in ABF (analysis base file) format. The optimized peak detection parameters for processing in MSDial were set as follows: MS1 tolerance—0.015 m*/z*, MS2 tolerance—0.025 m*/z*, minimum peak width—5 s, minimum peak height—2000 counts, for alignment: retention time (t_R_) tolerance—0.15 min, MS1 tolerance—0.015 m*/z*.

Relative quantitation relied on integrated peak areas as intensity values of corresponding analytes. For this, the integrated peak areas of the metabolites annotated in each sample were organized as a digital matrix which was subjected to processing and statistical interpretation by means of the MetaboAnalyst 5.0 online platform (available for free at www.metaboanalyst.ca). Prior to the statistical analysis, the data were filtered to remove the features which were detected in less than 20% of the samples. Imputation of missing values relied on the MetImp algorithm (https://metabolomics.cc.hawaii.edu/software/MetImp/) and the random forest method for analytes detected in at least 80% of samples. Variables of the data sets were pareto-scaled and median normalized before multivariate analysis. Multivariate data analysis, including principal component analysis (PCA), OPLS-DA and hierarchical clustering were used to address intra- and inter-group variability and to assess the underlying group separation in the data.

### Metabolite annotation

Metabolites were annotated based on their corresponding accurate *m/z*, chromatographic retention times and similarity of the acquired tandem mass spectrometric (MS/MS) fragmentation patterns with those reported in the literature. Additionally, spectral libraries and in-silico fragmentation tools (e.g. MetFrag, MassBank—see https://www.ipb-halle.de/en/infrastructure/databases-and-tools/?MP=6-1058—and in-house library based on authentic reference standards) were employed for analyte annotation or identification. Briefly, the criteria for identification were as follows: the t_R_ was ± 0.1 min of reference standard, the mass error of MS1 was below or equal to 5 ppm, spectrum similarity match of the MS/MS fragmentation patterns.

### Supplementary Information

Supplementary Material 1. Figure S1: Comparison of the total ion chromatograms (TICs) acquired from methanolic extracts of different *P. sarmentosum* organs in the negative ion mode; Figure S2: PCA scores plot showing the close clustering of quality control samples (QC); Figure S3: The PCA and OPLS-DA plots derived from pairwise comparisons of metabolite profiles from different organs; Figure S4: The general fragmentation patterns of selected piperamides P108 (*m/z* 314), P105 (*m/z* 302), and P93 (*m/z* 300); Figure S5: Key characteristics product ions b and i of paprazine (P50), and *trans*-*N*-feruloyltyramine (P55); Figure S6: MS/MS spectrum of magnoflorine (P12); Figure S7: Proposed fragmentation pathway and MS2 spectra of reticuline (P16); Figure S8: Proposed structures of flavone derivatives giving peaks P48 and P54 with characteristic fragment ion at *m/z* 339; Figure S9: MS/MS spectral fragmentation of the [M + H]^+^ ion of *trans*-asarone (P95); Figure S10: MS/MS spectral fragmentation of the peak P1– P154; Table S1: RP-UHPLC-QqTOF-MS/MS data of metabolites annotated in four organs of *P. sarmentosum* in positive ionization mode.

## Data Availability

The data presented in this study are available on request from the corresponding author.
