# Supplementary material for: Comparative metabolite analysis of Piper sarmentosum organs approached by LC–MS-based metabolic profiling
Source: Nat Prod Bioprospect. 2024 May 14;14(1):30. doi: 10.1007/s13659-024-00453-z (PMC11093948; doi:10.1007/s13659-024-00453-z)
Supplement: Supplementary file 1 — Supplementary Material 1. Figure S1: Comparison of the total ion chromatograms (TICs) acquired from methanolic extracts of different P. sarmentosum organs in the negative ion mode; Figure S2: PCA scores plot showing the close clustering of quality control samples (QC); Figure S3: The PCA and OPLS-DA plots derived from pairwise comparisons of metabolite profiles from different organs; Figure S4: The general fragmentation patterns of selected piperamides P108 (m/z 314), P105 (m/z 302), and P93 (m/z 300); Figure S5: Key characteristics product ions b and i of paprazine (P50), and trans-N-feruloyltyramine (P55); Figure S6: MS/MS spectrum of magnoflorine (P12); Figure S7: Proposed fragmentation pathway and MS2 spectra of reticuline (P16); Figure S8: Proposed structures of flavone derivatives giving peaks P48 and P54 with characteristic fragment ion at m/z 339; Figure S9: MS/MS spectral fragmentation of the [M + H]+ ion of trans-asarone (P95); Figure S10: MS/MS spectral fragmentation of the peak P1– P154; Table S1: RP-UHPLC-QqTOF-MS/MS data of metabolites annotated in four organs of P. sarmentosum in positive ionization mode. [file 13659_2024_453_MOESM1_ESM.pdf]

Supplementary materials

Comparative metabolite analysis of *Piper sarmentosum* organs approached by LC-MS-based metabolic profiling

Ismail Ware[a,b,c], Katrin Franke[a,d,e*], Andrej Frolov[a], Kseniia Bureiko[a], Elana Kysil[a], Maizatulakmal Yahayu[c], Hesham Ali El Enshasy[c,f] and Ludger A. Wessjohann[a,e*]

[a] Department of Bioorganic Chemistry, Leibniz Institute of Plant Biochemistry, 06120 Halle (Saale), Germany
[b] Biotechnology Research Institute, Universiti Malaysia Sabah, Jalan UMS, Kota Kinabalu 88400, Sabah, Malaysia
[c] Institute of Bioproduct Development, Universiti Teknologi Malaysia (UTM), 81310 Johor Bahru, Johor, Malaysia
[d] Institute of Biology/Geobotany and Botanical Garden, Martin Luther University Halle-Wittenberg, 06108 Halle (Saale), Germany
[e] German Centre for Integrative Biodiversity Research (iDiv) Halle-Jena-Leipzig, 04103 Leipzig, Germany
[f] City of Scientific Research and Technology Applications, New Borg Al Arab, Alexandria 21934, Egypt

***** Correspondence: Katrin Franke, kfranke@ipb-halle.de, Tel. +49-345-5582-1380
Ludger A. Wessjohann, wessjohann@ipb-halle.de, Tel. +49-345-5582-1300

TABLE OF CONTENTS **PAGE**

Figure S1: Comparison of the total ion chromatograms (TICs) acquired of *P. sarmentosum* organs in the negative ion mode 2

Figure S2: PCA scores plot showing the close clustering of quality control samples (QC) 3

Figure S3: PCA and OPLS-DA plots derived from pairwise comparisons of metabolite profiles from different organs 4

Table S1: RP-UHPLC-QqTOF-MS/MS data of metabolites annotated in four organs of *P. sarmentosum* in pos. ionization mode. 6

Figure S4: The general fragmentation patterns of selected piperamides P108 (*m*/*z* 314), P105 (*m*/*z* 302), and P93 (*m*/*z* 300).......... 15

Figure S5: Key characteristics product ions **b** and **i** of paprazine (P50), and *trans*-*N*-feruloyltyramine (P55). 15

Figure S6: MS/MS spectrum of magnoflorine (P12).......... 16

Figure S7: Proposed fragmentation pathway and MS^2 spectra of reticuline (P16). 16

Figure S8: Proposed structures of flavone derivatives giving peaks P48 and P54 with characteristic fragment ion at *m*/*z* 339. 17

Figure S9: MS/MS fragmentation of the $[M+H]^+$ ion of *trans*-asarone (P95). 17

Figure S10: MS/MS spectra of the peaks P1–P154 18-98

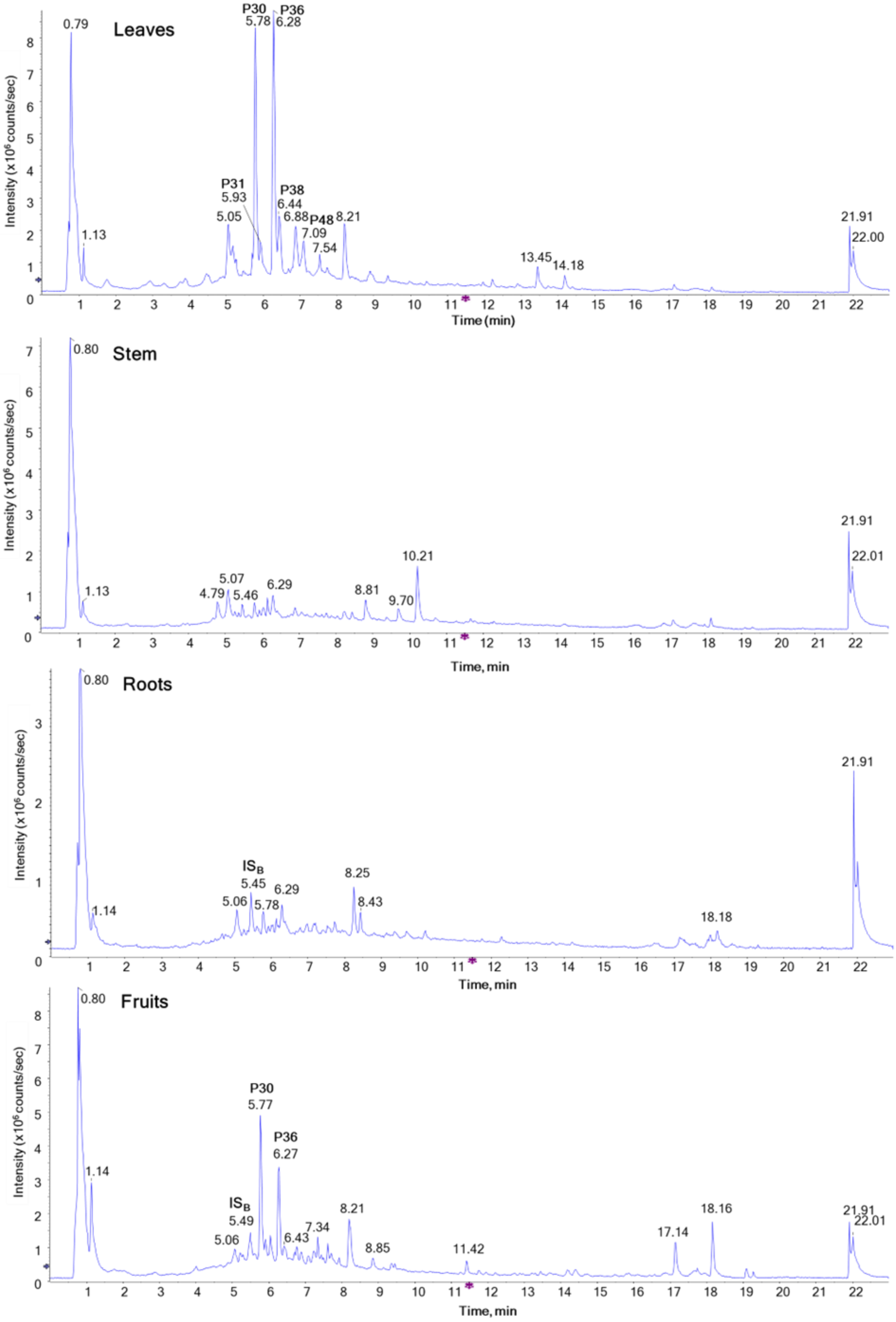

Figure S1: Comparison of the total ion chromatograms (TICs) acquired from methanolic extracts of different *P. sarmentosum* organs in the negative ion mode**:** IS$_B$ = internal standard kinetin, P = peak number.

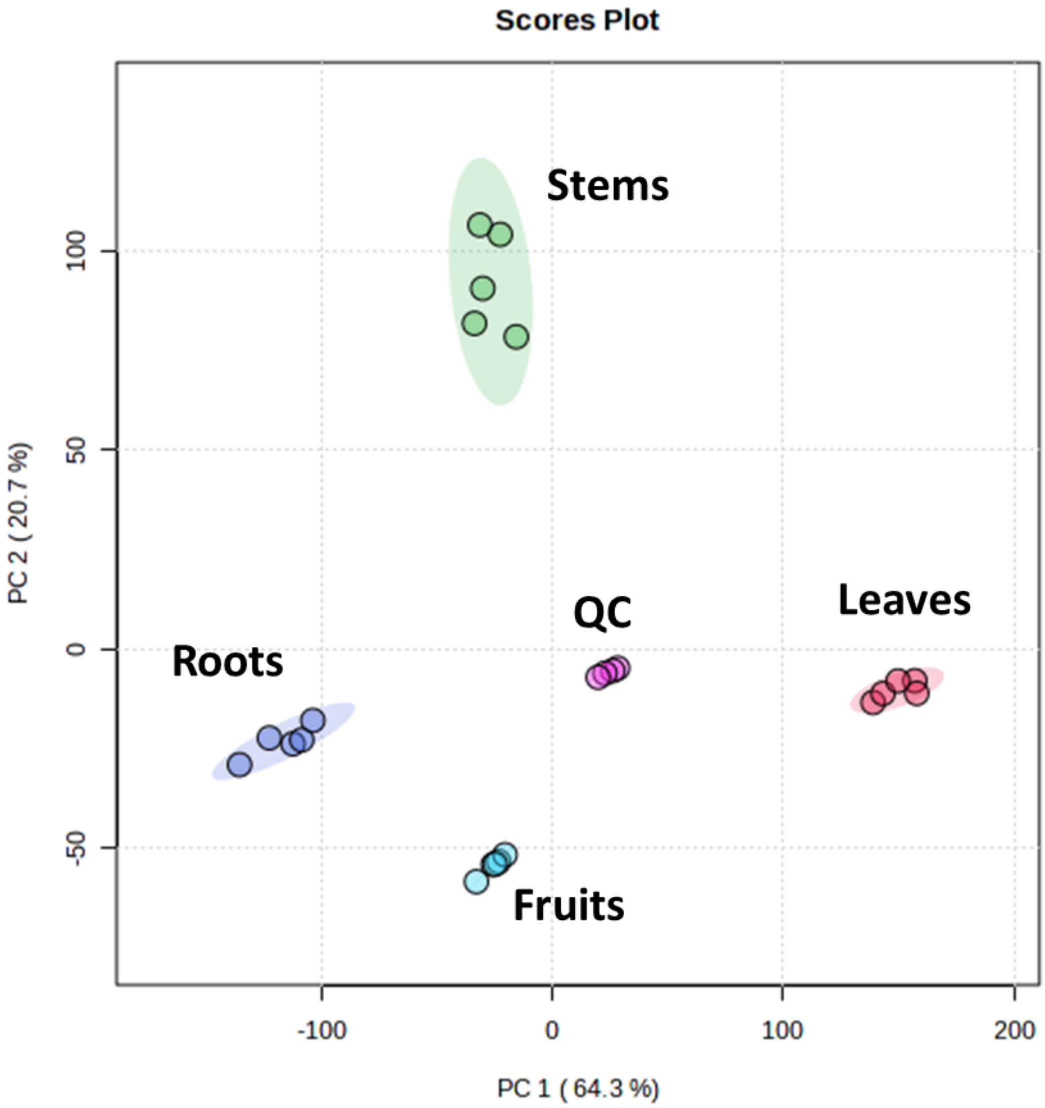

Figure S2: PCA scores plot showing the close clustering of quality control samples (QC).

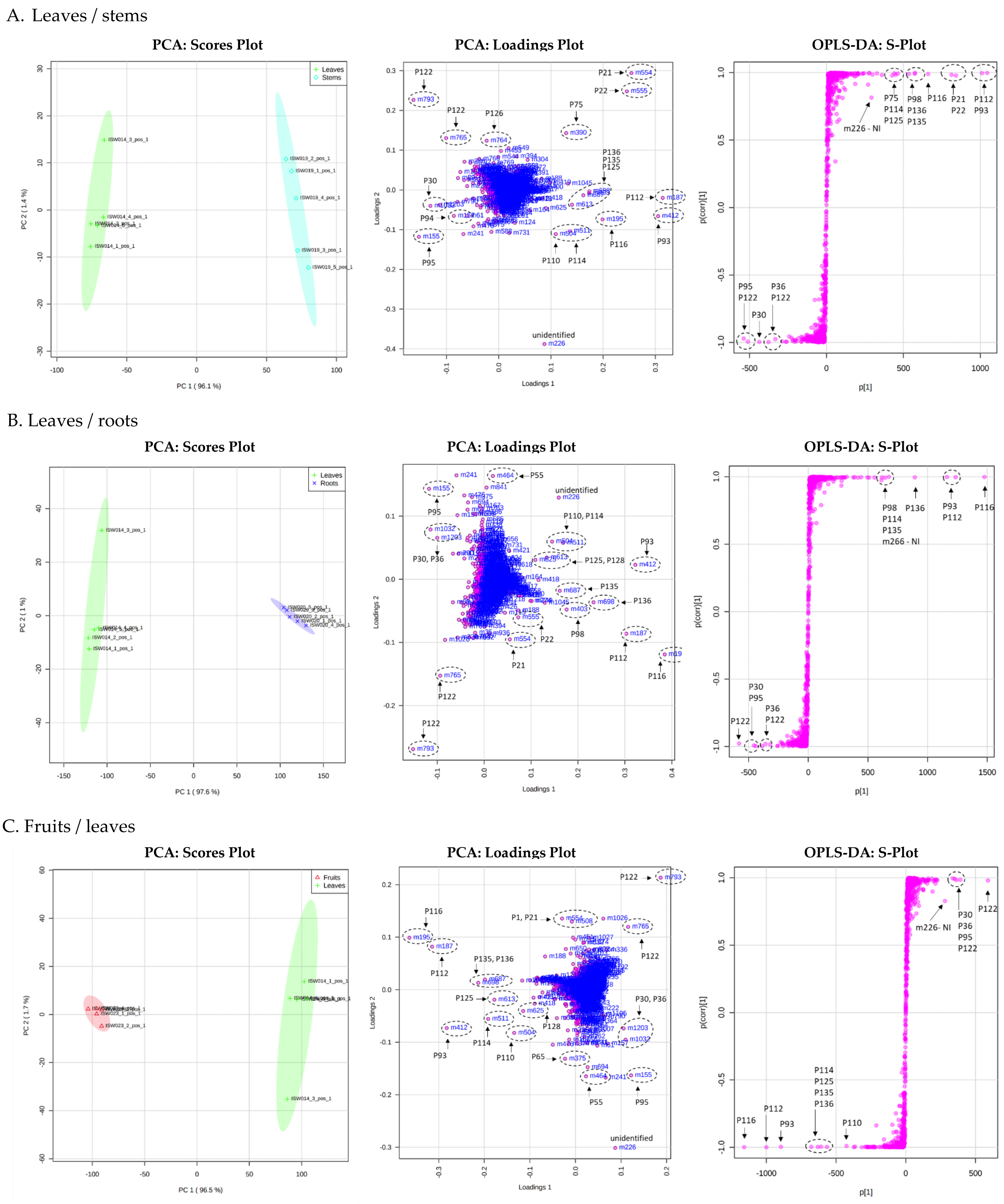

Figure S3: *Cont.*

D. Roots / stems

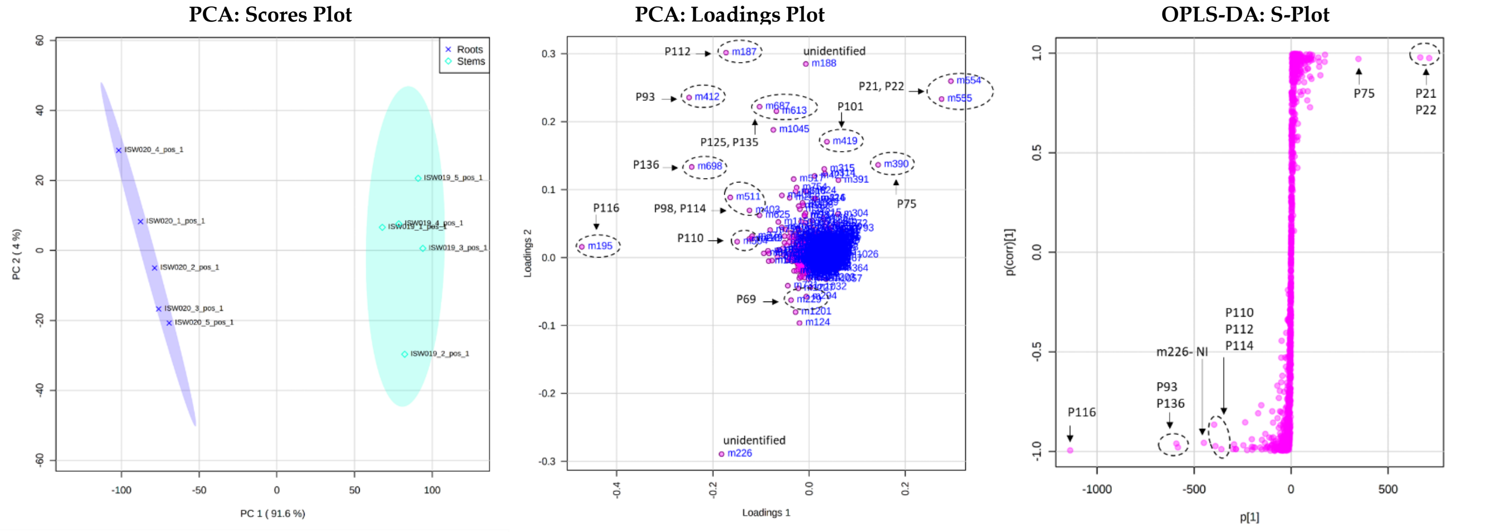

E. Fruits / roots

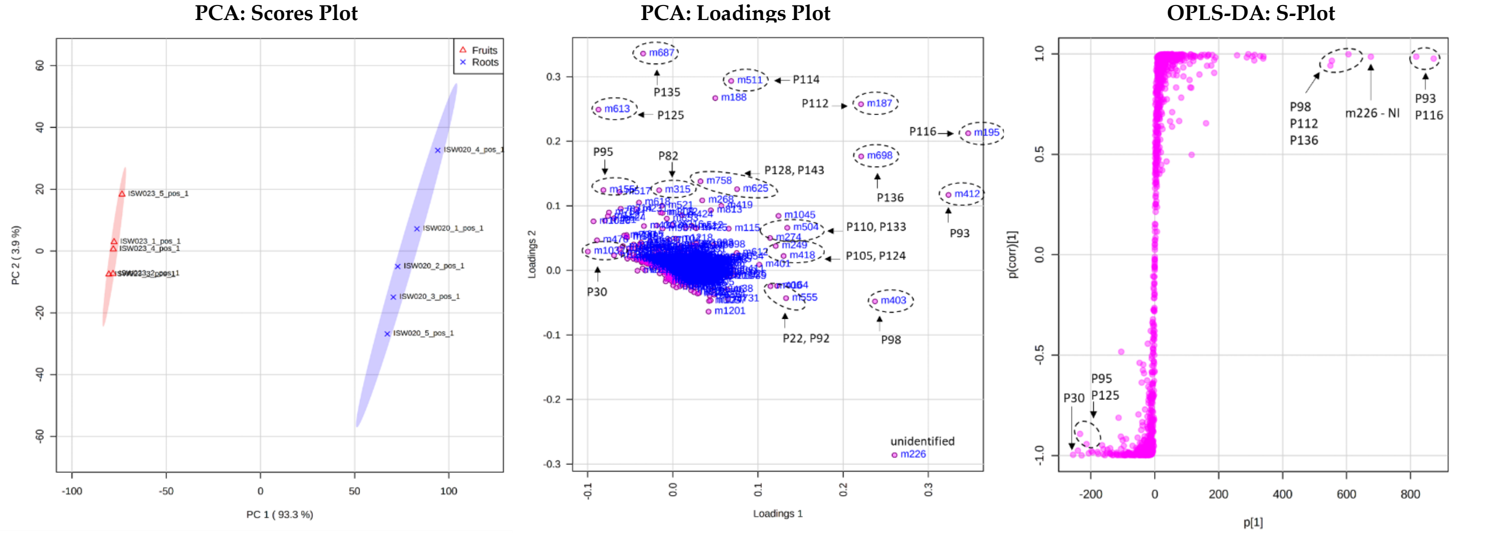

F. Fruits / stems

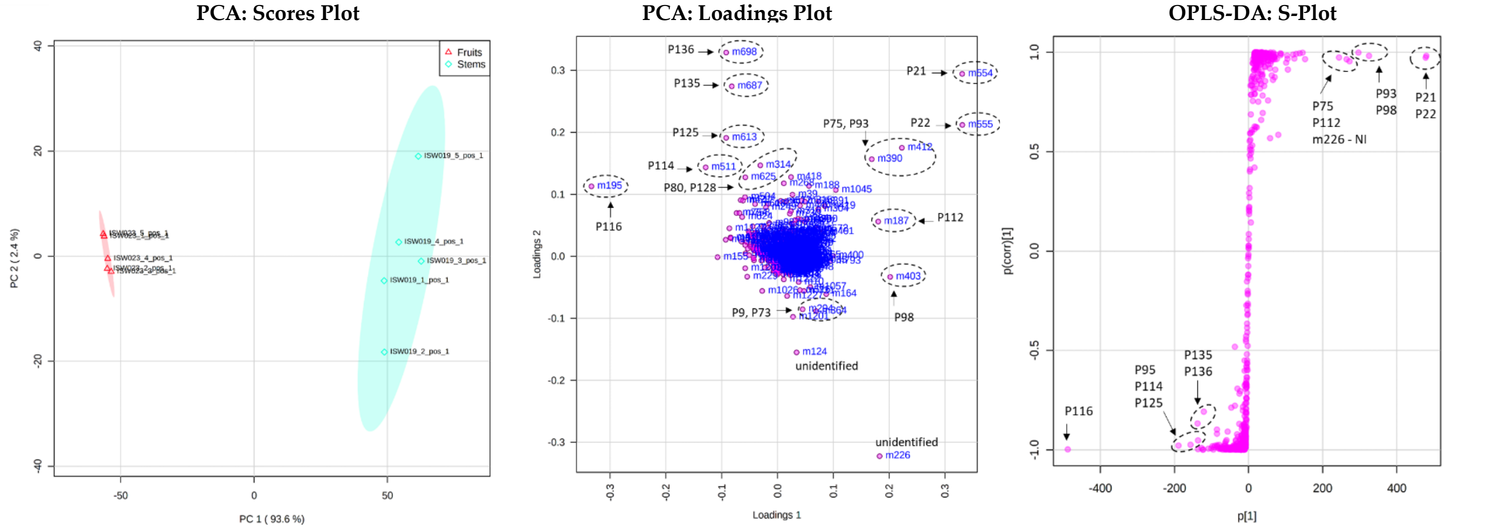

Figure S3: PCA and OPLS-DA plots derived from pairwise comparisons of metabolite profiles from different organs of *P. sarmentosum*.

Table S1:
RP-UHPLC-QqTOF-MS/MS data of metabolites tentatively annotated in organs of *P. sarmentosum* in positive ionization mode.

Peak no.	t_R (min)	Molecular formula	Precursor ion	[*m/z*]	Diff. (ppm)	[a]MS^2, [*m/z*] *m/z* (key ions, rel. intensity [%])	CID, eV	Annotation (class)
**Alkaloids**								
P1	1.71	$C_{15}H_{22}NO_7^+$	M+H	328.1386	-1.52	310.1295 (**100**), 292.1201 (28), 282.1356 (3), 264.1231 (10), 246.1109 (2), 178.0861 (10), 166.0857 (10), 132.0808 (3), 120.0811 (7).	20	Fructosyl-phenylalanine
P2	2.59	$C_{11}H_{10}NO_2^+$	M+H	188.0706	0.00	146.0603 (**100**), 144.0808 (18), 118.0657 (28)	20	Naphthisoxazol A[b]
P3	2.60	$C_{11}H_{13}N_2O_2^+$	M+NH_4	205.0971	-0.49	188.0708 (**100**), 170.0585 (1), 159.0905 (4), 146.0592 (16), 144.0805 (5), 132.0791 (2), 130.0640 (1), 118.0636 (4)	15	Naphthisoxazol A
P5	4.09	$C_{19}H_{24}NO_3^+$	M+H	314.1751	0.00	299.1215 (21), 298.1066 (15), 271.1321 (19), 269.1173 (39), 237.0909 (19), 209.0942 (18), 175.0750 (m, 32), 145.0620 (8), 137.0590 (15), 107.0482 (k, 46), 58.0651 (88)	30	Armepavine (isomer) (Benzylisoquinolines)
P6	4.19	$C_{18}H_{22}NO_5^+$	M+H	332.1484	-2.41	315.1242 (4), 181.0864 (10), 164.0709 (l, **100**), 146.0595 (13), 118.0645 (6)	20	Dimetolquinol (Benzylisoquinolines)
P7	4.29	$C_{23}H_{30}NO_8^+$	M+H	448.1966	0.00	286.1140 (**100**), 269.1170 (93), 237.0914 (17), 209.0959 (11), 178.0865 (18), 175.0749 (13), 145.0640 (13), 143.0489 (19), 137.0583 (21), 107.0489 (k, 95)	40	(Iso)coclaurine O-hexoside (isomer) (Benzylisoquinolines)
P9	4.44	$C_{17}H_{20}NO_3^+$	M+H	286.1436	-0.70	269.1179 (82), 237.0882 (5), 209.0960 (13), 175.0744 (m, 11), 145.0630 (4), 143.0473 (6), 137.0579 (3), 137.0579 (7), 107.0489 (k, 24)	20	Coclaurine (Benzylisoquinolines)
P10	4.56	$C_{23}H_{30}NO_8^+$	M+H	448.1957	-2.01	430.1871 (**100**), 412.1718 (5), 286.1447 (14), 269.1162 (12), 251.0889 (4), 177.0545 (4), 107.0490 (k, 4)	30	(Iso)coclaurine *O*-hexoside (Benzylisoquinolines)
P11	4.58	$C_{12}H_{14}NO_3^+$	M+H	220.0970	0.91	218.0827 (54), 137.0589 (32), 119.0484 (10), 91.0529 (17)	30	Oleracein E
P12	4.66	$C_{20}H_{24}NO_4^+$	M+H	342.1702	0.58	299.1289 (12), 297.1128 (**100**), 282.0888 (20), 265.0869 (75), 237.0904 (11), 58.0654 (38)	30	Magnoflorine (Aporphines Y)
P15	4.72	$C_{21}H_{26}NO_4^+$	M+H	356.1853	-0.84	311.1277 (20), 296.1066 (15), 285.1119 (15), 280.1076 (19), 279.1014 (11), 257.1159 (7), 72.0800 (22), 58.0646 (68)	30	Xanthoplanine (Aporphines Y)
P16	4.88	$C_{19}H_{24}NO_4^+$	M+H	330.1711	3.33	299.1288 (11), 267.1026 (8), 192.1020 (l, **100**), 175.0756 (m, 27), 143.0488 (10), 137.0596 (k, 28)	30	Reticuline (Benzylisoquinolines)
P17	4.96	$C_{20}H_{22}NO_4^+$	M+H	340.1547	1.18	325.1294 (6), 308.1287 (8), 309.1377 (14), 295.0970 (65), 263.0708 (17)	20	Dehydroliriofeine (isomer) (Benzylisoquinolines)
P21	5.00	$C_{20}H_{24}NO_4^+$	M+H	342.1705	1.46	297.1134 (**100**), 282.0895 (2), 265.0867 (78), 237.0907 (2), 58.0651 (3)	25	Magnoflorine (isomer) (Aporphines Y)
P22	5.02	$C_{19}H_{24}NO_3^+$	M+H	314.1756	1.59	269.1168 (10), 209.0951 (15),175.0741 (m, 7), 145.0646 (10), 143.0481 (18), 121.0645 (16), 107.0487 (k, **100**), 58.0658 (30)	40	Armepavine (Benzylisoquinolines)
P24	5.23	$C_{19}H_{22}NO_3^+$	M+H	312.1587	-2.24	294.1510 (**100**), 249.0911 (32), 217.0652 (50), 58.0654 (47)	25	Pulchine (Aporphines Y)
P25	5.25	$C_{20}H_{22}NO_4^+$	M+H	340.1550	2.06	325.1317 (34), 308.1284 (48), 295.0972 (**100**), 269.0900 (9), 263.0708 (80)	25	Dehydroliriofeine (Aporphines Y)
P26	5.35	$C_{17}H_{20}NO_3^+$	M+H	286.1435	-1.05	269.1184 (78), 237.0905 (24), 209.0946 (10), 178.0852 (l, 10), 175.0729 (m, 27), 145.0634 (8), 143.0475 (13), 137.0900 (22), 107.0483 (k, **100**)	25	Coclaurine (isomer) (Benzylisoquinolines)
P27	5.37	$C_{20}H_{20}NO_5^+$	M+H	356.1501	2.53	311.0914 (**100**), 296.0687 (7), 279.0663 (7), 251.0701 (3), 58.0656 (2)	25	Leucoxine (Aporphines Y)
P41	6.59	$C_{20}H_{24}NO_2^+$	M+H	310.1798	-1.29	265.1238 (**100**), 250.0996 (58), 234.1042 (26), 58.0651 (20)	30	Atherospermine
P42	6.67	$C_9H_8NO^+$	M+H	146.0597	-2.05	118.0652 (**100**), 91.0541 (19)	20	Indole-3-carboxaldehyde[b]
P44	6.69	$C_{15}H_{16}NO_5^+$	M+H	290.1015	-2.76	272.0918 (11), 154.0871 (a, 38), 152.0709 (6), 137.0595 (i, 12), 137.0238 (b, 34)	15	*N*-[2-(3,4-dihydroxy-phenyl)ethyl]-3,4-dihydroxybenzamide (Phenolic amides V)

Table S1: *Cont.*

P46	6.85	$C_{13}H_{18}NO_2^+$	M+H	220.1326	-2.73	202.1198 (3), 131.0483 (b, 5), 105.0688 (d, 17), 91.0544 (5), 88.0756 (a, 67), 70.0650 (9)	20	1-(2-Hydroxy-pyrrolidin-1-yl)-3-phenylpropan-1-one (Phenolic amides U)
P50	7.44	$C_{17}H_{18}NO_3^+$	M+H	284.1279	-0.70	147.0453 (b, **100**), 121.0650 (i, 46), 119.0497 (3)	25	Paprazine[b] (Phenolic amides V)
P52	7.50	$C_{15}H_{16}NO_4^+$	M+H	274.1069	-1.82	154.0481 (j, 9), 138.0908 (a, 69), 137.0231 (b, 78), 121.0657 (i, **100**)	20	Houttuynamide A (Phenolic amides V)
P55	7.63	$C_{18}H_{20}NO_4^+$	M+H	314.1382	-1.59	177.0552 (b, **100**), 147.0444 (32), 121.0658 (i, 14)	20	*trans*-N-feruloyltyramine[b] (Phenolic amides V)
P57	8.16	$C_{15}H_{20}NO_3^+$	M+H	262.1434	-1.53	191.0719 (b, **100**), 176.0458 (3), 163.0761 (d, 13), 160.0513 (3), 148.0522 (5), 133.0555 (1), 123.0793 (1), 119.0473 (1), 105.0693 (3), 98.0602 (c, 5), 72.0809 (a, 1), 55.0537 (3)	30	Demethoxy-piperlotine C (Phenolic amides U)
P58	8.34	$C_{38}H_{41}N_2O_{10}^+$	M+H	685.2736	-2.92	548.1947 (b_1,**100**), 520.1975 (d_1, 58), 394.1298 (37), 383.1143 (b_2, 80), 357.1354 (d_2,11), 351.0883 (27), 231.0650 (21)	35	Lignanamides A (Lignanamides)
P59	8.40	$C_{16}H_{18}NO_3^+$	M+H	272.1271	-3.67	255.1043 (10), 151.0758 (l, 26), 105.0339 (k, **100**), 91.0538 (3)	20	Norcoclaurine (Benzylisoquinolines)
P60	8.46	$C_{37}H_{39}N_2O_9^+$	M+H	655.2631	-2.90	518.1823 (b_1, **100**), 490.1889 (d_1, 64), 394.1306 (11), 353.1031 (b_2, 22), 327.1207 (d_2, 11), 321.0761 (10), 231.0645 (14)	30	Methoxy-cannabisin D (Lignanamides)
P61	8.52	$C_{16}H_{22}NO_4^+$	M+H	292.1538	-1.71	221.0925 (b, **100**), 206.0588 (44), 193.0968 (d, 1), 191.0340 (7), 181.0961 (1), 178.0633 (3), 167.0694 (1), 163.0390 (1), 150.0678 (0.5), 135.0429 (0.5), 107.0490 (0.5), 98.0602 (c, 8), 70.0647 (a, 0.5)	30	Piperlotine C (Phenolic amides U)
P62	8.56	$C_{15}H_{20}NO_3^+$	M+H	262.1431	-2.67	191.0707 (b, **100**), 176.0461 (6), 163.0752 (d, 45), 160.0511 (7), 148.0519 (14), 132.0563 (5), 120.0565 (3), 119.0477 (5), 98.0594 (c, 5), 72.0810 (a, 1), 55.0540 (3)	35	Demethoxypiper-lotine C (isomer-A) (Phenolic amides U)
P63	8.73	$C_{15}H_{10}NO_3^+$	M+H	252.0662	2.78	234.0534 (7), 224.0693 (14), 197.0579 (7), 196.0763 (13), 178.0670 (7)	30	Hippacine
P64	8.75	$C_{16}H_{16}NO_4^+$	M+H	286.1063	-3.84	268.0948 (**100**), 254.0903 (19), 238.0844 (44), 237.0766 (5), 198.0529 (5)	20	Ganoapplanatumine B
P65	8.91	$C_{16}H_{22}NO_4^+$	M+H	292.1534	-3.08	221.0831 (b, **100**), 206.0591 (25), 193.0874 (d, 7), 191.0352 (3), 190.0638 (7), 189.0562 (1), 178.0634 (3), 175.0392 (1), 162.0689 (2), 161.0611 (2), 149.0599 (1), 135.0453 (1), 133.0660 (1), 98.0604 (c, 4), 72.0805 (2), 70.0654 (a, 1), 55.0548 (2)	30	Piperlotine C (isomer) (Phenolic amides U)
P66	8.97	$C_{18}H_{20}NO_3^+$	M+H	298.1429	-3.02	177.0555 (b, **100**), 149.0605 (4), 145.0299 (41), 117.0333 (10), 105.0697 (i, 19)	25	Feruloylphenethyl-amine (Phenolic amides V)
P68	9.54	$C_{37}H_{39}N_2O_9^+$	M+H	655.2634	-2.44	518.1819 (b_1, 23), 492.2034 (d_1, 22), 381.0976 (14), 355.1181 (b_2, 23)	25	Methoxycannabisin D (isomer) (Lignanamides)
P69	9.58	$C_{14}H_{18}NO_2^+$	M+H	232.1323	-3.88	161.0602 (b, **100**), 133.0647 (d, 26), 118.0413 (12), 98.0591 (c, 3), 90.0454 (3), 72.0801 (a, 5), 55.0548 (3)	30	Piperlotine A (Phenolic amides U)
P70	9.60	$C_{16}H_{12}NO_3^+$	M+H	266.0813	0.38	251.0580 (**100**), 223.0612 (9), 195.0679 (37), 168.0556 (7), 167.0728 (37)	40	Piperolactam A[b] (Aporphines Z)
P71	9.76	$C_{18}H_{14}NO_4^+$	M+H	308.0905	-3.89	293.0714 (77), 265.0774 (**100**), 237.0797 (4), 209.0855 (74), 208.0770 (4), 181.0893 (7)	40	Norcepharadione B[b] (Aporphines Y)
P72	9.85	$C_{14}H_{16}NO_2^+$	M+H	230.1170	-2.61	161.0597 (b, **100**), 133.0639 (d, 36), 118.0397 (5), 105.0684 (5), 96.0432 (c, 18), 78.0332 (7), 68.0501 (a, 9)	30	Dehydropiperlotine A (Phenolic amides U)
P73	9.91	$C_{15}H_{20}NO_3^+$	M+H	262.1436	-0.76	191.0714 (b, **100**), 176.0464 (5), 163.0758 (d, 31), 148.0519 (41), 120.0564 (1), 119.0488 (4), 98.0597 (c, 5), 91.0543 (5), 72.0805 (a, 5), 55.0543 (4)	35	Demethoxypiperlotine C (isomer-B) (Phenolic amides U)
P74	9.91	$C_{34}H_{29}N_2O_8^+$	M+H	593.1903	-2.53	456.1101 (b_1, **100**), 430.1305 (18), 428.1130 (d_1, 15), 344.1492 (13), 330.1345 (6), 250.0510 (6), 236.0711 (13), 207.0654 (6), 121.0642 (25)	30	Lignanamides B (Lignanamides)

Table S1: *Cont.*

P75	10.11	$C_{17}H_{14}NO_4^+$	M+H	296.0922	1.69	281.0697 (44), 280.0514 (4), 266.0462 (6), 264.0670 (12), 263.0592 (74), 236.0707 (4), 235.0642 (**100**)	30	Piperolactam B (Aporphines Z)
P76	10.15	$C_{18}H_{14}NO_4^+$	M+H	308.0914	-0.97	292.0623 (80), 290.0827 (**100**), 264.0672 (96), 247.0642 (74)	35	Piperadione/ Aristolodione (Aporphines Y)
P77	10.27	$C_{18}H_{14}NO_5^+$	M+H	324.0864	-0.62	309.0645 (52), 308.0563 (84), 291.0534 (63), 263.0573 (15)	30	Cepharanone D (Aporphines Z)
P78	10.33	$C_{16}H_{20}NO_3^+$	M+H	274.1439	0.36	161.0601 (g, 5), 139.0996 (f, 14), 137.0577 (5), 135.0438 (e, **100**), 124.0745 (24), 123.0817 (5), 117.0684 (8), 113.0588 (8), 99.0444 (5), 98.0594 (c, 5), 95.0854 (8), 72.0902 (a, 14)	30	Piperamide-C5:1 (Piperamides E)
P79	10.35	$C_{14}H_{16}NO_3^+$	M+H	246.1120	-2.03	214.0875 (**100**), 196.0761 (33), 186.0917 (29), 133.0648 (30), 114.0550 (c, 62), 105.0697 (9), 82.0286 (a, 30)	15	Piperlotine G (Phenolic amides U)
P80	10.44	$C_{16}H_{18}NO_3^+$	M+H	272.1277	-1.47	201.0568 (b, **100**), 173.0599 (d, 6), 171.0446 (11), 159.0438 (g, 3), 143.0491 (11), 135.0445 (e, 38), 115.0543 (11), 98.05893 (c, 6), 72.0905 (a, 1), 55.0541 (2)	30	Piperyline (Piperamides E)
P81	10.60	$C_{18}H_{12}NO_4^+$	M+H	306.0750	-3.59	278.0815 (**100**), 263.0567 (24), 248.0701 (43), 220.0750 (49)	40	Cepharadione A[b] (Aporphines Y)
P82	10.64	$C_{16}H_{18}NO_3^+$	M+H	272.1279	-0.73	201.0561 (b, **100**), 173.0603 (d, 7), 171.0446 (12), 159.0443 (g, 3), 143.0493 (15), 135.0445 (e, 41), 115.0544 (16), 98.0600 (c, 5), 70.0653 (a, 1), 55.0544 (2)	30	Piperyline (isomer-A) (Piperamides E)
P83	10.64	$C_{18}H_{14}NO_5^+$	M+H	324.0857	-2.78	309.0670 (39), 308.0573 (**100**), 292.0615 (19), 291.0548 (53), 290.0472 (35), 263.0595 (51), 235.0623 (19)	35	Cepharanone D (isomer) (Aporphines Z)
P84	10.88	$C_{16}H_{18}NO_3^+$	M+H	272.1276	-1.84	201.0550 (b, **100**), 173.0592 (d, 6), 171.0439 (15), 159.0435 (g, 5), 143.0487 (11), 137.0835 (f, 3), 135.0439 (e, 35), 115.0538 (15), 98.0598 (c, 5), 55.0540 (2)	30	Piperyline (isomer-B) (Piperamides E)
P87	11.09	$C_{17}H_{14}NO_3^+$	M+H	280.0959	-3.21	265.0739 (22), 264.0647 (39), 149.0247 (24)	25	Aristolactam BII[b] (Aporphines Z)
P88	11.15	$C_{19}H_{20}NO_3^+$	M+H	310.1433	-1.61	278.1193 (44), 265.1238 (24), 250.1244 (44), 233.0977 (**100**)	15	*N*-Formylnornuciferin (Aporphines Y)
P91	11.61	$C_{17}H_{20}NO_3^+$	M+H	286.1430	-2.80	201.0553 (b, **100**), 173.0609 (d, 6), 171.0443 (24), 159.0441 (g, 7), 151.0934 (4), 143.0486 (31), 135.0438 (e, 32), 117.0695 (1), 115.0539 (32), 112.0745 (c, 6), 86.0954 (a, 1), 84.0938 (3), 69.0696 (4)	35	Piperine (Piperamides A)
P92	11.66	$C_{18}H_{20}NO_3^+$	M+H	298.1430	-2.68	227.0715 (b, **100**), 199.0759 (d, 20), 197.0600 (19), 169.0657 (47), 161.0604 (g, 7), 159.0439 (3), 150.0910 (5), 141.0702 (19), 135.0429 (e, 1), 131.0490 (7), 98.0602 (c, 36), 72.0810 (a, 3), 70.0645 (1)	25	Piperettyline (Piperamides E)
P93	11.66	$C_{18}H_{22}NO_3^+$	M+H	300.1590	-1.33	229.0956 (b, 4), 201.0904 (d, 10), 187.0747 (5), 171.0901 (1), 161.0596 (g, 73), 139.0982 (h, 7), 135.0433 (e, 8), 131.0488 (**100**), 124.0750 (7), 103.0537 (18), 98.0595 (c, 23), 84.0912 (1), 72.0806 (a, 8)	25	Nigrinodine (Piperamides E)
P97	11.90	$C_{18}H_{16}NO_4^+$	M+H	310.1064	-3.22	295.0855 (44), 294.0742 (7), 280.0618 (67), 279.0901 (13), 277.0753 (71), 276.0658 (13), 262.,513 (16), 252.0649 (10), 250.0873 (18), 249.0800 (26), 221.0836 (9)	30	Aristolactam BIII (Aporphines Z)
P98	11.92	$C_{18}H_{20}NO_3^+$	M+H	298.1437	-0.34	227.0728 (b, **100**), 199.0767 (d, 25), 197.0612 (25), 171.0934 (1), 169.0661 (53), 161.0633 (g, 8), 159.0444 (3), 150.0913 (8), 141.0705 (23), 135.0444 (e, 1), 131.0493 (10), 124.0760 (3), 115.0537 (3), 103.0542 (3), 98.0608 (c, 56), 72.0903 (a, 3), 70.0854 (1), 55.0547 (5)	25	Piperettyline (isomer-A) (Piperamides E)
P99	11.98	$C_{20}H_{22}NO_4^+$	M+H	340.1540	-0.88	308.1302 (**100**), 295.1349 (81), 293.1057 (30), 280.1355 (16), 263.1076 (26), 249.1150 (18)	20	Dehydronorglaucine (Aporphines Y)

Table S1: *Cont.*

P100	12.00	$C_{19}H_{18}NO_3^+$	M+H	308.1274	-2.27	293.1072 (42), 280.1316 (25), 277.1123 (19), 249.1163 (31)	20	*N*-Demethyl-*N*-formyldehydronuciferine (Aporphines Y)
P101	12.08	$C_{18}H_{24}NO_3^+$	M+H	302.1744	-2.32	272.1650 (1), 231.1029 (b, 10), 213.0917 (d, 13), 201.0915 (4), 187.0756 (4), 180.1393 (17), 154.1227 (5), 140.1068 (1), 135.0453 (e, **100**), 98.0601 (c, 16), 84.0808 (1), 72.0814 (a, 39), 70.0654 (3), 55.0538 (1)	25	Piperamide-C7:1 (Piperamides E)
P102	12.21	$C_{18}H_{20}NO_3^+$	M+H	298.1440	0.67	227.0714 (b, **100**), 199.0764 (d, 25), 197.0600 (28), 169.0651 (45), 161.0597 (g, 9), 150.0913 (6), 141.0899 (23), 139.0937 (1), 131.0493 (10), 124.0763 (1), 115.0538 (3), 103.0543 (3), 98.0600 (c, 41), 72.0808 (a, 4), 70.0643 (1), 55.0543 (3)	25	Piperettyline (isomer-B) (Piperamides E)
P103	12.27	$C_{19}H_{18}NO_3^+$	M+H	308.1274	-2.27	293.1077 (10), 280.1362 (22), 277.1117 (11), 249.1198 (5)	20	*N*-Demethyl-*N*-formyldehydronuciferine (isomer) (Aporphines Y)
P104	12.29	$C_{18}H_{26}NO_2^+$	M+H	288.1951	-2.43	194.1546 (1), 168.1384 (4), 151.1120 (66), 133.1010 (12), 121.0845 (22), 109.0641 (3), 105.691 (2), 95.0493 (13), 93.0694 (4), 91.0541 (2), 81.0326 (3), 69.0692 (3), 67.0544 (1)	20	2,4-Decadienoic acid *p*-hydroxyphenethyl-amide (Phenolic amide)
P105	12.31	$C_{18}H_{24}NO_3^+$	M+H	302.1745	-1.99	229.0869 (b, 6), 203.1082 (d, 17), 201.0920 (22), 187.0754 (3), 180.1388 (5), 173.0965 (5), 161.0510 (g, **100**), 135.0442 (e, 19), 131.0498 (53), 107.0493 (1), 103.0541 (8), 100.0759 (c, 1), 81.0701 (13), 79.0542 (1), 74.0966 (a, 4), 57.0702 (4)	20	Chingchengenamide A (Piperamides B)
P106	12.41	$C_{14}H_{22}NO^+$	M+H	220.1694	-0.91	149.0949 (b, 5), 137.0828 (24), 136.0745 (5), 121.1009 (d, 5), 107.0490 (**100**), 98.0593 (c, 37), 93.0587 (5), 91.0634 (10), 79.0531 (15), 77.0376 (9), 72.0801 (a, 5), 70.0651 (10), 55.0544 (47)	30	1-(Pyrrolidinyl)-10-2,4,6- decatrienamide (Pyyrolamides F)
P107	12.53	$C_{18}H_{22}NO_3^+$	M+H	300.1594	0.00	227.0705 (b, **100**), 201.0892 (d, 39), 199.0751 (43), 197.0598 (53), 171.0789 (13), 169.0644 (60), 161.0583 (g, 18), 141.0693 (27), 131.0487 (40), 103.0535 (13), 79.0526 (6), 57.0694 (i, 19)	25	3,4-Dehydrofutoamide (Piperamides B)
P108	12.75	$C_{19}H_{24}NO_3^+$	M+H	314.1747	-1.27	229.0834 (b, 1), 201.0925 (d, 4), 192.1385 (1), 187.0757 (1), 171.0310 (1), 166.1243 (1), 161.0609 (g, 39), 153.1152 (h, 3), 140.1070 (1), 138.0920 (9), 131.0502 (**100**), 112.0765 (c, 11), 103.0548 (15), 86.0971 (a, 5), 84.0806 (1), 69.0703 (3)	30	Piperdardine (Piperamides A)
P109	12.80	$C_{19}H_{22}NO_3^+$	M+H	312.1603	2.88	227.0716 (b, **100**), 199.0758 (d, 15), 197.0631 (15), 169.0660 (50), 161.0612 (g, 7), 141.0695 (15), 131.0487 (7), 112.0750 (c, 31), 86.0970 (a, 6), 69.0704 (8)	25	Piperettine (Piperamides A)
P110	12.98	$C_{20}H_{24}NO_3^+$	M+H	326.1752	0.31	255.1031 (b, 4), 227.1069 (d, 5), 187.0754 (9), 165.1152 (h, **100**), 161.0594 (g, 32), 150.0905 (5), 135.0439 (e, 4), 131.0488 (29), 103.0538 (4), 98.0599 (c, 58), 72.0804 (a, 3), 70.0643 (1)	25	6,7-Dehydro-brachyamide B (Piperamides E)
P111	13.06	$C_{19}H_{22}NO_3^+$	M+H	312.1582	-3.84	227.0720 (b, **100**), 199.0759 (d, 22), 197.0603 (30), 169.0650 (42), 161.0582 (g, 10), 141.0692 (14), 138.0894 (4), 131.0489 (8), 115.0530 (6), 112.0753 (c, 58), 86.0963 (a, 4), 69.0701 (14)	25	Piperettine (isomer) (Piperamides A)
P112	13.14	$C_{14}H_{24}NO^+$	M+H	222.1852	0.00	194.1906 (2), 168.1744 (1), 150.0918 (h, 15), 124.0761 (27), 110.0963 (15), 98.0604 (c, 71), 95.0492 (15), 91.0539 (3), 81.0342 (**100**), 72.0810 (a, 13), 70.0654 (14), 69.0698 (10), 67.0545 (29), 53.0390 (92), 55.0540 (38)	40	Sarmentine (Pyrrolamides F)
P113	13.32	$C_{20}H_{20}NO_4^+$	M+H	338.1384	-0.89	323.1179 (21), 310.1471 (18), 307.1236 (**100**), 279.1285 (46)	30	Dehydro-formouregine[b] (Aporphines Y)
P114	13.39	$C_{20}H_{26}NO_3^+$	M+H	328.1898	-2.74	229.1236 (d, 22), 199.1124 (7), 161.0600 (g, 11), 135.0449 (e, **100**), 131.0494 (7), 98.0602 (c, 22), 84.0804 (5), 72.0812 (a, 12)	30	Brachyamide B (Piperamides E)
P116	13.59	$C_{14}H_{26}NO^+$	M+H	224.2006	-1.34	168.1390 (j, 39), 151.1121 (b, 13), 123.1168 (d, 18), 112.0760 (13), 109.1010 (33), 98.0901 (46),	30	Pellitorine (Piperamides C)

Table S1: *Cont.*

						95.0496 (42), 93.0696 (12), 83.0855 (21), 81.0344 (61), 79.0538 (16), 74.0966 (a, 1), 69.0701 (54), 67.0544 (43), 57.0704 (i, **100**)		
P117	13.83	$C_{20}H_{28}NO_3^+$	M+H	330.2056	-2.42	229.1217 (d, 1), 208.1695 (2), 161.0590 (g, 1), 135.0440 (e, **100**), 131.0479 (1), 98.0597 (c, 9), 84.0604 (1), 72.0807 (a, 11), 70.0650 (1)	30	Tricholein (Piperamides E)
P118	14.02	$C_{22}H_{26}NO_3^+$	M+H	352.1896	-3.12	253.1194 (d, 1), 239.1052 (1), 223.1125 (1), 213.0901 (1), 187.0754 (**100**), 165.1149 (34), 157.0649 (44), 150.0913 (4), 135.0423 (e, 3), 129.0696 (22), 128.0618 (5), 98.0595 (c, 31), 70.0647 (a, 1), 55.0541 (2)	30	1-(Pyrrolidinyl-11-(3',4'-methylenedioxy-phenyl)-2,4,8,10-undecatetraen-1-one (Piperamides E)
P119	14.14	$C_{18}H_{14}NO_4^+$	M+H	308.0906	-0.32	293.0707 (**100**), 292.0618 (21), 276.0666 (9), 275.0581 (14), 265.0754 (12), 262.0868 (9), 247.0646 (9)	30	Piperadione (Aporphines)
P120	14.24	$C_{21}H_{28}NO_3^+$	M+H	342.2052	-3.51	255.1015 (b, 2), 229.1224 (d, 2), 227.1053 (5), 197.0943 (1), 187.0773 (4), 181.1454 (h, 21), 166.1228 (4), 161.0598 (g, 44), 135.0426 (6), 131.0487 (**100**), 103.0533 (e, 16), 86.0951 (a, 1), 79.0528 (1), 71.0843 (5)	30	Pipernonaline (isomer) (Piperamides A)
P121	14.42	$C_{22}H_{28}NO_3^+$	M+H	354.2060	-1.13	255.1388 (d, 7), 241.1222 (6), 187.0757 (**100**), 161.0594 (g, 6), 157.0692 (49), 135.0438 (e, 16), 129.0699 (30), 128.0801 (6), 98.0569 (c, 15), 72.0815 (a, 7)	30	1-(Pyrrolidinyl-11-(3',4'-methylenedioxy-phenyl)-2,4,10-undecatrien-1-one (Piperamides E)
P123	14.48	$C_{21}H_{28}NO_3^+$	M+H	342.2054	-2.92	257.1174 (b, 3), 229.1223 (d, 37), 199.1114 (12), 187.0744 (6), 171.0790 (5), 161.0594 (g, 18), 135.0441 (e, **100**), 131.0480 (12), 112.0754 (c, 31), 98.0955 (8), 86.0983 (a, 52), 84.0803 (8), 69.0702 (14)	30	Pipernonaline/ Pipernonatine (Piperamides A)
P124	14.50	$C_{15}H_{28}NO^+$	M+H	238.2162	-1.26	168.1390 (j, **100**), 151.1126 (b, 32), 123.1172 (d, 36), 112.0754 (23), 109.1014 (48), 100.1121 (23), 98.0903 (47), 95.0494 (56), 83.0956 (21), 81.0638 (43), 71.0859 (43), 69.0706 (i, 81), 67.0550 (45), 55.0550 (45)	30	Homopellitorine / N-2'-methylbutyl-decadienamide (Piperamide)
P125	14.61	$C_{22}H_{28}NO_3^+$	M+H	354.2063	-0.28	255.1390 (d, 4), 215.1071 (3), 187.0760 (3), 173.0601 (2), 161.0600 (g, 3), 135.0447 (e, **100**), 131.0490 (6), 98.0602 (c, 39), 91.0540 (3), 84.0805 (4), 72.0811 (a, 12), 55.0543 (3)	30	1-(Pyrrolidinyl)-11-(3',4'-methylenedioxy-phenyl)-undecatrien-1-one (isomer) (Piperamides E)
P128	14.93	$C_{22}H_{30}NO_3^+$	M+H	356.2220	0.00	283.1337 (b, 1), 255.1399 (d, 2), 215.1071 (1), 187.0769 (1), 175.0758 (2), 161.0605 (g, 4), 135.0450 (e, **100**), 131.0498 (7), 107.0857 (2), 103.0545 (2), 93.0697 (2), 91.0543 (3), 86.0969 (3), 79.0538 (2), 74.0963 (a, 1), 67.0548 (1), 57.0702(6)	30	Retrofractamide B (Piperamides B)
P129	15.13	$C_{22}H_{30}NO_3^+$	M+H	356.2211	-2.53	285.1486 (b, 3), 255.1393 (d, 3), 234.1884 (5), 187.0760 (5), 161.0605 (g, 6), 135.0444 (e, **100**), 131.0495 (7), 98.0594 (c, 11), 84.0808 (13), 74.0956 (a, 1), 72.0810 (10)	30	11-(3,4-methylenedi-oxyphenyl)-2,10-un-decenoyl]pyrrolidine (Piperamides E)
P131	15.22	$C_{18}H_{28}NO^+$	M+H	274.2159	-2.19	165.1153 (**100**), 164.1070 (15), 150.0913 (h, 15), 136.1123 (4), 109.1004 (3), 98.0603 (c, 95), 95.0484 (3), 72.0817 (a, 3), 70.0549 (3), 67.0542 (2), 55.0542 (i, 3)	25	1-(Pyrrolidinyl-14-2,4,7,9-tetradeca-tetraen-1-one (Pyrrolamides F)
P132	15.34	$C_{16}H_{28}NO^+$	M+H	250.2164	-0.40	232.2049 (3), 196.2059 (9), 182.1901 (3), 179.1417 (b, 4), 152.1079 (9), 150.0920 (h, 14), 138.0917 (8), 124.1117 (8), 109.1009 (8), 98.0602 (c, 97), 97.1006 (17), 95.0493 (27), 83.0488 (8), 81.0691 (18), 72.0810 (a, 26), 70.0652 (18), 55.0543 (i, 63), 53.0386 (58)	35	2,4-Dodecadienoyl pyrrolidine (Pyrrolamides F)
P133	15.58	$C_{16}H_{30}NO^+$	M+H	252.2320	-0.79	198.1711 (j, 53), 179.1434 (b, 10), 154.1229 (d, 13), 126.0915 (13), 109.1012 (14), 98.0601 (c, 29), 97.1016 (24), 95.0853 (49), 93.0705 (8), 84.0403 (8), 83.0959 (18), 81.0339 (41), 79.0539 (14), 74.0954 (c, 3), 69.0702 (29), 67.0545 (18), 57.0545 (i, 18), 55.0548 (37)	30	Kalecide (Piperamides C)

Table S1: *Cont.*

P134	15.80	$C_{24}H_{32}NO_3^+$	M+H	382.2362	-3.92	309.1495 (b, 7), 281.1557 (d, 14), 260.1999 (24), 241.1218 (17), 227.1081 (7), 215.1090 (8), 201.0916 (6), 187.0750 (10), 161.0800 (g, 43), 135.0438 (e, **100**), 131.0508 (22), 98.0591 (c, 17), 74.0961 (a, 7), 72.0804 (17), 57.0684 (7)	25	Dehydroguineesine (Piperamides B)
P135	16.29	$C_{24}H_{32}NO_3^+$	M+H	382.2363	-3.66	311.1674 (b, 1), 283.1719 (d, 1), 213.0928 (1), 201.0920 (1), 187.0766 (4), 175.0760 (2), 161.0808 (g, 9), 135.0451 (e, **100**), 131.0500 (15), 103.0545 (3), 98.0606 (c, 26), 84.0812 (22), 81.0334 (2), 72.0812 (a, 5), 55.0545 (3)	35	Brachyamide A (Piperamides E)
P136	16.40	$C_{24}H_{34}NO_3^+$	M+H	384.2525	-2.08	311.1664 (b, 4), 283.1715 (d, 8), 187.0762 (4), 175.0758 (4), 161.0602 (g, 9), 135.0446 (e, **100**), 131.0491 (5), 123.0439 (4), 109.1010, 95.0851 (2), 86.0965 (18), 81.0994 (2), 74.0961 (a, 1), 57.0999 (3)	30	Guineensine (Piperamides B)
P137	16.83	$C_{24}H_{36}NO_3^+$	M+H	386.2677	-3.37	313.1826 (b, 36), 285.1875 (d, 1), 264.2325 (6), 175.0766 (7), 161.0600 (g, 17), 135.0446 (e, **100**), 131.0497 (4), 109.1011 (3), 95.0855 (4), 86.0967 (19), 81.0703 (3), 74.0968 (a, 4), 57.0999 (4)	30	Piperflaviflorine A (Piperamides B)
P138	16.84	$C_{24}H_{34}NO_3^+$	M+H	384.2524	-2.34	313.1819 (b, 6), 262.2164 (4), 175.0755 (4), 161.0603 (g, 9), 135.0441 (e, **100**), 131.0437 (7), 98.0588 (c, 15), 84.0806 (15), 72.0803 (a, 7)	35	Dihydrobrachyamide A (Piperamides E)
P139	17.01	$C_{25}H_{36}NO_3^+$	M+H	398.2680	-2.51	311.1662 (b, 6), 283.1703 (d, 15), 187.0749 (2), 161.0593 (g, 3), 135.0442 (e, 24), 131.0437 (1), 107.0491 (1), 100.1118 (10), 95.08046 (1), 88.1112 (1), 81.0688 (1)	25	Piperflaviflorine B (Piperamides)
P140	17.25	$C_{22}H_{34}NO^+$	M+H	328.2629	-1.83	178.1221 (7), 164.1064 (h, 12), 126.0906 (20), 119.0844 (11), 107.0847 (11), 105.0689 (11), 98.0597 (**100**), 91.0540 (17), 81.0693 (16), 79.0539 (12), 72.0800 (17), 67.0534 (i, 11), 55.0541 (20)	30	1-(Pyrrolidinyl)-18-2,4,9,11,13-octadecatetraen-1-one (Pyrrolamides F)
P141	17.43	$C_{18}H_{32}NO^+$	M+H	278.2471	-2.52	250.2538 (17), 224.2375 (11), 164.1062 (6), 152.1066 (h, 11), 136.0749 (13), 124.0753 (64), 110.0954 (26), 98.0595 (c, **100**), 95.0489 (25), 81.0696 (25), 79.0533 (29), 72.0809 (a, 47), 70.0645 (17), 69.0699 (25), 67.0539 (29), 55.0543 (i, 64), 53.0388 (60)	40	Achilleaamide (Pyrrolamides F)
P142	17.78	$C_{22}H_{36}NO^+$	M+H	330.2782	-2.72	259.2068 (b, 6), 236.2014 (9), 222.1353 (13), 208.1706 (8), 152.1077 (h, 16), 139.0996 (12), 124.0758 (14), 121.1013 (18), 119.0859 (14), 107.0862 (26), 98.0604 (c, **100**), 95.0859 (32), 93.0703 (43), 91.0541 (14), 81.0705 (34), 79.0545 (13), 72.0816 (a, 56), 67.0546 (42), 55.0541 (i, 16)	30	1-(Pyrrolidinyl)-18-2,4,11,13-octadecatetraen-1-one (Pyrrolamides F)
P143	17.79	$C_{26}H_{38}NO_3^+$	M+H	412.2834	-2.91	339.1982 (b, 53), 311.2033 (d, 13), 290.2498 (9), 161.0605 (g, 17), 135.0455 (e, **100**), 86.0972 (30), 81.0696 (3), 57.0704 (2)	30	Brachystamide B (Piperamides B)
P144	17.86	$C_{26}H_{36}NO_3^+$	M+H	410.2674	-3.90	339.1998 (b, 5), 288.2342 (4), 187.0780 (3), 161.0603 (g, 13), 135.0453 (e, **100**), 98.0606 (c, 25), 84.0811 (18), 81.0340 (5), 72.0815 (a, 6), 55.0547 (3)	40	1-(Pyrrolidinyl)-15-(3',4'-methylenedioxy-phenyl)-2,4,14-pentadecatrien-1-one (Piperamides E)
P145	18.25	$C_{22}H_{38}NO^+$	M+H	332.2935	-3.91	276.2377 (j, 7), 233.2235 (d, 7), 208.1695 (21), 185.1174 (29), 166.1219 (21), 147.0799 (30), 135.1171 (37), 133.1023 (26), 128.1058 (26), 121.1015 (90), 109.1009 (49), 107.0954 (60), 95.0951 (84), 93.0699 (**100**), 91.0536 (36), 83.0850 (20), 81.0332 (8), 79.0542 (47), 69.0700 (27), 67.0540 (27), 57.0698 (i, 56)	30	*N*-Isobutyl- 2,4,10,12-octadecatetraenmide (Piperamides C)
P146	18.39	$C_{26}H_{38}NO_3^+$	M+H	412.2830	-3.88	341.2134 (b, 5), 313.2174 (d, 1), 290.2510 (8), 161.0603 (g, 12), 135.0452 (e, **100**), 131.0491 (6), 98.0609 (c, 11), 84.0813 (18), 72.0812 (a, 17)	40	1-(Pyrrolidinyl)-15-(3',4'-methylenedioxy-phenyl)-12,14-pentadecadien-1-one (Piperamides D)

Table S1: *Cont.*

P147	18.47	$C_{22}H_{38}NO^+$	M+H	332.2940	-2.41	278.2501 (j, 3), 259.2063 (b, 4), 250.2184 (5), 231.2102 (d, 4), 222.1853 (5), 166.1231 (15), 135.1172 (19), 133.1011 (11), 128.1069 (5), 121.1012 (32), 109.1012 (23), 107.0953 (26), 95.0857 (38), 93.0701 (30), 91.0540 (16), 83.0855 (6), 81.0702 (35), 79.0543 (15), 74.0965 (a, 5), 69.0706 (11), 67.0545 (31), 57.0702 (i, 32)	30	*N*-Isobutyl- 2,4,10,12-octadecatetraenmide (isomer) (Piperamides C)
P148	18.63	$C_{22}H_{36}NO^+$	M+H	330.2786	-1.51	234.1869 (2)., 178.1232 (9), 164.1072 (9), 150.0919 (h, 8), 137.0826 (8), 124.0753 (20), 121.1021 (7), 119.0855 (9), 107.0850 (14), 98.0601 (c, **100**), 95.0853 (30), 93.0698 (23), 91.0539 (18), 81.0895 (21), 79.0544 (23), 72.0809 (a, 22), 67.0544 (56), 55.0543 (i, 53)	40	1-(Pyrrolidinyl)-2,4,10,12-octadecatetraenmide (Pyrrolamides F)
P149	18.88	$C_{22}H_{38}NO^+$	M+H	332.2941	-2.11	166.1228 (h, 4), 152.1079 (23), 139.0992 (13), 126.0916 (17), 124.0762 (72), 98.0604 (**100**), 95.0855 (25), 93.0698 (17), 91.0536 (6), 81.0699 (32), 79.0543 (13), 72.0813 (38), 67.0545 (i, 19), 55.0548 (45)	40	1-(Pyrrolidinyl)-18-2,4,11-octadecatetraen-1-one (Pyrrolamides F)
P150	19.04	$C_{22}H_{42}NO^+$	M+H	336.3251	-2.97	226.2172 (18), 198.1843 (32), 170.1539 (25), 156.1369 (20), 142.1211 (28), 123.0798 (42), 109.1009 (24), 97.0644 (49), 95.0844 (30), 81.0688 (20), 74.0961 (a, 68), 69.0696 (42), 57.0691 (i, **100**), 55.0535 (21)	30	Pipericine (Piperamides C)
P151	19.22	$C_{22}H_{38}NO^+$	M+H	332.2937	-3.31	250.2169 (3), 236.2025 (4), 222.1859 (4), 166.1219 (h, 4), 152.1073 (36), 139.0982 (11), 126.0913 (9), 124.0761 (60), 98.0602 (**100**), 95.0856 (28), 93.0690 (16), 91.0538 (5), 81.0697 (28), 79.0541 (15), 72.0811 (40), 67.0543 (i, 38), 55.0545 (39)	40	1-(Pyrrolidinyl)-18-2,4,11-octadecatetraen-1-one (Pyrrolamides F)
P152	19.26	$C_{20}H_{36}NO^+$	M+H	306.2786	-1.63	278.2849 (5), 252.2592 (4), 235.2056 (b, 1), 180.1382 (1), 152.1071 (h, 5), 150.0909 (2), 138.0903 (3), 124.0756 (8), 109.1006 (4), 98.0599 (c, 42), 95.343 (7), 83.0436 (4), 81.0335 (20), 72.0909 (a, 13), 70.0650 (5), 67.0544 (5), 55.0545 (i, 12), 53.0395 (6)	35	1-(Pyrrolidinyl)-2,4-hexadecadienmide (Pyrrolamides F)
P153	19.39	$C_{22}H_{40}NO^+$	M+H	334.3093	-3.29	261.2221 (b, 1), 233.2268 (2), 180.1372 (1), 137.1329 (3), 135.1164 (4), 128.1072 (4), 123.1173 (5), 109.1009 (7), 107.0852 (4), 97.1011 (4), 95.0351 (8), 83.0953 (3), 81.0700 (5), 74.0964 (a, 1), 67.0536 (1), 57.0703 (i, 8)	25	*N*-Isobutyl-2,4,12-octadecatrienamide (Piperamides C)
P154	19.67	$C_{22}H_{38}NO^+$	M+H	332.2944	-1.20	304.3026 (2), 278.2837 (3), 152.1073 (h, 11), 150. 0922 (23), 138.0922 (7), 126.0911 (9), 124.0763 (60), 98.0601 (c, **100**), 95.0856 (30), 93.0699 (18), 91.0538 (17), 81.0694 (26), 79.0542 (34), 72.0808 (a, 26), 70.0657 (23), 55.0545 (i, 94)	50	1-(Pyrrolidyl)-2,4,12-octadecatrienamide (Pyrrolamides F)
**Flavonoids**								
P29	5.41	$C_{26}H_{29}O_{14}^+$	M+H	565.1539	-2.30	547.15 (85), 529.1394 (62), 511.1286 (86), 499.1243 (68), 457.1161 (34), 445.1148 (39), 427.1081 (72), 409.0944 (37), 295.0675 (14)	25	Isoschaftoside
P30	5.69	$C_{27}H_{31}O_{15}^+$	M+H	595.1657	0.00	475.1249 (3), 433.1178 (89), 415.1050 (60), 397.0942 (37), 367.0829 (11), 337.0727 (20), 313.0748 (**100**), 283.0616 (12), 271.0617 (28)	40	Vitexin 2''-*O*-galactoside[b]
P31	5.82	$C_{27}H_{31}O_{14}^+$	M+H	579.1709	0.17	433.1141 (**100**), 415.1033 (41), 397.0905 (17), 379.0803 (3), 367.0804 (6), 337.0731 (5), 313.0714 (23), 295.0602 (3), 283.0605 (5), 271.0620 (3)	35	Vitexin-2''-*O*-rhamnoside
P32	5.84	$C_{26}H_{29}O_{14}^+$	M+H	565.1536	-2.83	433.1161 (**100**), 415.1050 (38), 397.0932 (30), 367.0901 (14), 337.0750 (19), 313.0734 (44)	35	Vitexin 2''-*O*-pentoside
P34	5.94	$C_{21}H_{21}O_{10}^+$	M+H	433.1120	-2.08	415.1032 (79), 397.0931 (62), 379.0816 (19), 367.0822 (22), 337.0719 (19), 313.0716 (**100**), 295.0615 (6), 283.0611 (17)	30	Vitexin
P36	6.18	$C_{33}H_{39}O_{19}^+$	M+H	739.2074	-0.81	577.1591 (51), 559.1480 (29), 475.1263 (57), 313.0730 (34), 271.0623 (9)	30	Vitexin 4''-(3-hydroxy-3-methylglutaroyl)-2''-*O*-β-D-gluco-pyranoside[b]

Table S1: *Cont.*

P37	6.29	$C_{26}H_{29}O_{14}^+$	M+H	565.1536	-2.83	403.1050 (**100**), 385.0926 (11), 367.0937 (9), 313.0715 (18), 283.0614 (7), 271.0622 (30)	25	Apigenin 8-C-pento-pyranosyl-2′′-*O*-hexoside
P38	6.33	$C_{33}H_{39}O_{18}^+$	M+H	723.2119	-1.66	577.1553 (**100**), 559.1439 (60), 459.1289 (40), 313.0713 (99), 271.0607 (13)	35	Vitexin 4''-(3-hydroxy-3-methylglutaroyl)-2''-*O*-rhamnoside
P39	6.45	$C_{27}H_{29}O_{14}^+$	M+H	577.1535	-2.95	559.1465 (16), 433.1160 (3), 415.1065 (4), 313.0731 (25), 295.0613 (4), 271.0606 (4)	20	Vitexin 4''-(3'''-hydroxy-3'''-methyl-glutarate)
P43	6.67	$C_{39}H_{49}O_{24}^+$	M+H	901.2358	-0.44	739.2129 (13), 637.1618 (17), 577.1625 (31), 559.1639 (9), 325.0961 (**100**), 307.0845 (17), 271.0628 (9), 163.0398 (21)	30	Acylated flavonoid glycoside- A
P47	6.94	$C_{44}H_{49}O_{23}^+$	M+H	945.2641	-1.90	681.1854 (11), 577.1555 (27), 559.1455 (17), 369.1207 (**100**), 351.1087 (41), 313.0715 (28), 271.0605 (10), 225.0740 (9), 207.0651 (58)	40	Acylated flavonoid glycoside- B
P48	6.98	$C_{43}H_{47}O_{22}^+$	M+H	915.2543	-1.09	651.1671 (10), 577.1547 (22), 559.1430 (19), 339.1071 (**100**), 271.0605 (9), 177.0544 (87)	40	Acylated flavonoid glycoside- C
P54	7.57	$C_{43}H_{47}O_{22}^+$	M+H	915.2535	-1.97	651.1821 (21), 577.1538 (51), 559.1480 (30), 339.1105 (19), 271.0581 (30), 177.0524 (**100**)	40	Acylated flavonoid *C*-glycoside- D
P86	10.98	$C_{16}H_{13}O_5^+$	M+H	285.0754	-1.05	285.0764 (14), 270.0519 (31), 242.0573 (**100**), 153.0183 (9)	50	Acacetin
P115	13.53	$C_{17}H_{15}O_5^+$	M+H	299.0908	-2.01	284.0695 (8), 256.0746 (**100**), 241.0511 (5), 227.0714 (3), 213.0551 (5), 197.0608 (3), 167.0344 (14), 124.0154 (8)	50	7-O,4'-O-Dimethyl-apigenin[b]
**Other metabolites**								
P4	3.75	$C_{14}H_{28}NO_{10}^+$	$M+NH_4$	370.1703	-1.35	353.1434 (24), 335.1338 (50), 307.1032 (**100**), 289.0941 (56), 271.0798 (10), 163.0503 (42), 145.0500 (29), 127.0375 (13), 103.0375 (7), 85.0293 (4)	15	Formyl-(3-Hydroxy-3-methylglutaryl) hexopyranoside
P8	4.37	$C_{13}H_{19}O_6^+$	M+H	271.1177	0.37	197.0677 (9), 179.0677 (9), 151.0370 (7), 147.0649 (6), 125.0586 (19)	15	Benzyl-*β*-*D*-glucoside[b]
P13	4.68	$C_{17}H_{13}O_3^+$	M+H	265.0860	0.38	250.0603 (27), 237.0907 (34), 222.0672 (21), 219.0905 (39), 209.0977 (47), 191.0835 (21)	25	Quinone derivative; salvinolactone
P14	4.68	$C_{18}H_{17}O_4^+$	M+H	297.1119	-0.67	282.0888 (68), 265.0854 (46), 237.0904 (63), 219.0828 (25), 209.0980 (31), 207.0800 (31), 191.0864 (25), 165.0696 (20)	30	Tanshinol B
P18	4.98	$C_9H_7O_2^+$	M+H	147.0440	-0.68	119.0497 (**100**), 91.0550 (86), 65.0393 (5)	20	Coumarin
P19	4.99	$C_{17}H_{13}O_3^+$	M+H	265.0858	-0.38	250.0969 (**100**), 237.0905 (37), 233.0597 (30), 222.0691 (9), 205.0655 (22)	25	Quinone derivative (isomer)
P20	4.99	$C_{18}H_{17}O_4^+$	M+H	297.1119	-0.67	282.0875 (48), 267.0670 (14), 265.0962 (**100**), 254.0936 (17), 250.0629 (18), 247.0746 (14), 239.0725 (14), 237.0926 (54), 233.0617 (48), 222.0697 (17), 209.0952 (10), 205.0653 (61)	30	Tanshinol B (isomer)
P23	5.11	$C_{19}H_{31}O_8^+$	M+H	387.2008	-1.29	225.1479 (30),207.1387 (**100**),189.1274 (6),149.0944 (6),113.0607 (7)	15	Roseoside[b]
P28	5.39	$C_{16}H_{32}NO_{10}^+$	$M+NH_4$	398.2014	-1.76	381.1735 (4), 363.1659 (47), 307.1029 (**100**), 289.0922 (14), 271.0802 (4), 187.0610 (3), 163.0605 (47), 145.0482 (19), 127.0386 (7), 103.0393 (4)	15	Butyl-(3-Hydroxy-3-methylglutaryl) hexopyranoside
P33	5.89	$C_{22}H_{36}NO_{13}^+$	$M+NH_4$	522.2167	-2.68	505.1856 (1), 360.1673 (4), 343.1417 (33), 325.1153 (65), 289.0922 (6), 181.0874 (**100**), 163.0593 (24), 145.0493 (13), 140.0464 (4), 127.0393 (3), 85.0279 (1)	15	Coniferinoside
P35	6.02	$C_{28}H_{44}NO_{13}^+$	$M+NH_4$	602.2806	-0.17	585.2547 (1), 423.2019 (**100**), 405.1911 (27), 387.1793 (4), 355.1529 (2), 251.1277 (1), 193.0843 (1), 167.0697 (1)	15	8,8'-Bisdihydro-siringenin hexoside
P40	6.53	$C_{11}H_{17}O_3^+$	M+H	197.1168	-2.03	179.1072 (29), 161.0957 (4), 135.1168 (11), 133.1006 (3), 107.0856 (3)	15	Loliolide[b]
P45	6.77	$C_{20}H_{32}NO_{10}^+$	$M+NH_4$	446.2014	-1.57	429.1784 (7), 411.1678 (22), 307.1042 (**100**), 289.0933 (13), 271.0824 (3), 163.0604 (22), 145.0494 (10), 127.0390 (6)	15	Undatuside C

Table S1: *Cont.*

P49	7.33	$C_{11}H_{11}O_4^+$	M+H	207.0645	-3.38	192.0414 (31), 191.0337 (61), 179.0699 (9), 163.0395 (66), 151.0747 (**100**), 136.0513 (24), 121.0645 (14), 107.0493 (17), 91.0529 (11)	30	Scoparone[b]
P51	7.46	$C_{12}H_{15}O_3^+$	M+H	207.1012	-1.93	192.0775 (7), 177.0906 (7), 176.0837 (**100**), 161.0591 (15)	20	3,4-Dimethoxy-benzalacetone
P53	7.50	$C_{22}H_{37}O_{11}^+$	M+H	477.2318	-2.51	459.2245 (7), 441.2163 (3), 395.1535 (3), 339.1859 (3), 307.1057 (20), 289.0906 (5), 171.1391 (**100**), 163.0600 (12), 153.1259 (5), 145.0518 (4), 135.1135 (3), 107.0837 (3), 93.0694 (4)	15	Zingiberoside C
P56	7.72	$C_{10}H_{13}O_4^+$	M+H	197.0809	0.51	182.0578 (13), 169.0865 (54), 167.0336 (15), 154.0622 (60), 139.0390 (19), 138.0675 (**100**), 123.0441 (15)	25	Asarylaldehyde[b]
P67	9.53	$C_{11}H_{11}O_4^+$	M+H	207.0648	-1.93	179.0635 (35), 177.0544 (48), 175.0399 (17), 164.0455 (35), 151.0387 (81), 149.0602 (**100**), 147.0458 (38), 134.0346 (17), 121.0259 (14), 119.0488 (57), 91.0536 (30)	25	3-(7-Methoxy-1,3-benzodioxol-5-yl)prop-2-enal
P85	10.96	$C_{11}H_{13}O_4^+$	M+H	209.0804	-1.91	193.0509 (9), 179.0359 (**100**), 149.0903 (7), 135.0443 (7), 134.0379 (3), 121.0654 (11), 107.0496 (5), 106.0417 (9), 105.0345 (4), 91.0554 (21), 79.0550 (7), 77.0394 (8)	35	Methyl ferulate
P89	11.45	$C_{12}H_{17}O_3^+$	M+H	209.1166	-2.87	194.0961 (**100**), 181.0886 (54), 179.0717 (83), 178.1007 (53), 177.0925 (20), 168.0794 (8), 163.0769 (20), 151.0765 (51), 149.0605 (12), 121.0666 (30), 91.0550 (7)	25	Asarone (isomer-A)
P90	11.49	$C_{22}H_{27}O_6^+$	M+H	387.1796	-1.55	327.1805 (79), 225.1117 (73), 219.1020 (6), 163.0746 (58), 121.0647 (**100**)	15	Eudesmin
P94	11.68	$C_{12}H_{17}O_3$	M+H	209.1168	-1.91	194.0946 (15), 181.0864 (7), 178.0996 (15), 177.0909 (3), 168.0802 (**100**), 162.0679 (3), 153.0552 (22), 151.0760 (1), 125.0594 (1)	20	Isoasarone[b]
P95	11.87	$C_{12}H_{17}O_3^+$	M+H	209.1171	-0.48	194.0950 (46), 181.0869 (24), 179.0726 (**100**), 178.0997 (26), 177.0914 (6), 163.0757 (18), 162.0678 (8), 151.0763 (73), 147.0790 (8), 136.0520 (17), 123.0447 (15), 121.0648 (20), 107.0495 (7), 91.0543 (15)	30	*trans*-Asarone[b]
P96	11.88	$C_{24}H_{33}O_7^+$	M+H	433.2209	-2.77	265.1448 (92), 247.1337 (98), 237.1491 (19), 225.1104 (14), 216.1150 (36), 209.1184 (69), 197.0803 (27), 181.0868 (**100**)	25	Grandisin
P122	14.45	$C_{24}H_{33}O_6^+$	M+H	417.2267	-1.20	385.1992 (5), 249.1497 (**100**), 217.1229 (39), 209.1178 (29), 181.0860 (17)	15	Andamanicin[b]
P126	14.72	$C_{24}H_{33}O_6^+$	M+H	417.2276	0.96	385.2042 (8), 249.1508 (**100**), 209.1176 (14), 181.0846 (6)	15	Heterotropan
P127	14.83	$C_{24}H_{33}O_6^+$	M+H	417.2267	-1.20	249.1500 (**100**), 181.0851 (3)	15	Magnosalin[b]
P130	15.13	$C_{12}H_{17}O_3^+$	M+H	209.1169	-1.43	194.0951 (**100**), 181.0968 (82), 179.0710 (79), 178.0997 (46), 177.0935 (12), 168.0794 (7), 163.0767 (13), 162.0670 (12), 151.0756 (60), 147.0813 (10), 136.0515 (12), 135.0903 (12), 123.0436 (12), 121.0661 (12), 91.0531 (9)	25	Asarone (isomer-B)

[a] Relative intensity of parent ion is 100% if none of fragment ions stated
[b] Confirmed by co-elution with authentic standards (isolated compounds with the structure confirmed by NMR spectroscopy).

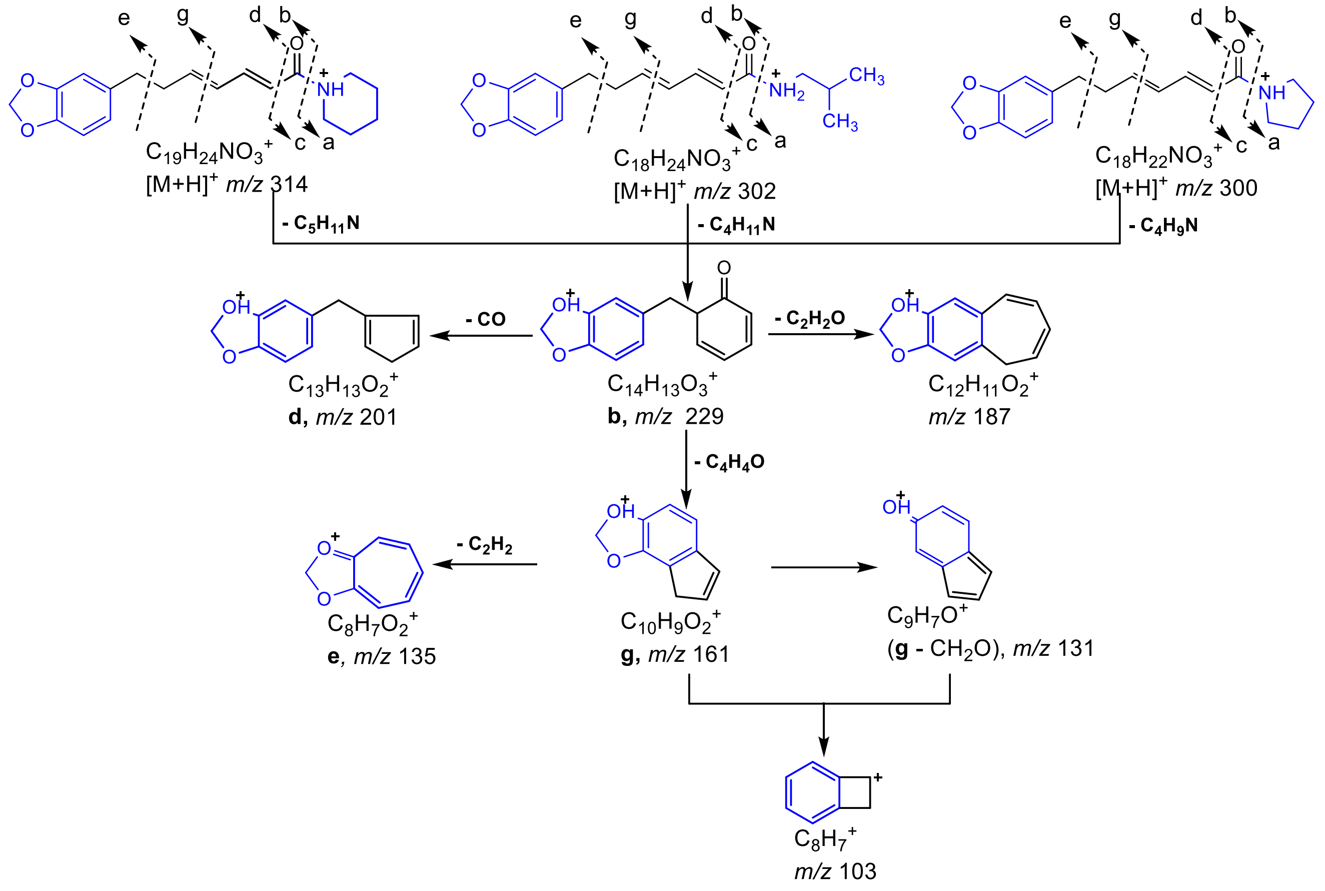

Figure S4: The general fragmentation patterns of selected piperamides P108 (*m/z* 314), P105 (*m/z* 302), and P93 (*m/z* 300).

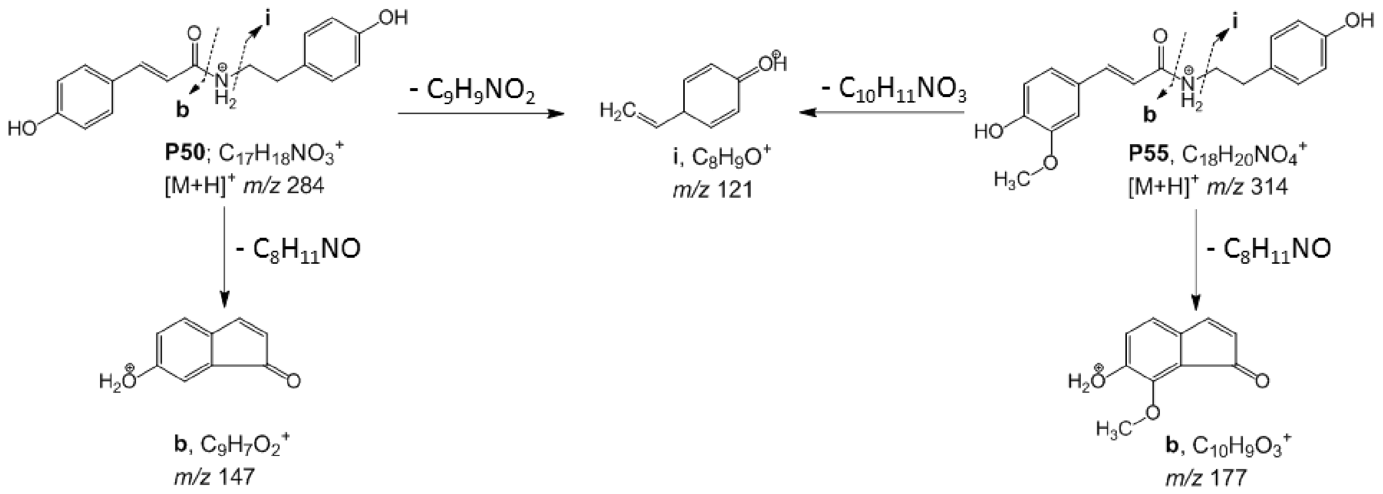

Figure S5: Key characteristics product ions **b** and **i** of paprazine (P50), and *trans*-*N*-feruloyltyramine (P55).

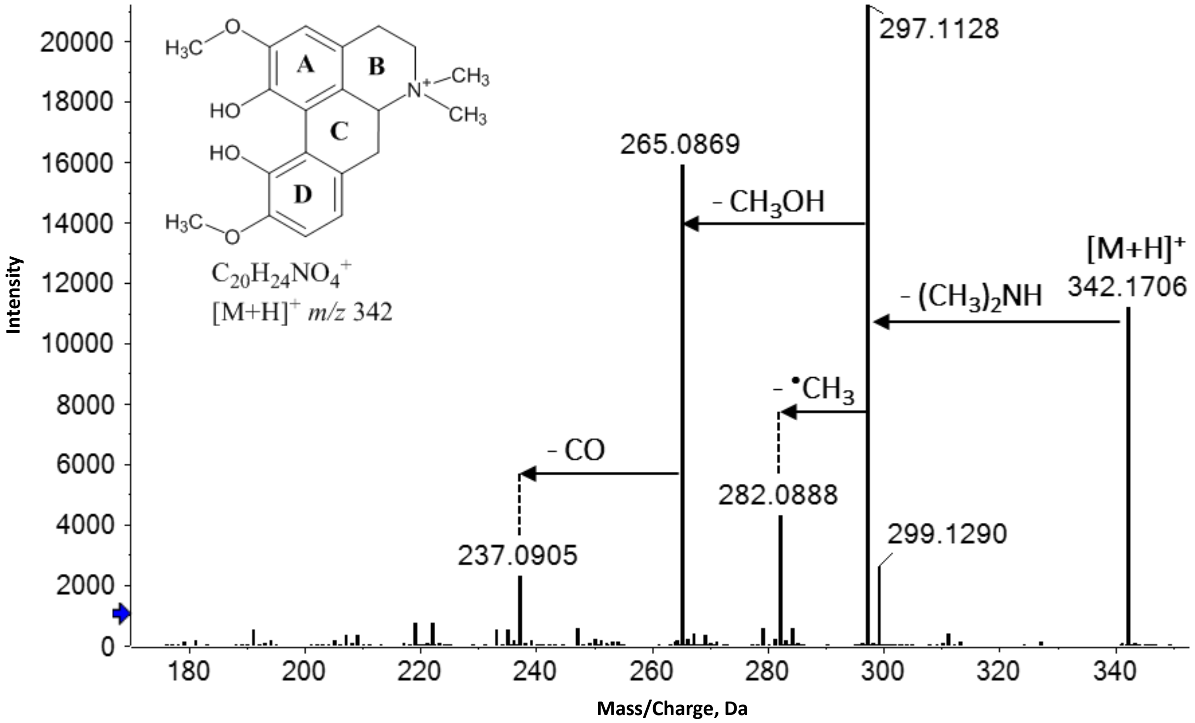

Figure S6: MS/MS spectrum of magnoflorine (P12).

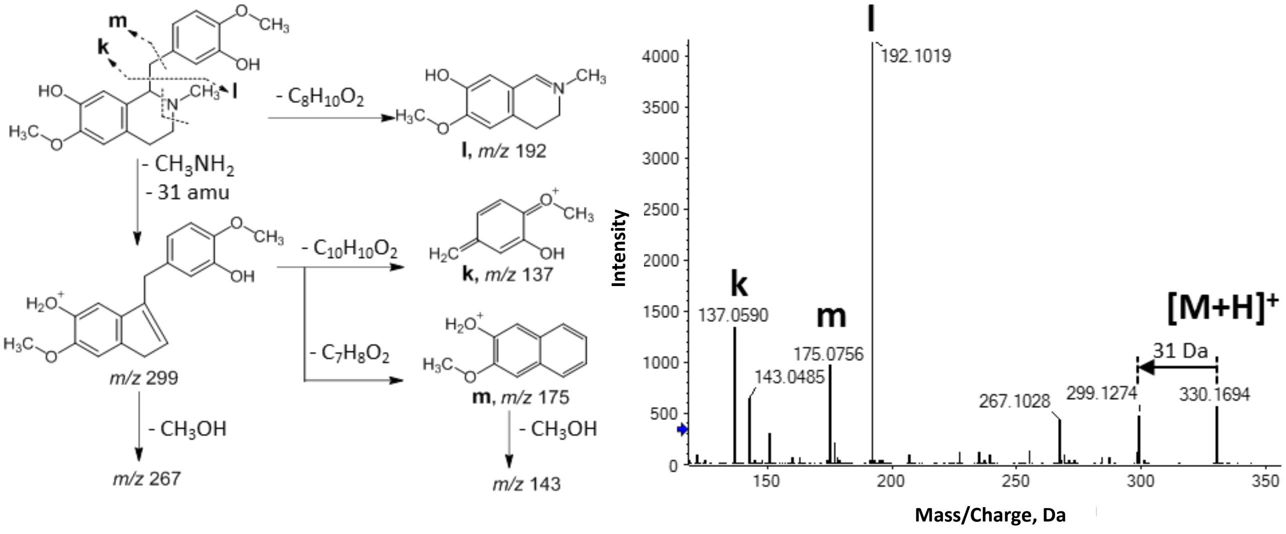

Figure S7: Proposed fragmentation pathway and MS[2] spectra of reticuline (P16).

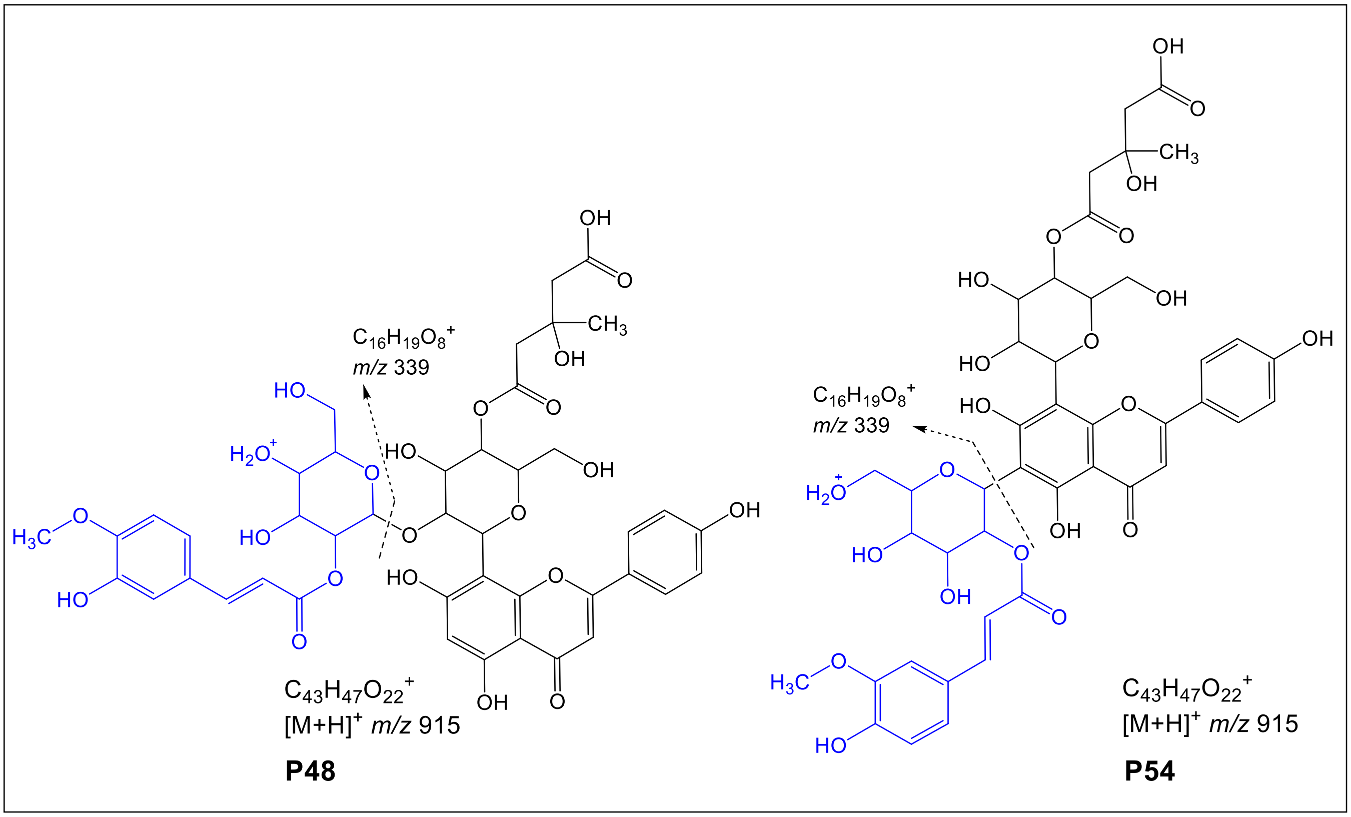

Figure S8: Proposed structures of flavone derivatives giving peaks P48 and P54 with their characteristic fragment ion at *m*/*z* 339 in blue.

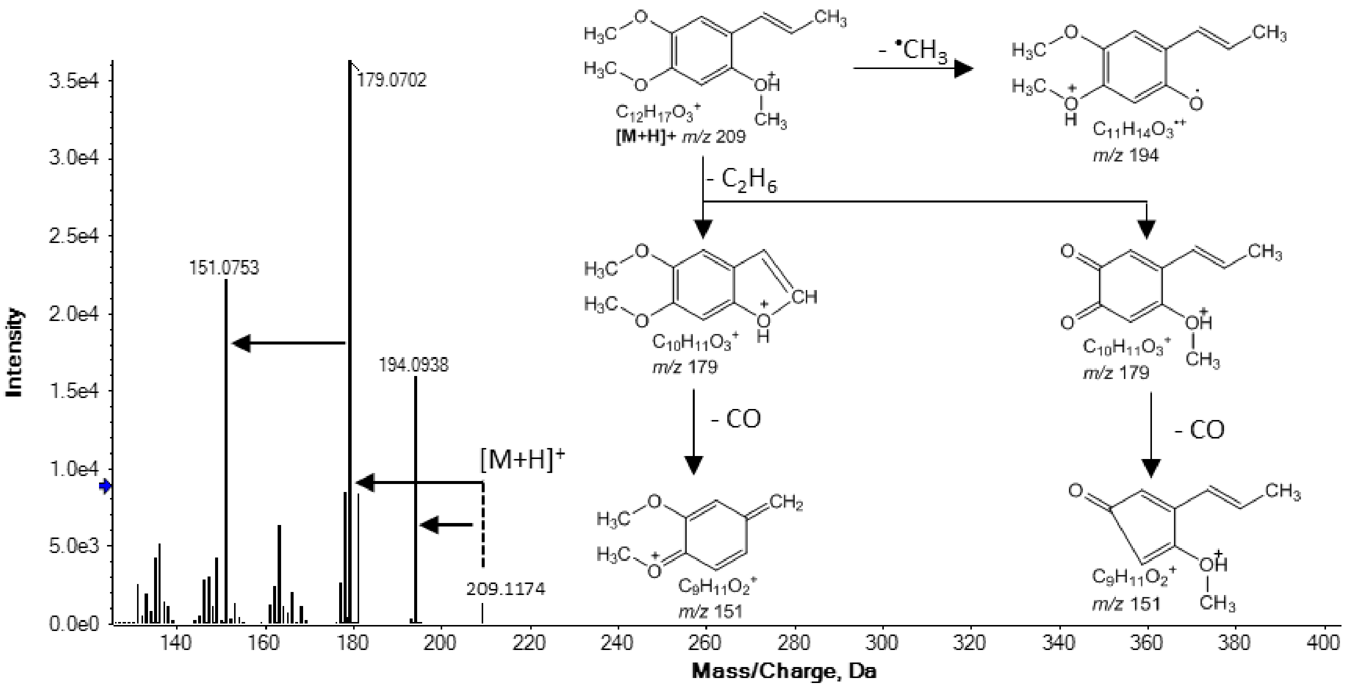

Figure S9: MS/MS fragmentation of the $[M+H]^+$ ion of *trans*-asarone (P95).

Figure S10: MSMS spectra of peaks P1-154. Compounds identified by authentic standards (isolated compounds with the structure confirmed by NMR spectroscopy) are marked with*.

P1, Fructosyl-phenylalanine

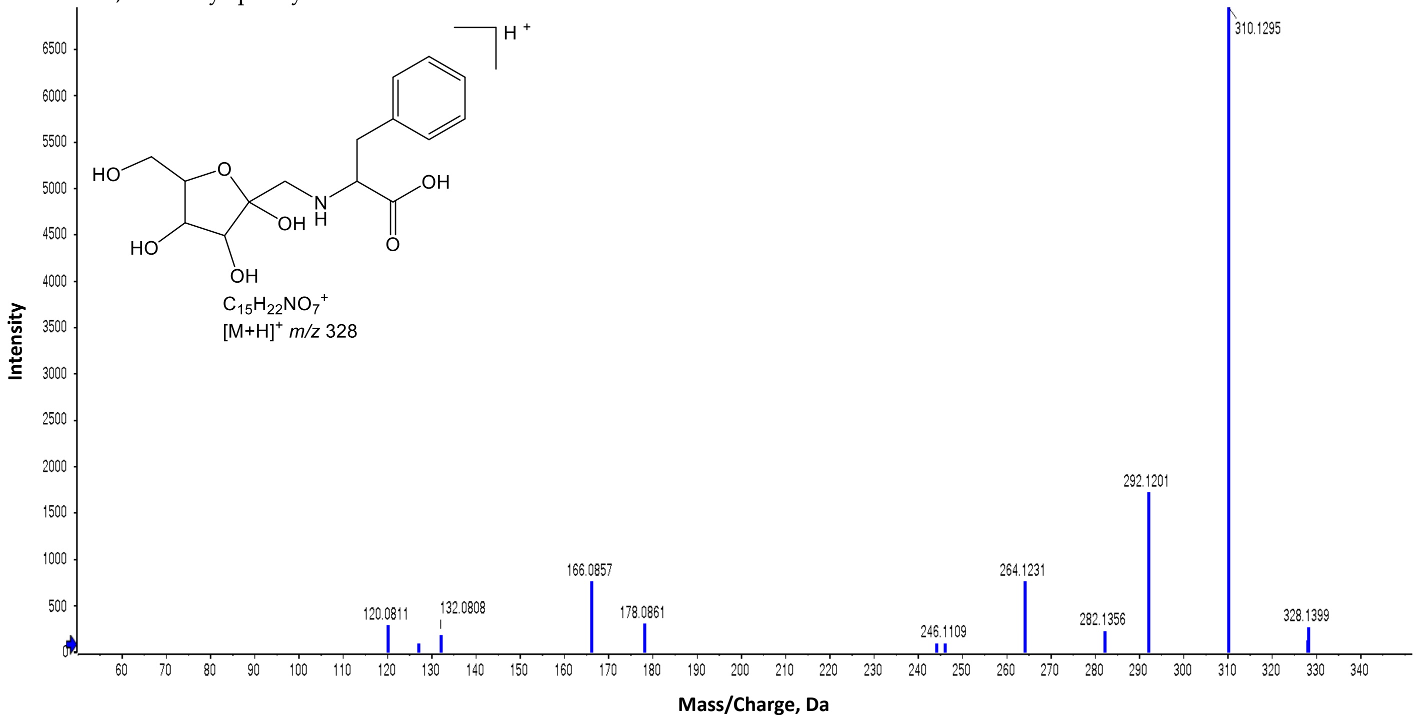

Figure S10_1: MS/MS spectrum of P1.

P2, Naphthisoxazol A*

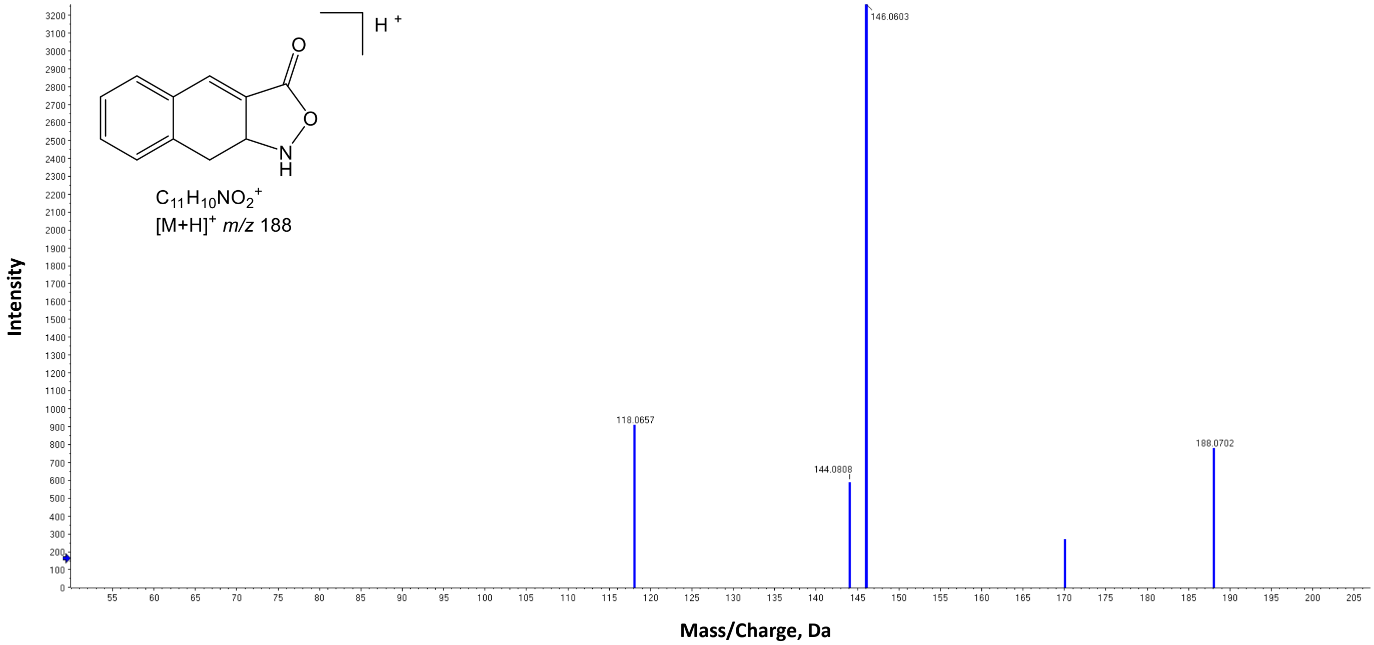

Figure S10_2: MS/MS spectrum of P2.

P3, Naphthisoxazol A

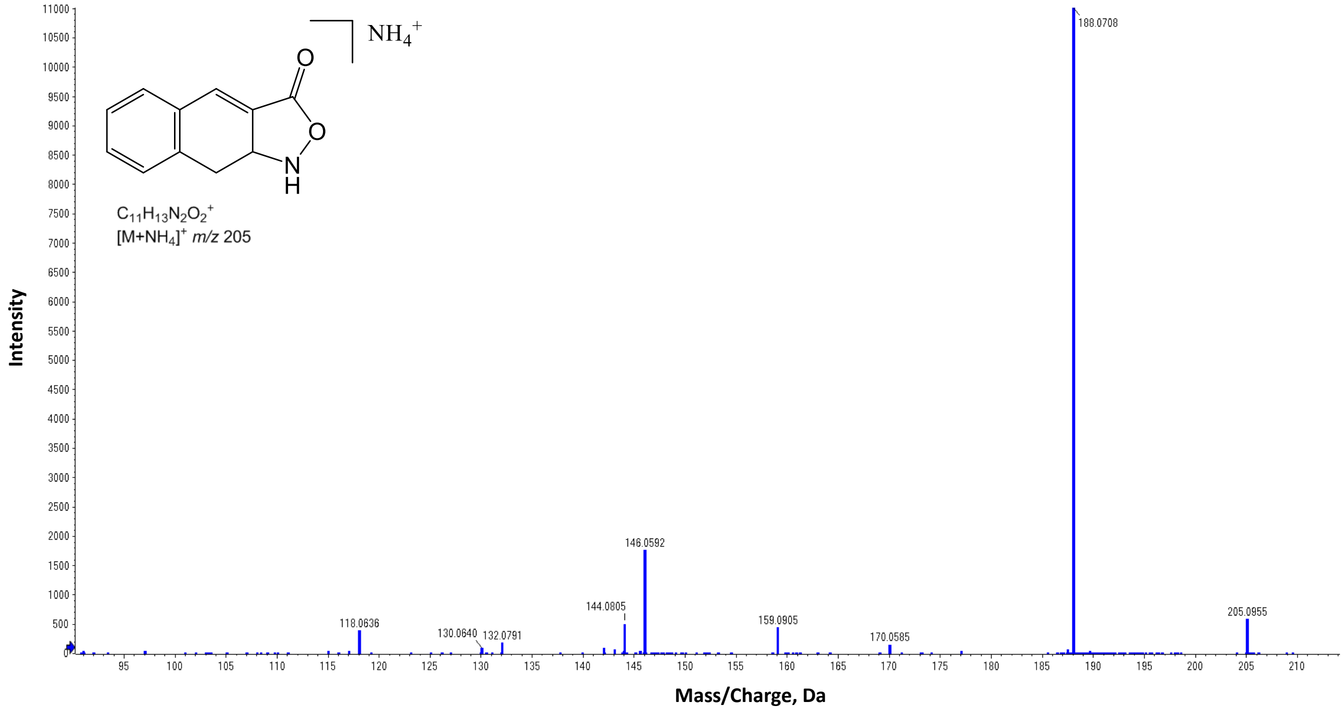

Figure S10_3: MS/MS spectrum of P3.

P4, Formyl-(3-Hydroxy-3-methylglutaryl) hexopyranoside

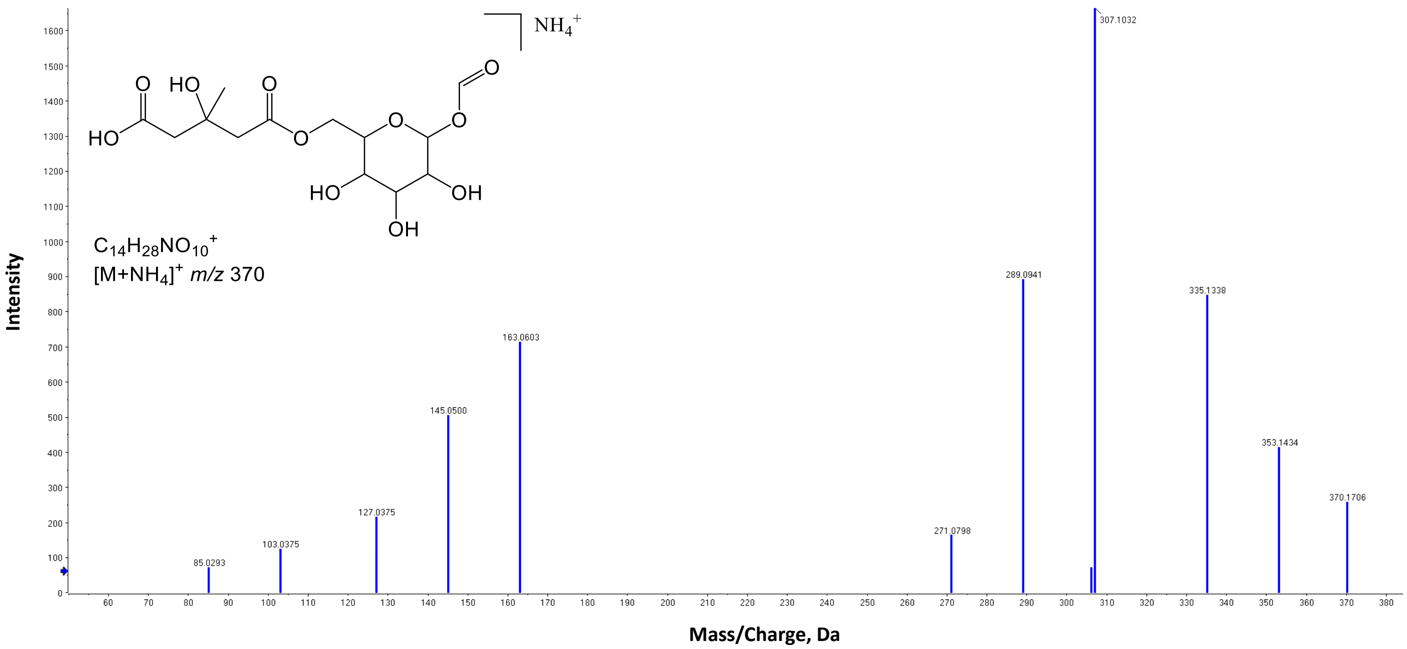

Figure S10_4: MS/MS spectrum of P4.

P5: $C_{19}H_{24}NO_3^+$, isomer of armepavine (formula see **P22**)

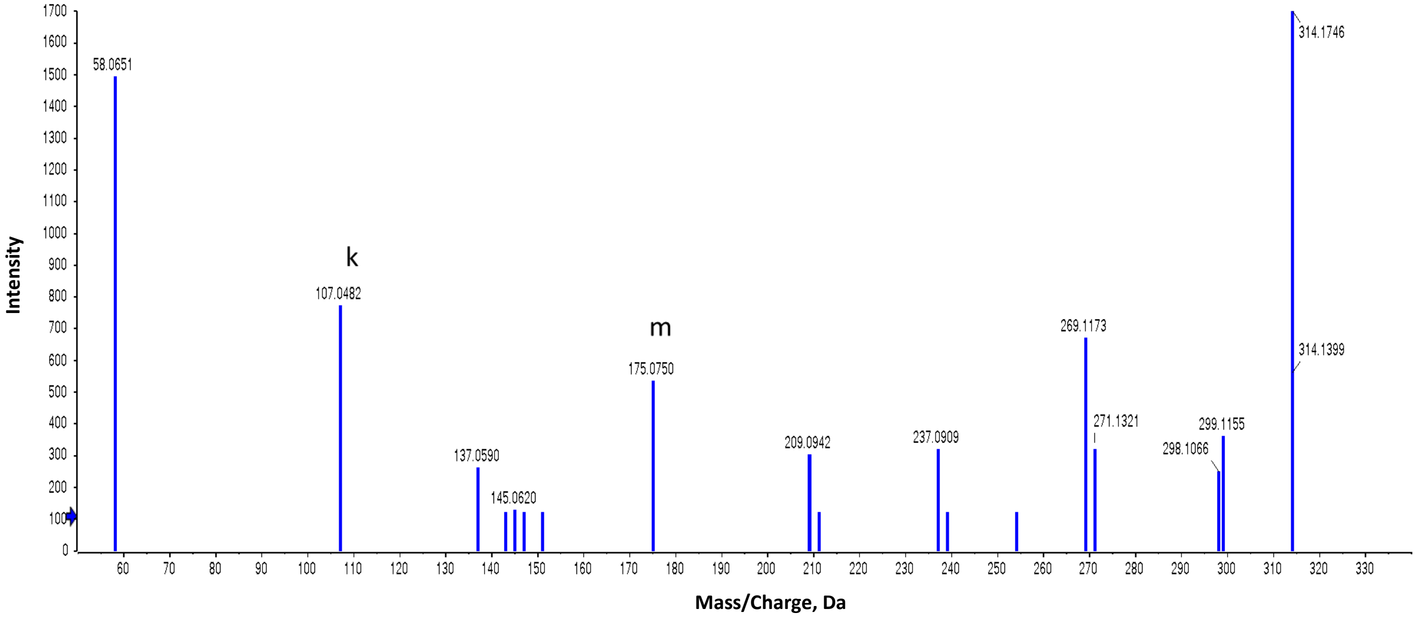

Figure S10_5: MS/MS spectrum of P5.

P6, Dimetolquinol

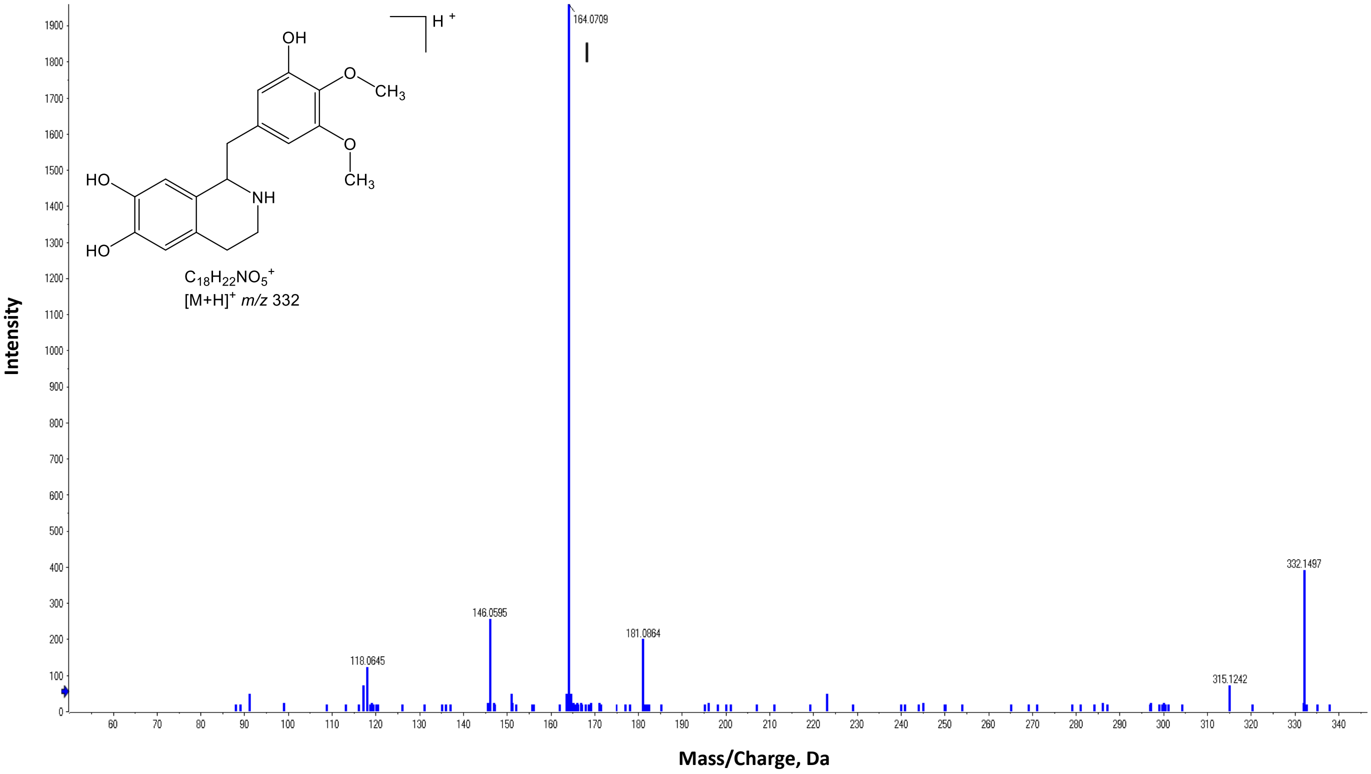

Figure S10_6: MS/MS spectrum of P6.

P7: $C_{23}H_{30}NO_8^+$, isomer of isococlaurine *O*-glucoside (formula see **P10**)

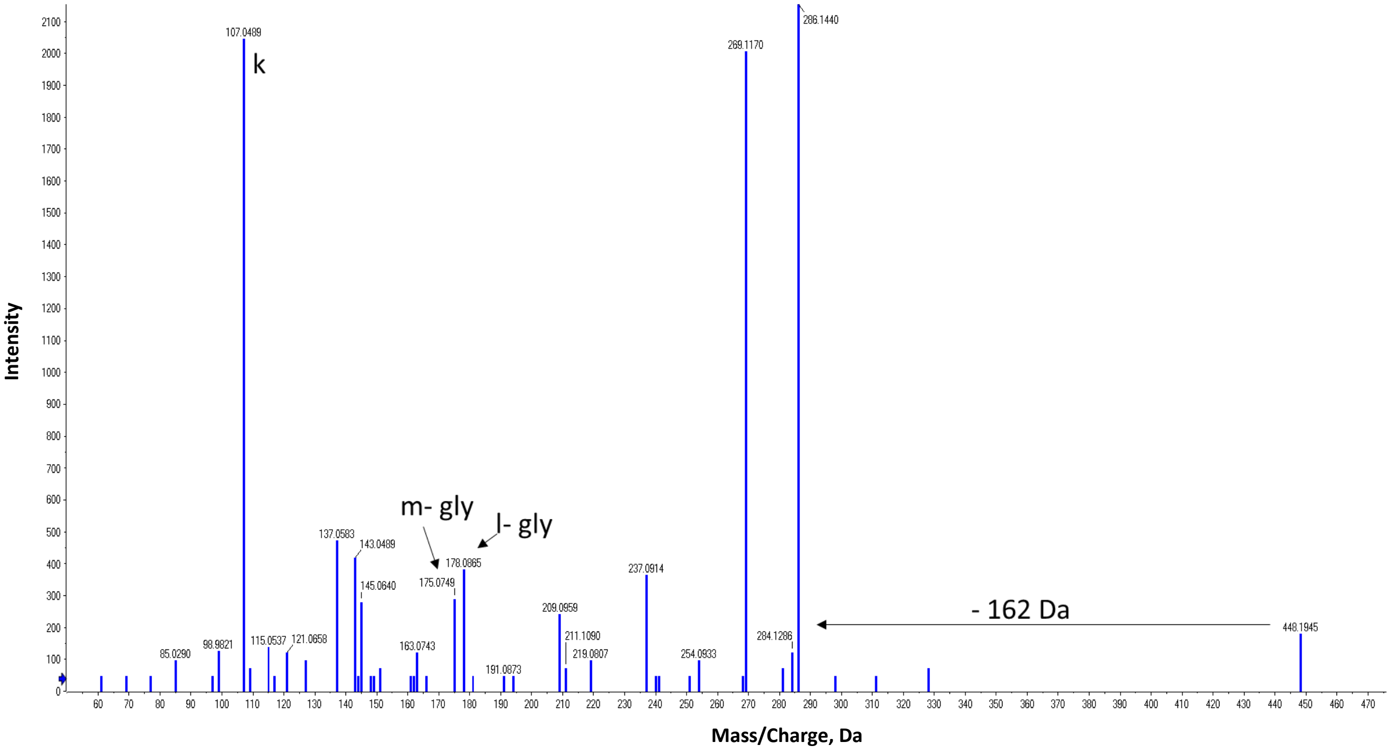

Figure S10_7: MS/MS spectrum of P7.

P8, Benzyl-*β*-D-glucoside*

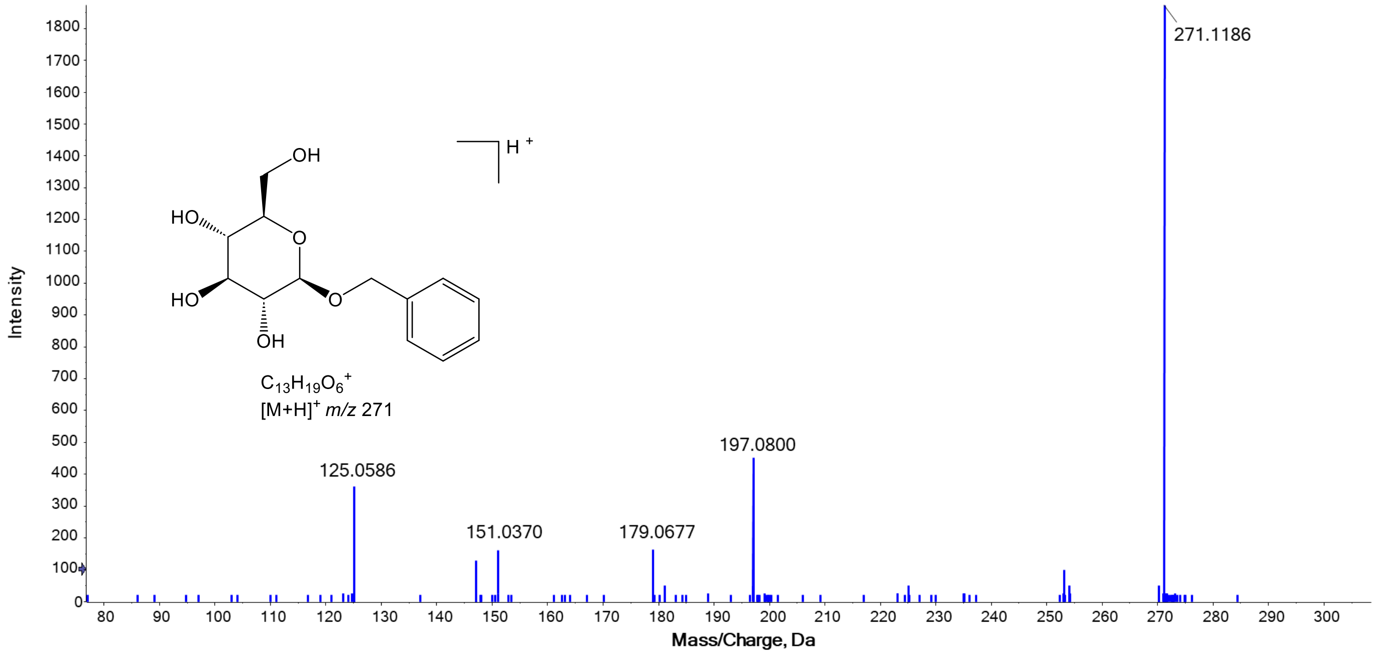

Figure S10_8: MS/MS spectrum of P8.

P9, Coclaurine

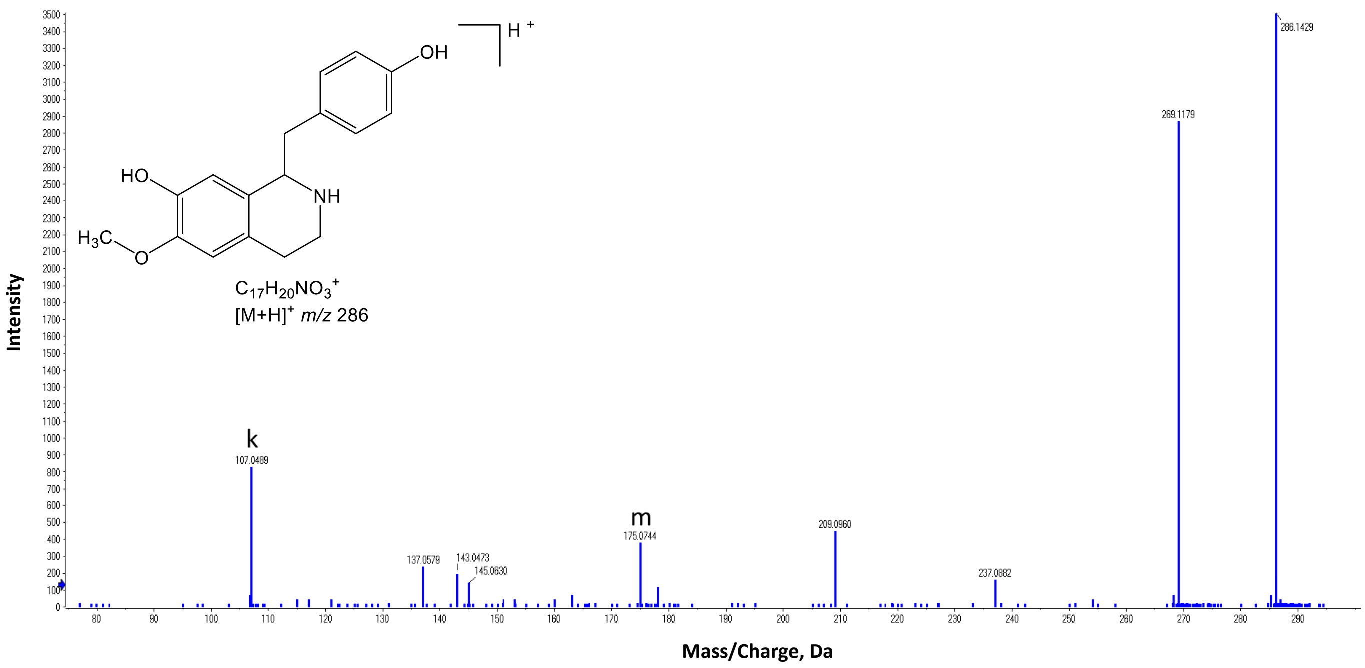

Figure S10_9: MS/MS spectrum of P9.

P10, (Iso)coclaurine *O*-hexoside

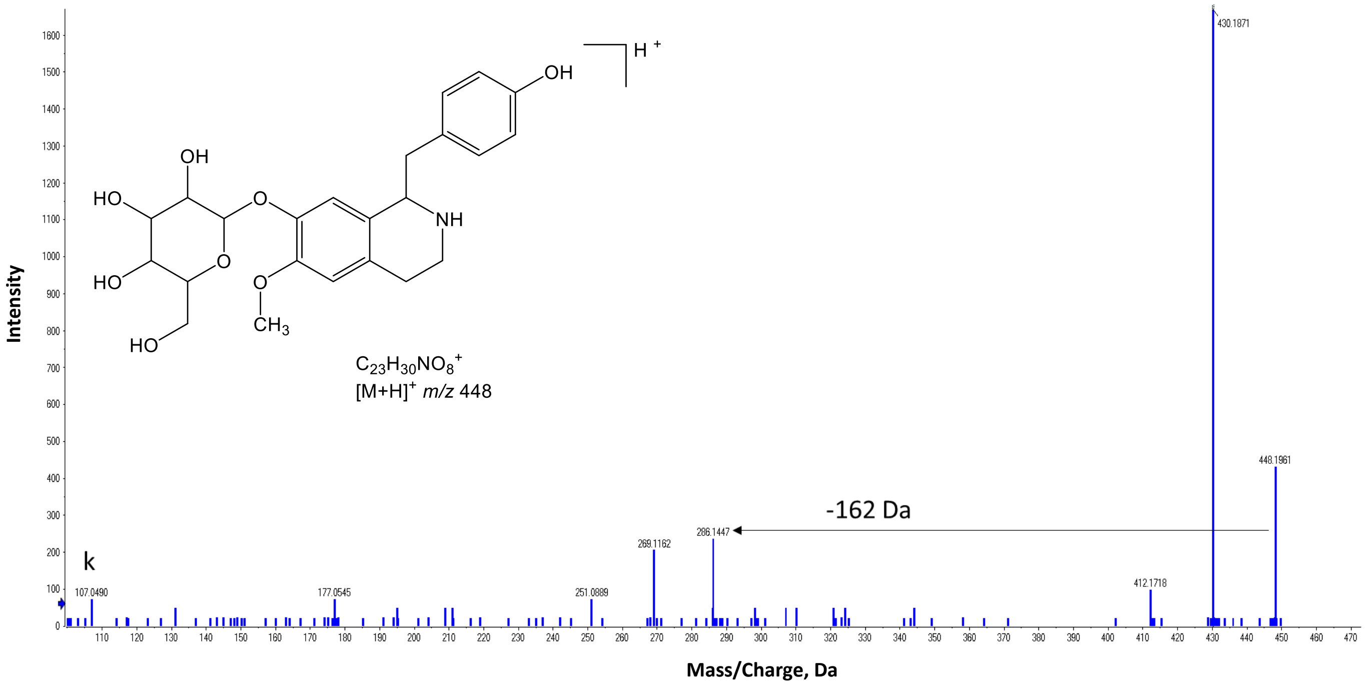

Figure S10_10: MS/MS spectrum of P10.

P11, Oleracein E

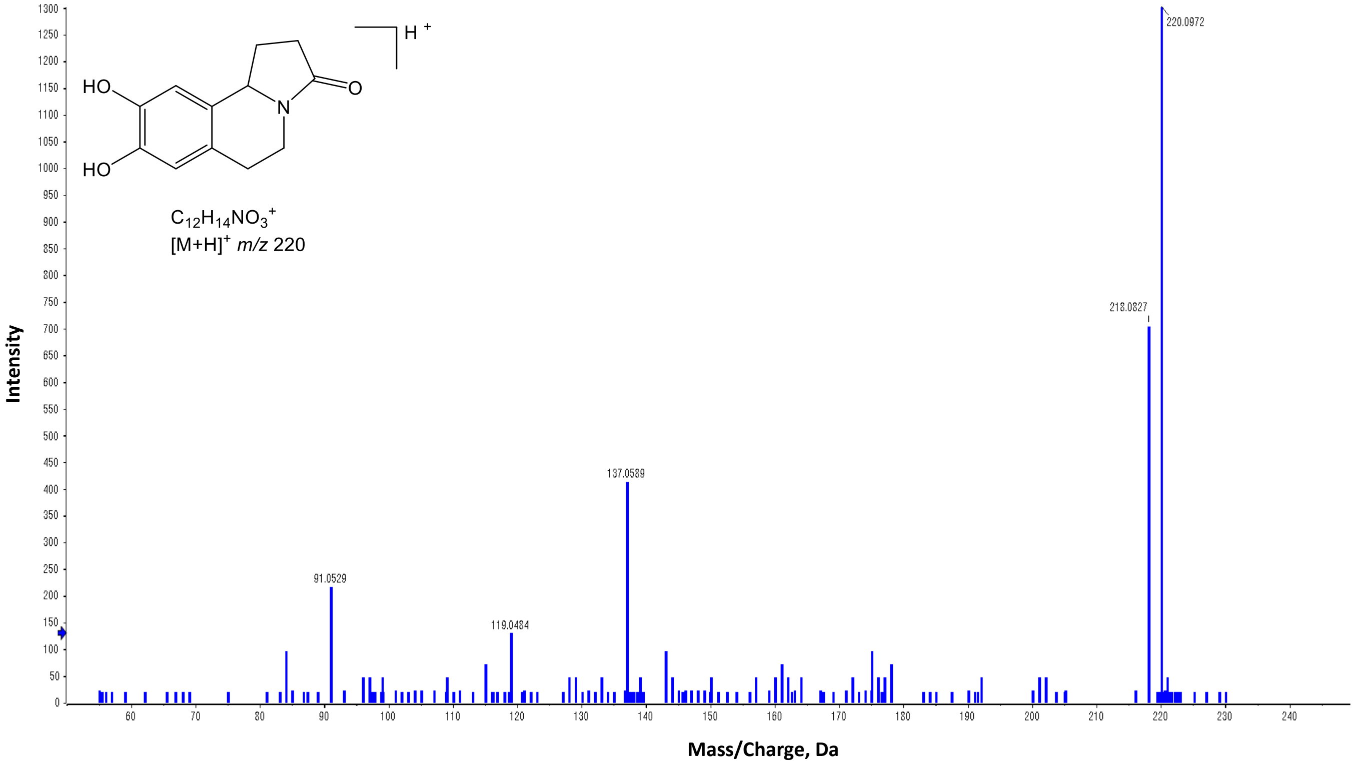

Figure S10_11: MS/MS spectrum of P11.

P12, Magnoflorine

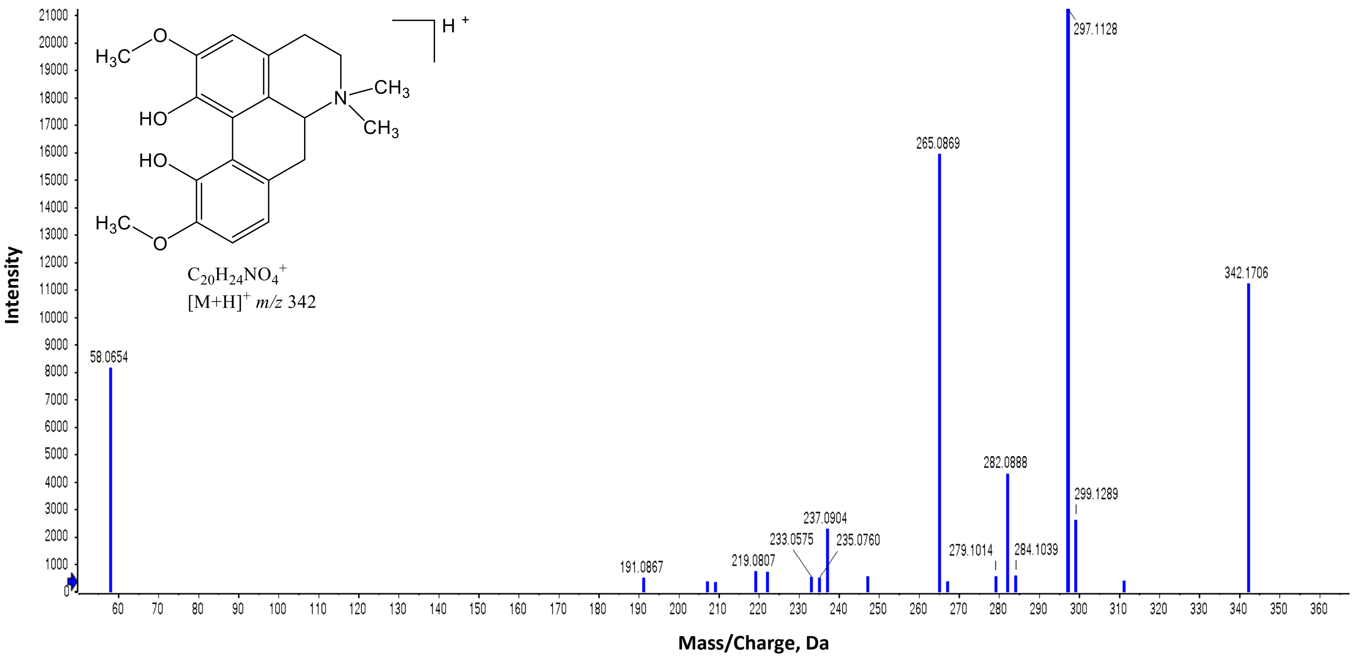

Figure S10_12: MS/MS spectrum of P12.

P13, Quinone derivative, salvinolactone

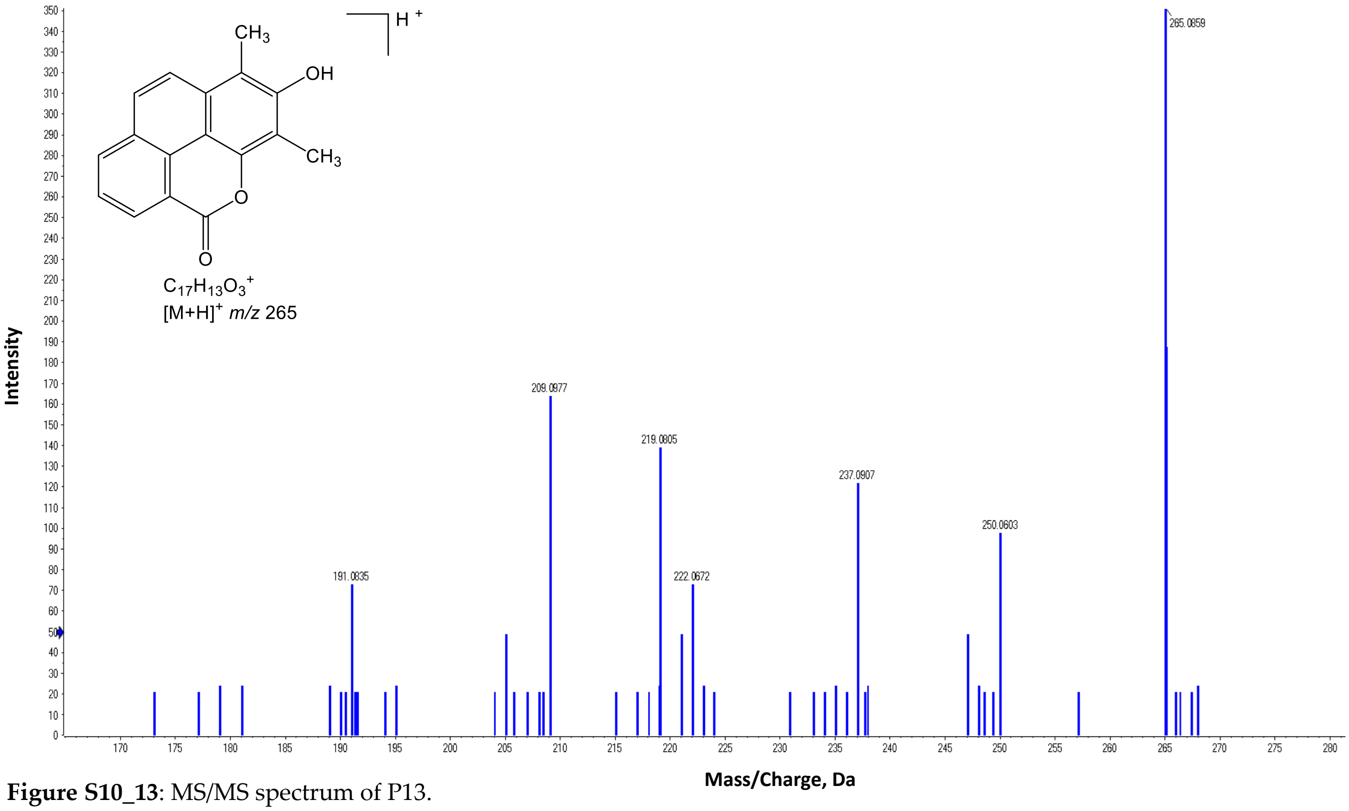

Figure S10_13: MS/MS spectrum of P13.

P14, Tanshinol B

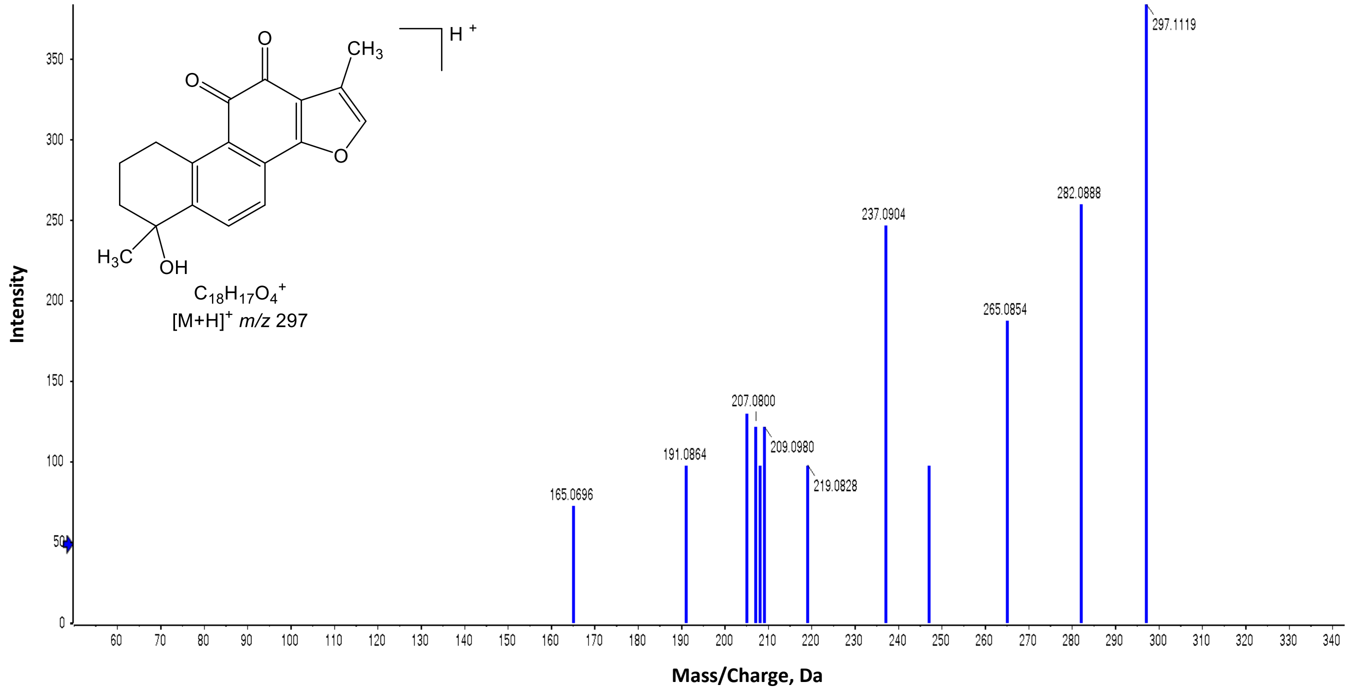

Figure S10_14: MS/MS spectrum of P14.

P15, Xanthoplanine

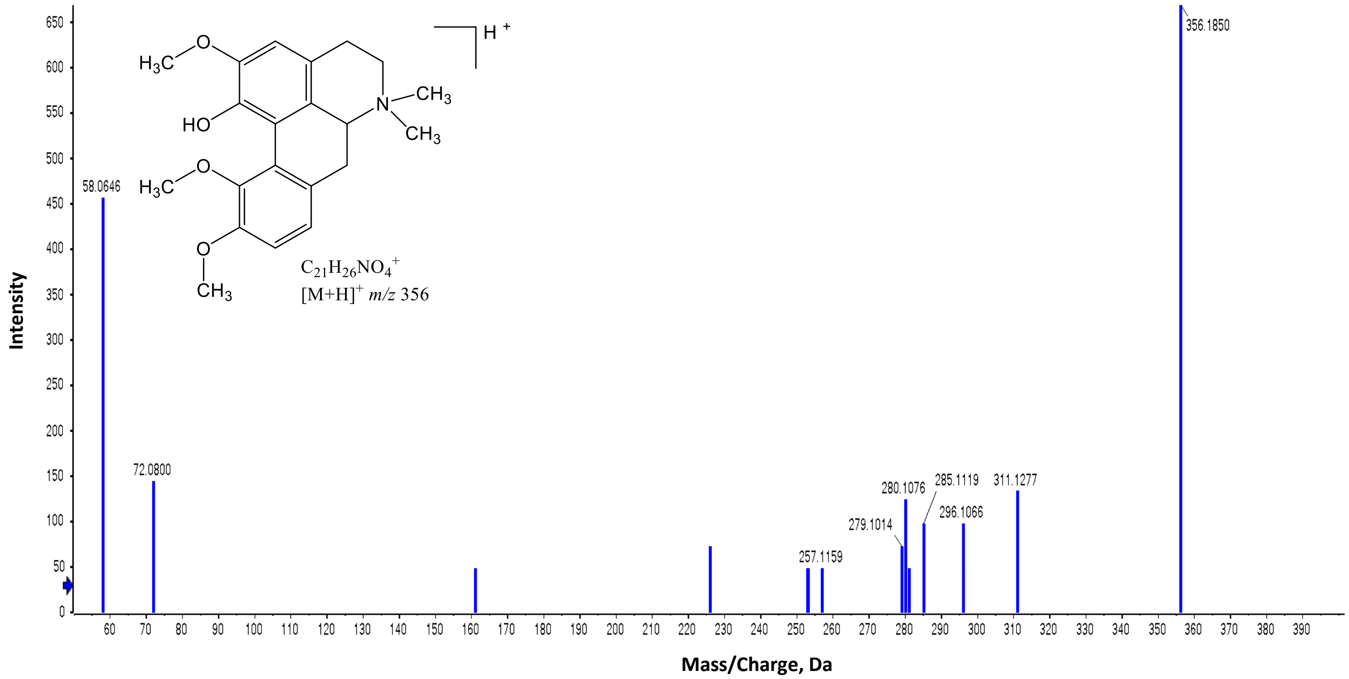

Figure S10_15: MS/MS spectrum of P15.

P16, Reticuline

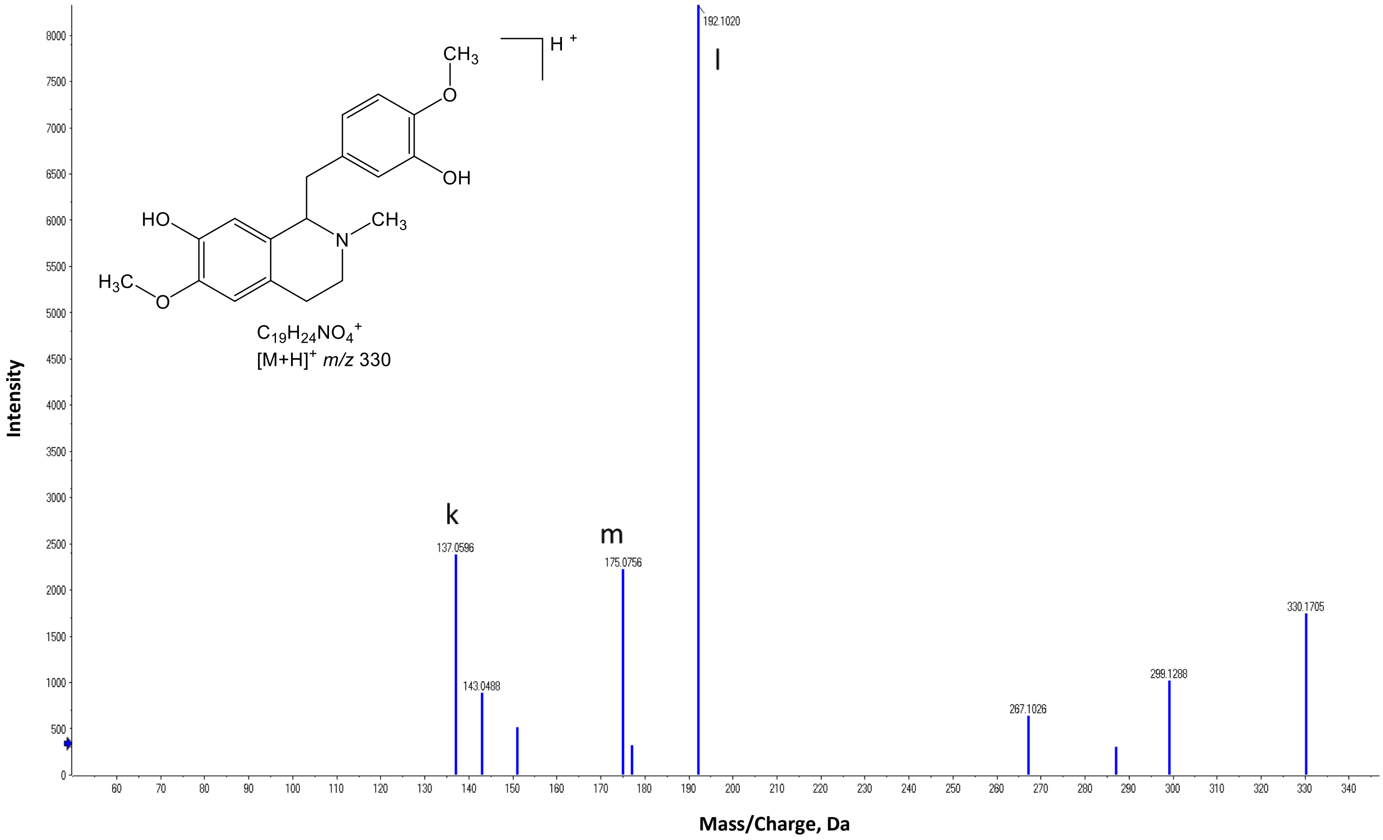

Figure S10_16: MS/MS spectrum of P16.

P17: $C_{20}H_{22}NO_4^+$, isomer of dehydroliriofernine (formula see **P25**)

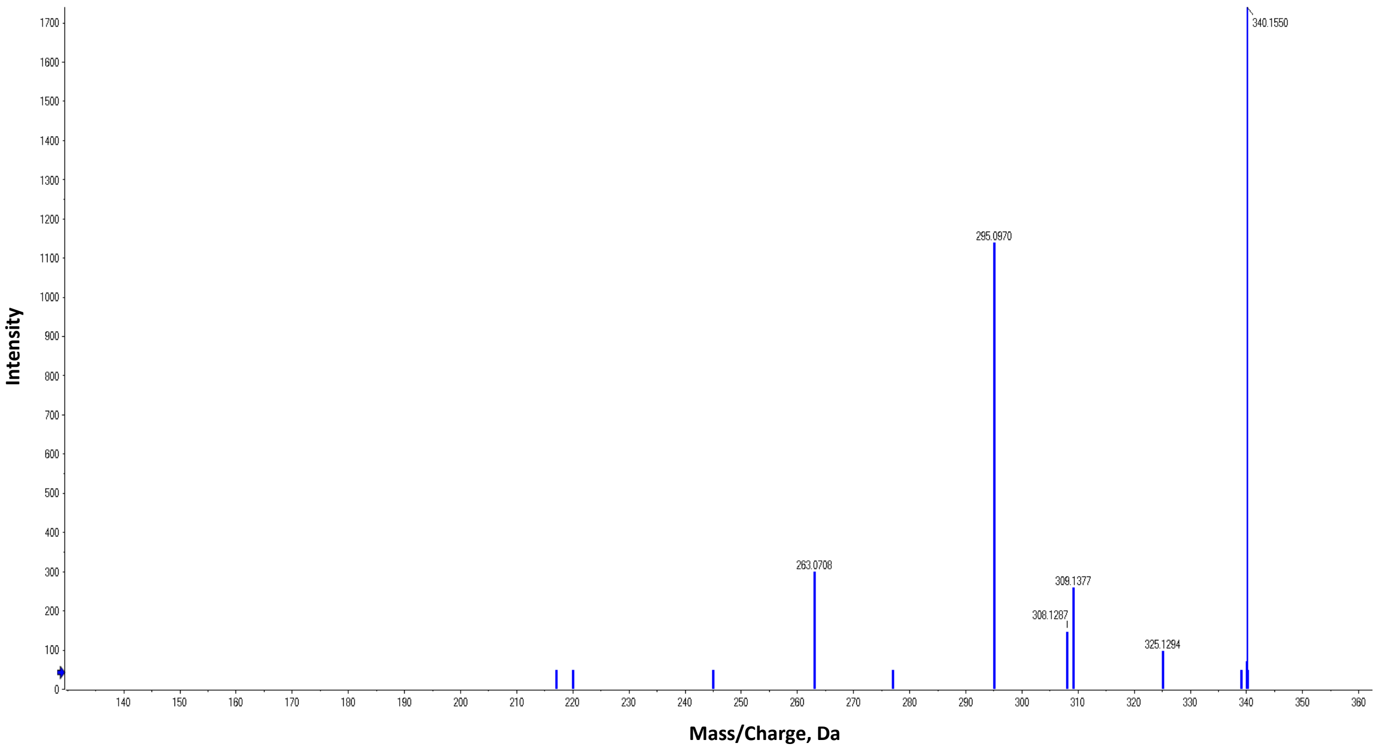

Figure S10_17: MS/MS spectrum of P17.

P18, Coumarin

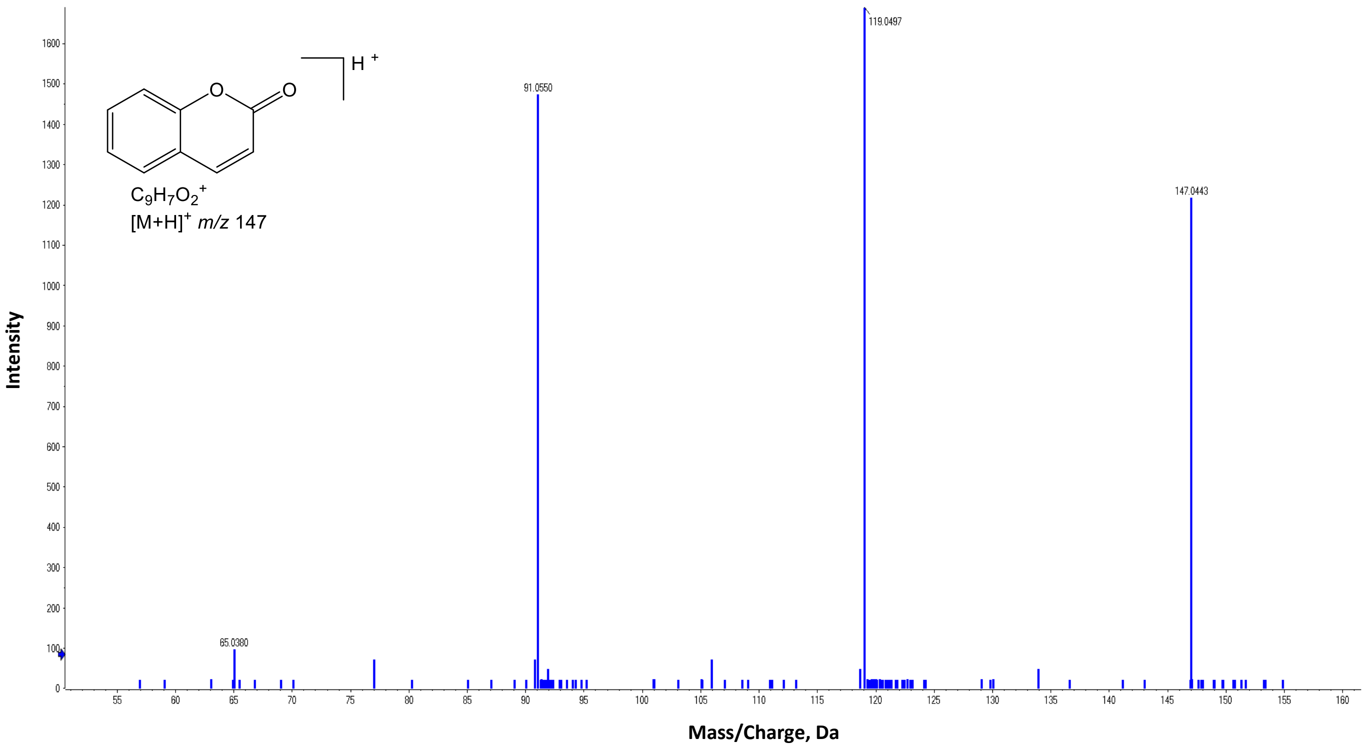

Figure S10_18: MS/MS spectrum of P18.

P19: $C_{17}H_{13}O_3^+$, isomer of quinone derivative **P13** (formula see **P13**)

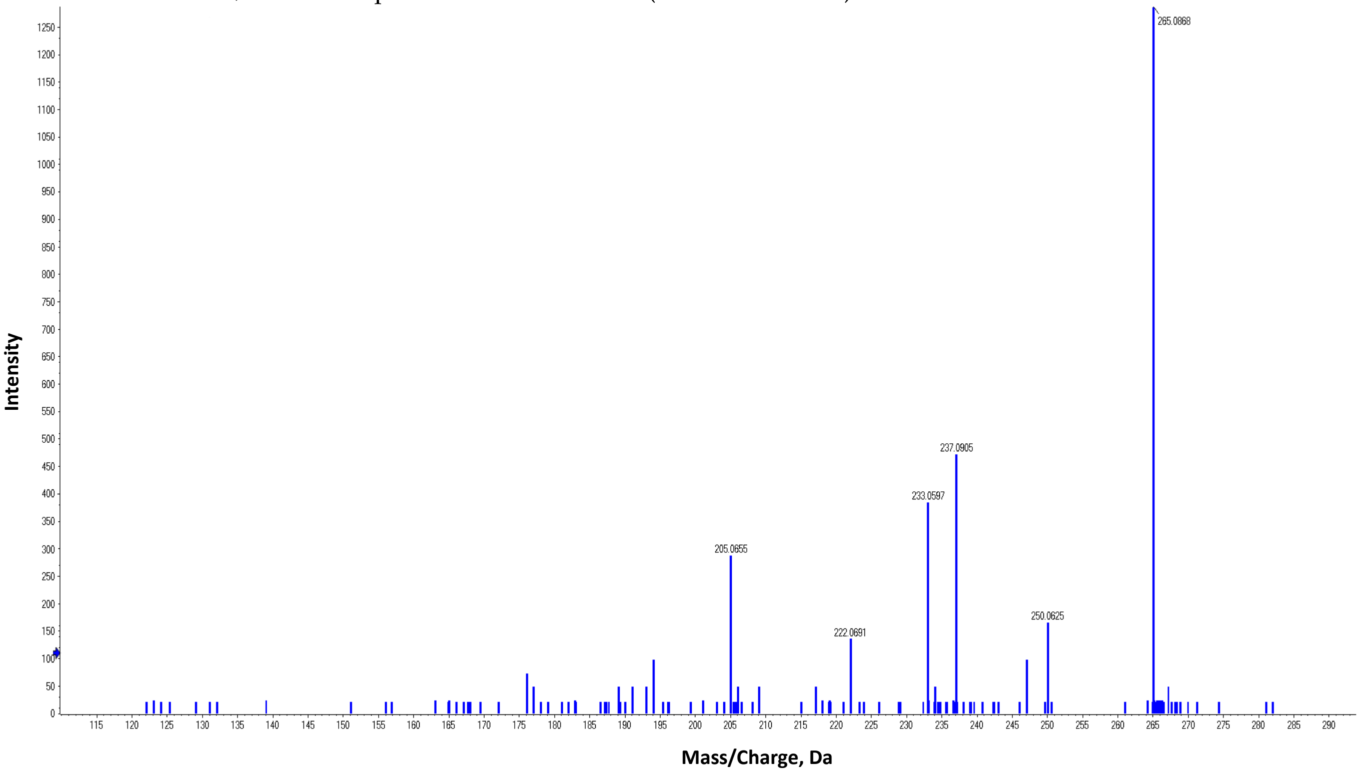

Figure S10_19: MS/MS spectrum of P19.

P20: $C_{18}H_{17}O_4^+$, isomer of tanshinol B (formula see **P14**)

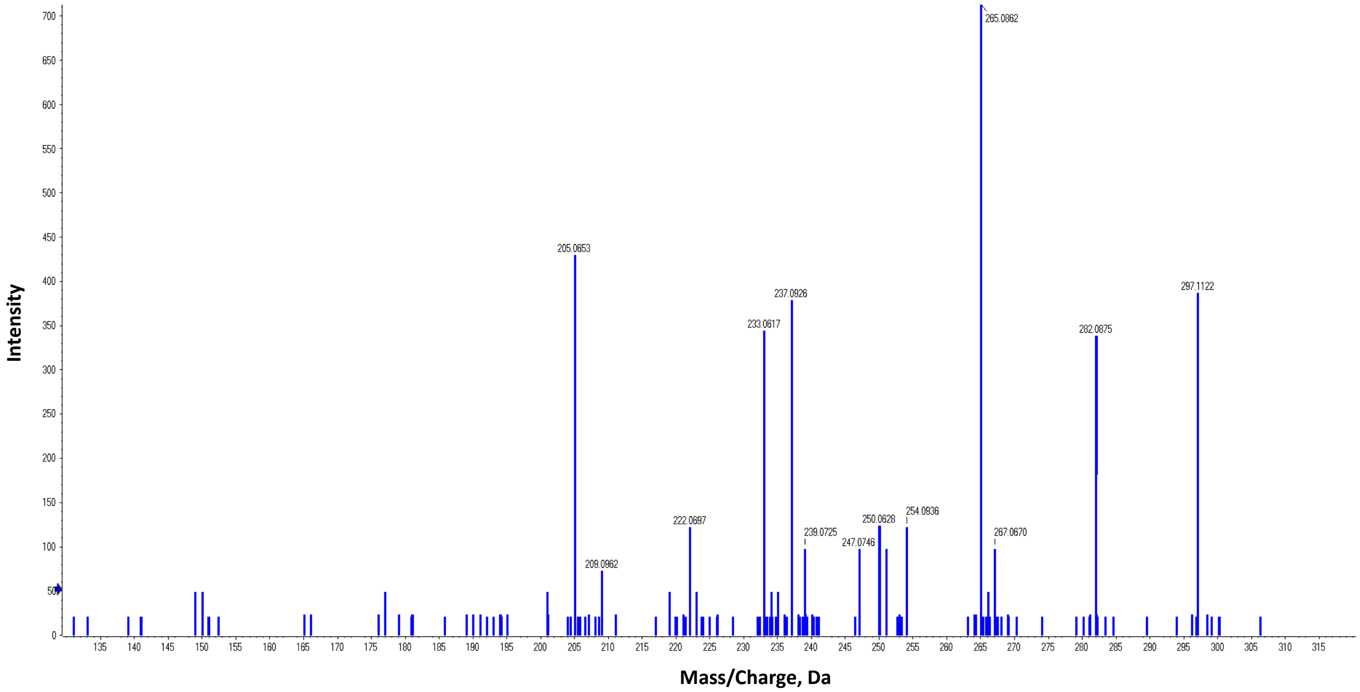

Figure S10_20: MS/MS spectrum of P20.

P21: $C_{20}H_{24}NO_4^+$, isomer of magnoflorine (formula see **P12**)

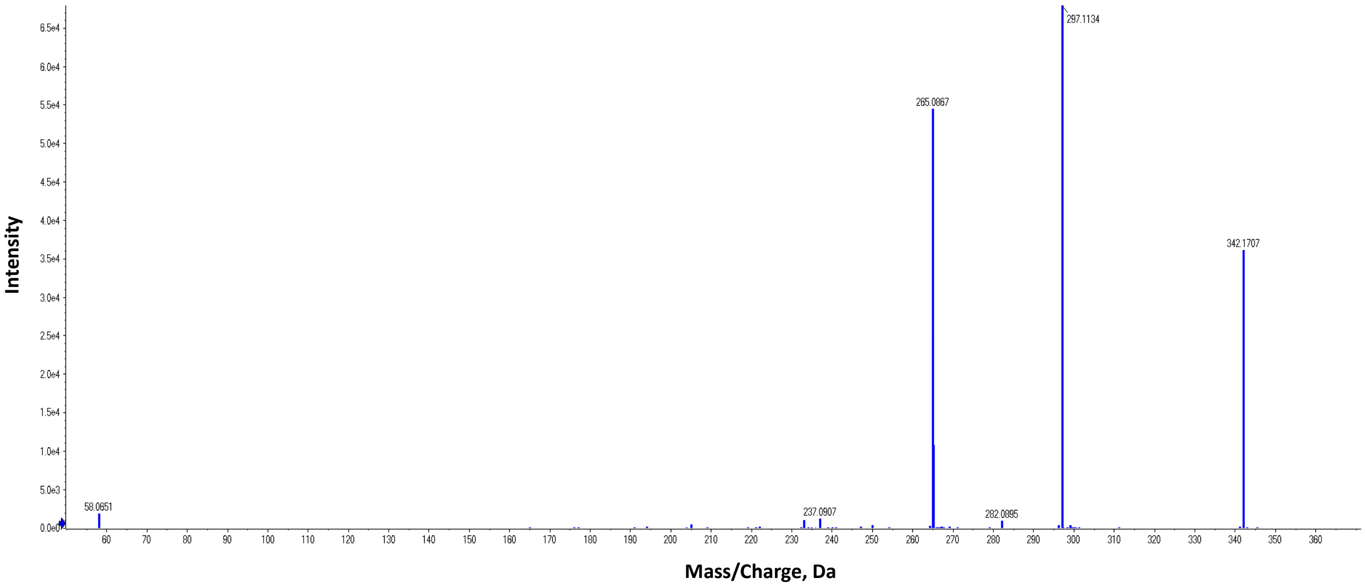

Figure S10_21: MS/MS spectrum of P21.

P22, Armepavine

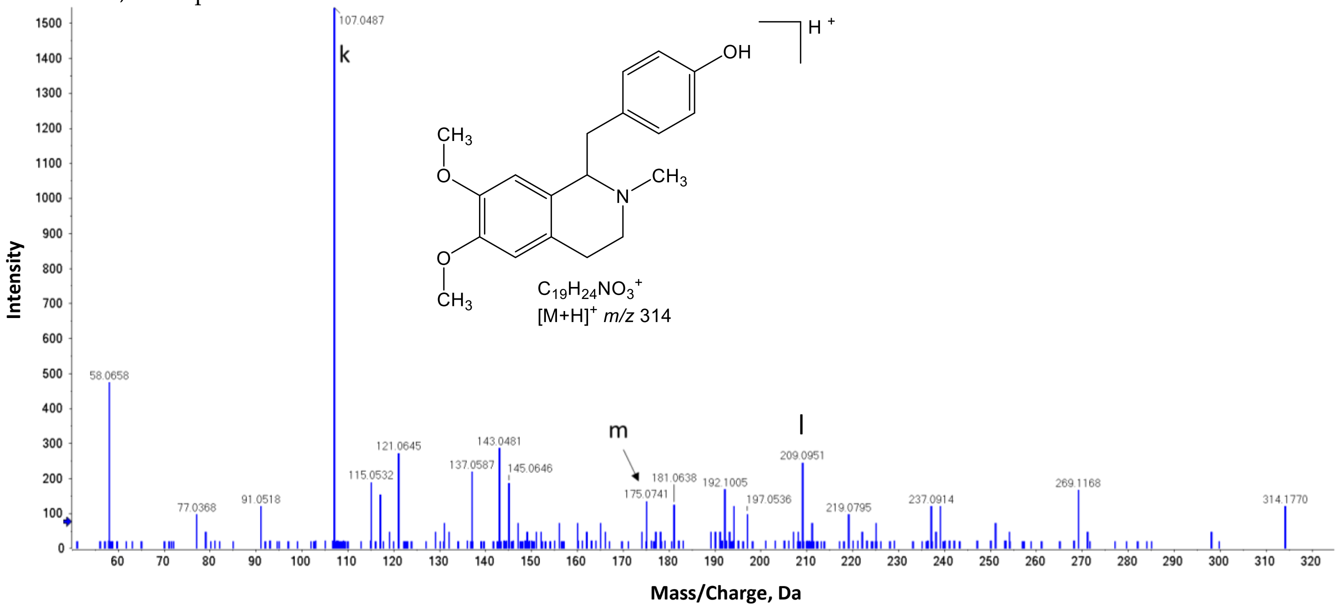

Figure S10_22: MS/MS spectrum of P22.

P23, Roseoside*

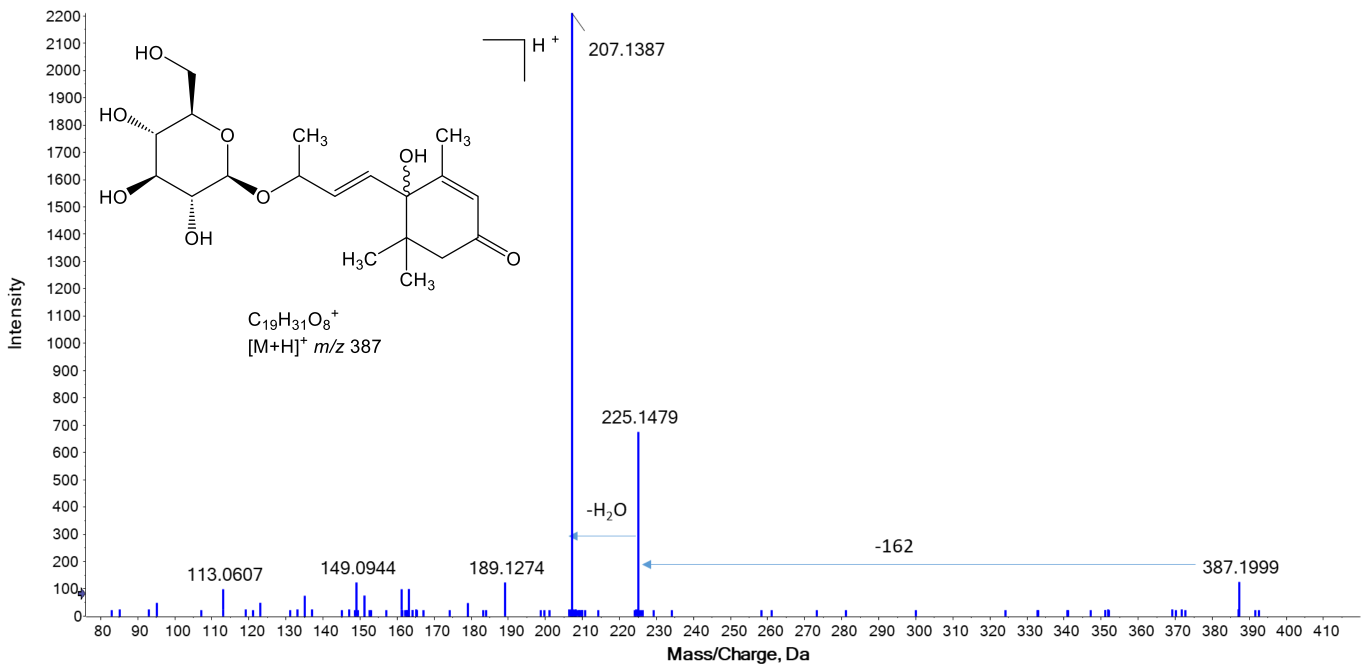

Figure S10_23: MS/MS spectrum of P23

P24, Pulchine

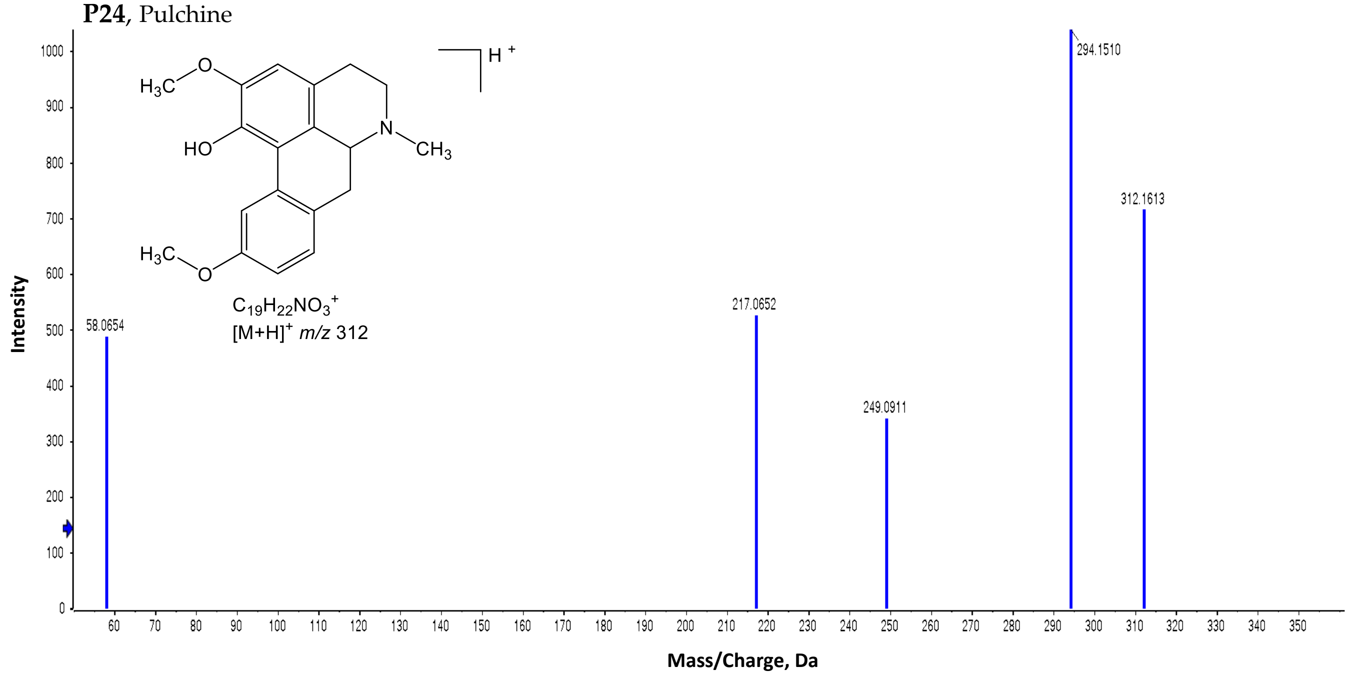

Figure S10_24: MS/MS spectrum of P24.

P25, Dehydroliriofenine

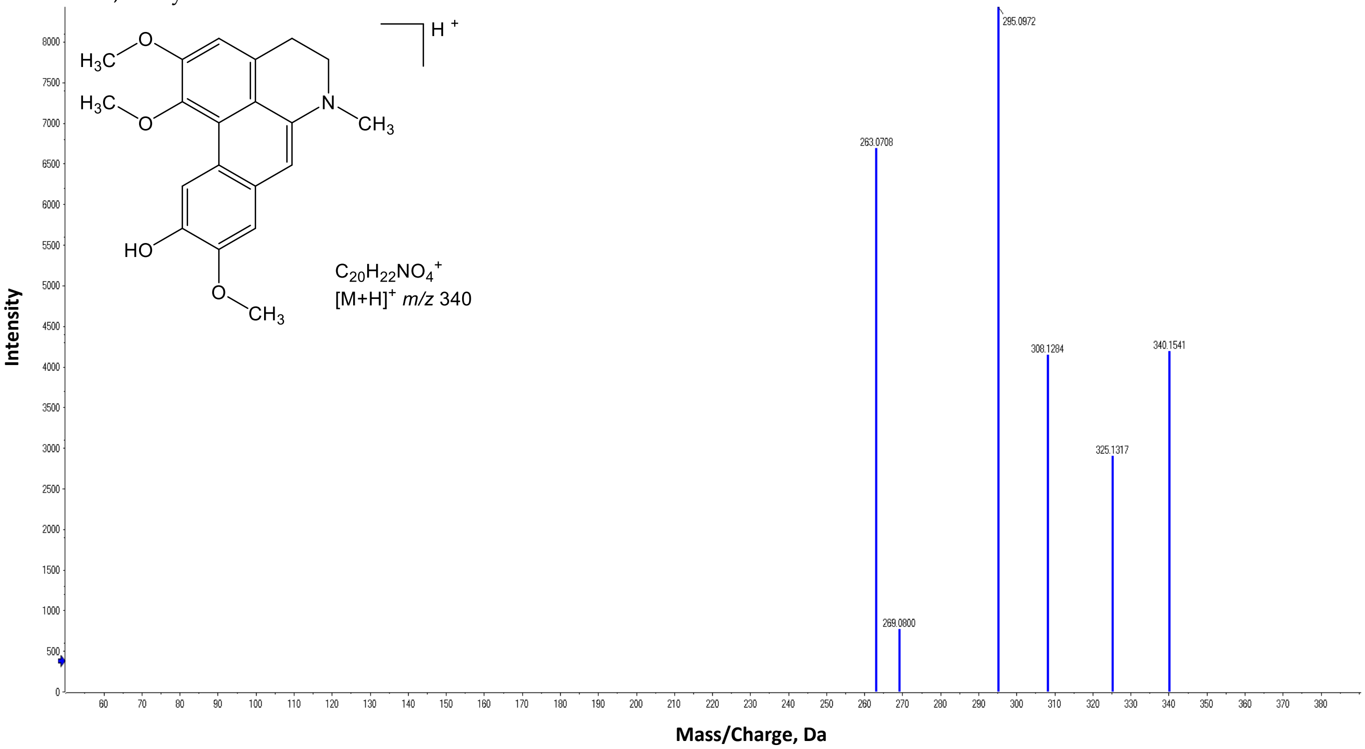

Figure S10_25: MS/MS spectrum of P25.

P26: $C_{17}H_{20}NO_3^+$, isomer of coclaurine (formula see **P9**)

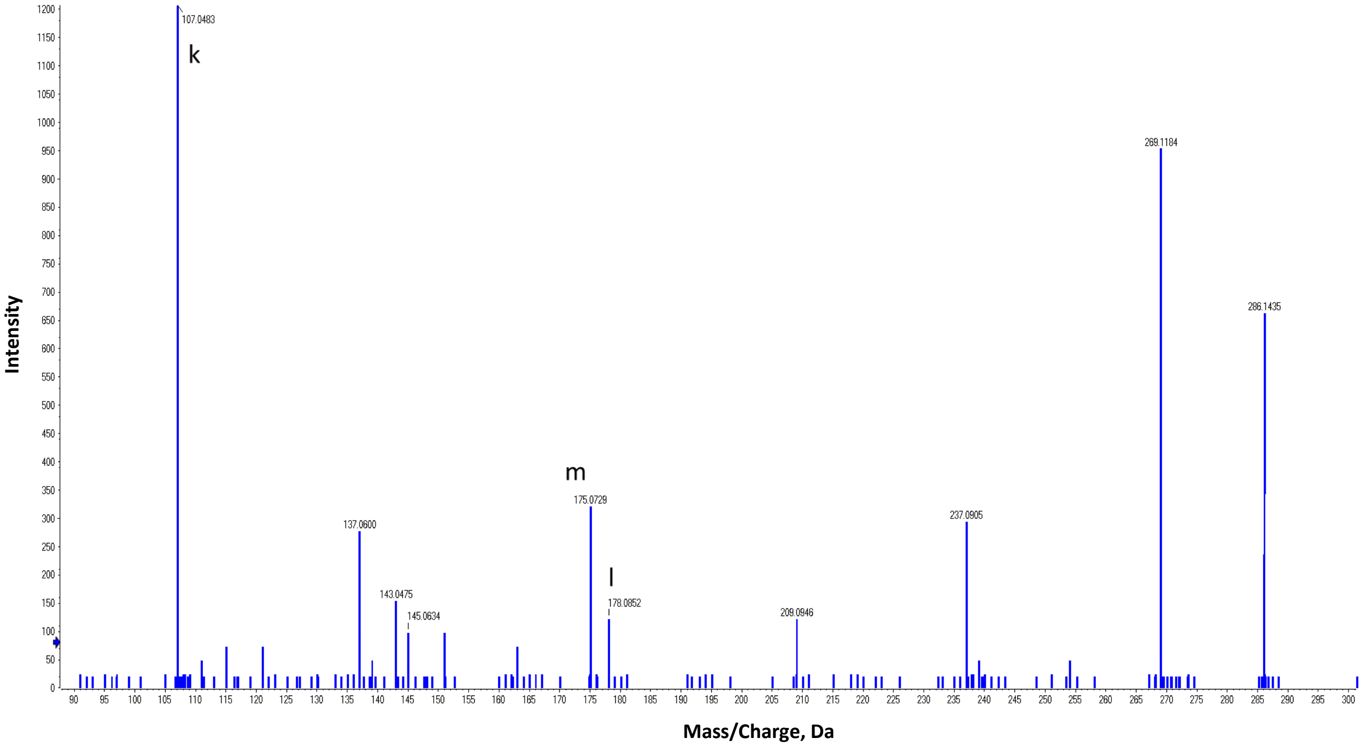

Figure S10_26: MS/MS spectrum of P26.

P27, Leucoxine

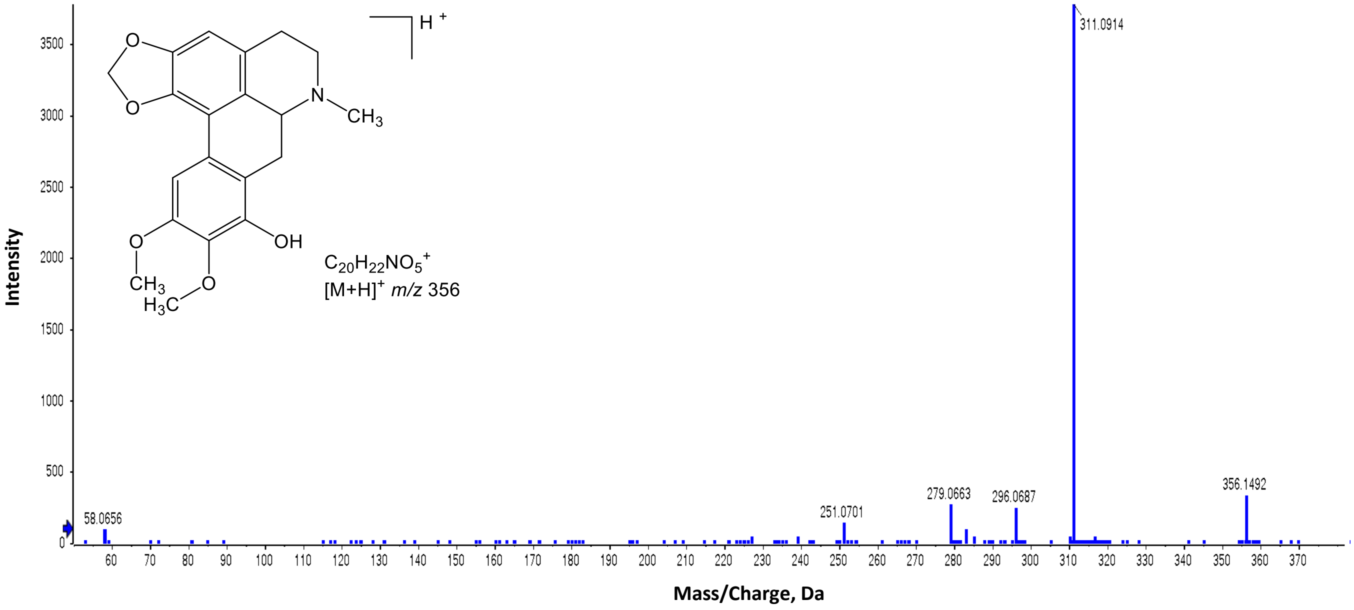

Figure S10_27: MS/MS spectrum of P27.

P28, Butyl-(3-Hydroxy-3-methylglutaryl) hexopyranoside

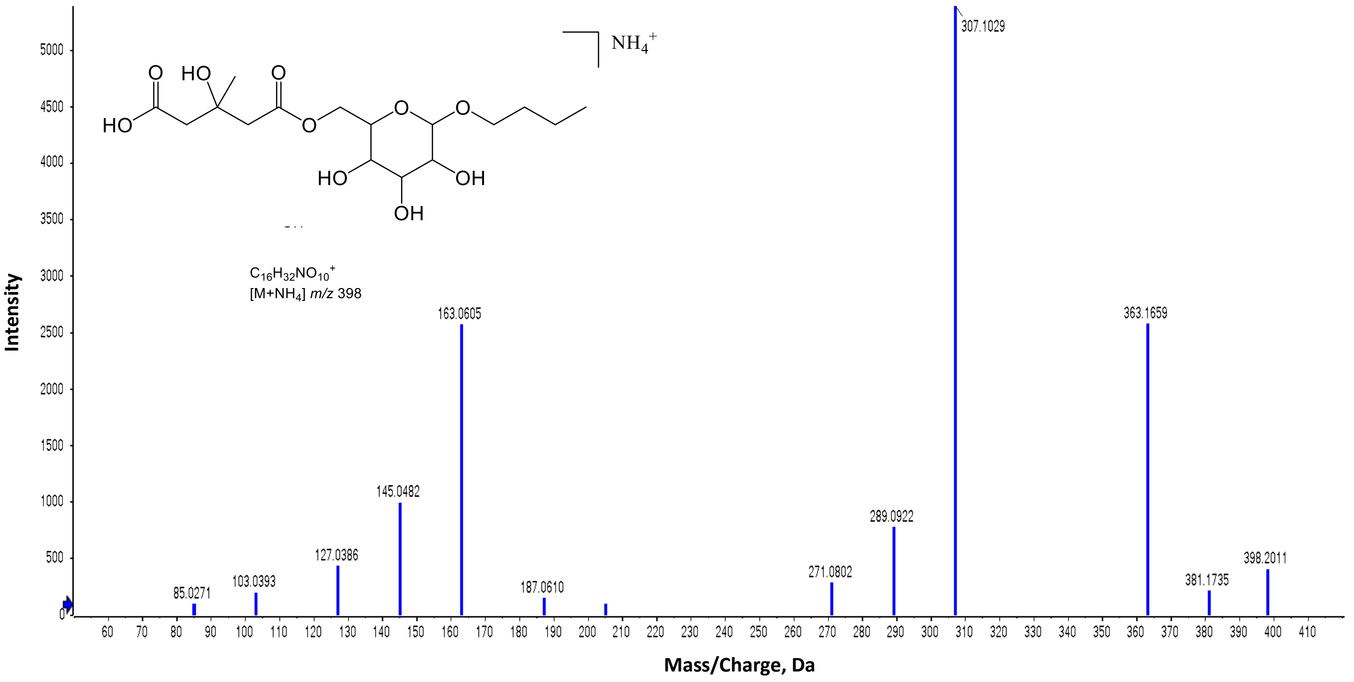

Figure S10_28: MS/MS spectrum of P28.

P29, Isoschaftoside

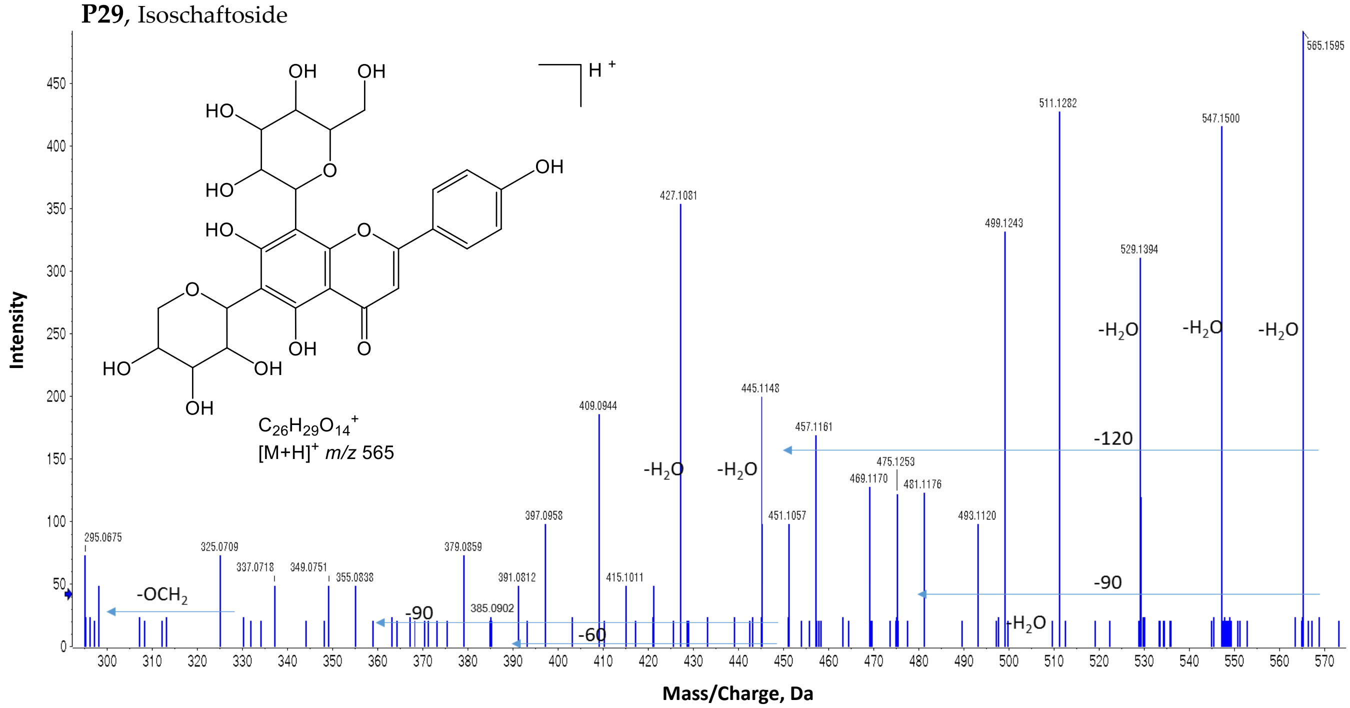

Figure S10_29: MS/MS spectrum of P29.

P30, Vitexin 2''-*O*-galactoside*

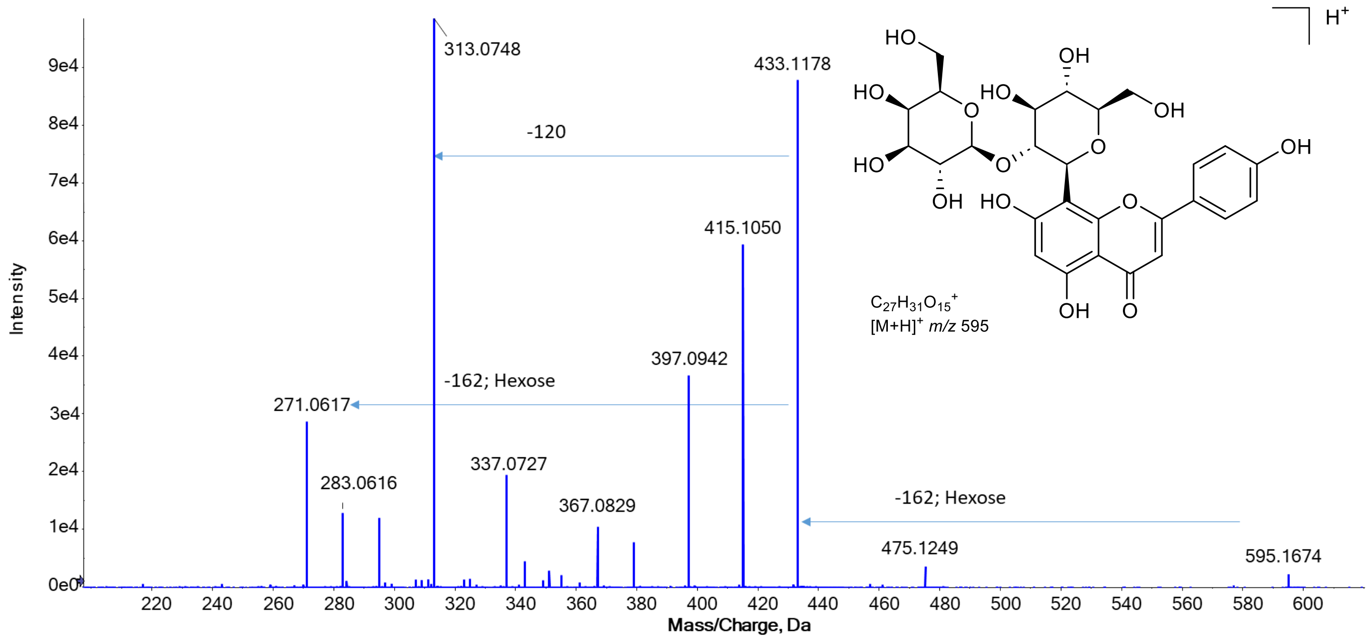

Figure S10_30: MS/MS spectrum of P30.

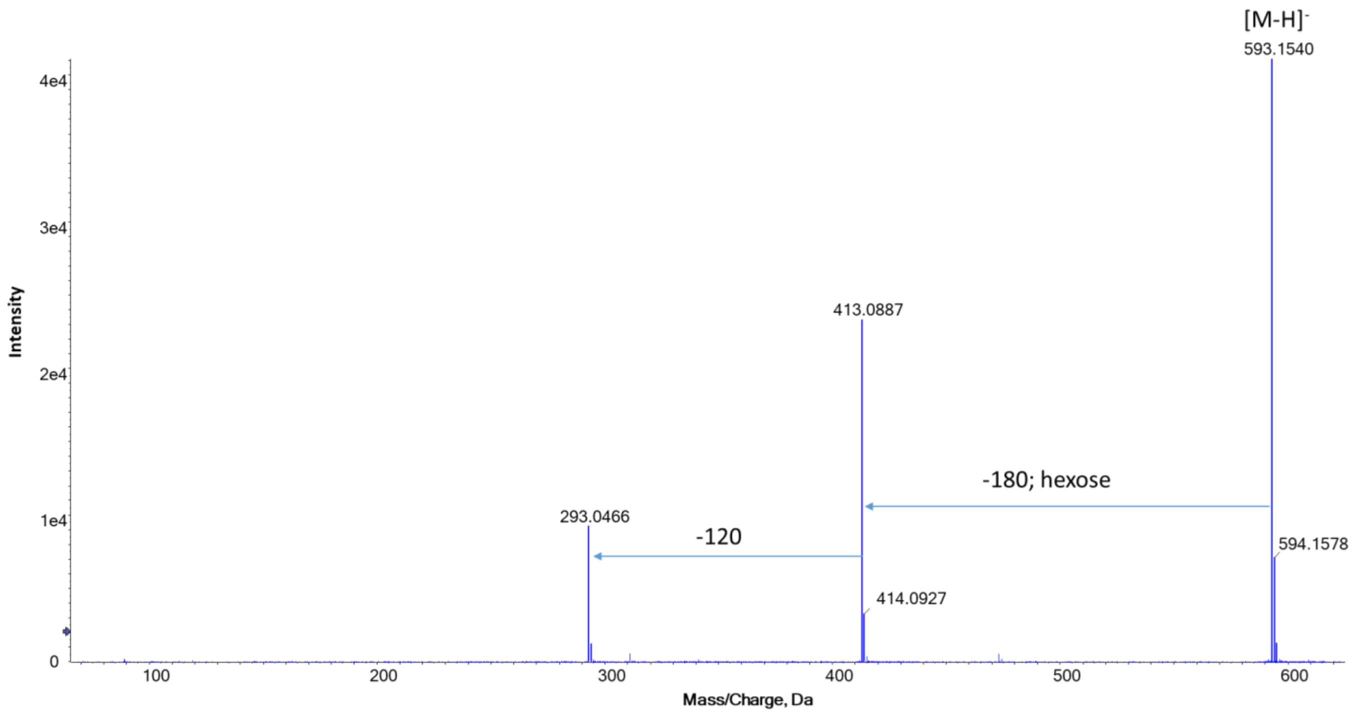

Figure S10_30b: MS/MS spectrum of P30 in negative ion mode.

P31, Vitexin 2''-*O*-rhamnoside

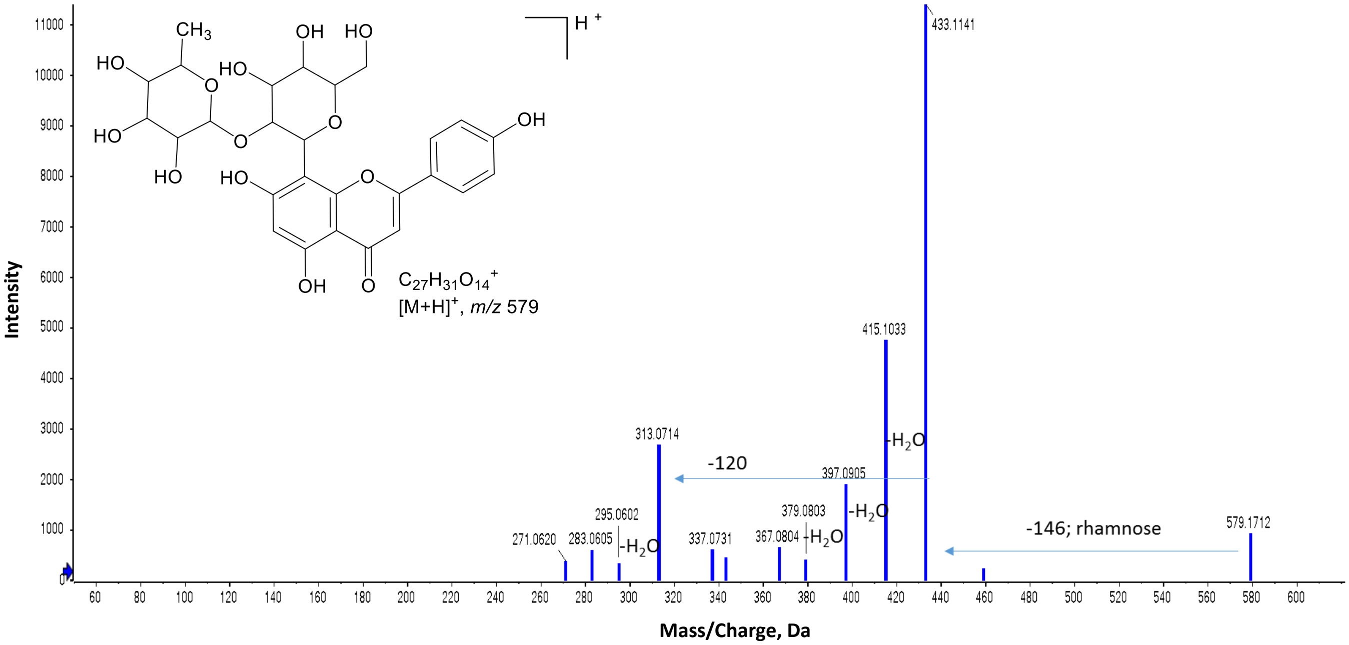

Figure S10_31: MS/MS spectrum of P31.

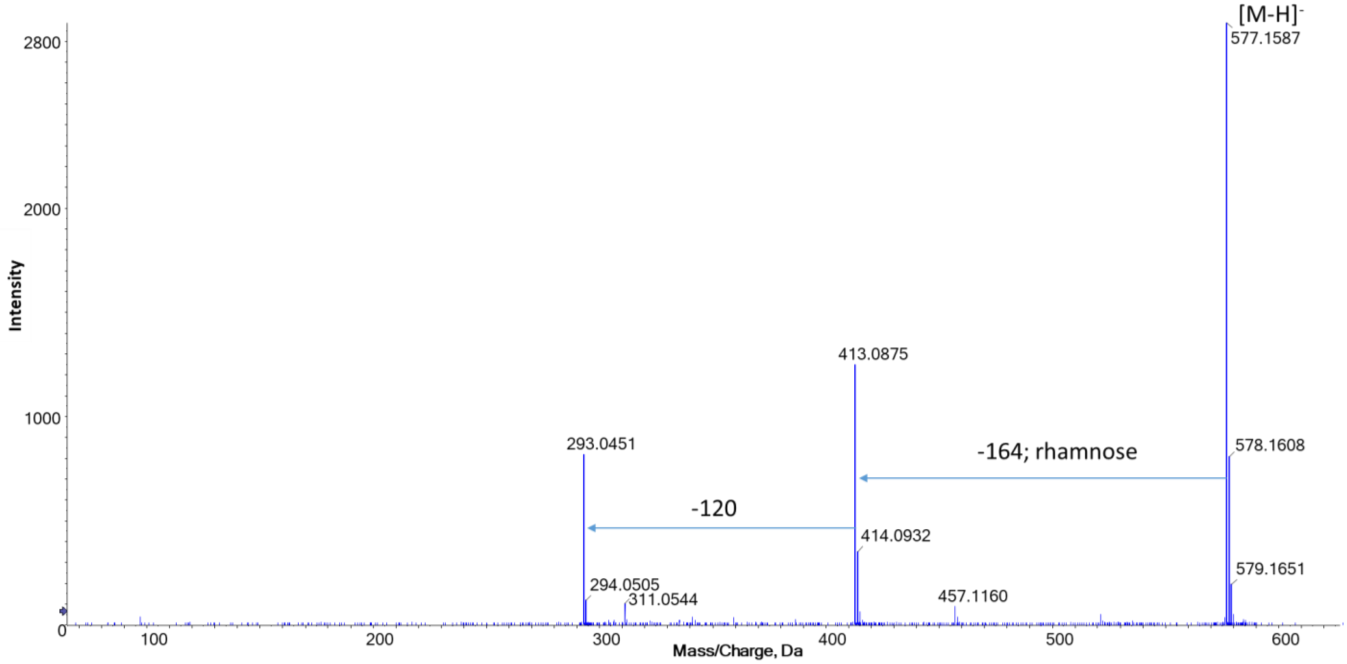

Figure S10_31b: MS/MS spectrum of P31 in negative ion mode.

P32, Vitexin-*O*-pentoside

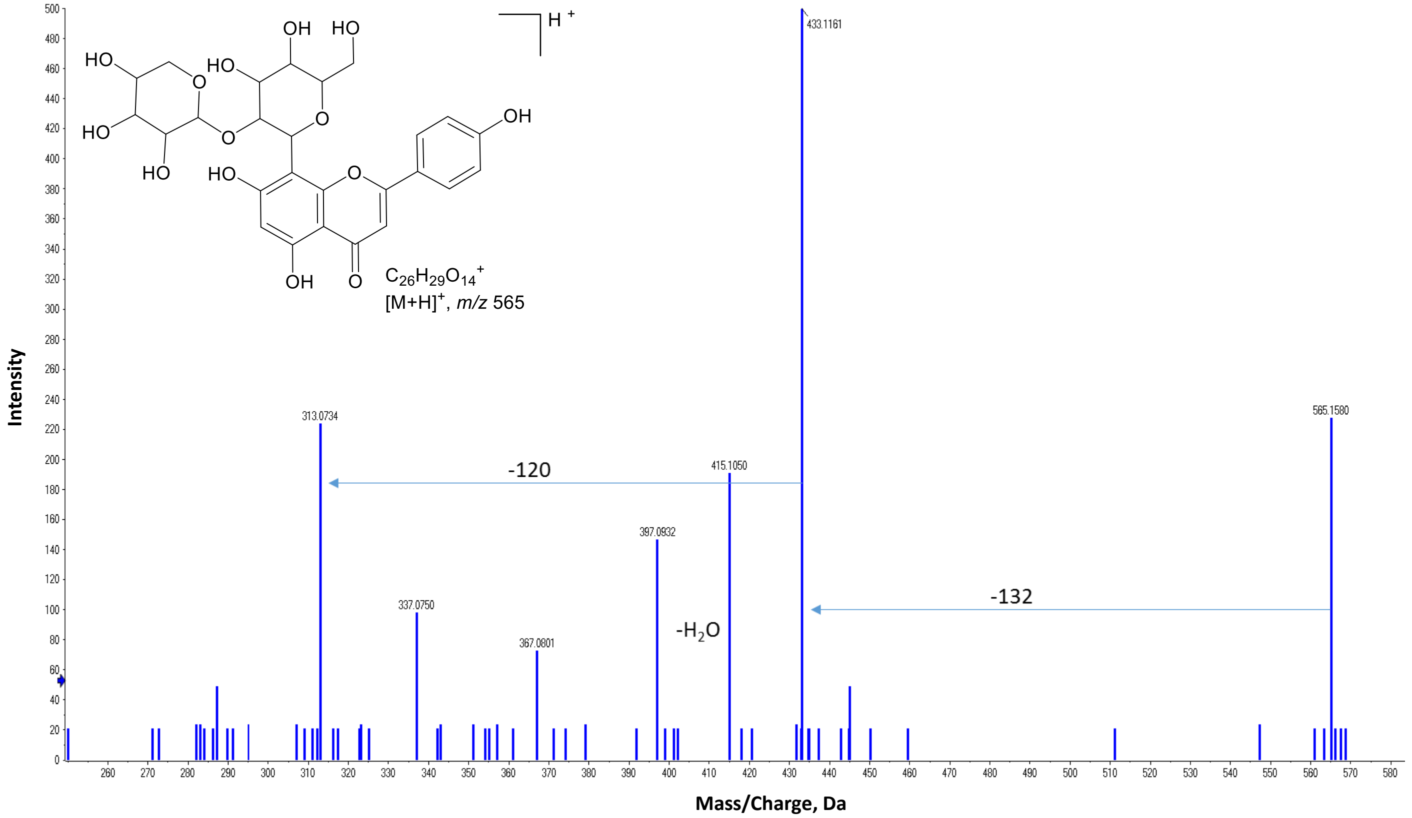

Figure S10_32: MS/MS spectrum of P32.

P33, Coniferinoside

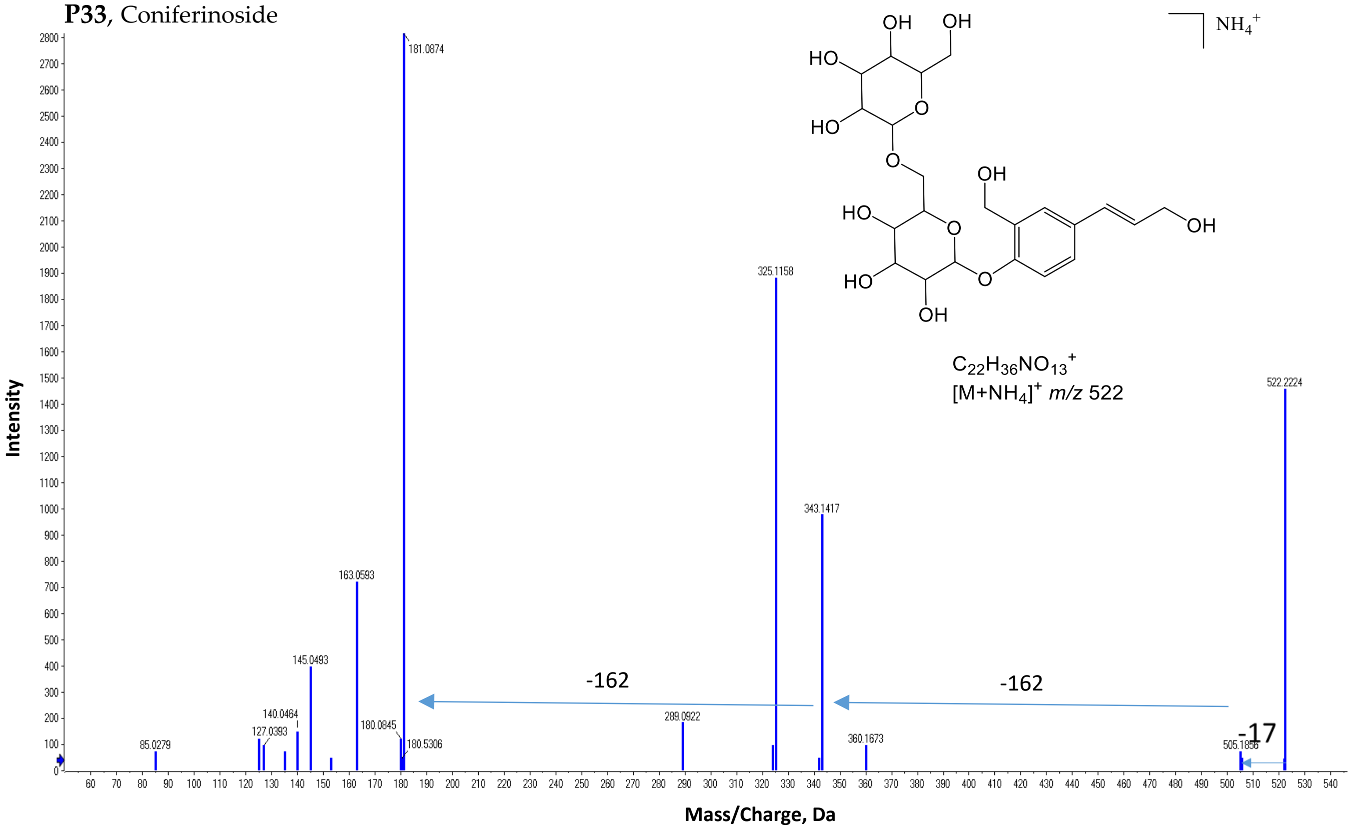

Figure S10_33: MS/MS spectrum of P33.

P34, Vitexin

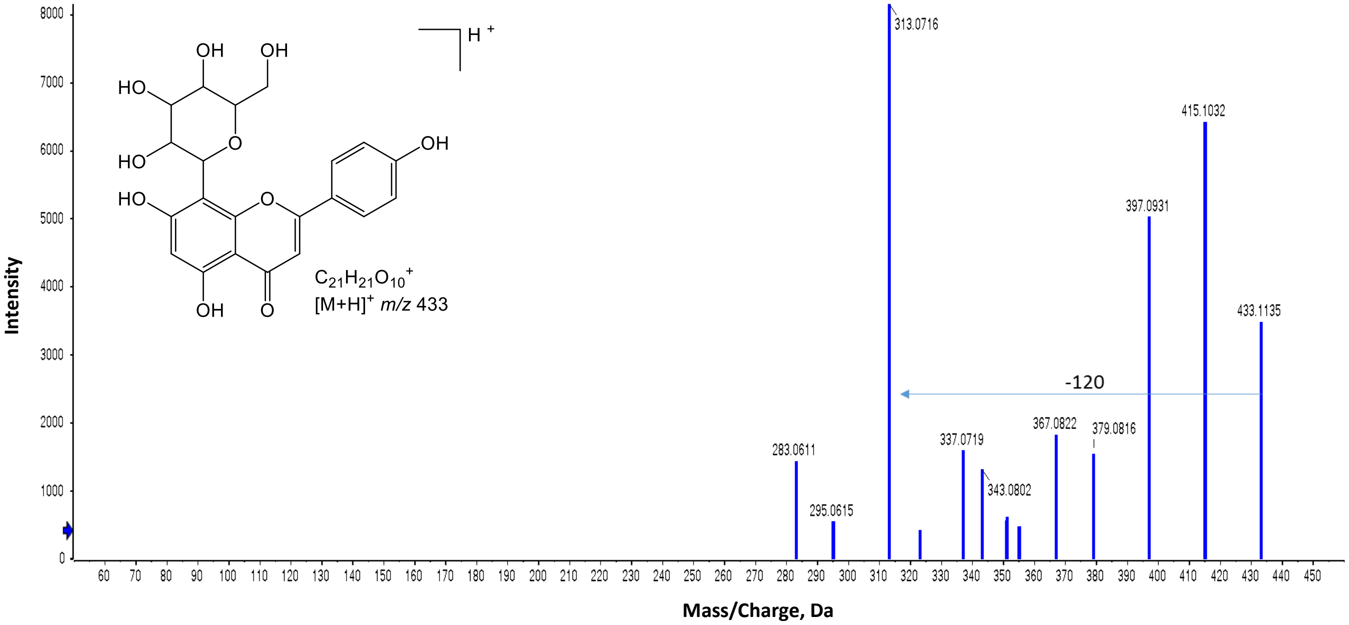

Figure S10_34: MS/MS spectrum of P34.

P35, 8,8'-Bisdihydrosiringenin hexoside

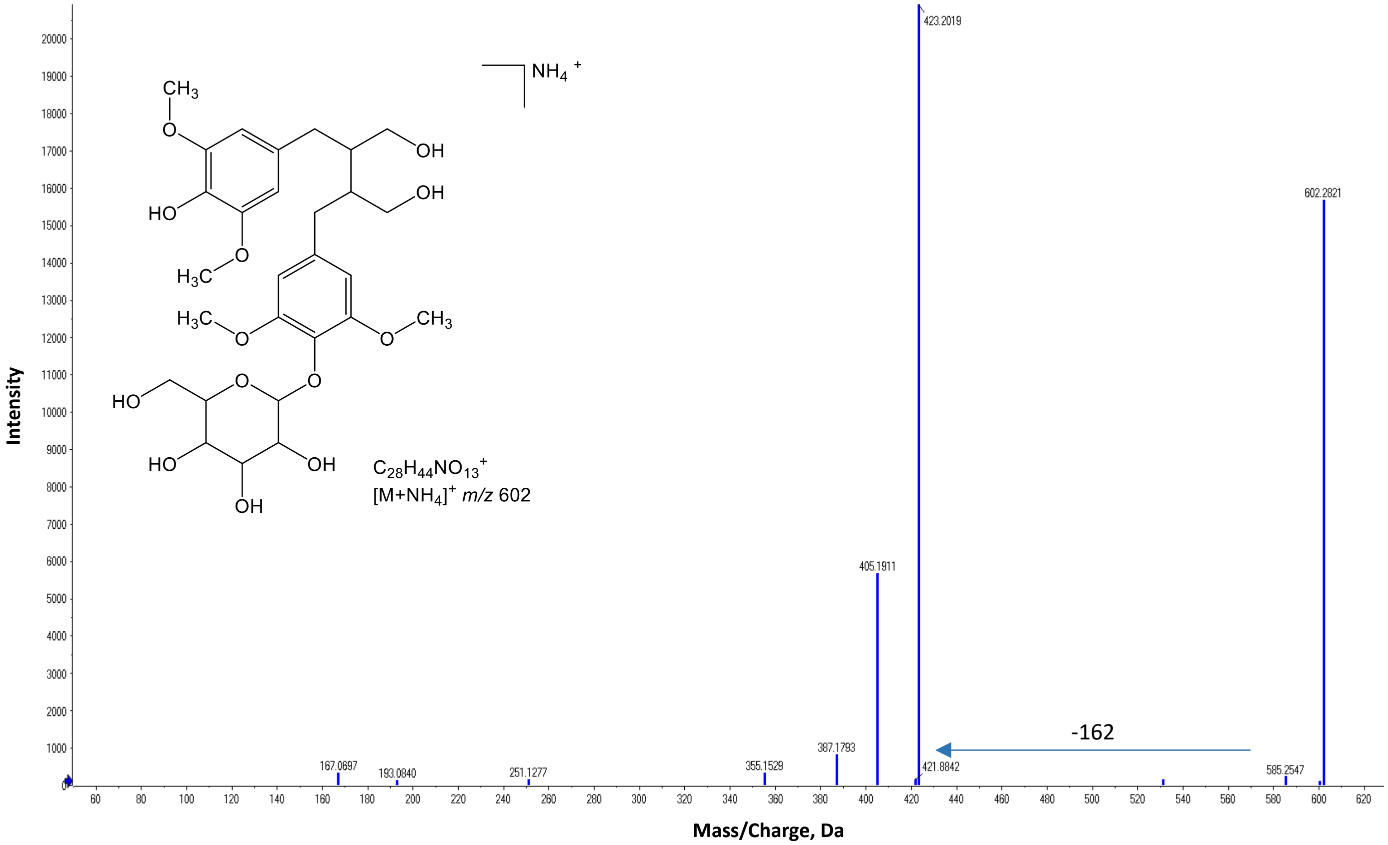

Figure S10_35: MS/MS spectrum of P35.

P36, Vitexin 4''-(3-hydroxy-3-methylglutaroyl)-2''-*O*-β-D-glucopyranoside*

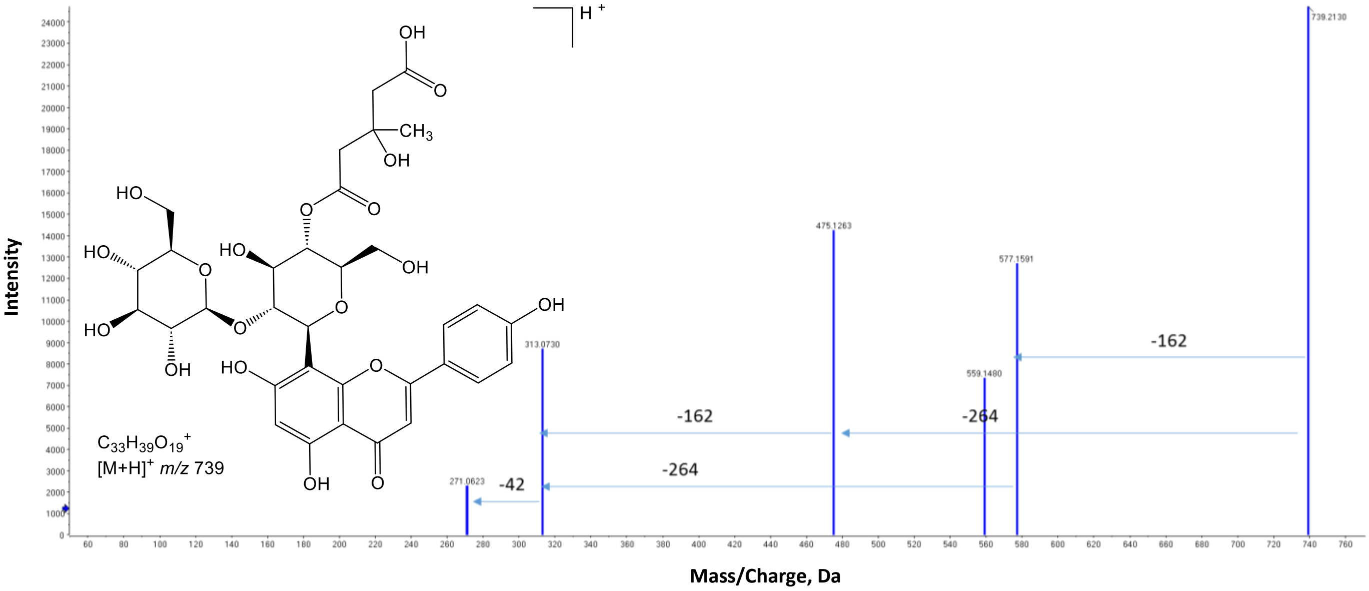

Figure S10_36: MS/MS spectrum of P36.

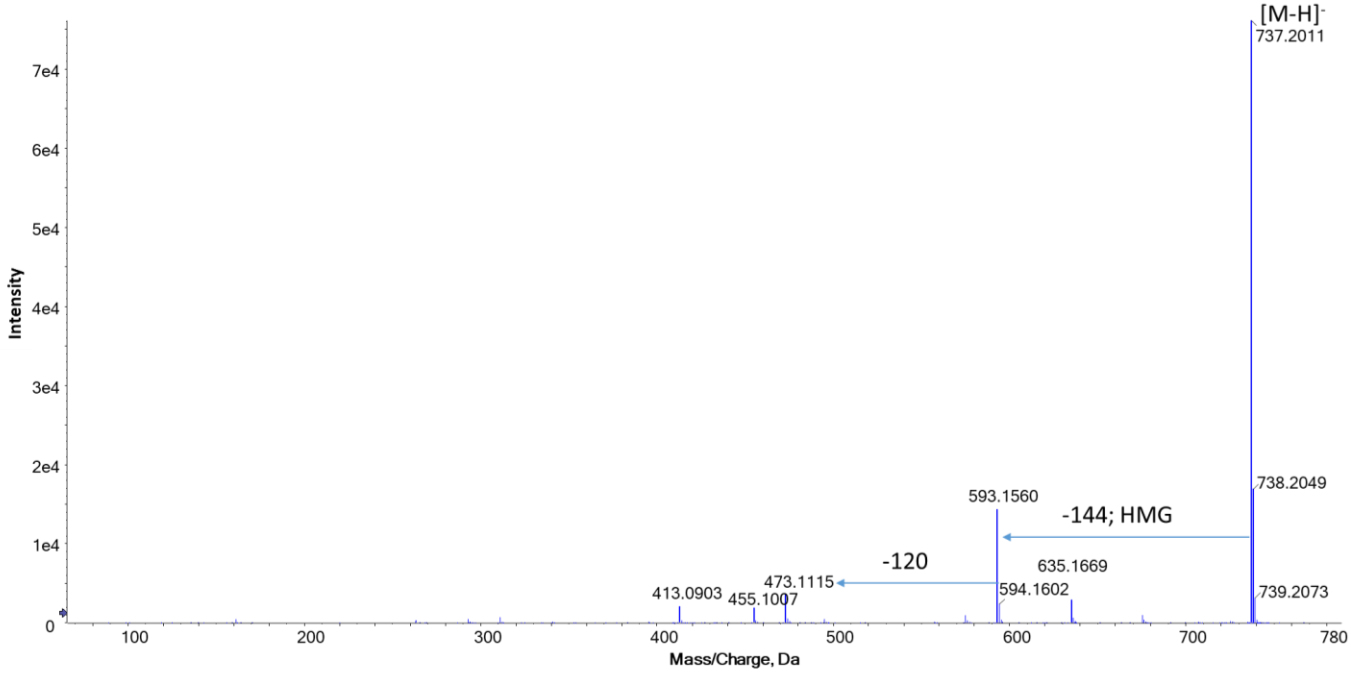

Figure S10_36b: MS/MS spectrum of P36 in negative ion mode.

P37, Apigenin 8-*C*-pentopyranosyl-2′′-*O*-hexoside

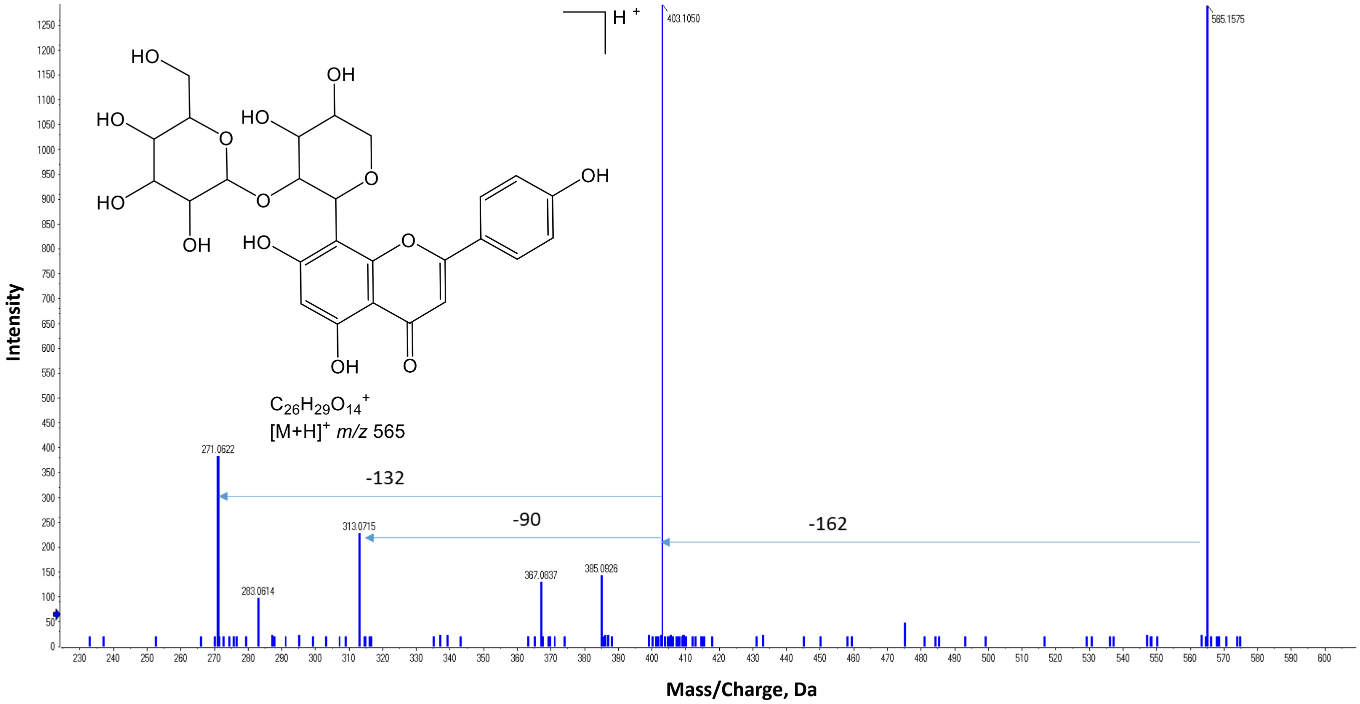

Figure S10_37: MS/MS spectrum of P37.

P38, Vitexin 4''-(3-hydroxy-3-methylglutaroyl)-2''-*O*-rhamnoside

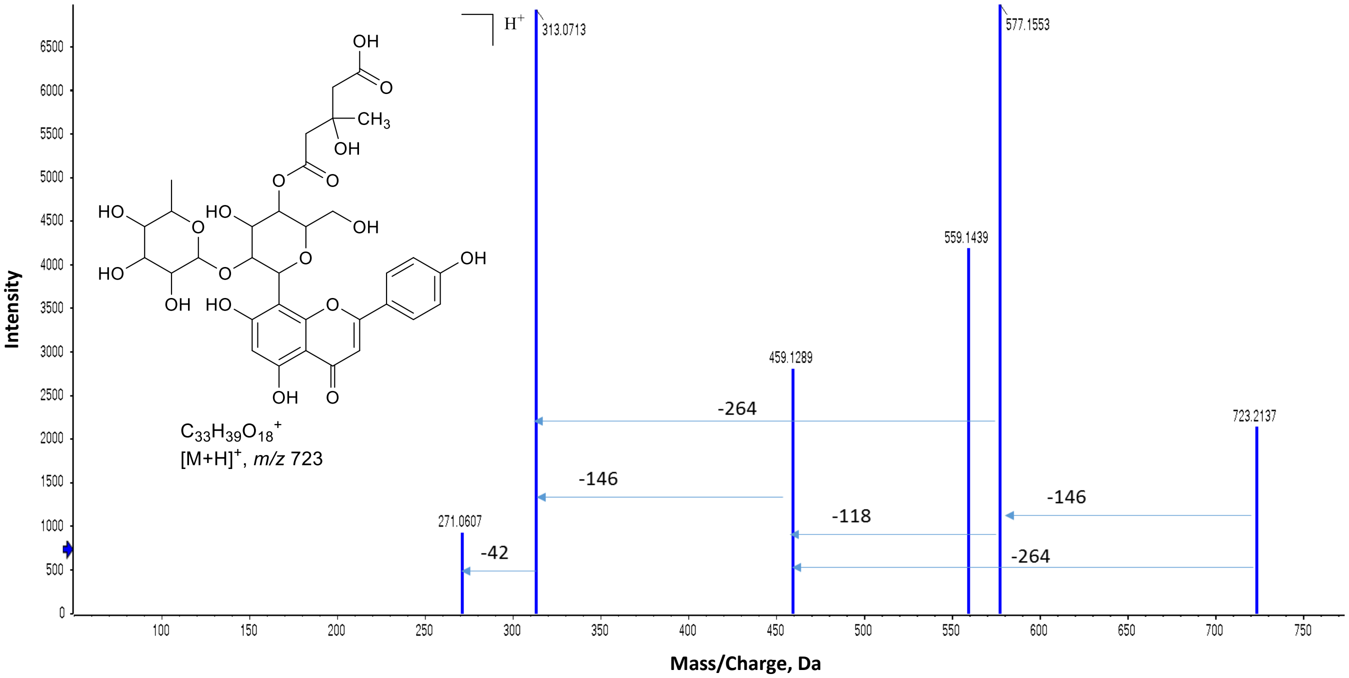

Figure S10_38: MS/MS spectrum of P38.

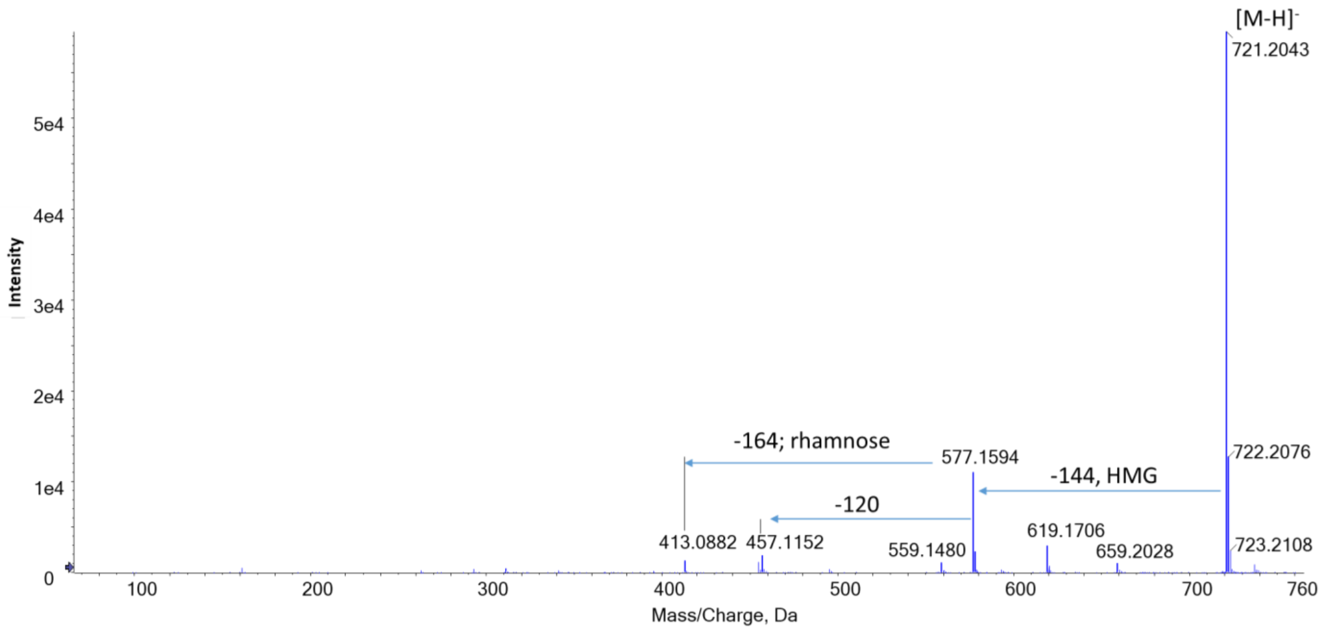

Figure S10_38b: MS/MS spectrum of P38 in negative ion mode.

P39, Vitexin 4''-(3'''-hydroxy-3'''-methyl-glutarate)

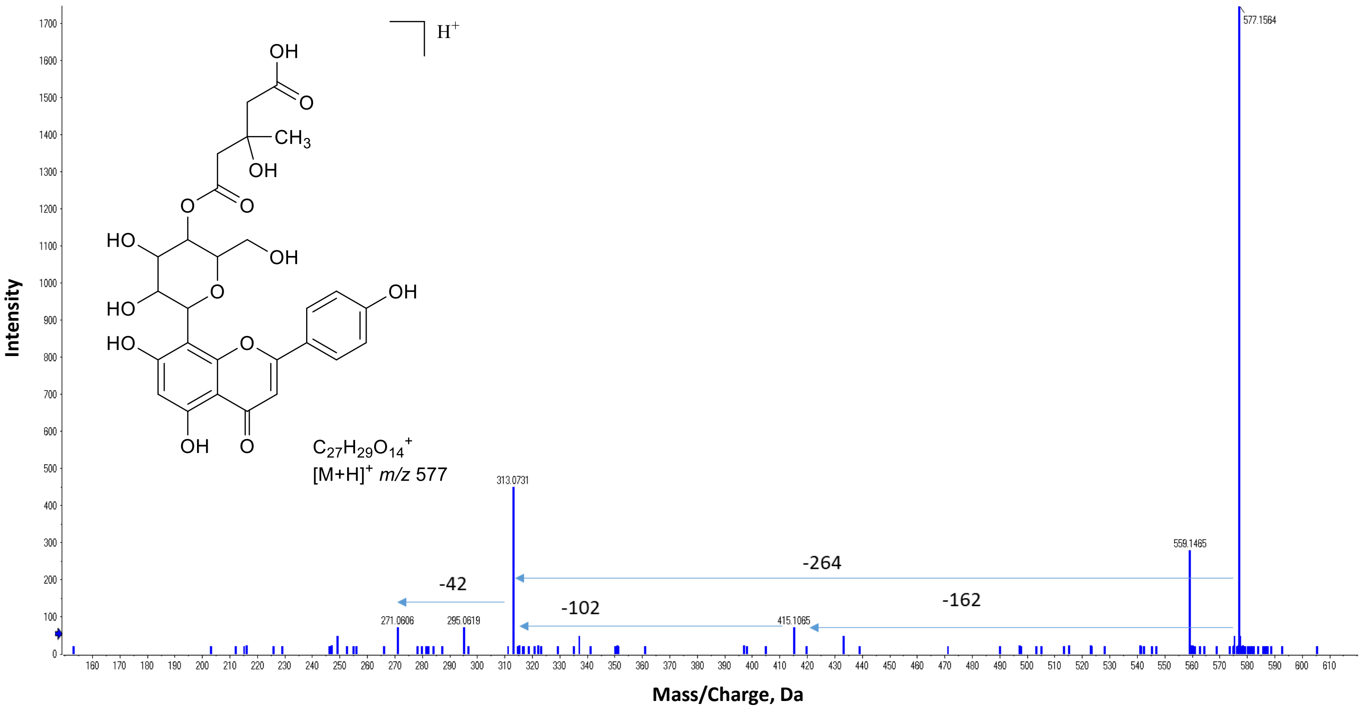

Figure S10_39: MS/MS spectrum of P39.

P40, Loliolide*

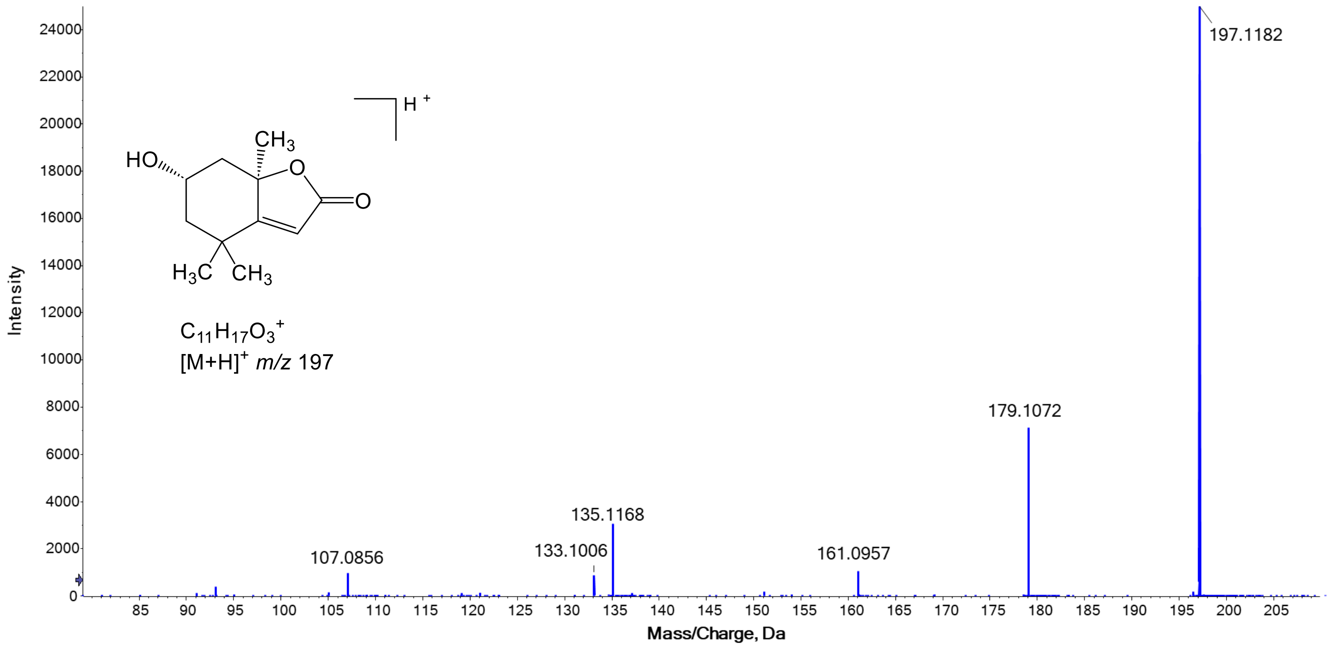

Figure S10_40: MS/MS spectrum of P40.

P41, Atherospermine

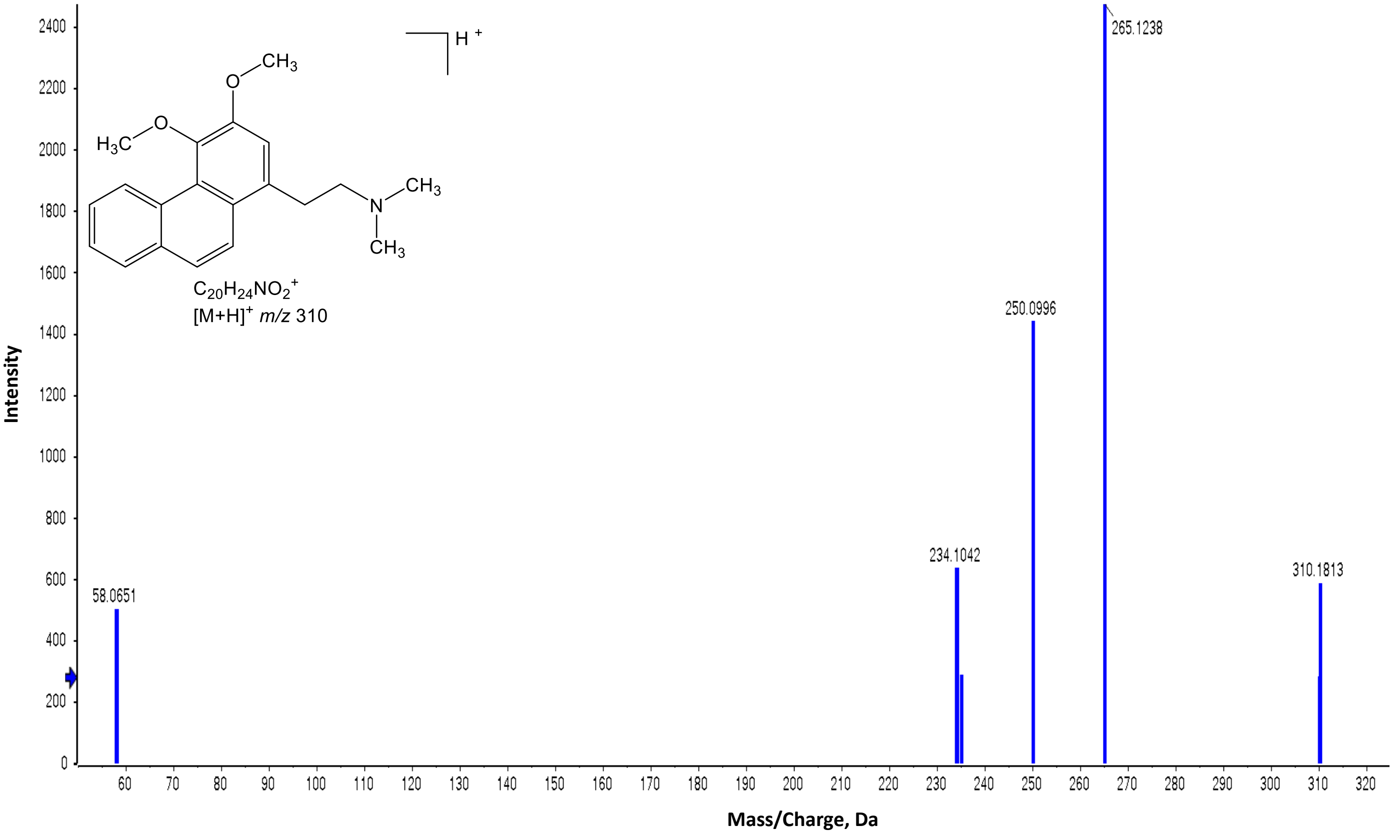

Figure S10_41: MS/MS spectrum of P41.

P42, Indole-3-carboxaldehyde*

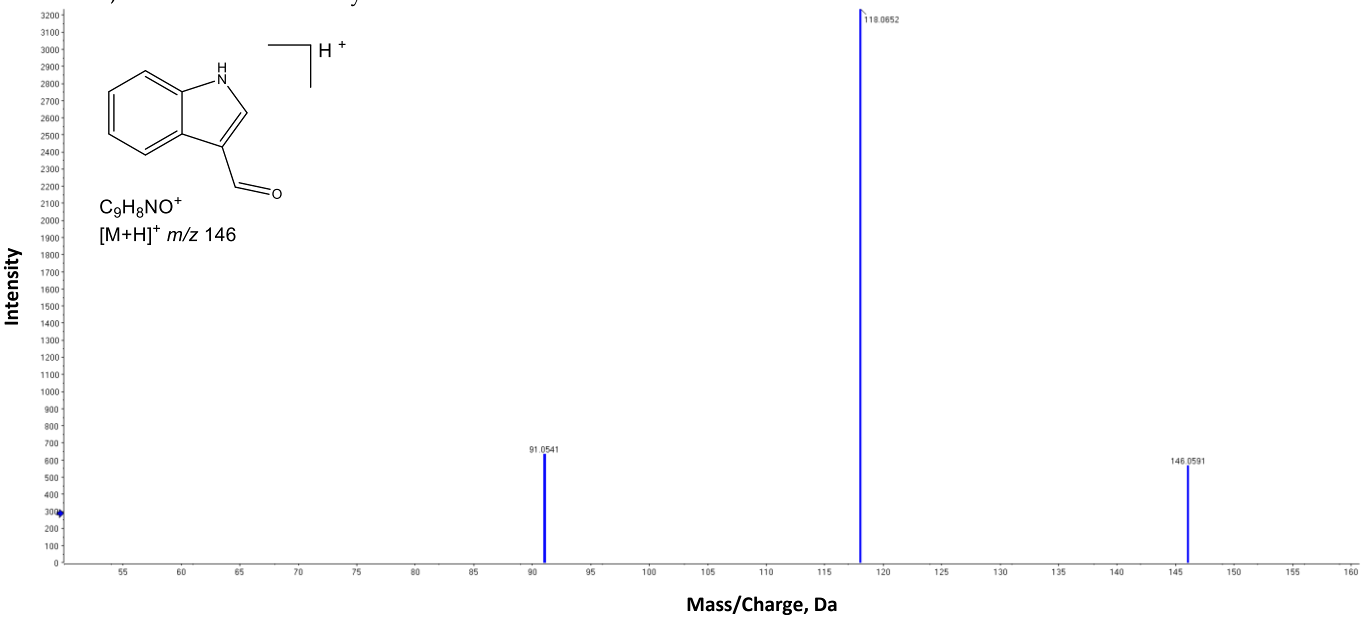

Figure S10_42: MS/MS spectrum of P42.

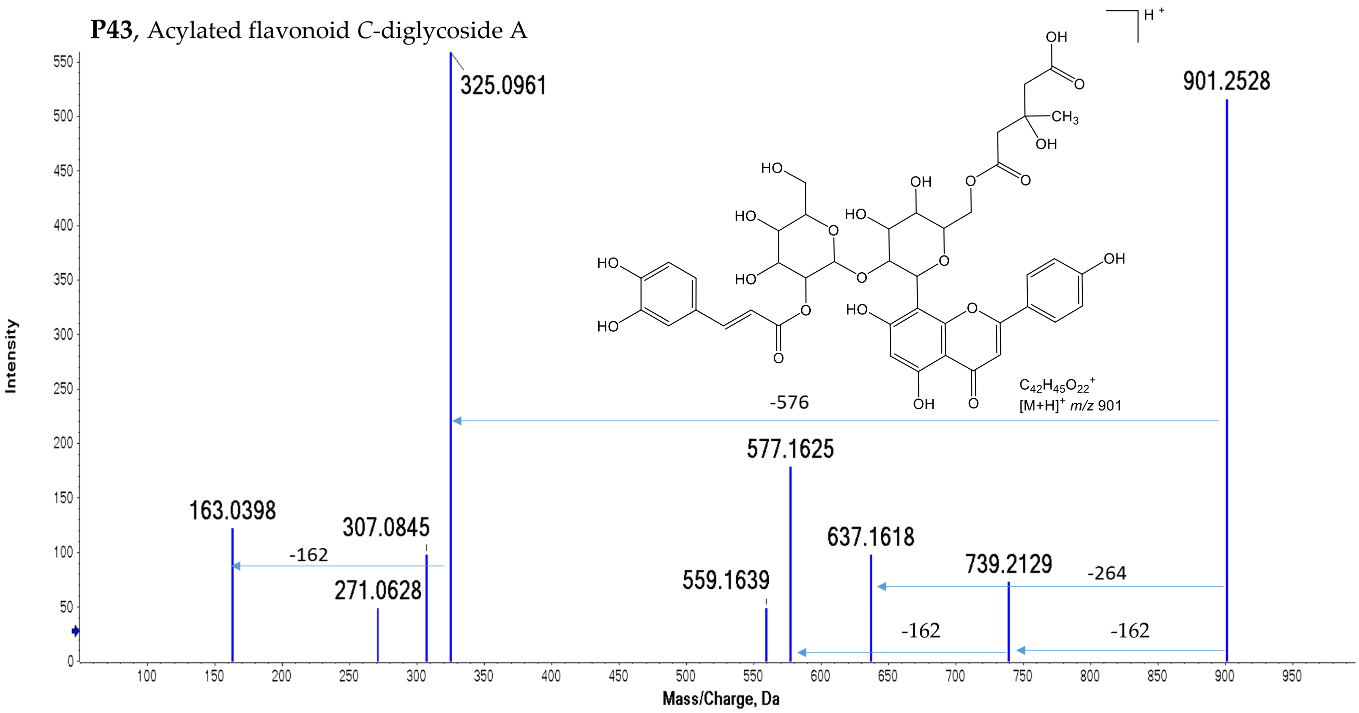

Figure S10_43: MS/MS spectrum of P43.

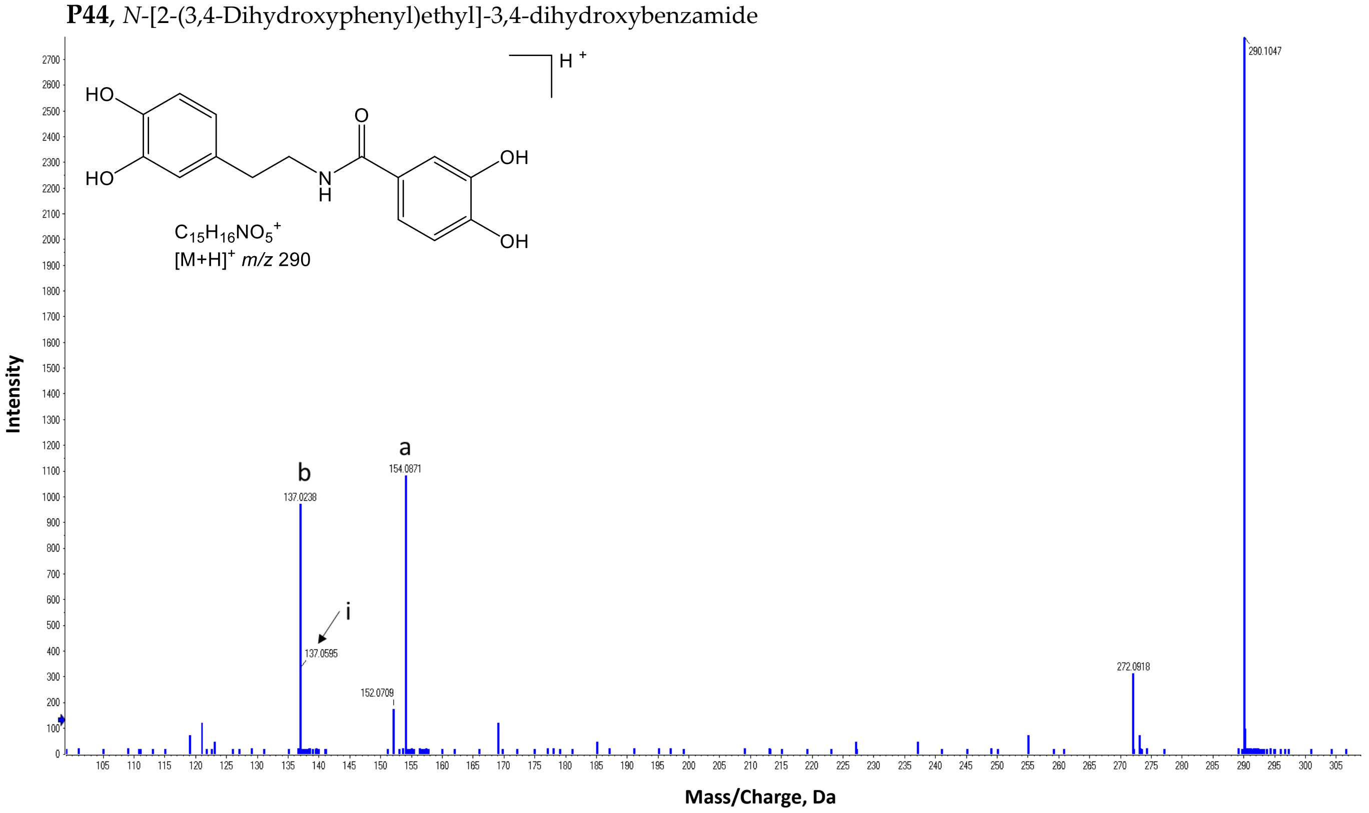

Figure S10_44: MS/MS spectrum of P44.

P45, Undatuside C

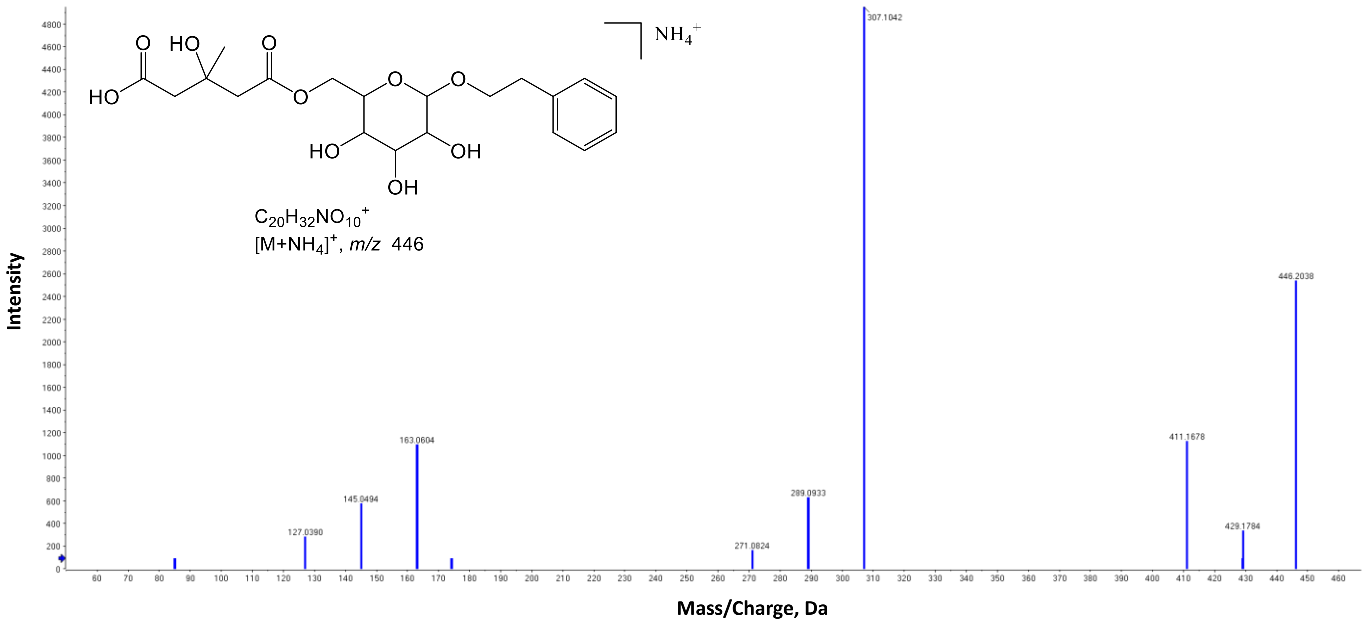

Figure S10_45: MS/MS spectrum of P45.

P46, 1-(2-Hydroxypyrrolidin-1-yl)-3-phenylpropan-1-one

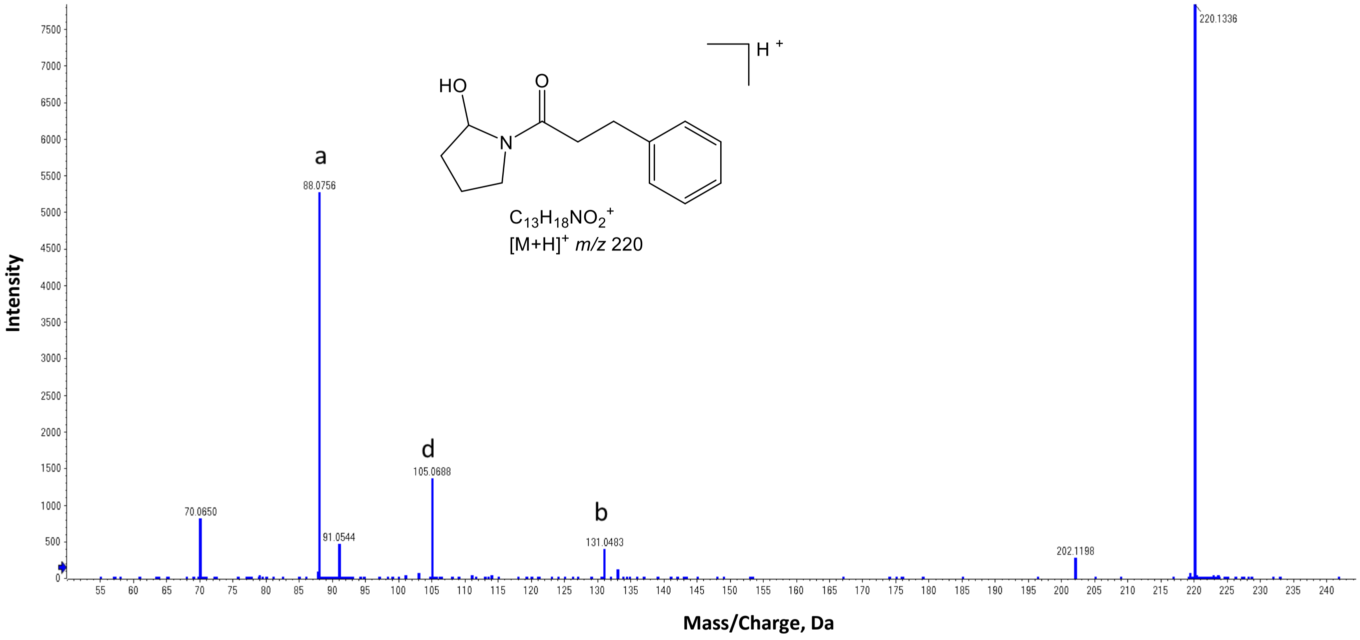

Figure S10_46: MS/MS spectrum of P46.

P47, Acylated flavonoid C-diglycoside B

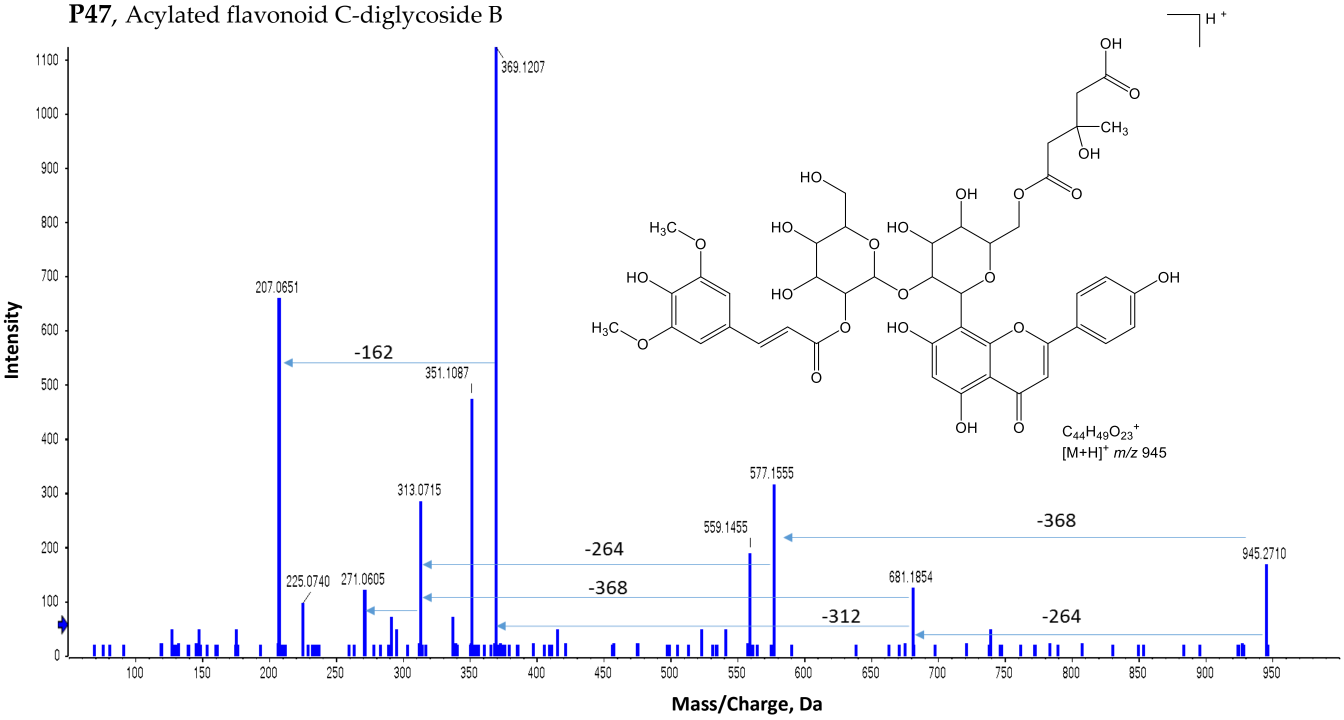

Figure S10_47: MS/MS spectrum of P47.

P48, Acylated flavonoid *C*-diglycoside C

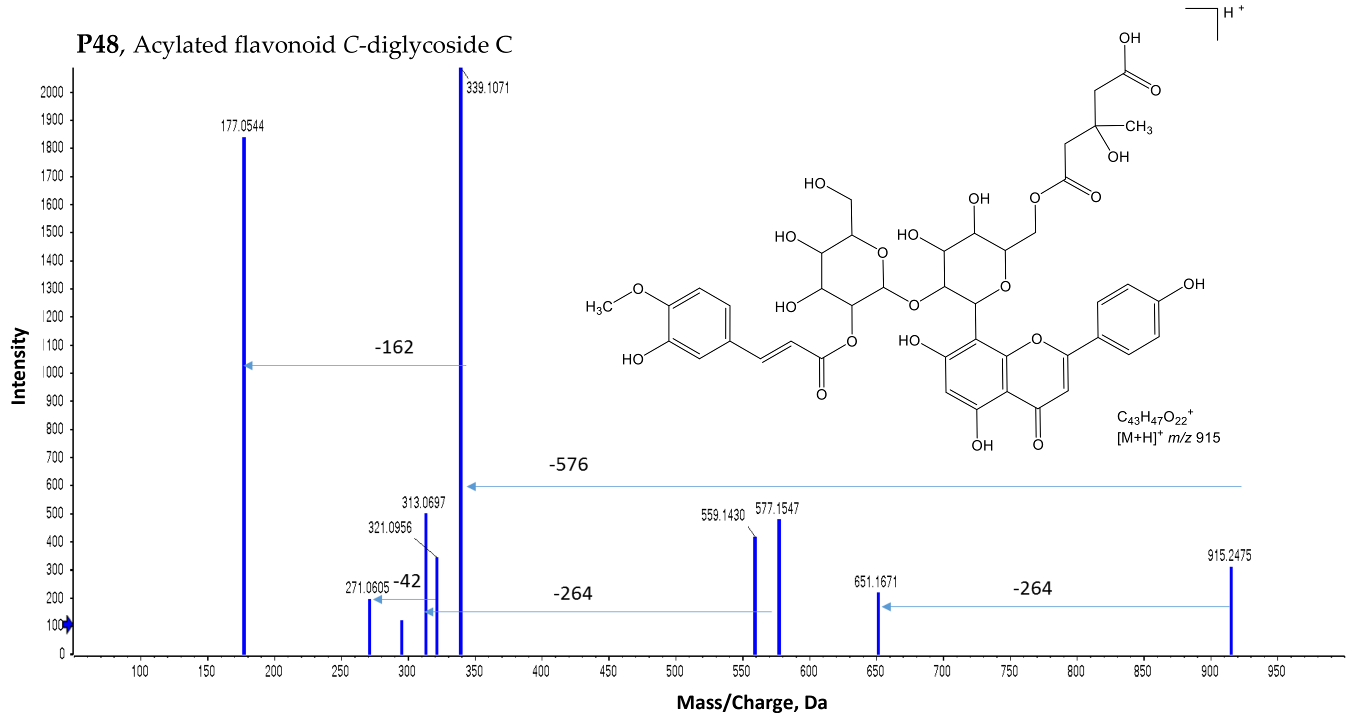

Figure S10_48: MS/MS spectrum of P48.

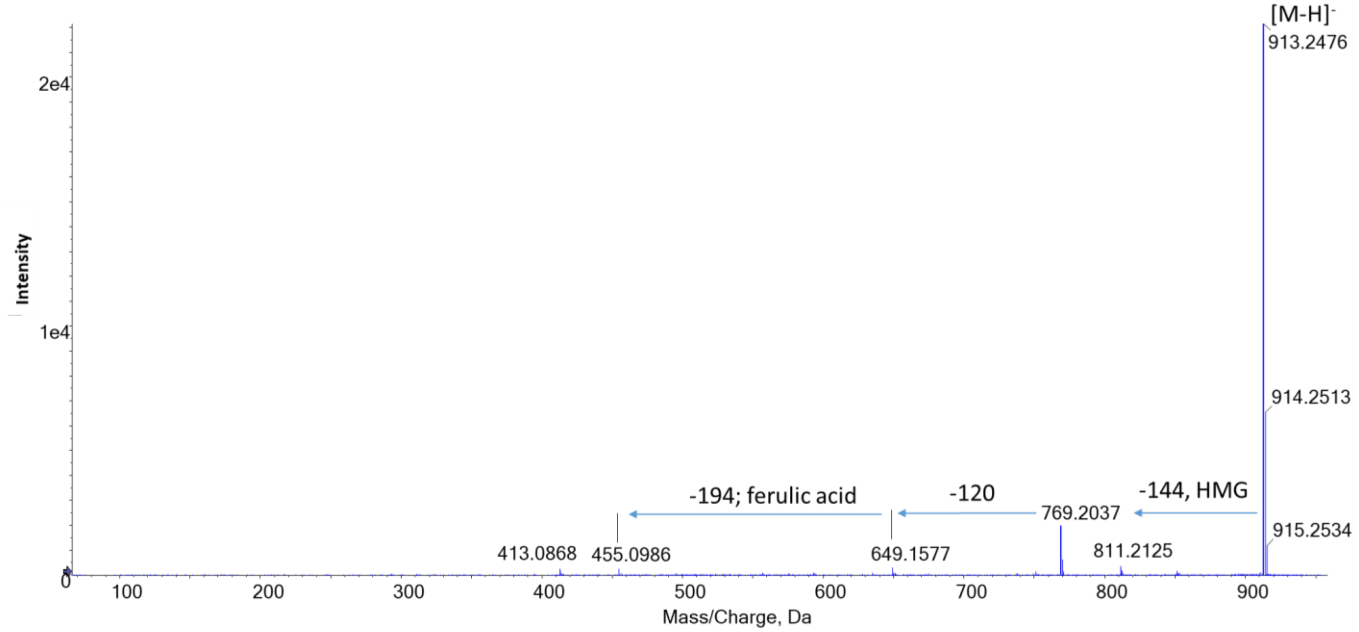

Figure S10_48b: MS/MS spectrum of P48 in negative ion mode.

P49, Scoparone*

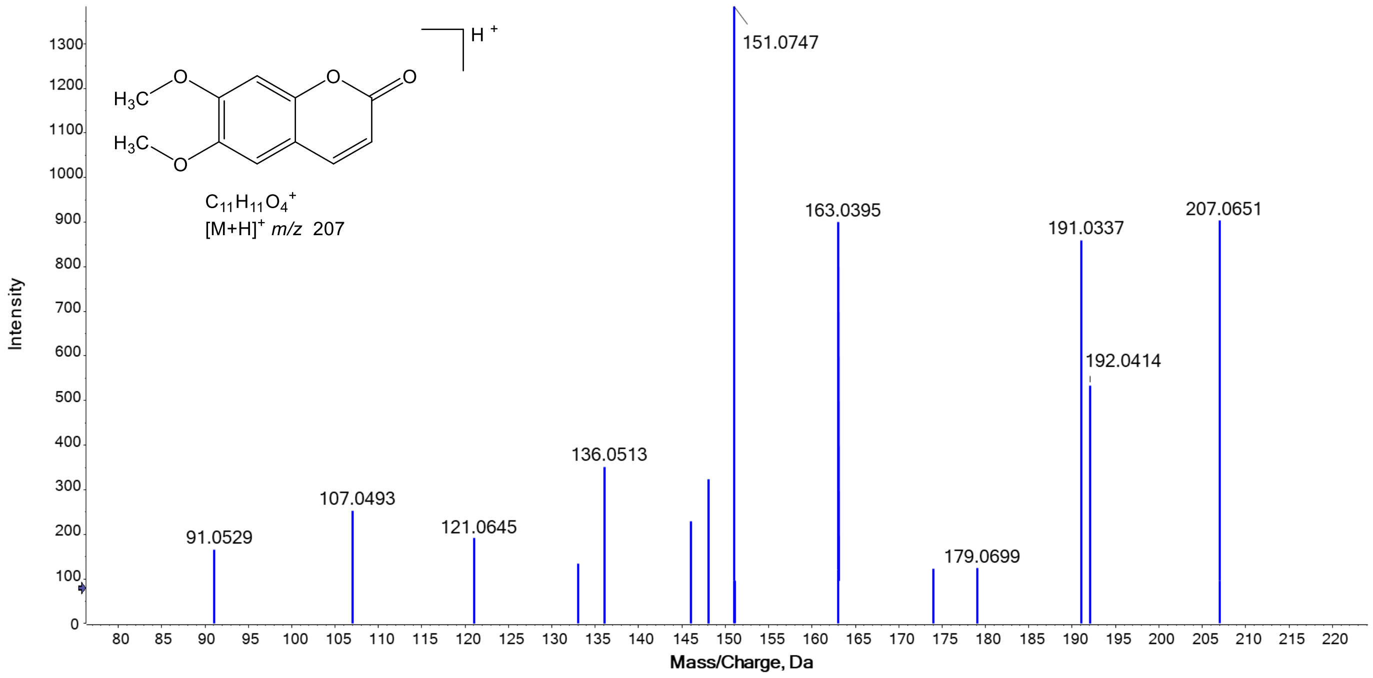

Figure S10_49: MS/MS spectrum of P49.

P50, Paprazine*

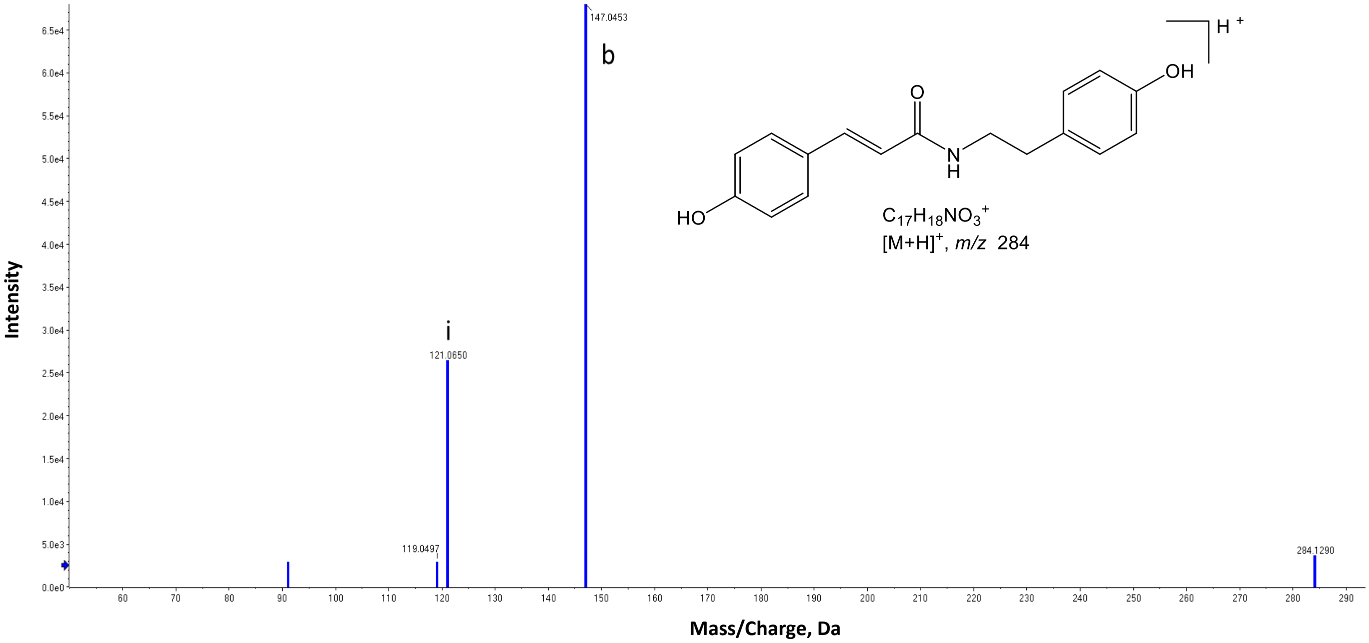

Figure S10_50: MS/MS spectrum of P50.

P51, 3,4-Dimethoxybenzalacetone

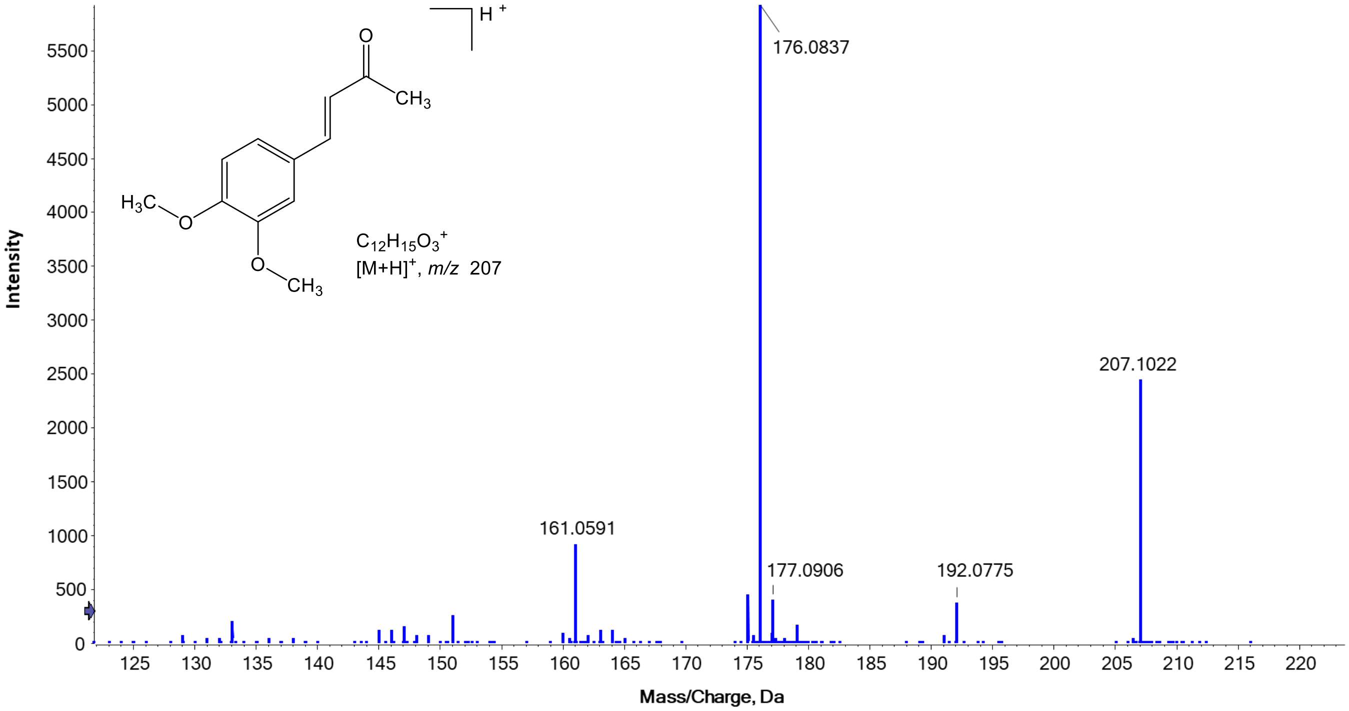

Figure S10_51: MS/MS spectrum of P51.

P52, Houttuynamide A

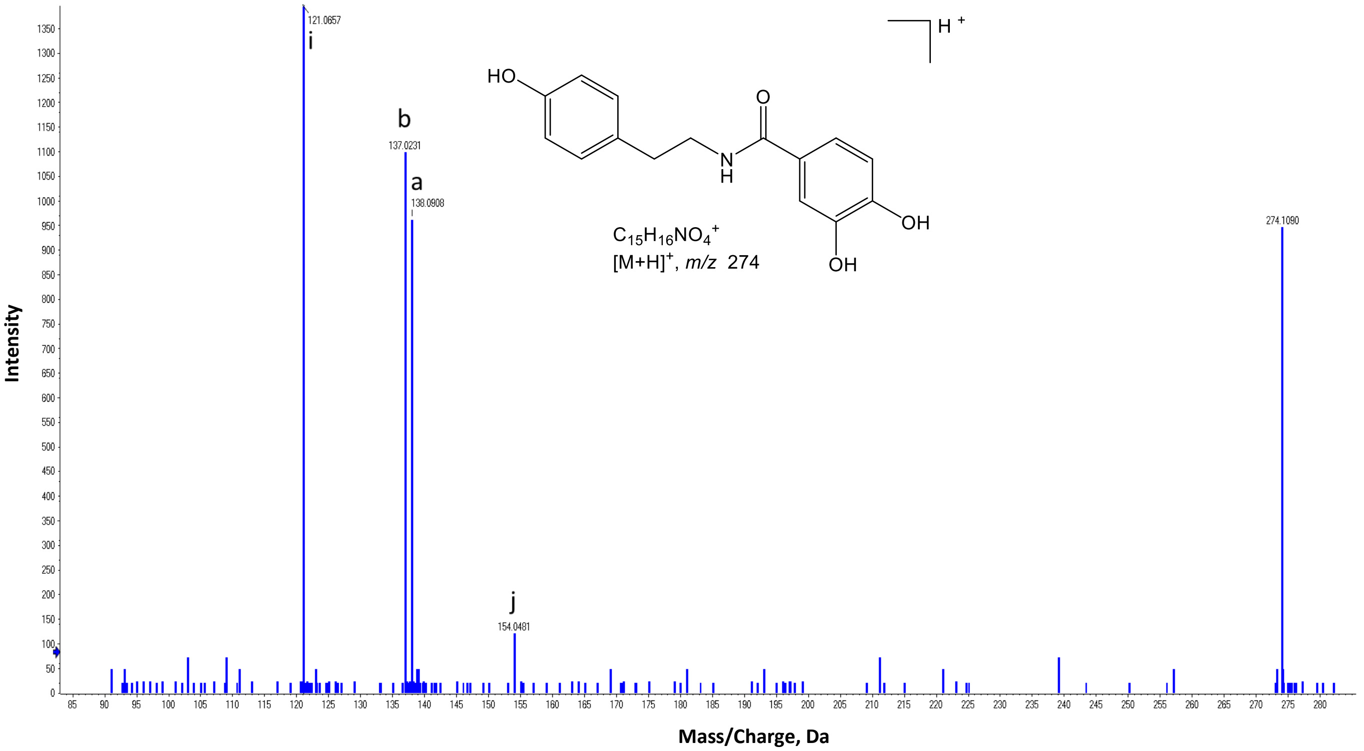

Figure S10_52: MS/MS spectrum of P52.

P53, Zingiberoside C

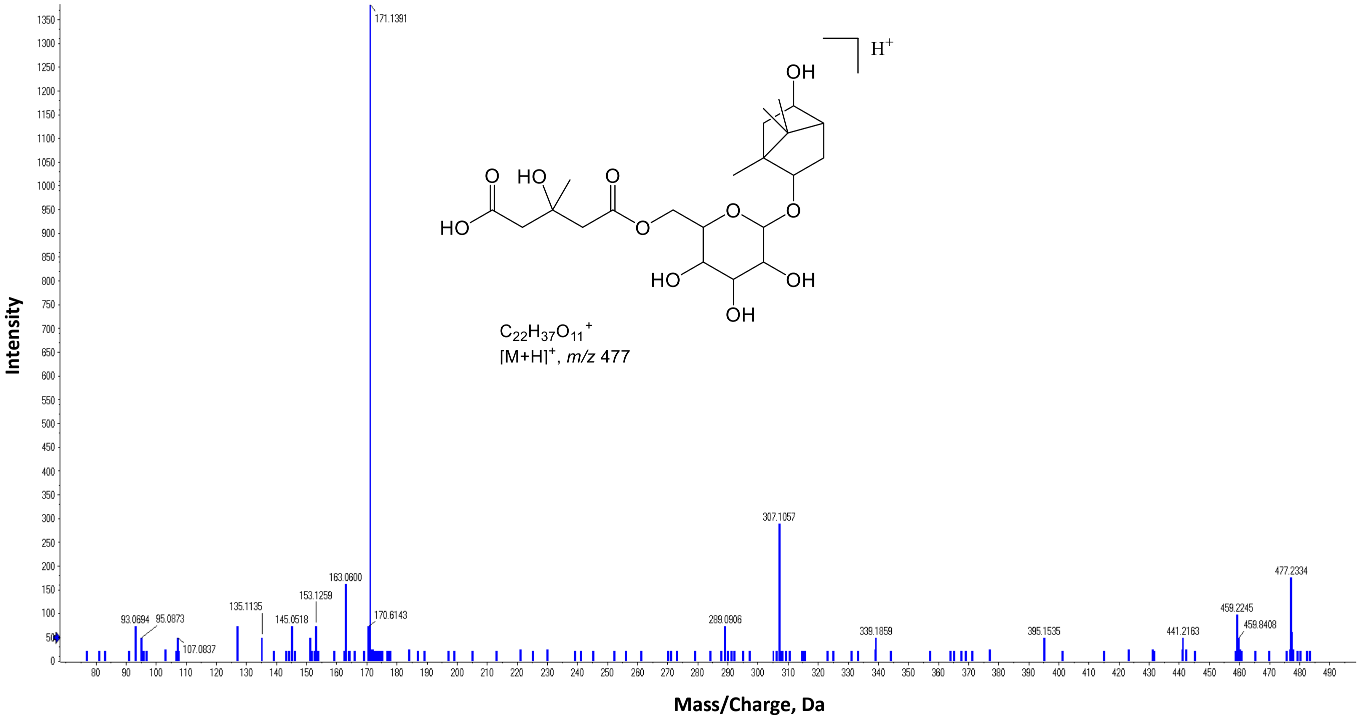

Figure S10_53: MS/MS spectrum of P53.

P54, Acylated flavonoid C-glycoside D

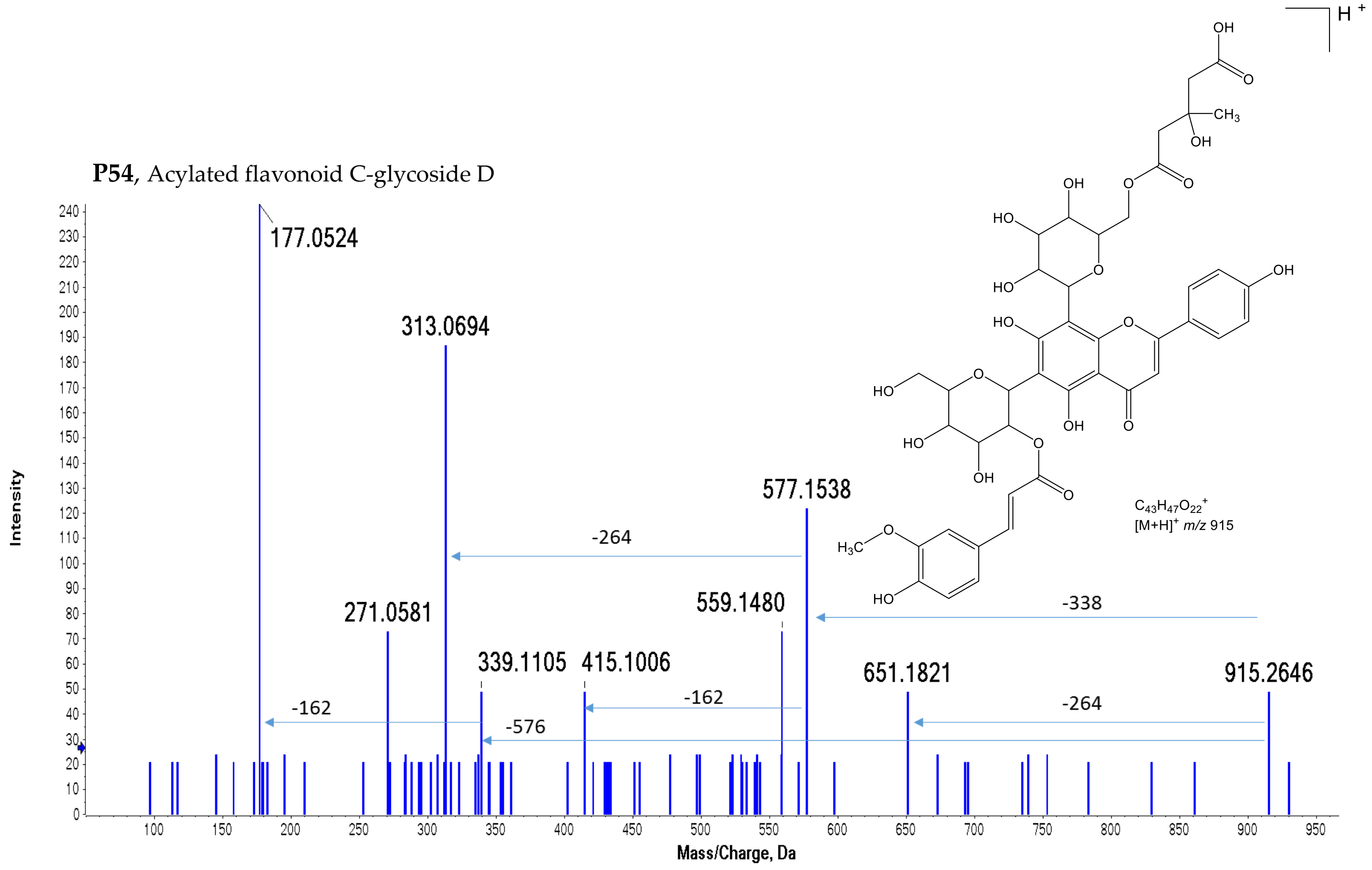

Figure S10_54: MS/MS spectrum of P54.

P55, *trans-N*-feruloyltyramine*

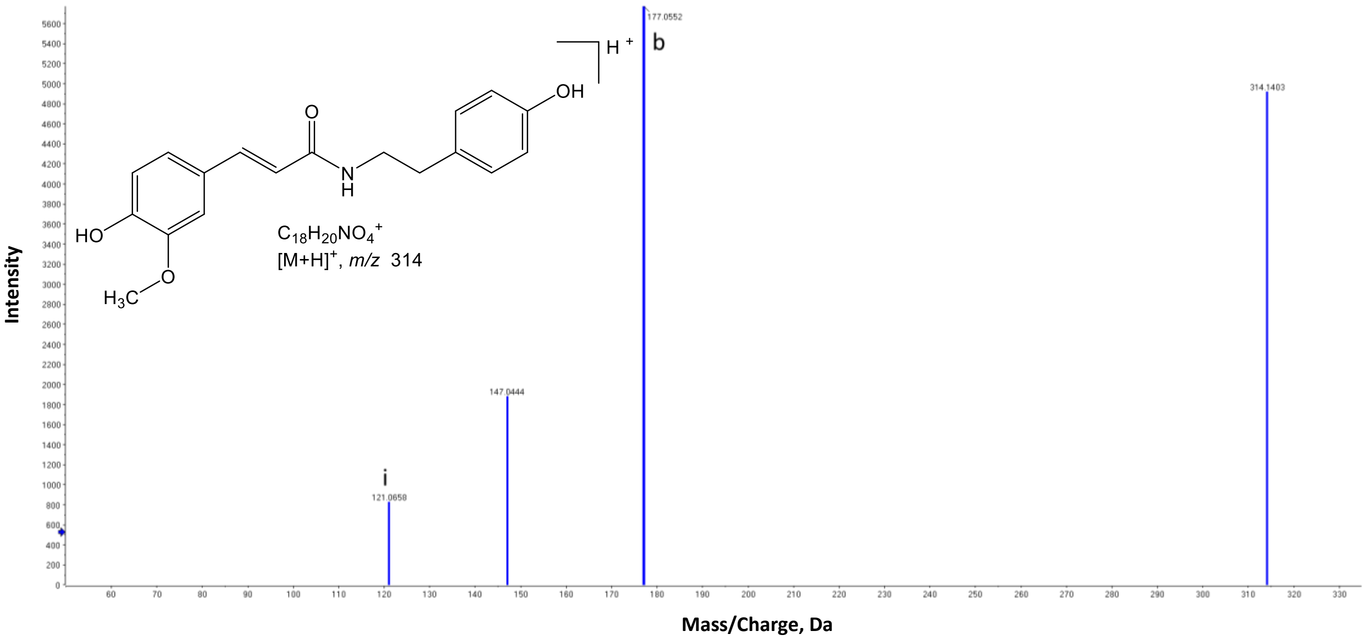

Figure S10_55: MS/MS spectrum of P55.

P56, Asarylaldehyde*

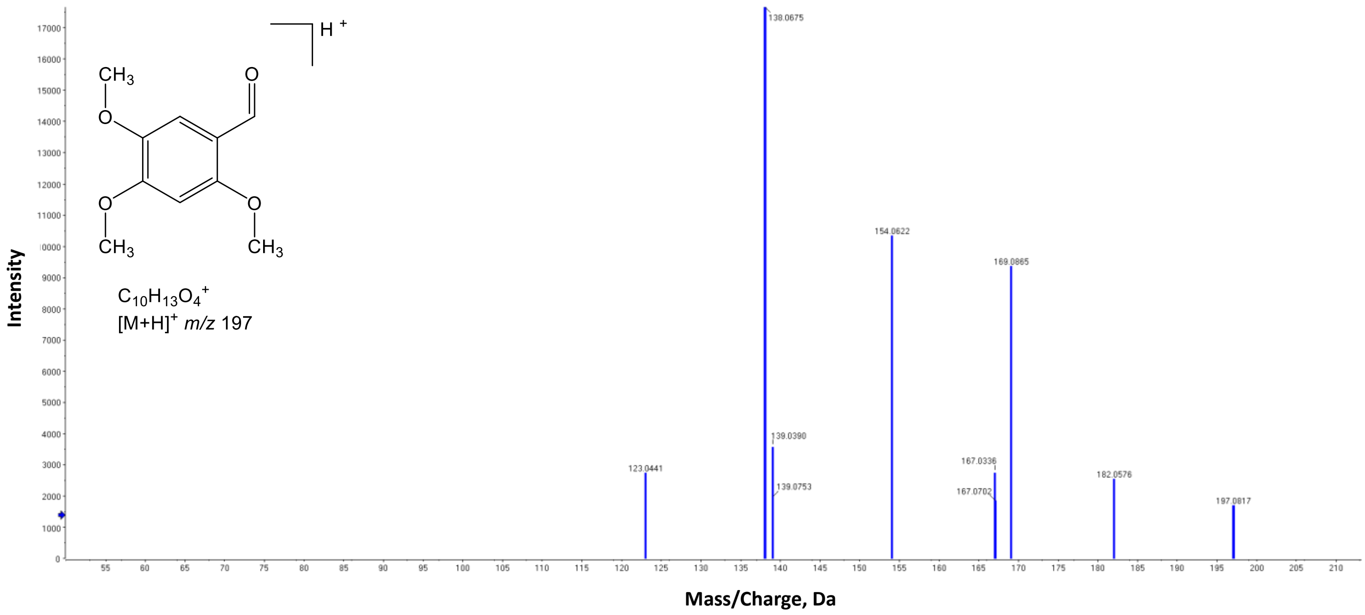

Figure S10_56: MS/MS spectrum of P56.

P57, Demethoxypiperlotine C

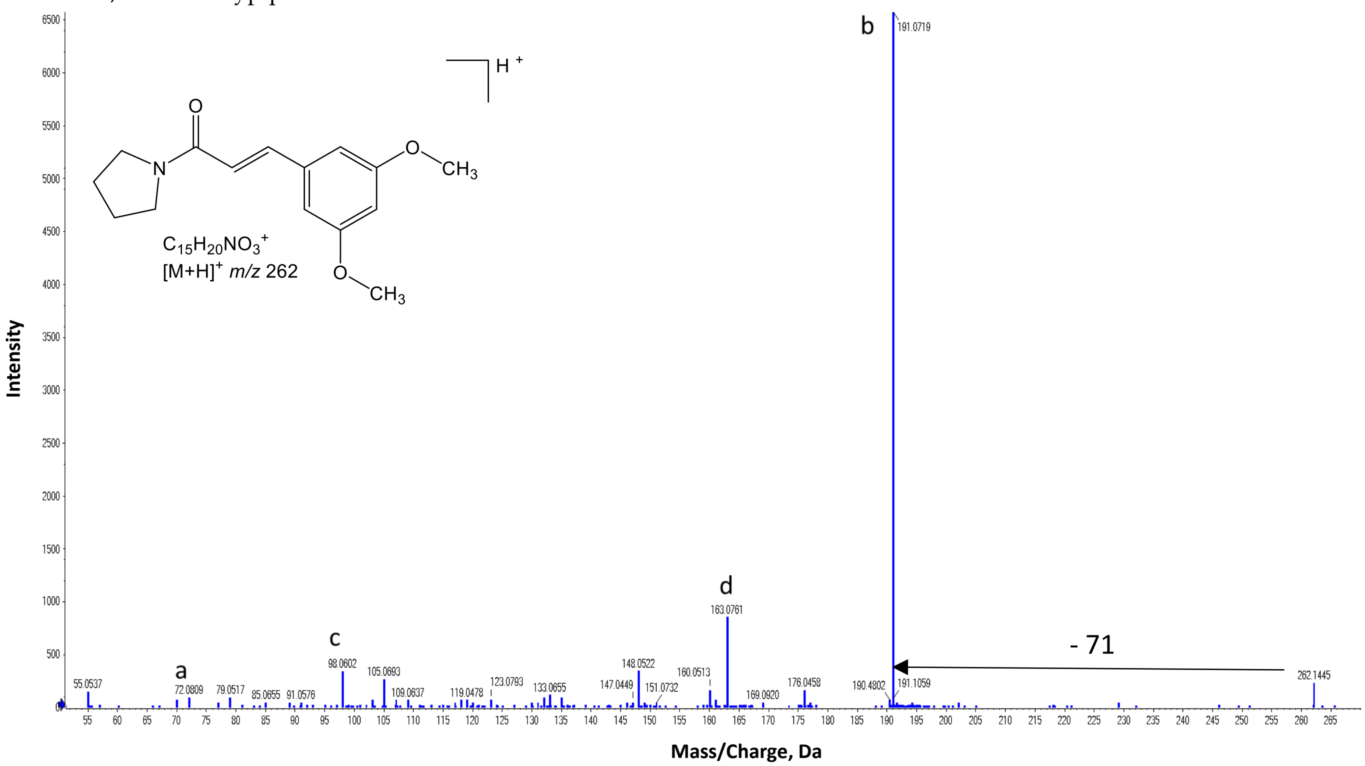

Figure S10_57: MS/MS spectrum of P57.

P58, Lignanamide A

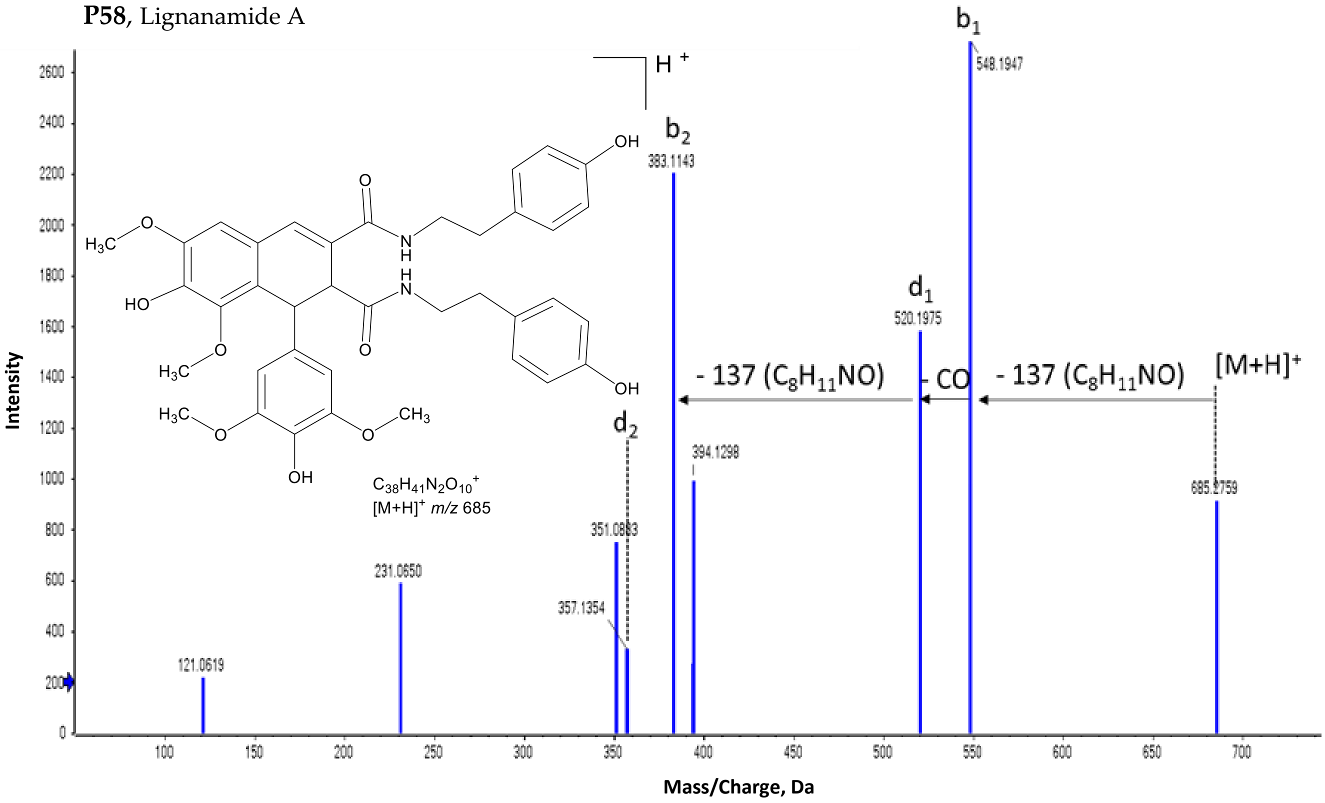

Figure S10_58: MS/MS spectrum of P58.

P59, Norcoclaurine

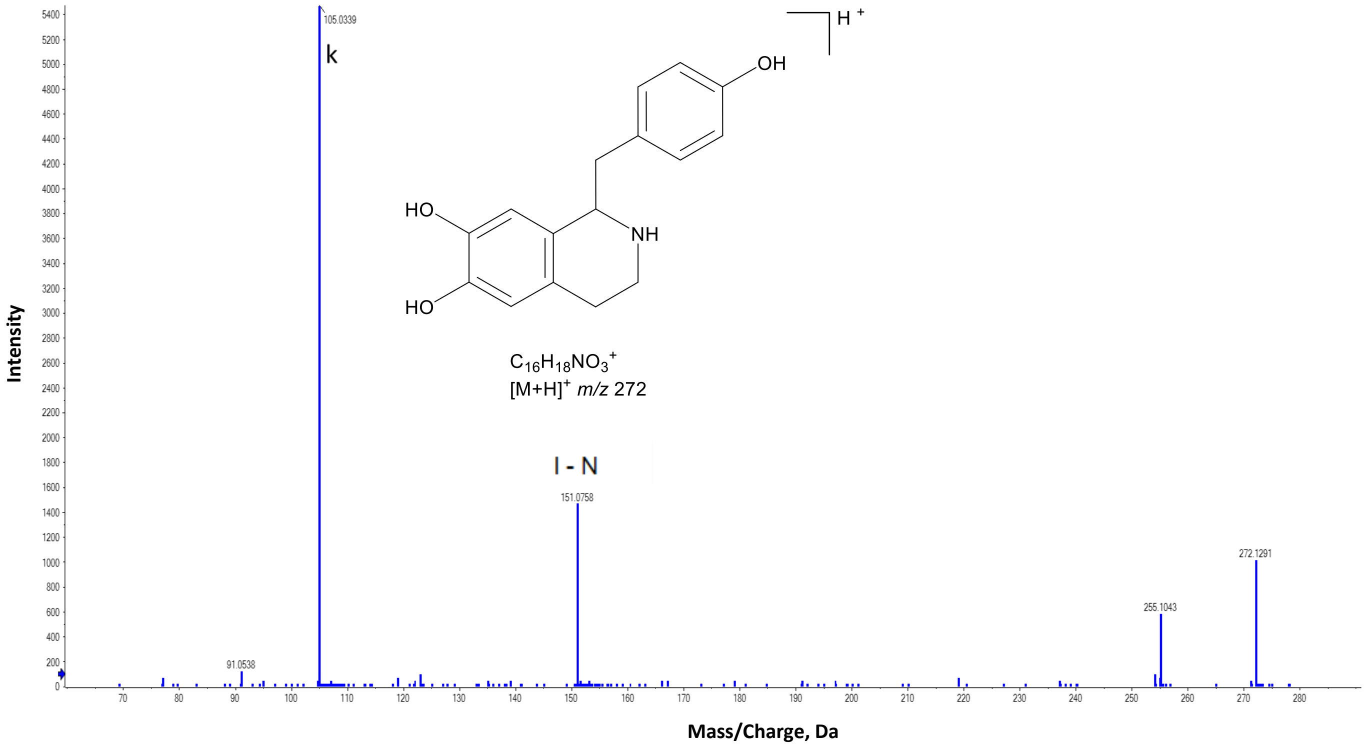

Figure S10_59: MS/MS spectrum of P59.

P60, Methoxycannabisin D

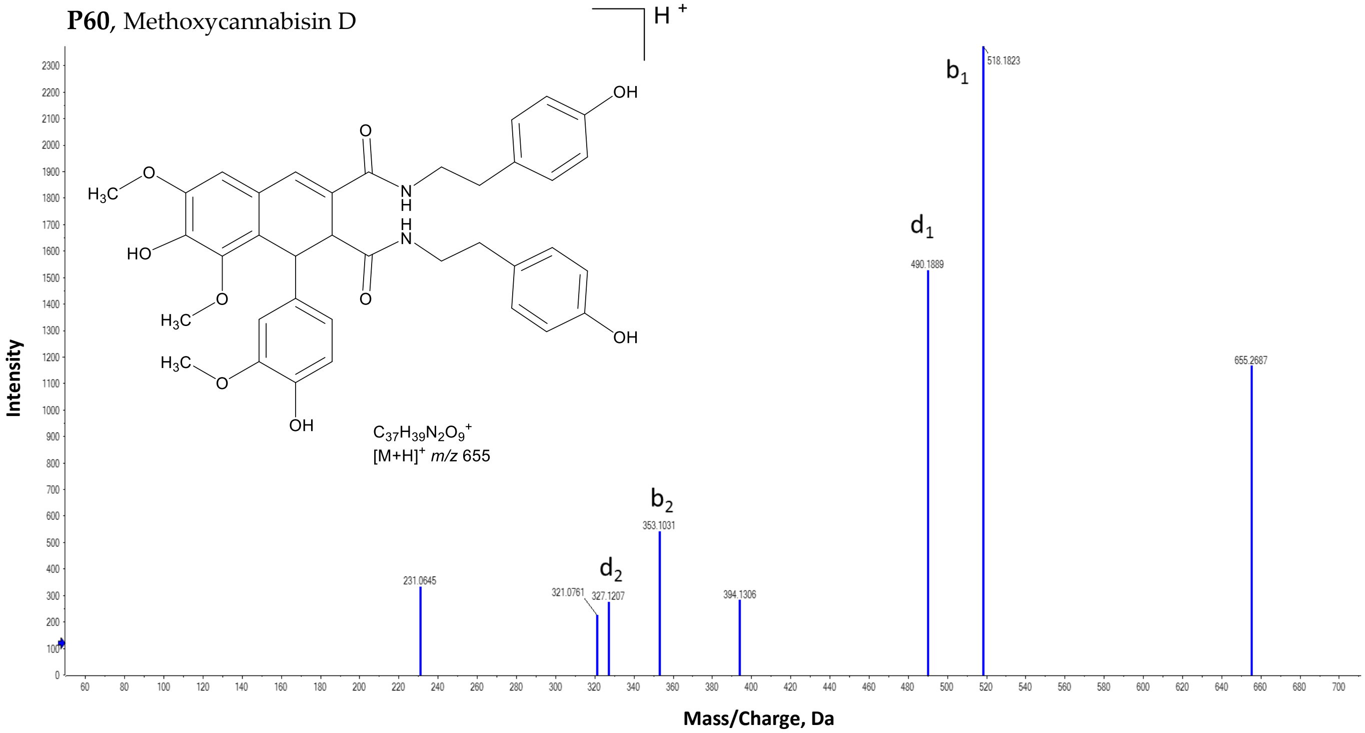

Figure S10_60: MS/MS spectrum of P60.

P61, Piperlotine C

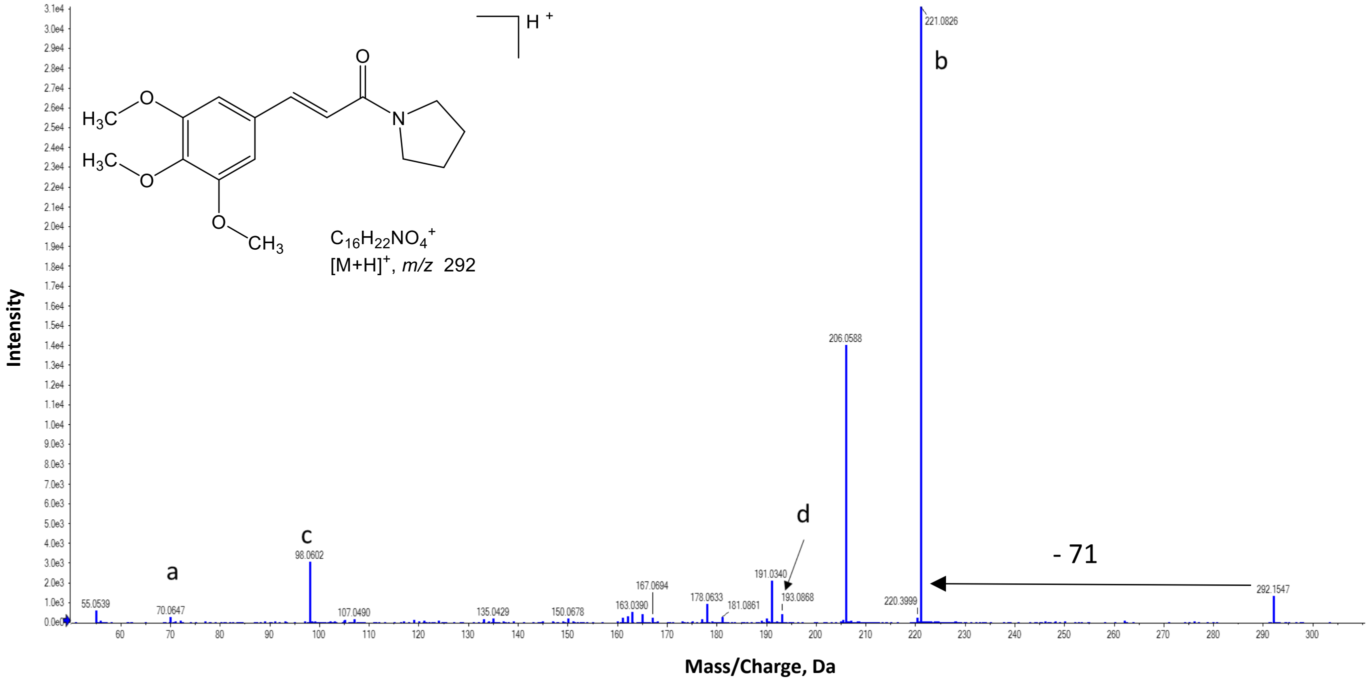

Figure S10_61: MS/MS spectrum of P61.

P62: $C_{15}H_{20}NO_3^+$, isomer of Demethoxypiperlotine C (formula see **P57**)

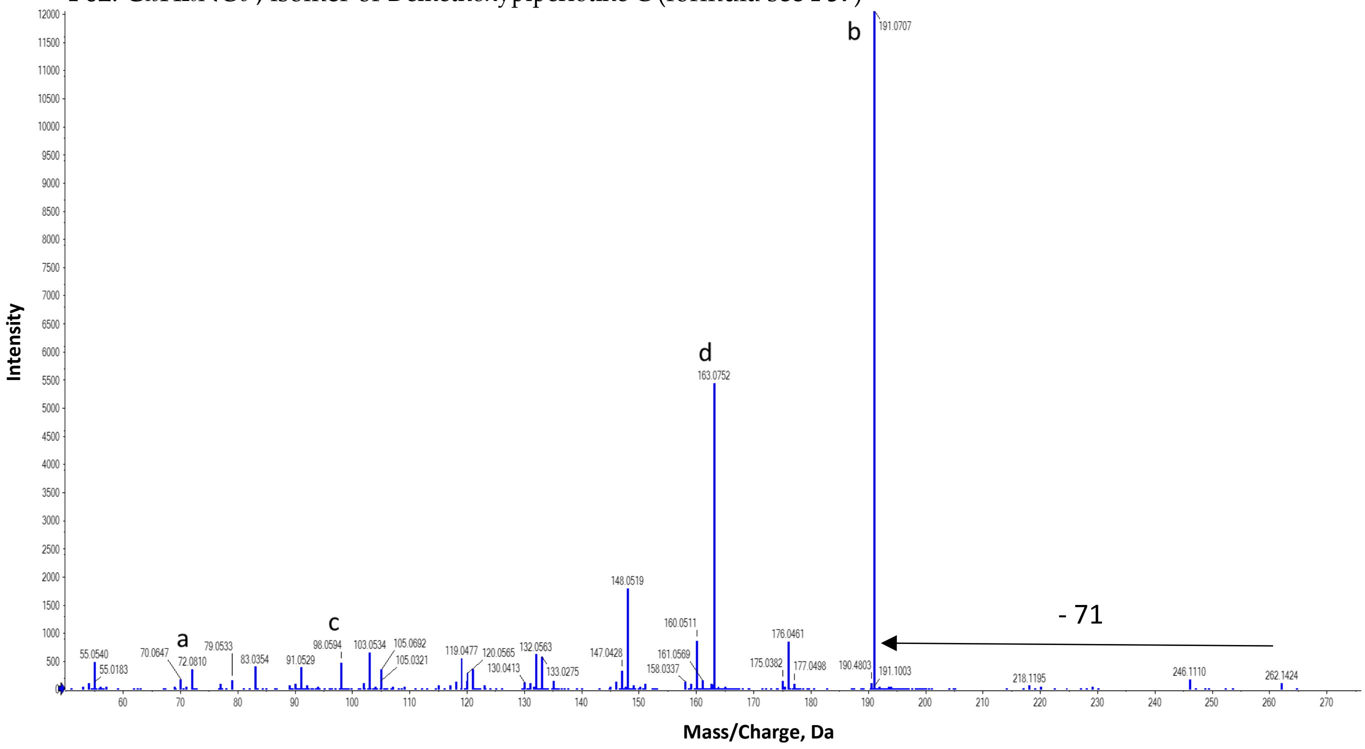

Figure S10_62: MS/MS spectrum of P62.

P63, Hippacine

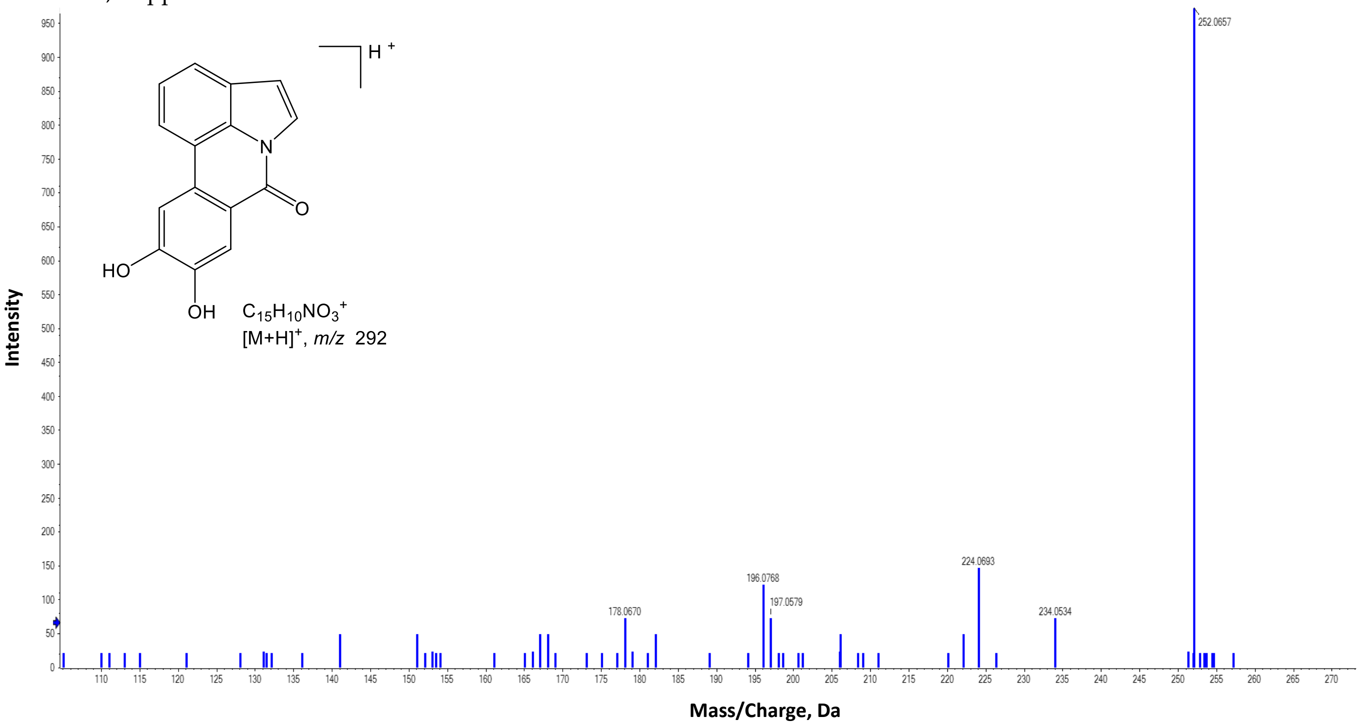

Figure S10_63: MS/MS spectrum of P63.

P64, Ganoapplanatumine B

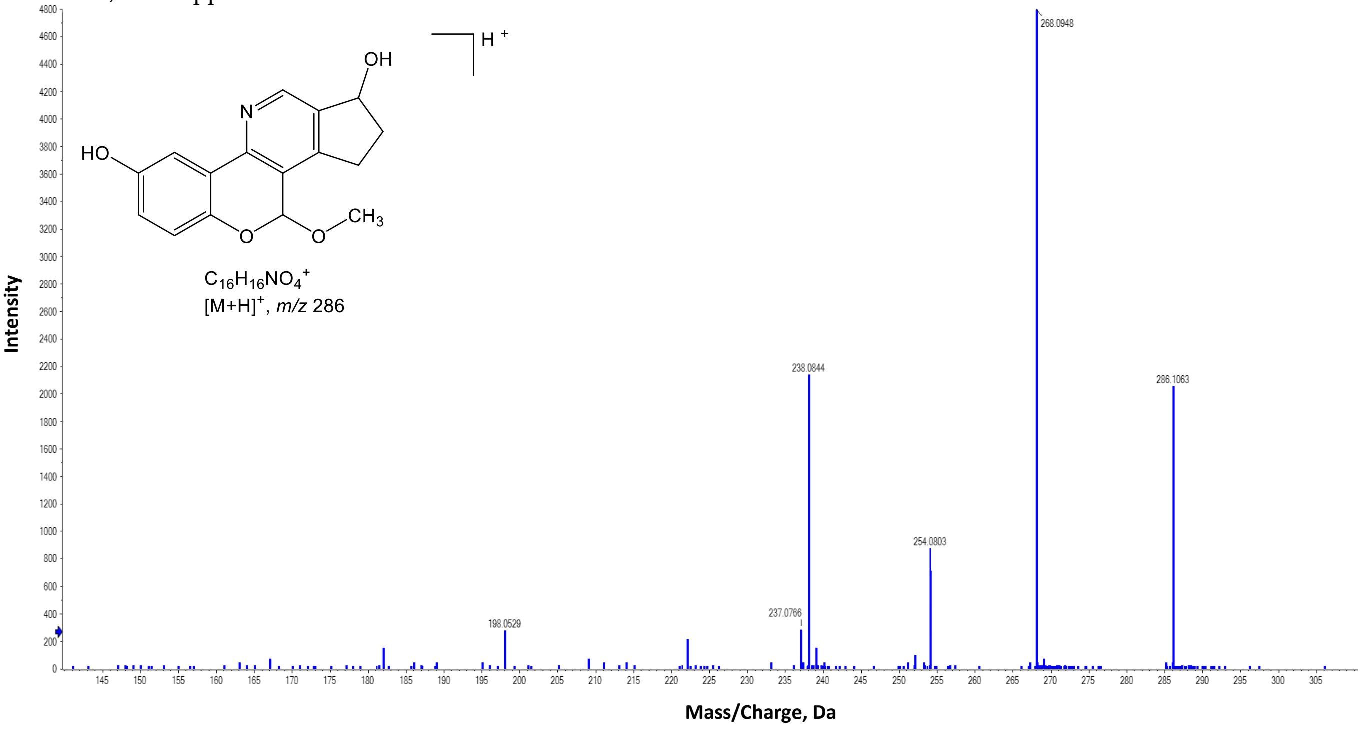

Figure S10_64: MS/MS spectrum of P64.

P65: $C_{16}H_{22}NO_4^+$, isomer of piperlotine C (formula see **P61**)

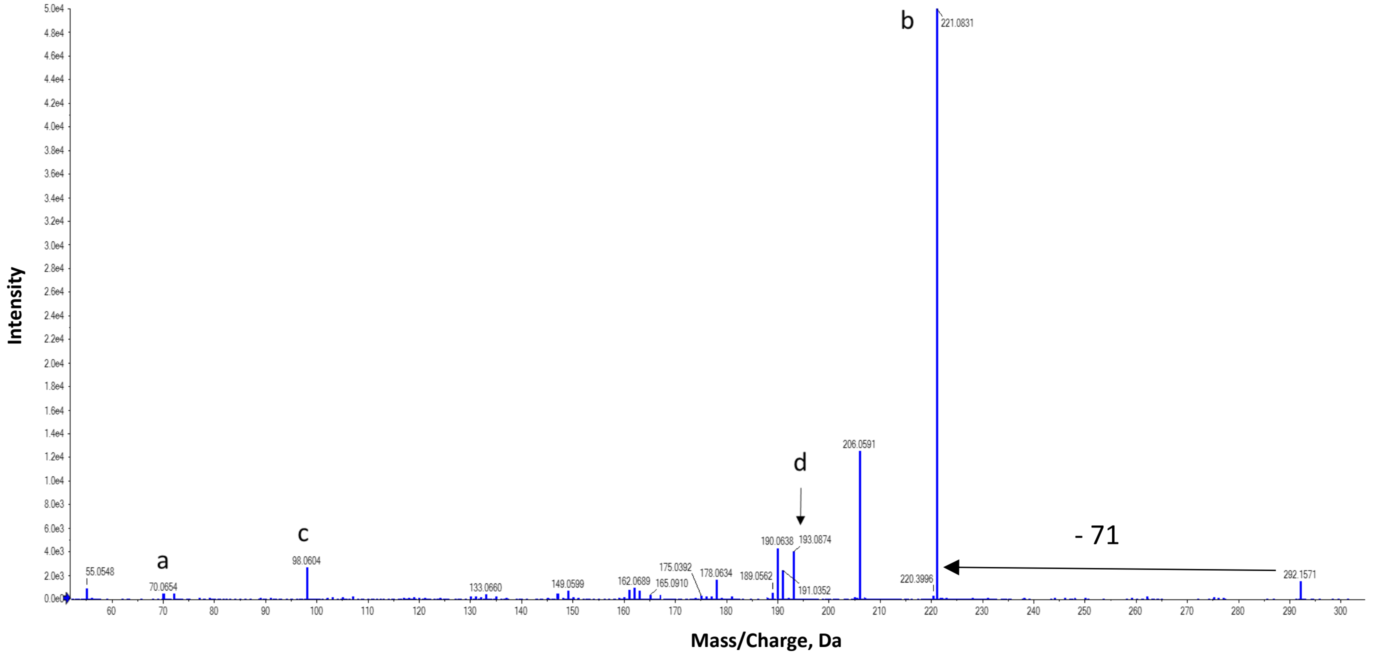

Figure S10_65: MS/MS spectrum of P65.

P66, Feruloylphenethylamine

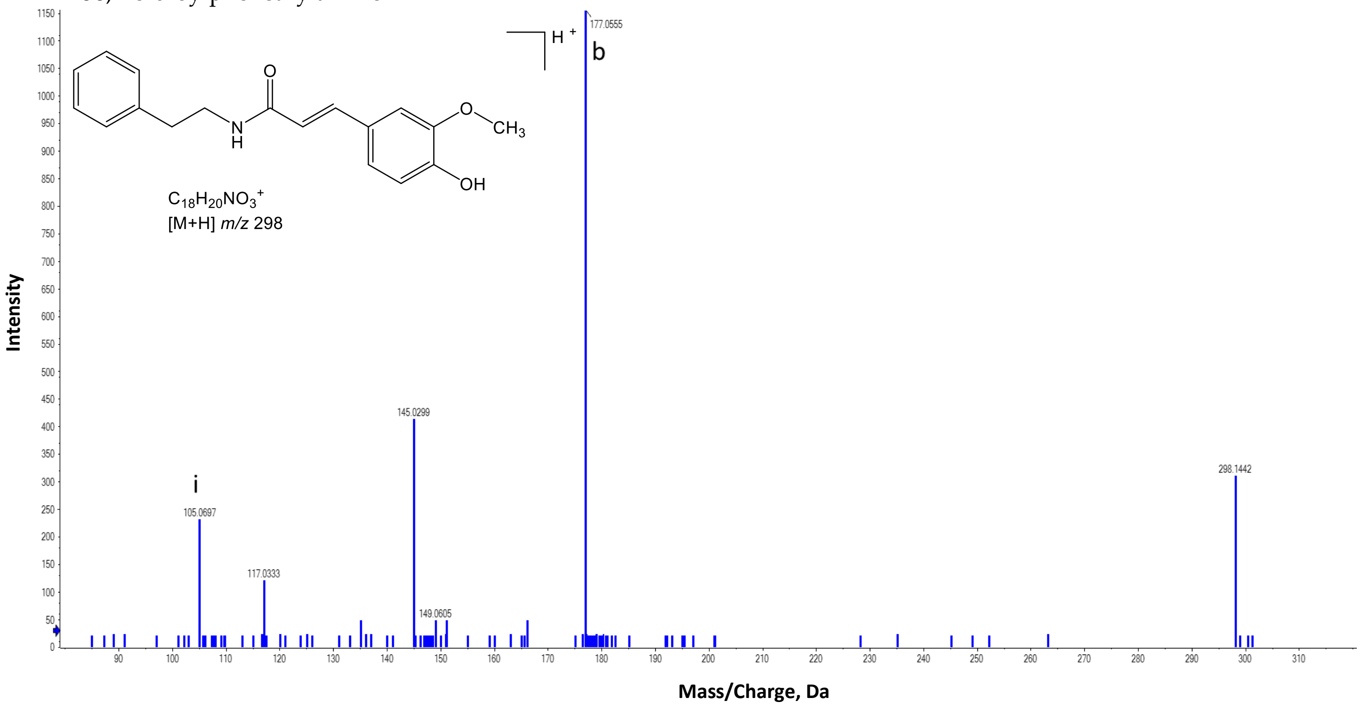

Figure S10_66: MS/MS spectrum of P66.

P67, 3-(7-methoxy-1,3-benzodioxol-5-yl)prop-2-enal

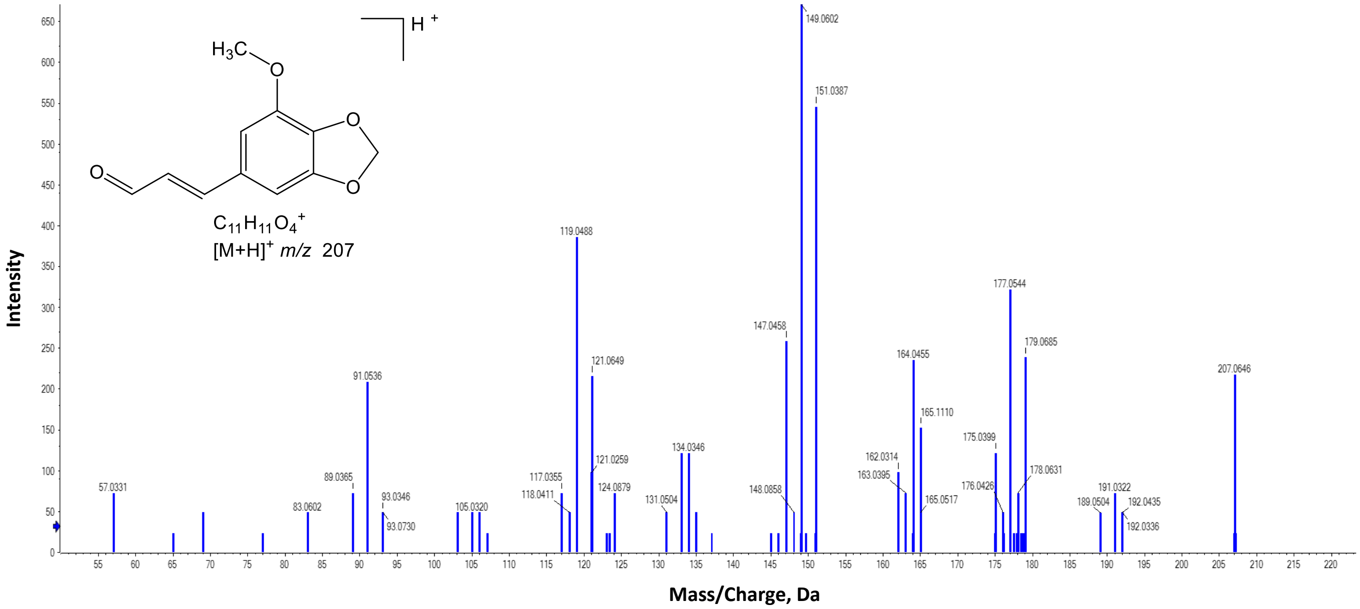

Figure S10_67: MS/MS spectrum of P67.

P68: $C_{37}H_{39}N_2O_9^+$, isomer of Methoxycannabisin D (formula see **P60**)

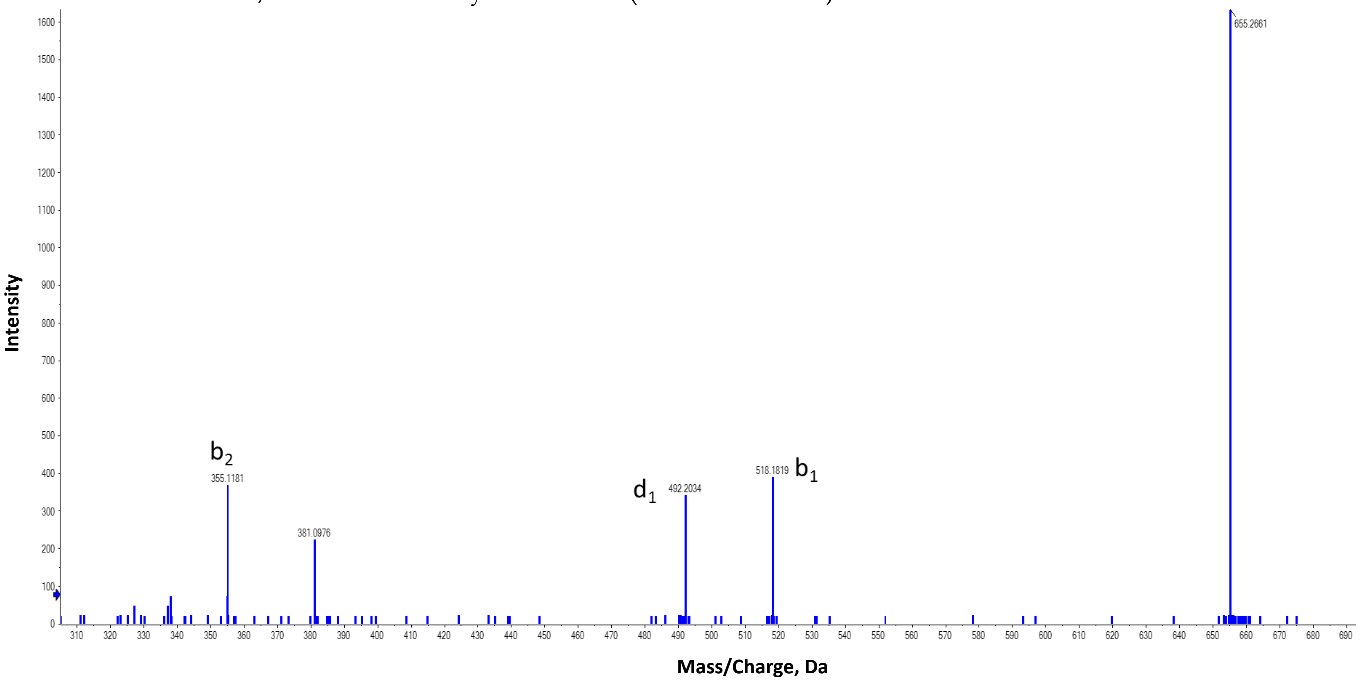

Figure S10_68: MS/MS spectrum of P68.

P69, Piperlotine A

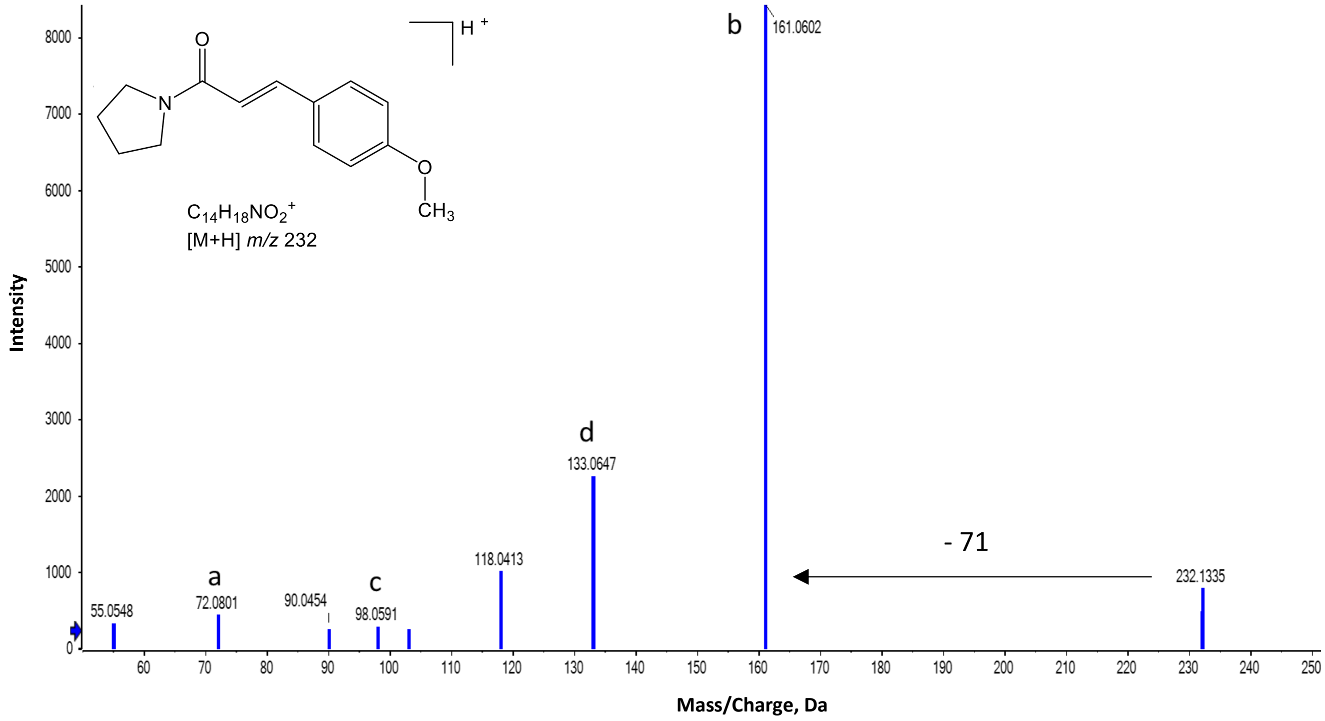

Figure S10_69: MS/MS spectrum of P69.

P70, Piperolactam A*

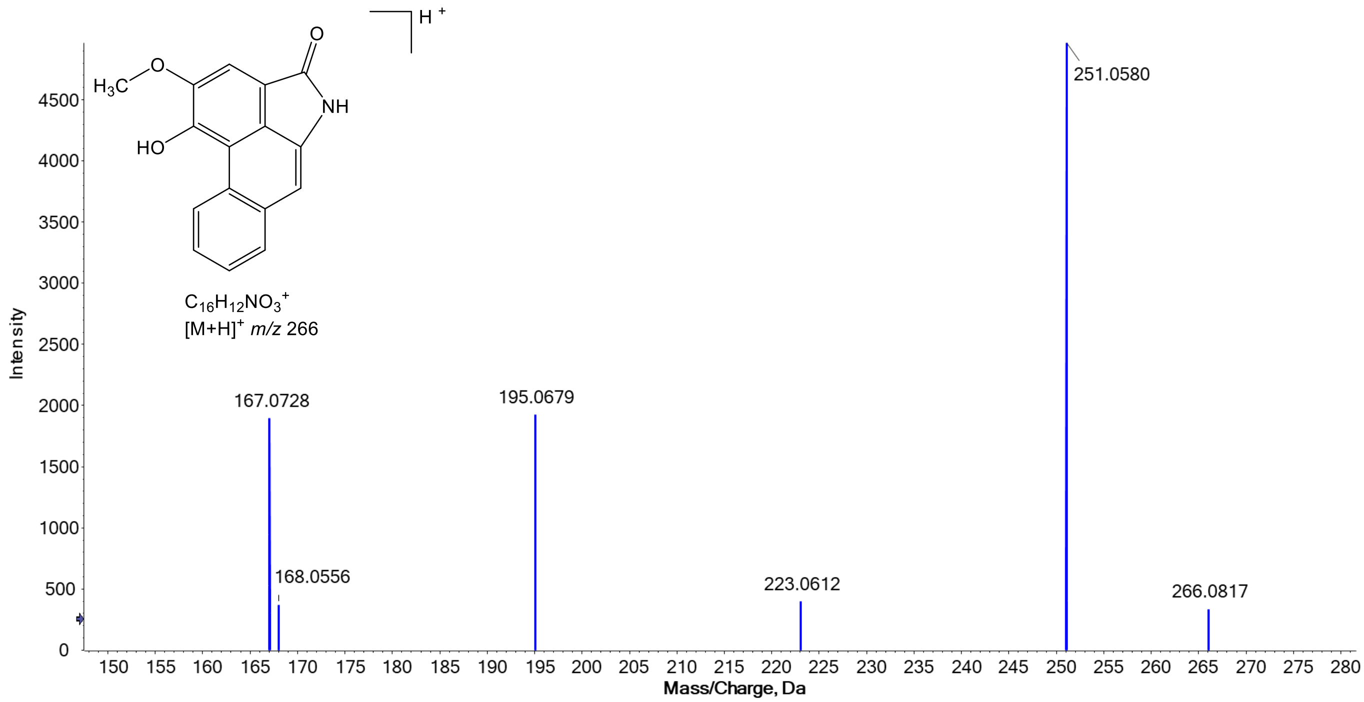

Figure S10_70: MS/MS spectrum of P70.

P71, Norcepharadione B*

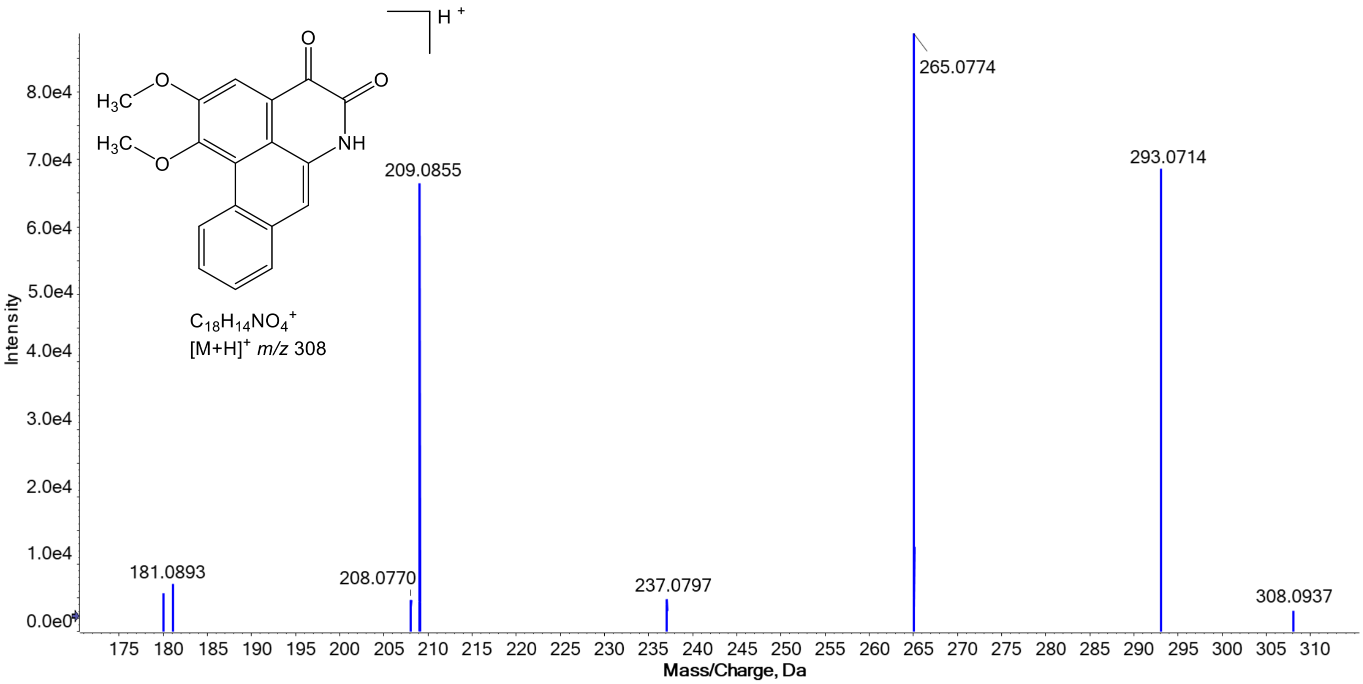

Figure S10_71: MS/MS spectrum of P71.

P72, Dehydropiperlotine A

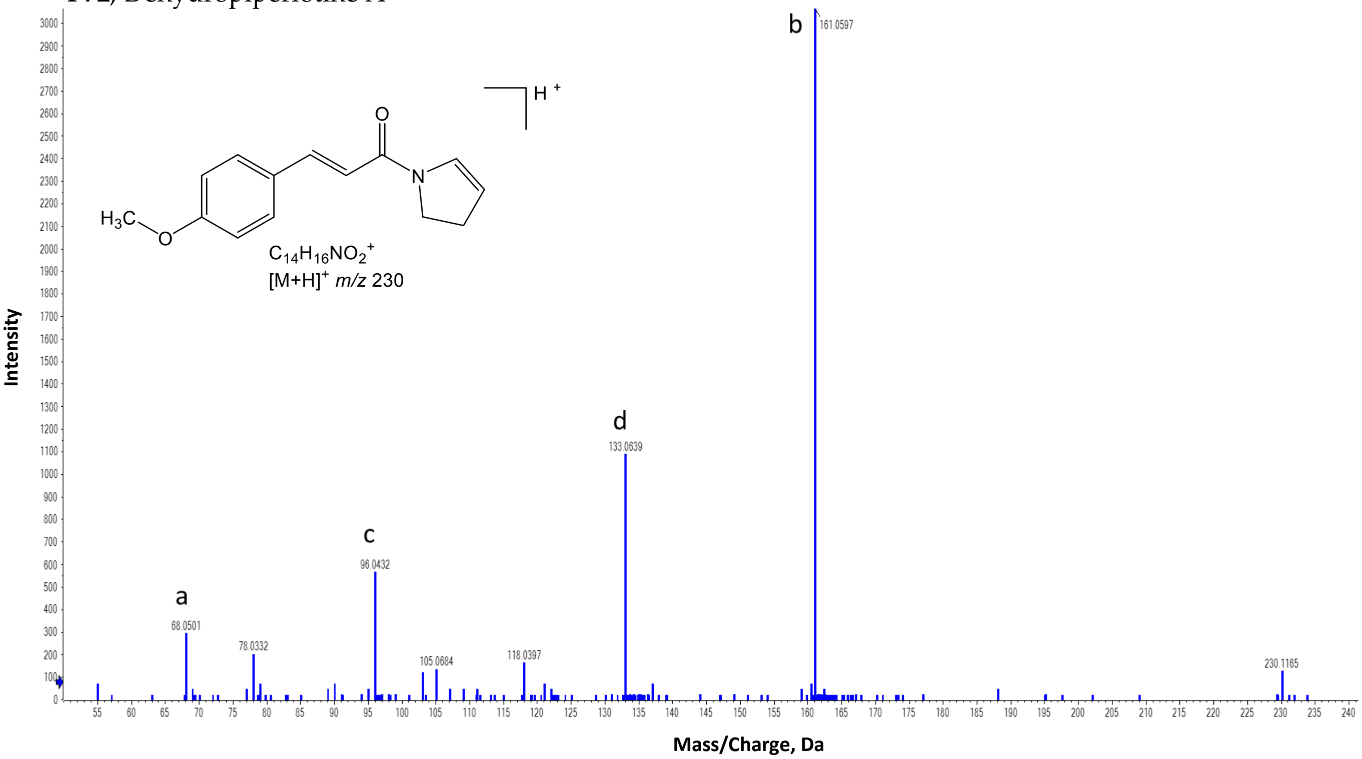

Figure S10_72: MS/MS spectrum of P72.

P73: $C_{15}H_{20}NO_3^+$, isomer of Demethoxypiperlotine C (formula see **P57**)

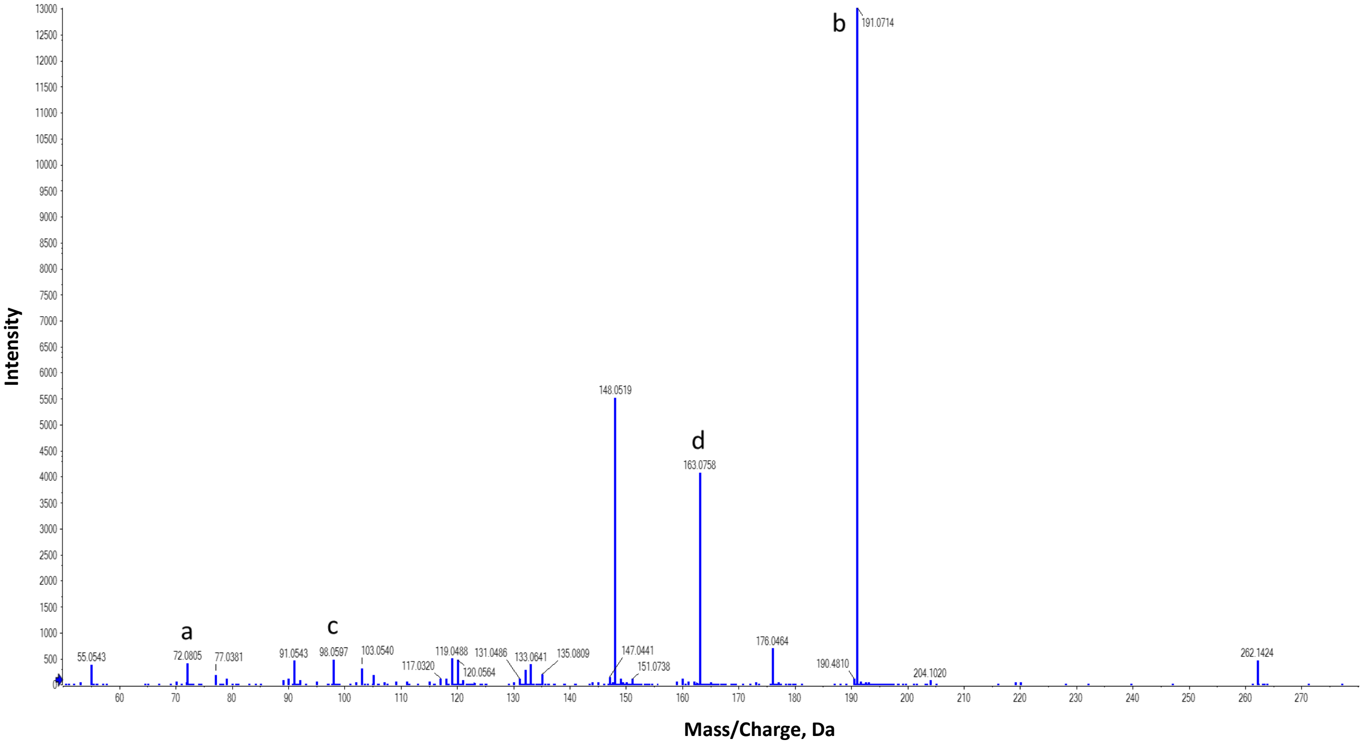

Figure S10_73: MS/MS spectrum of P73.

P74, Lignanamide B

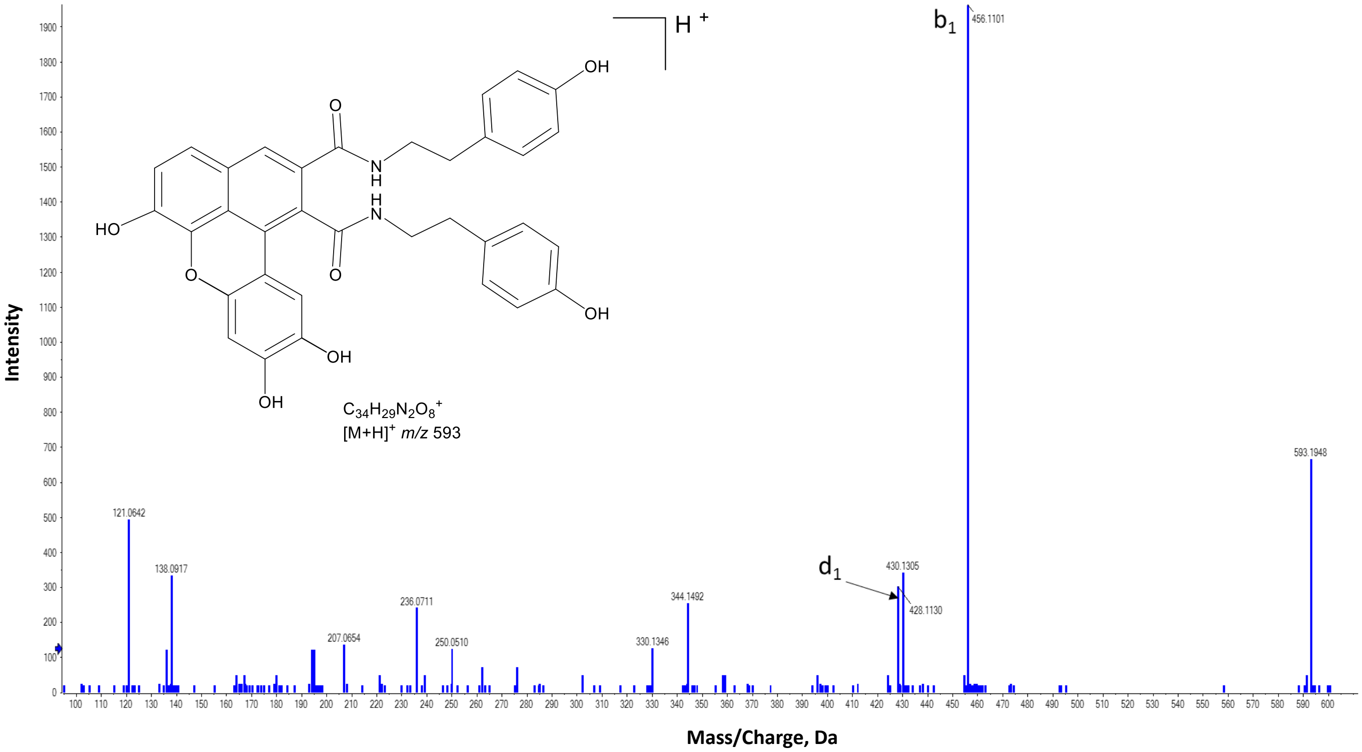

Figure S10_74: MS/MS spectrum of P74.

P75, Piperolactam B

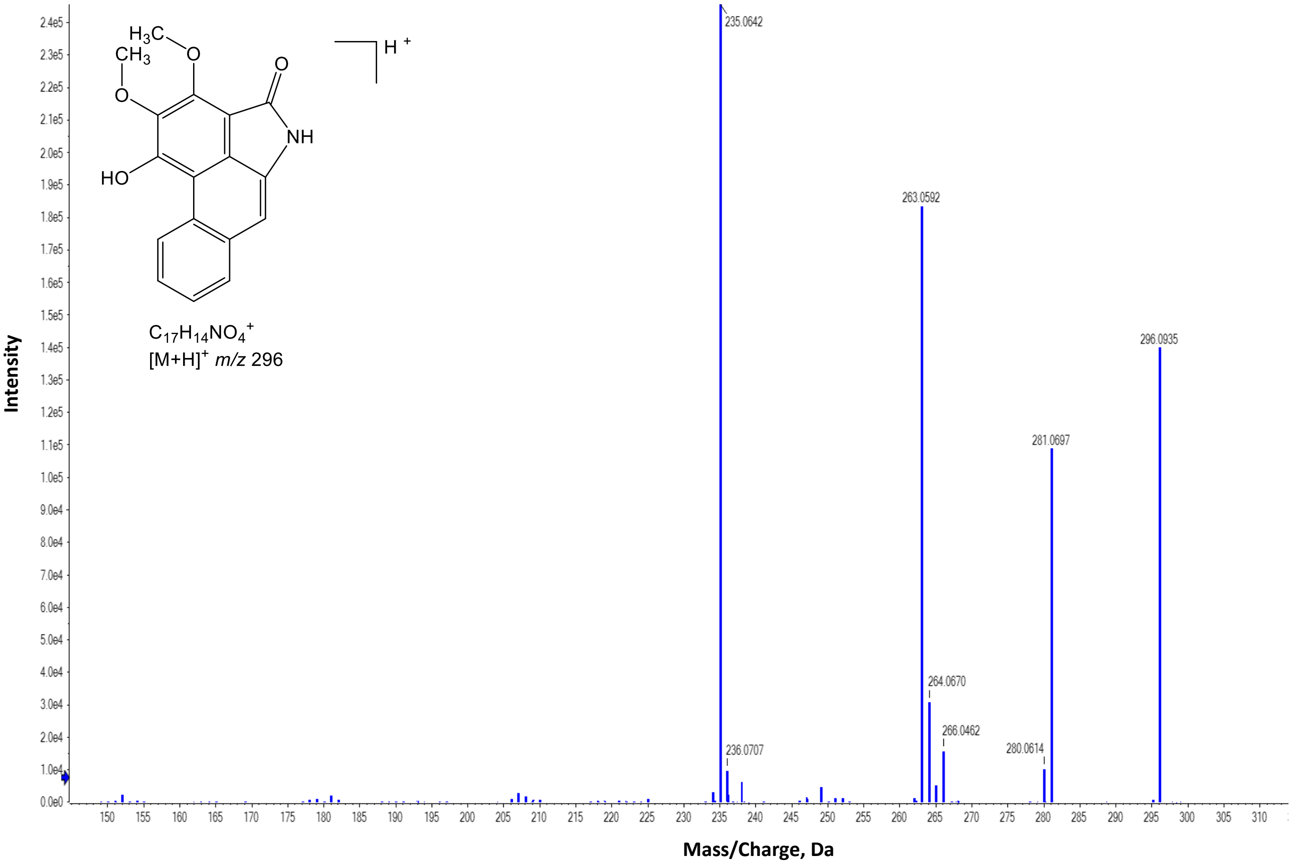

Figure S10_75: MS/MS spectrum of P75.

P76, Piperadione/ Aristolodione

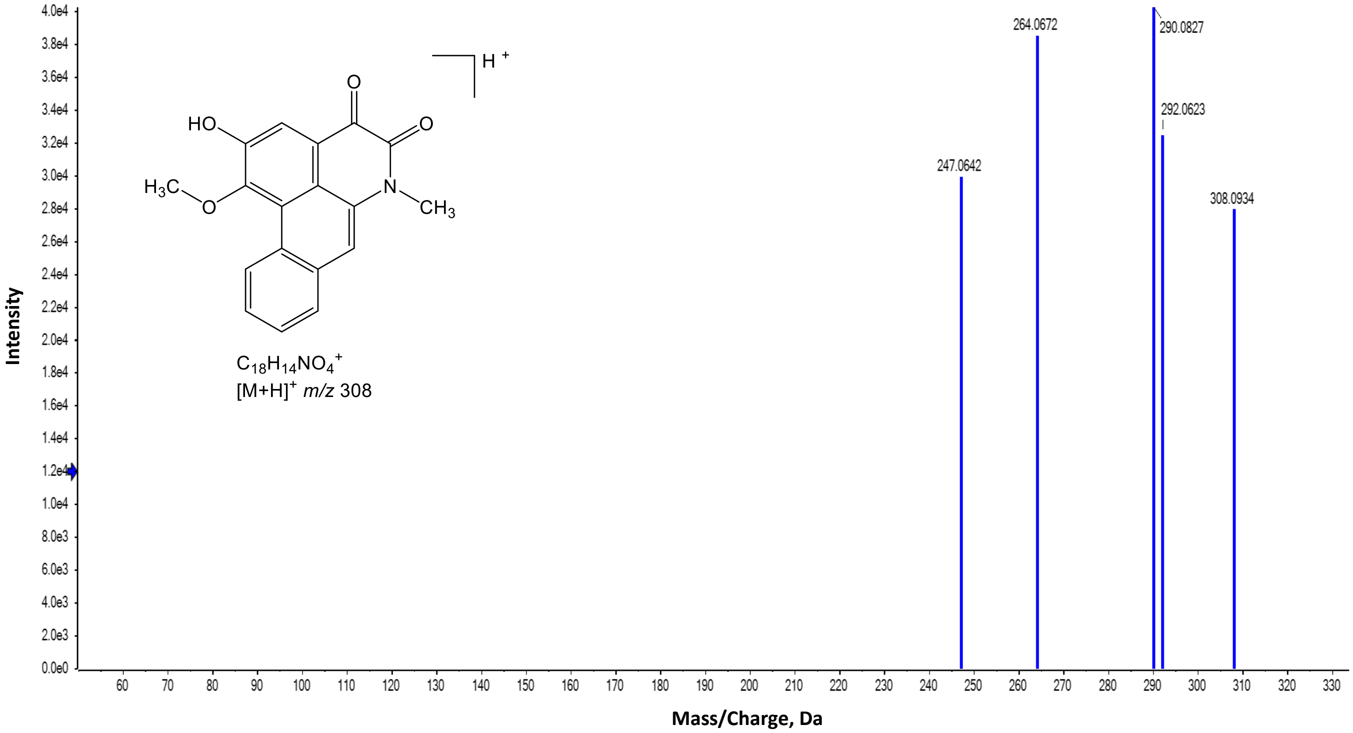

Figure S10_76: MS/MS spectrum of P76.

P77, Cepharanone D

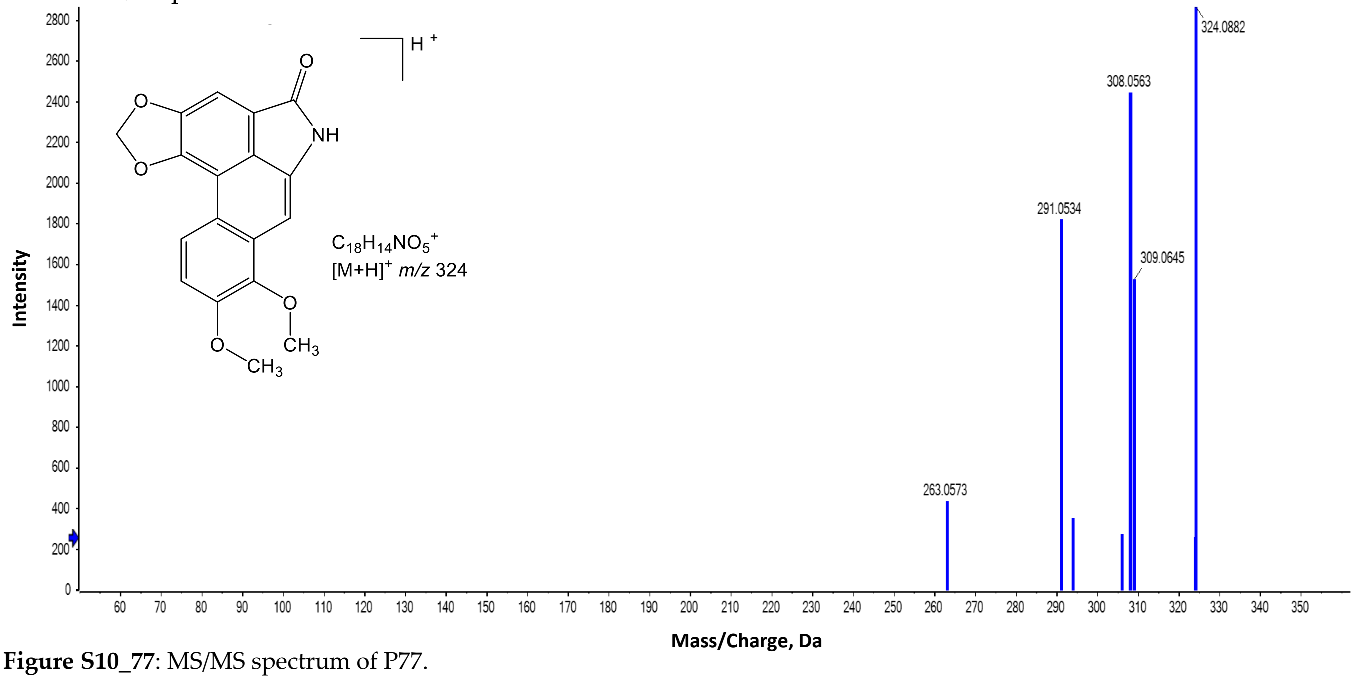

Figure S10_77: MS/MS spectrum of P77.

P78, Piperamide-C5:1

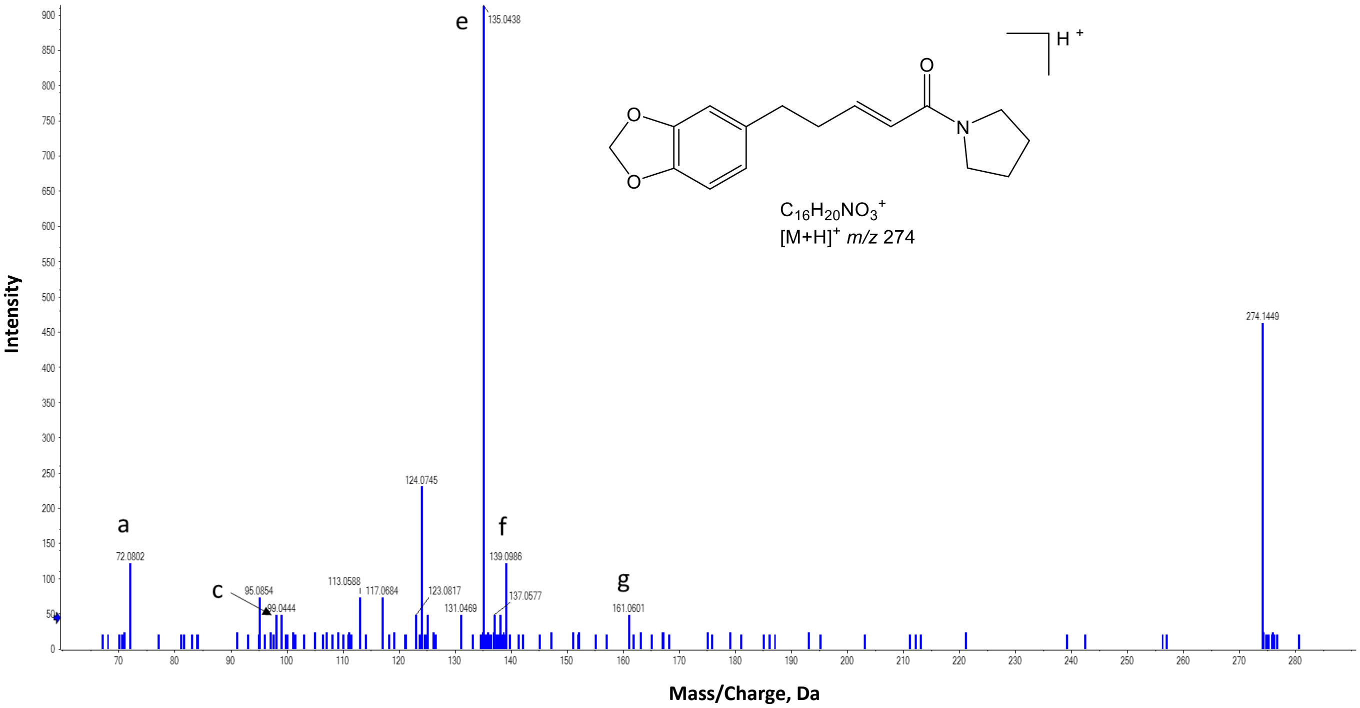

Figure S10_78: MS/MS spectrum of P78.

P79, Piperlotine G

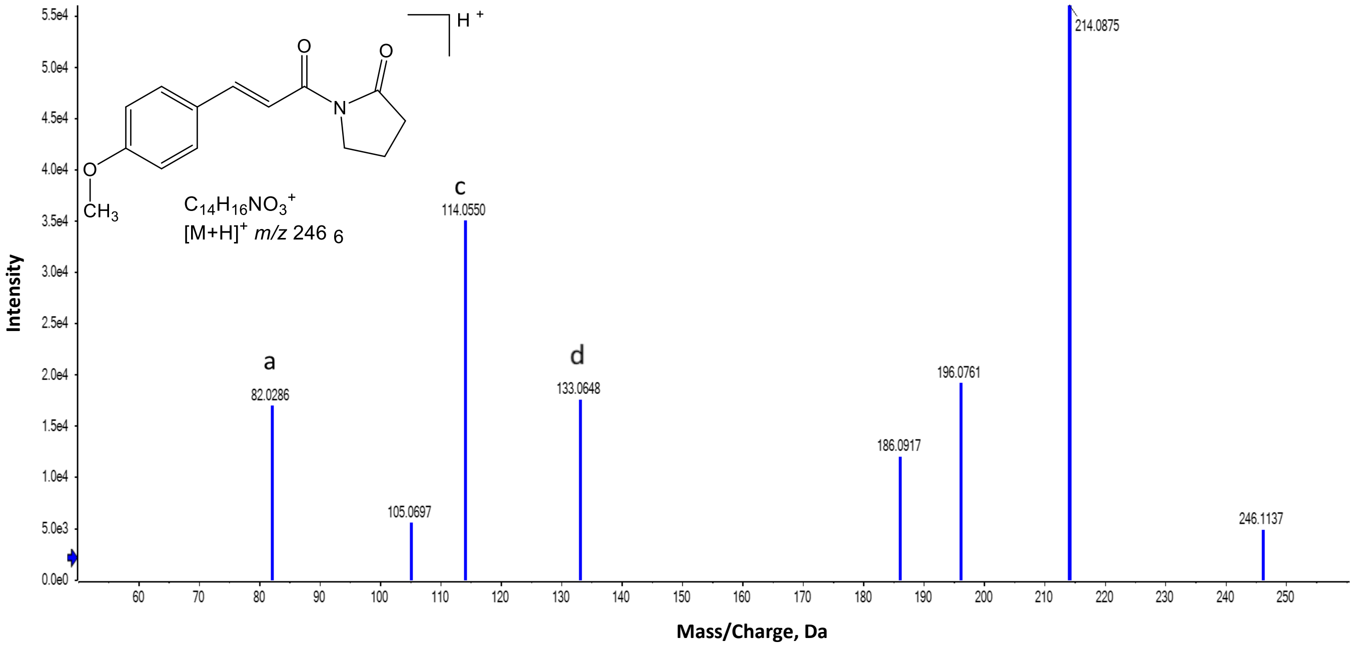

Figure S10_79: MS/MS spectrum of P79.

P80, Piperyline

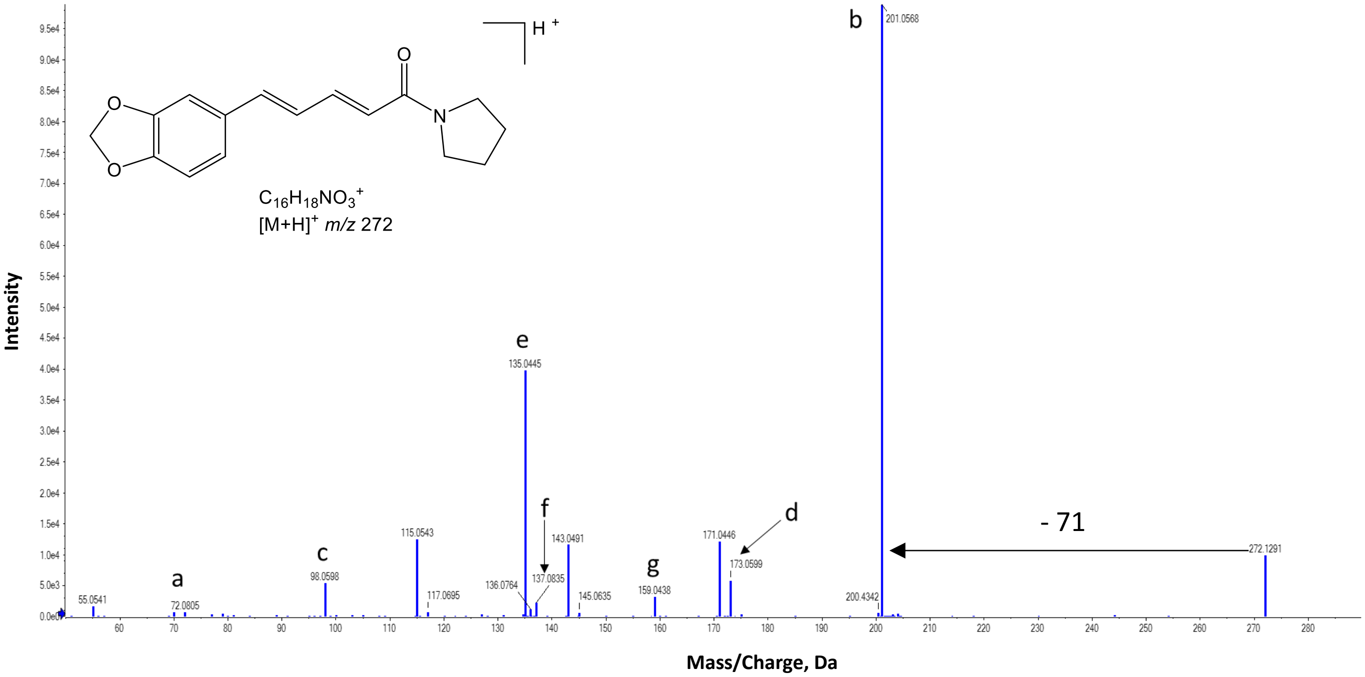

Figure S10_80: MS/MS spectrum of P80.

P81, Cepharadione A*

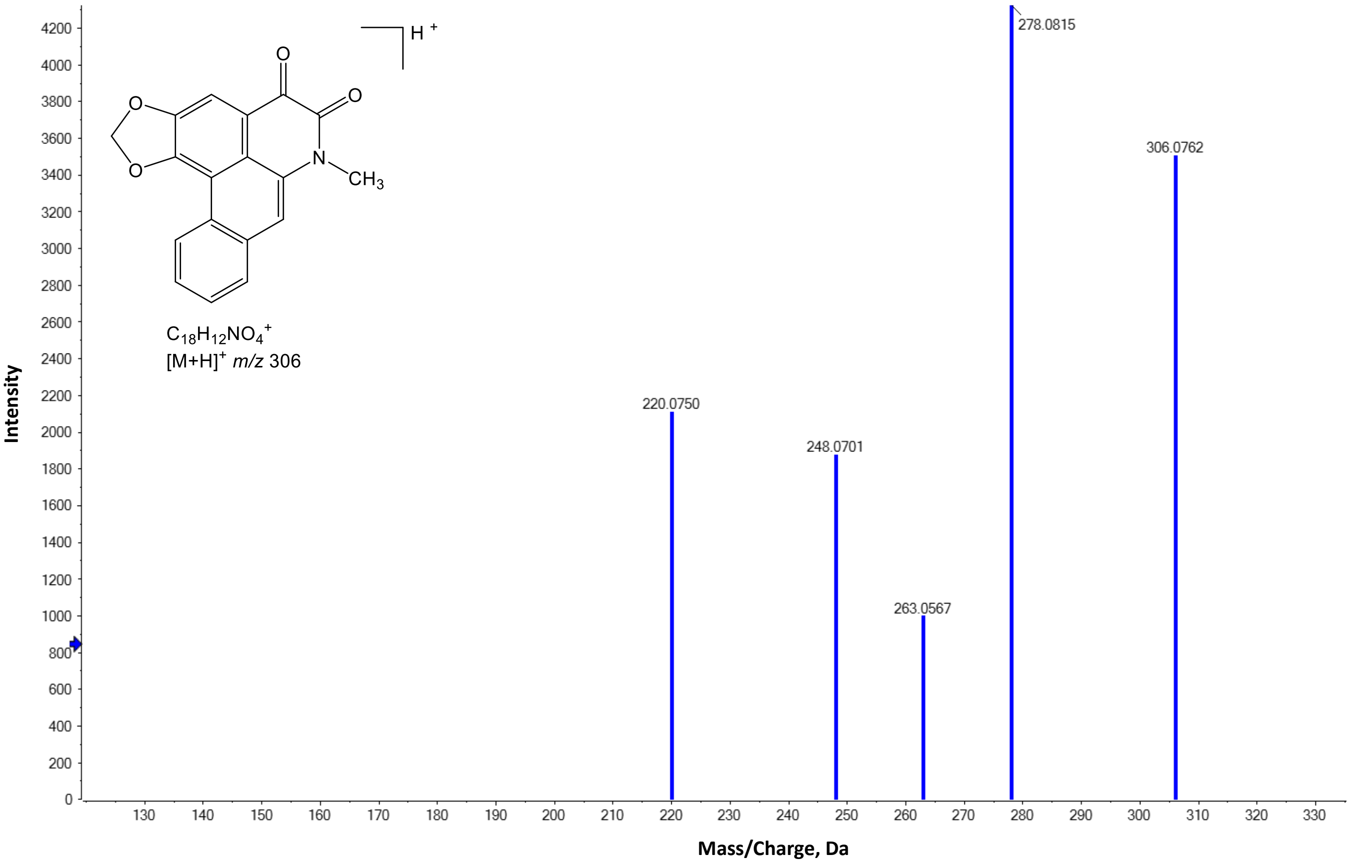

Figure S10_81: MS/MS spectrum of P81.

P82: $C_{16}H_{18}NO_3^+$, isomer of piperyline (formula see **P80**)

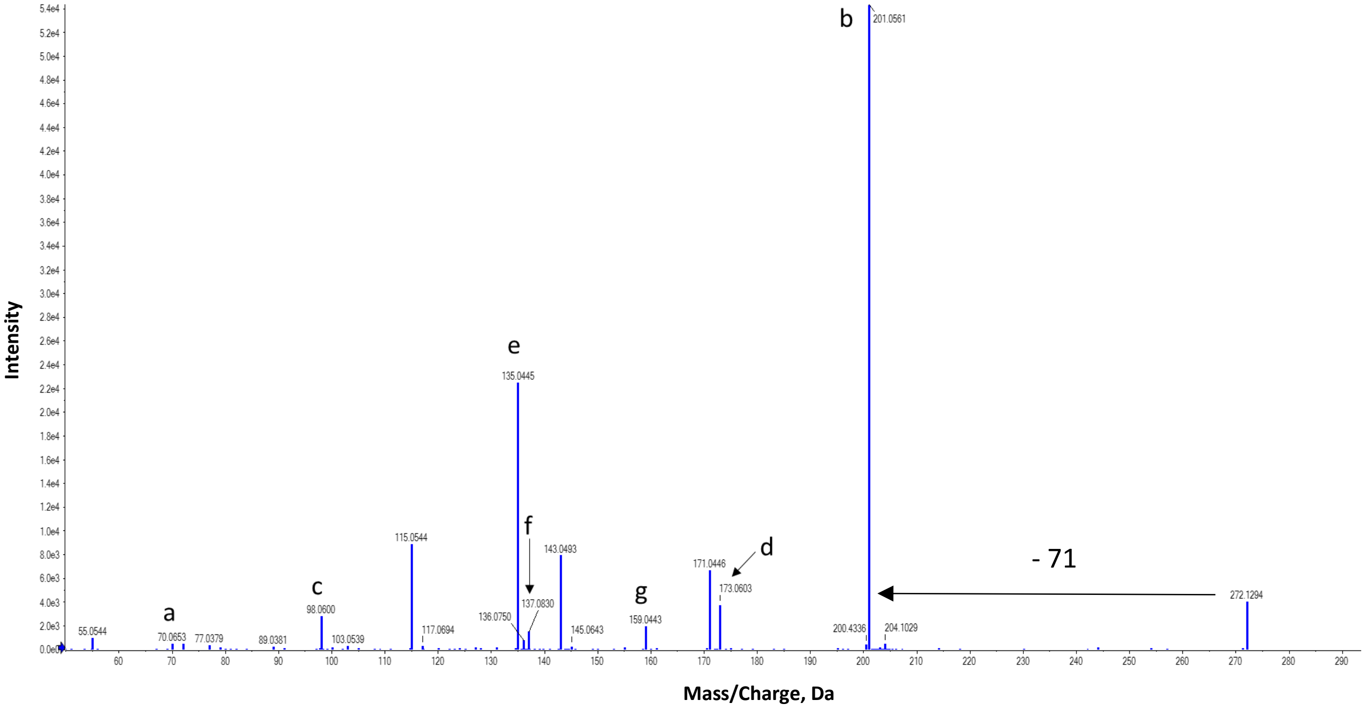

Figure S10_82: MS/MS spectrum of P82.

P83: $C_{18}H_{14}NO_5^+$, isomer of cepharanone D (formula see **P77**)

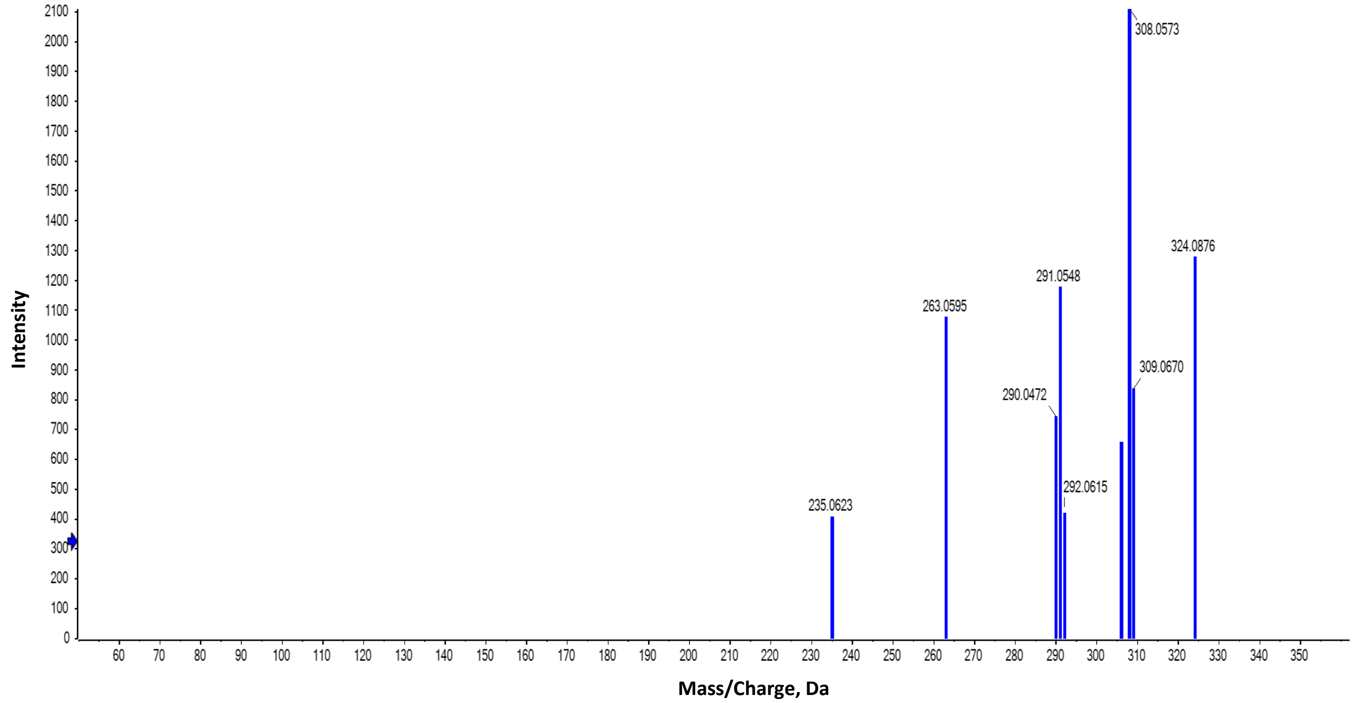

Figure S10_83: MS/MS spectrum of P83.

P84: $C_{16}H_{18}NO_3^+$, isomer of piperyline (formula see **P80**)

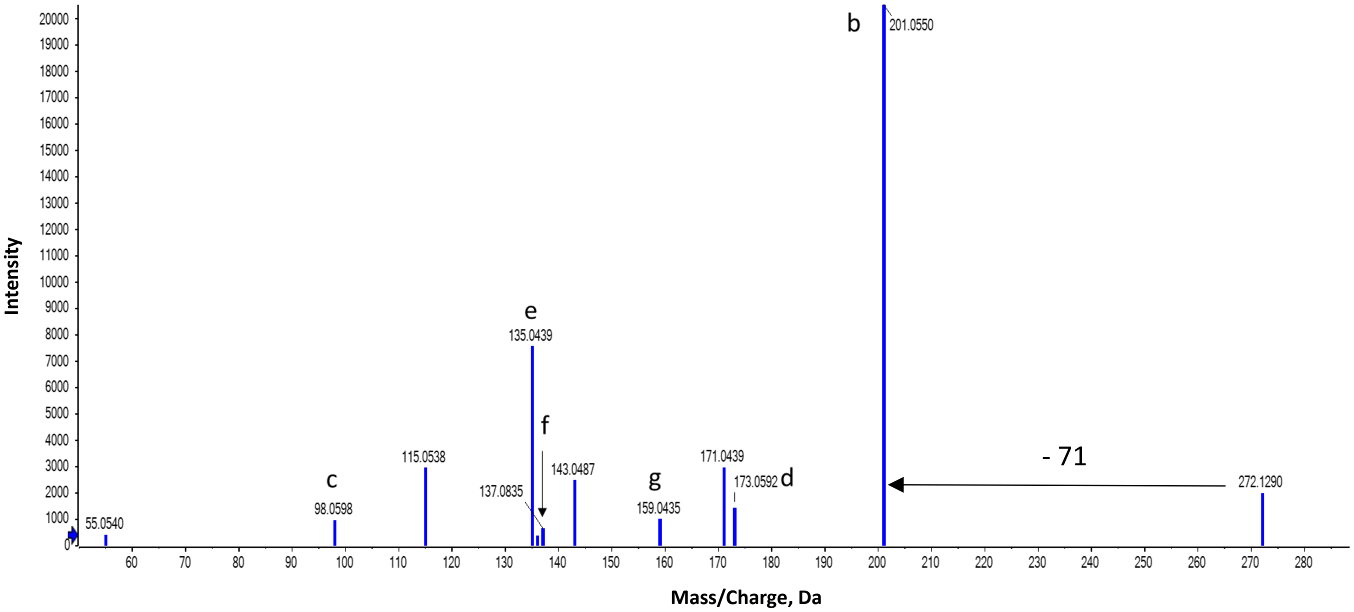

Figure S10_84: MS/MS spectrum of P84.

P85, Methyl ferulate

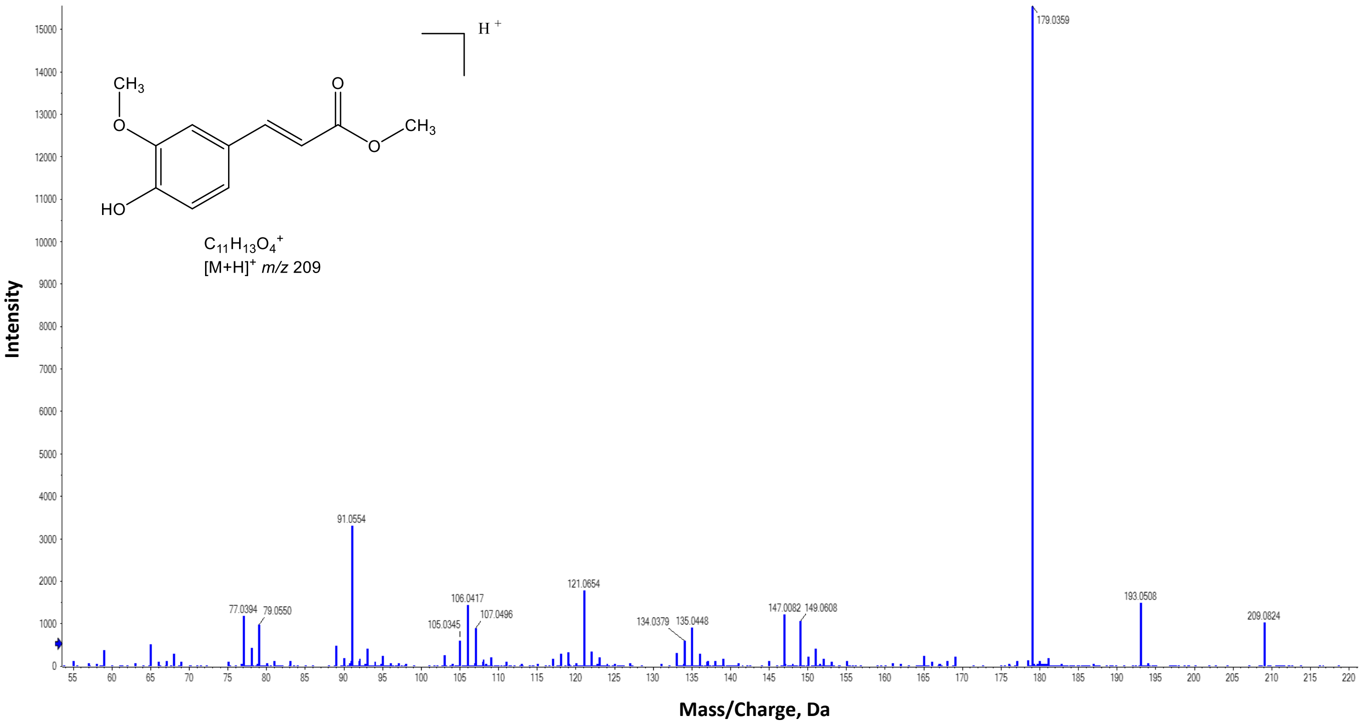

Figure S10_85: MS/MS spectrum of P85.

P86, Acacetin

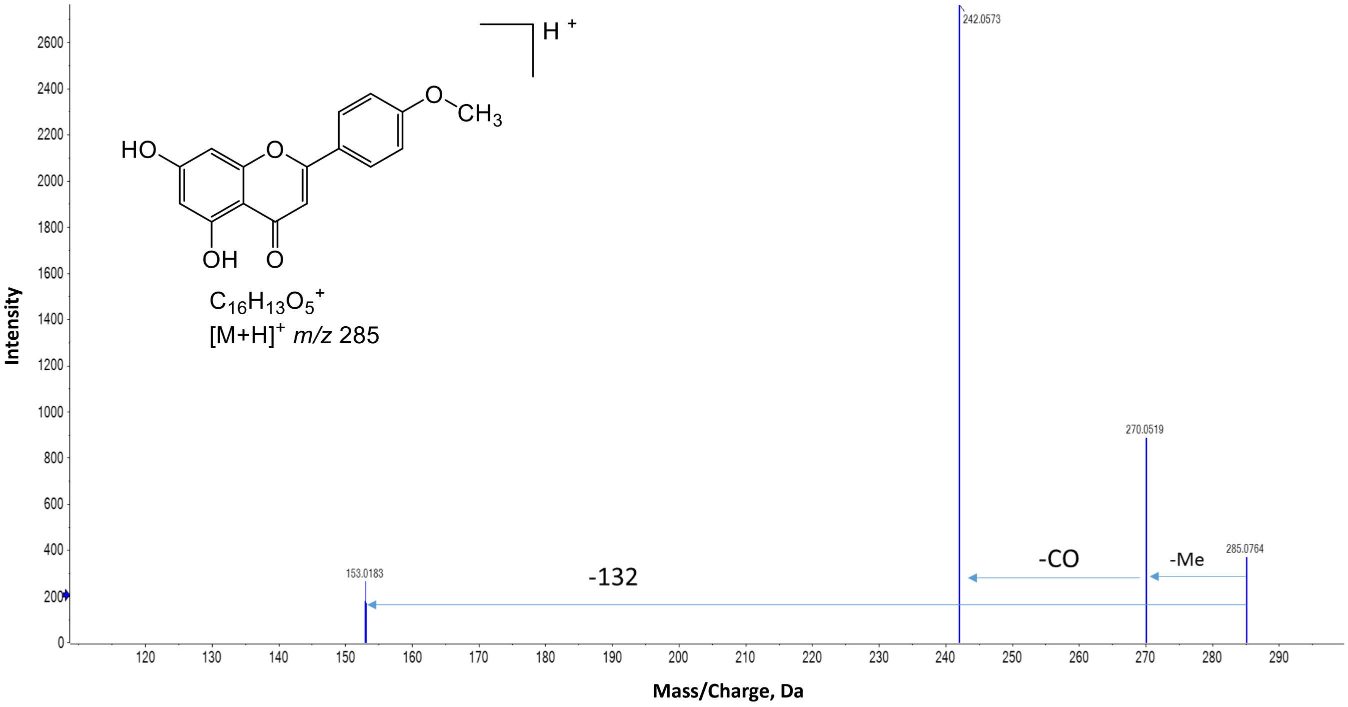

Figure S10_86: MS/MS spectrum of P86.

P87, Aristolactam BII*

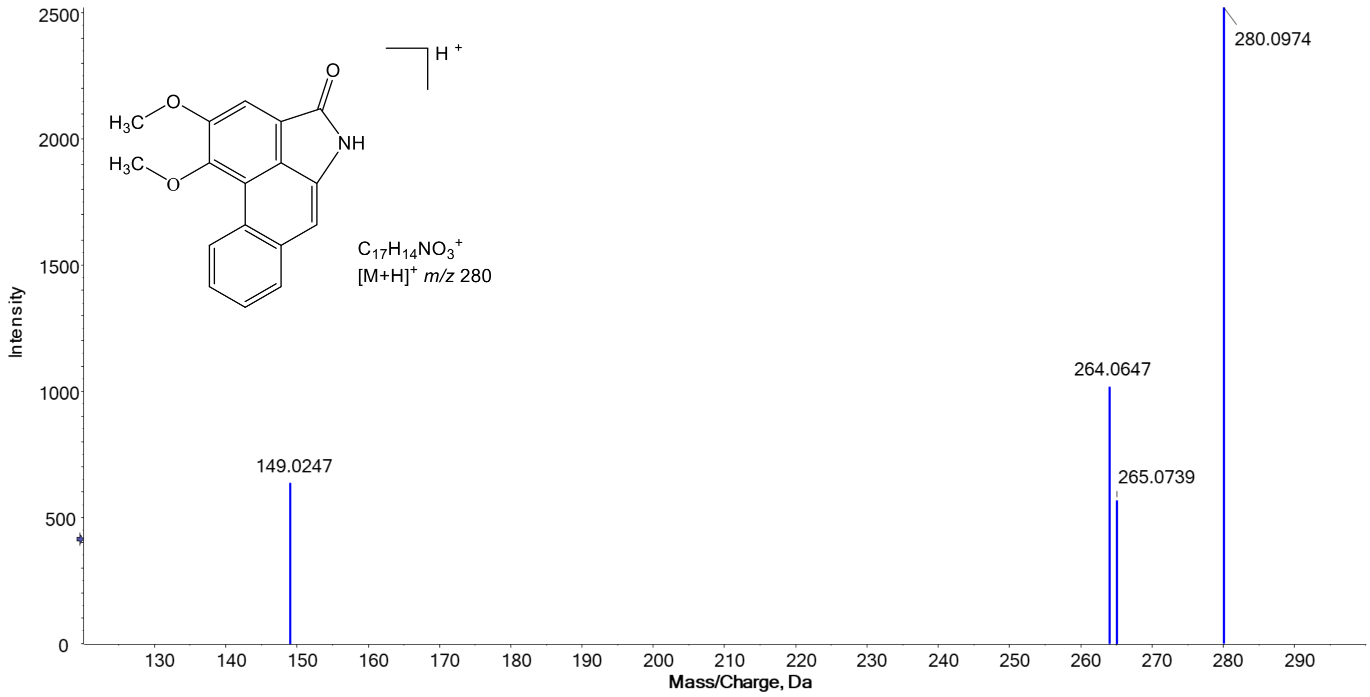

Figure S10_87: MS/MS spectrum of P87.

P88, *N*-Formylnornuciferin

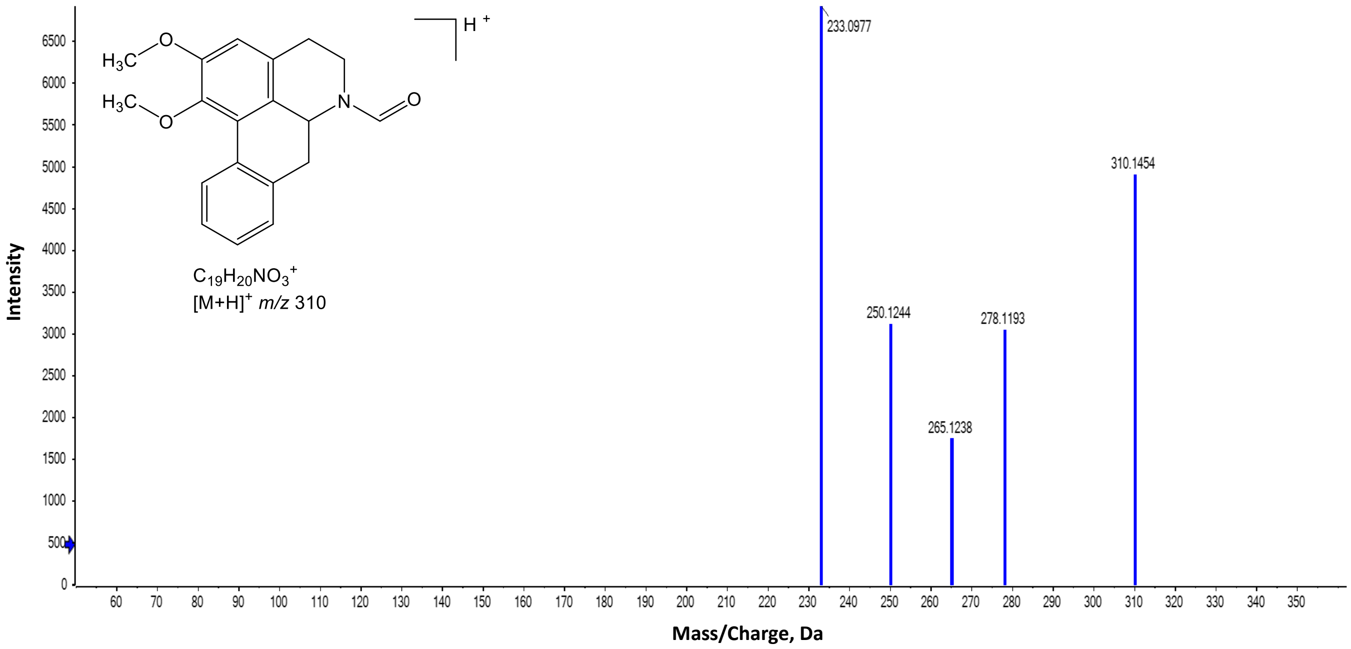

Figure S10_88: MS/MS spectrum of P88.

P89: $C_{12}H_{17}O_3^+$, isomer of *trans*-asarone (formula see **P95**)

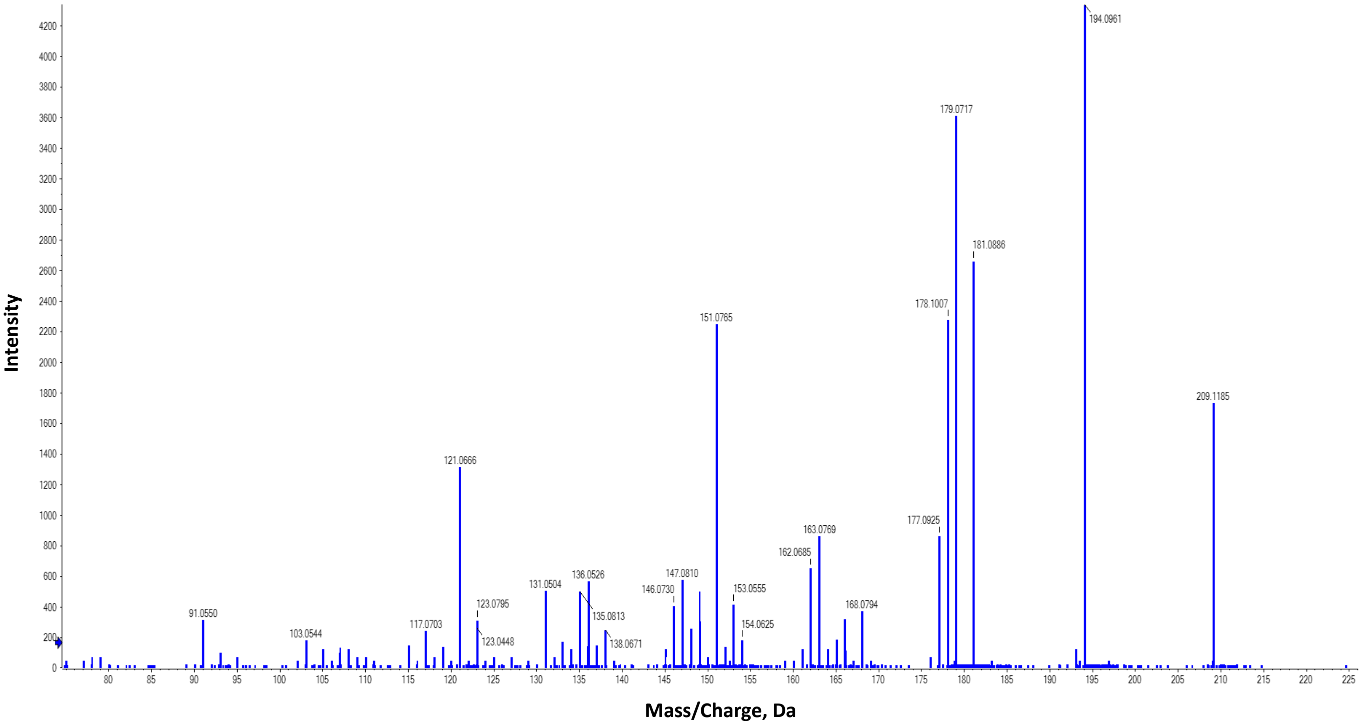

Figure S10_89: MS/MS spectrum of P89.

P90, Eudesmin

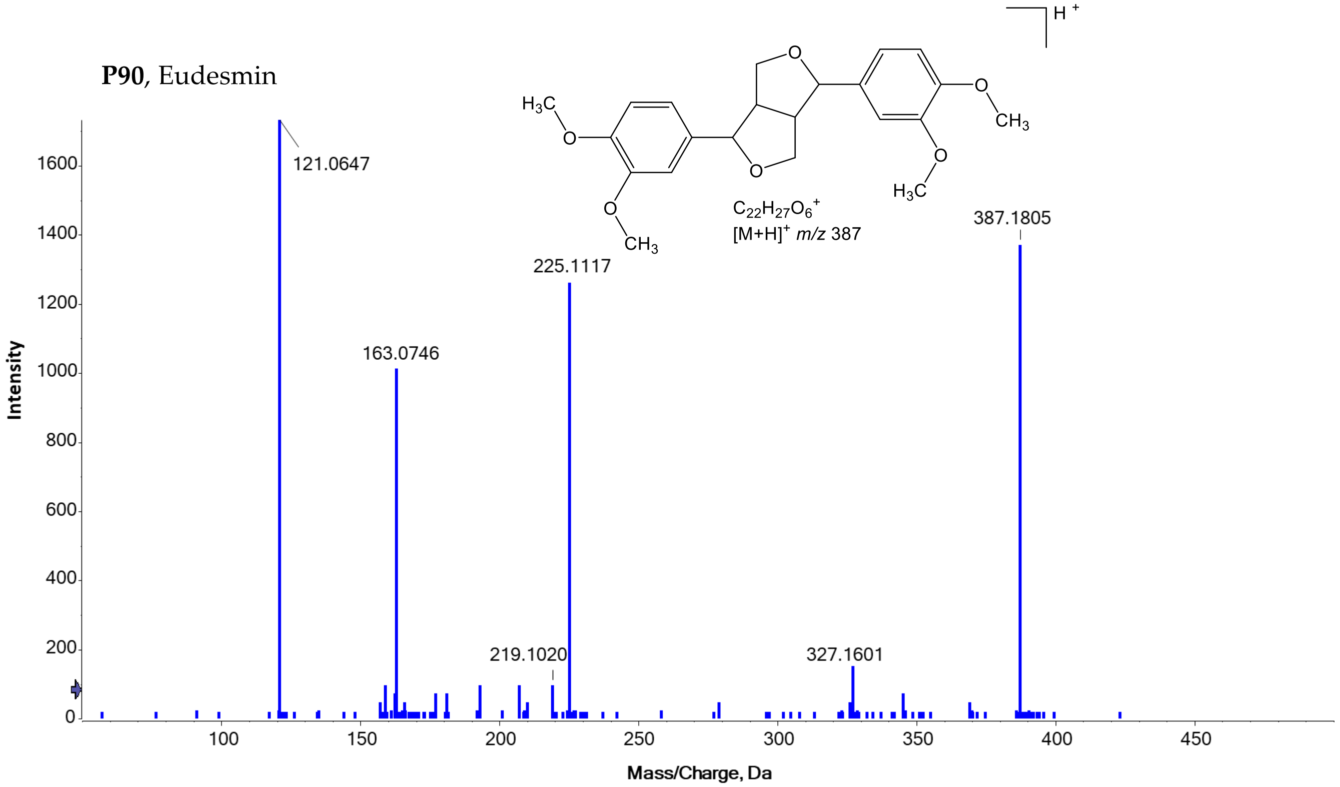

Figure S10_90: MS/MS spectrum of P90.

P91, Piperine

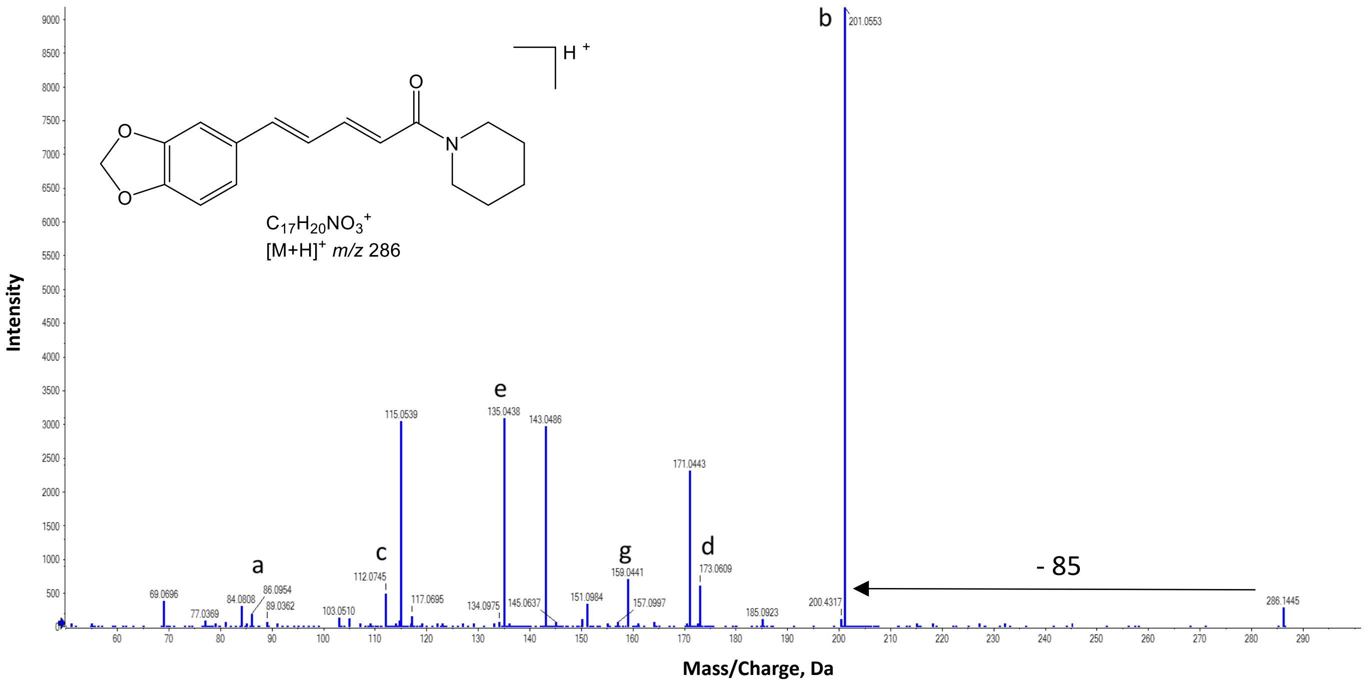

Figure S10_91: MS/MS spectrum of P91.

P92, Piperettyline

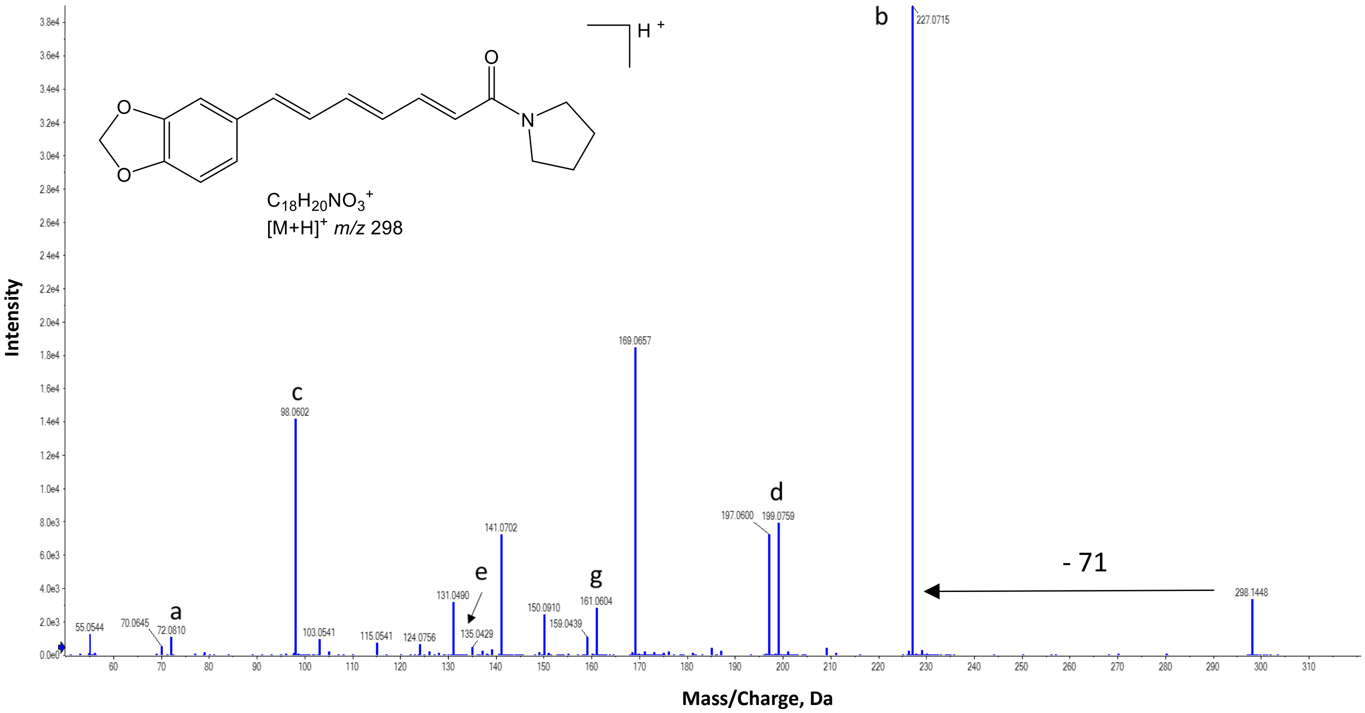

Figure S10_92: MS/MS spectrum of P92.

P93, Nigrinodine

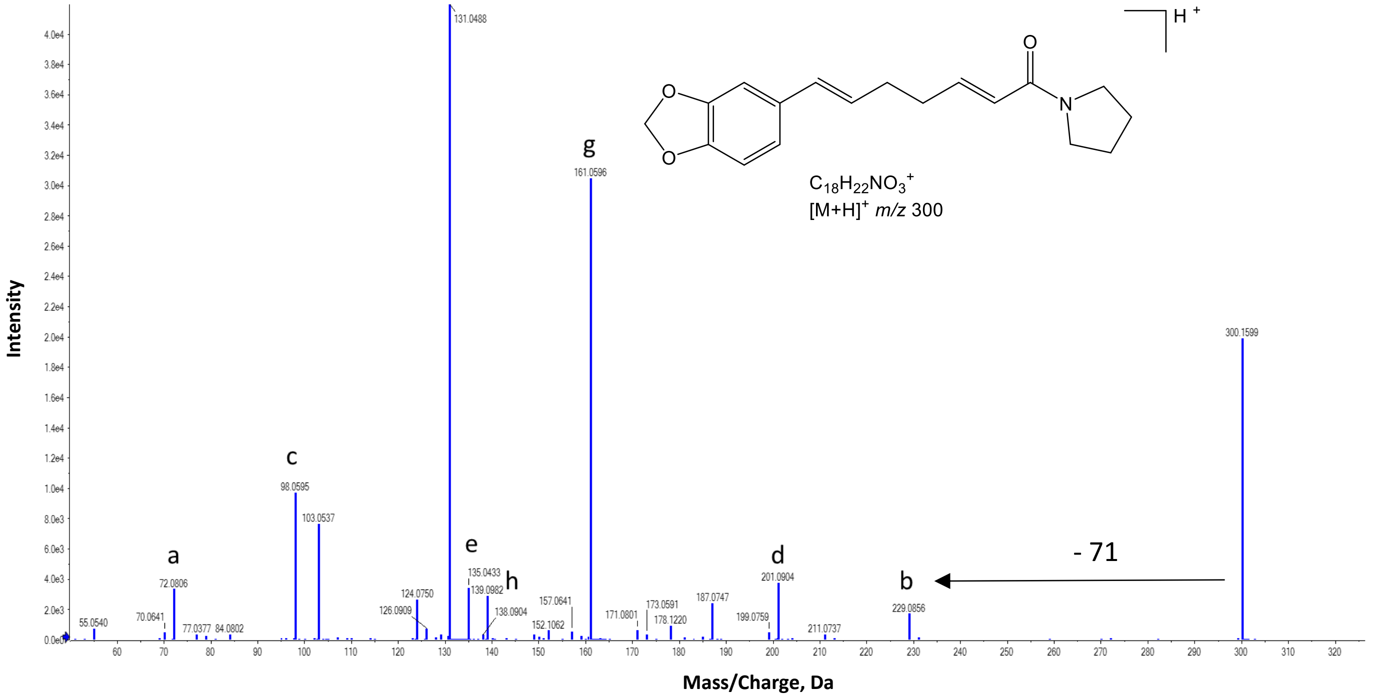

Figure S10_93: MS/MS spectrum of P93.

P94, Isoasarone*

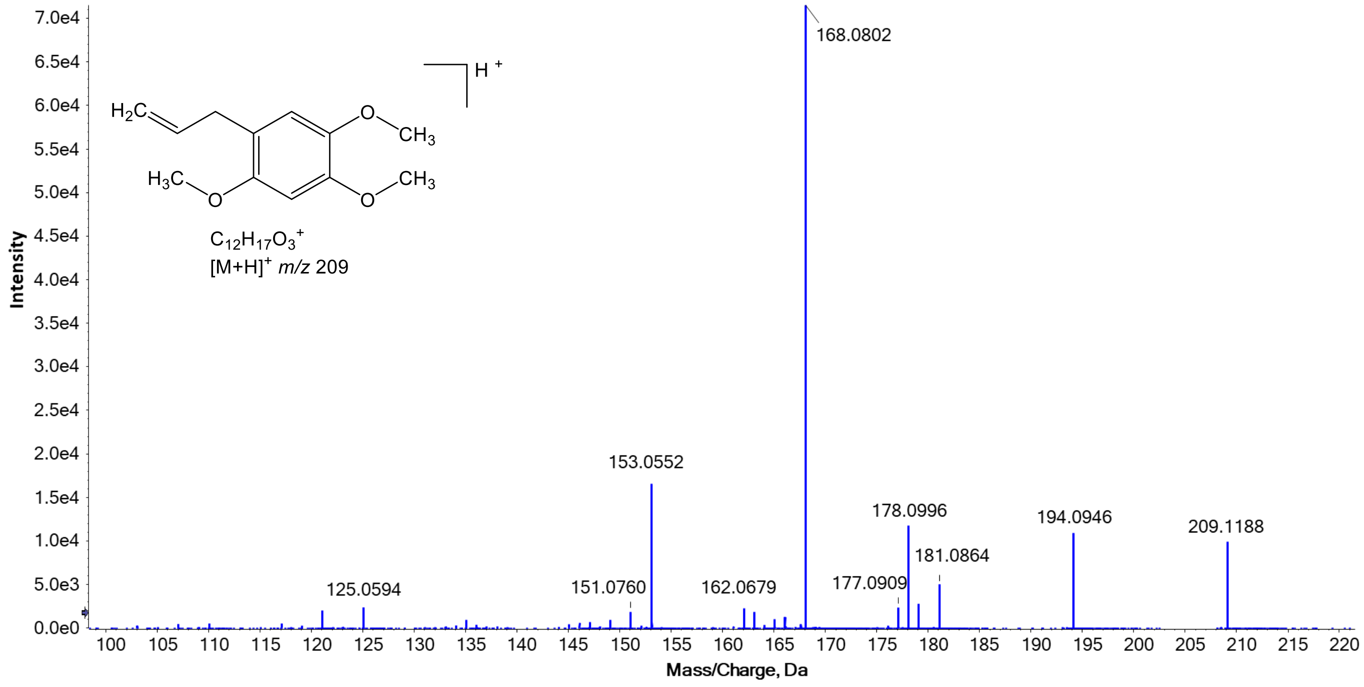

Figure S10_94: MS/MS spectrum of P94.

P95, *trans*-Asarone*

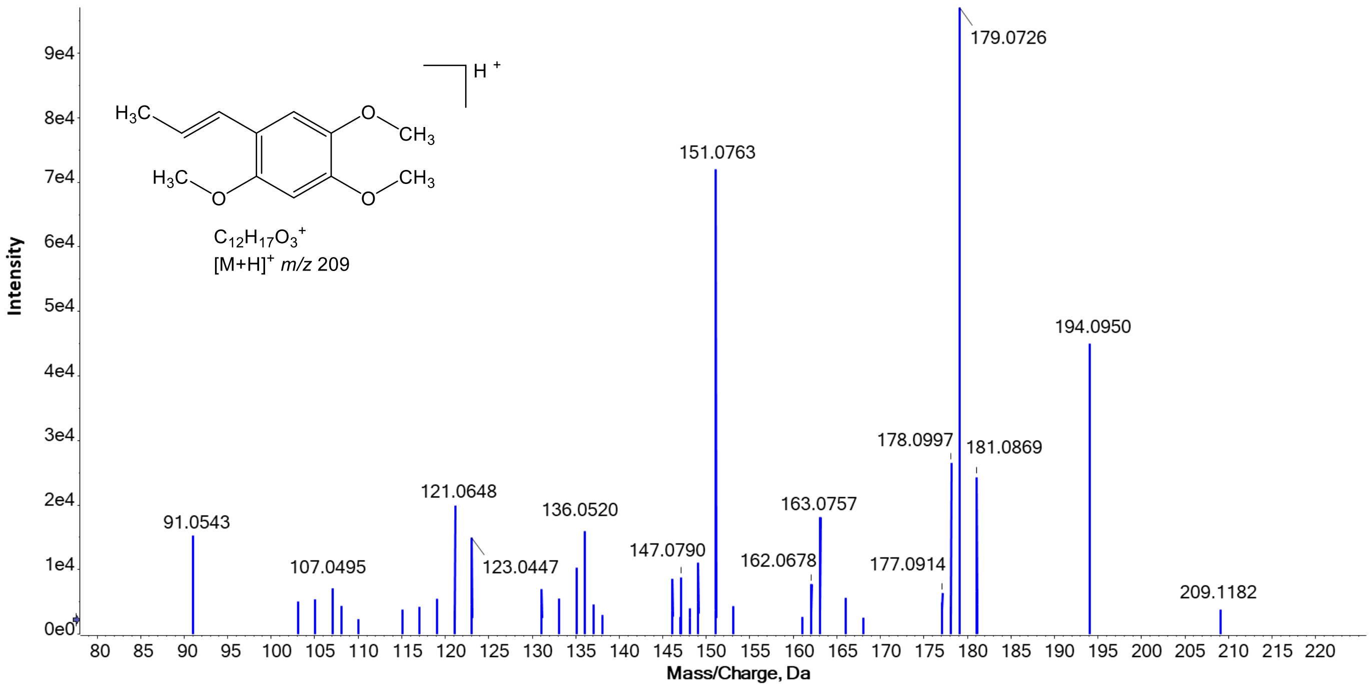

Figure S10_95: MS/MS spectrum of P95.

P96, Grandisin

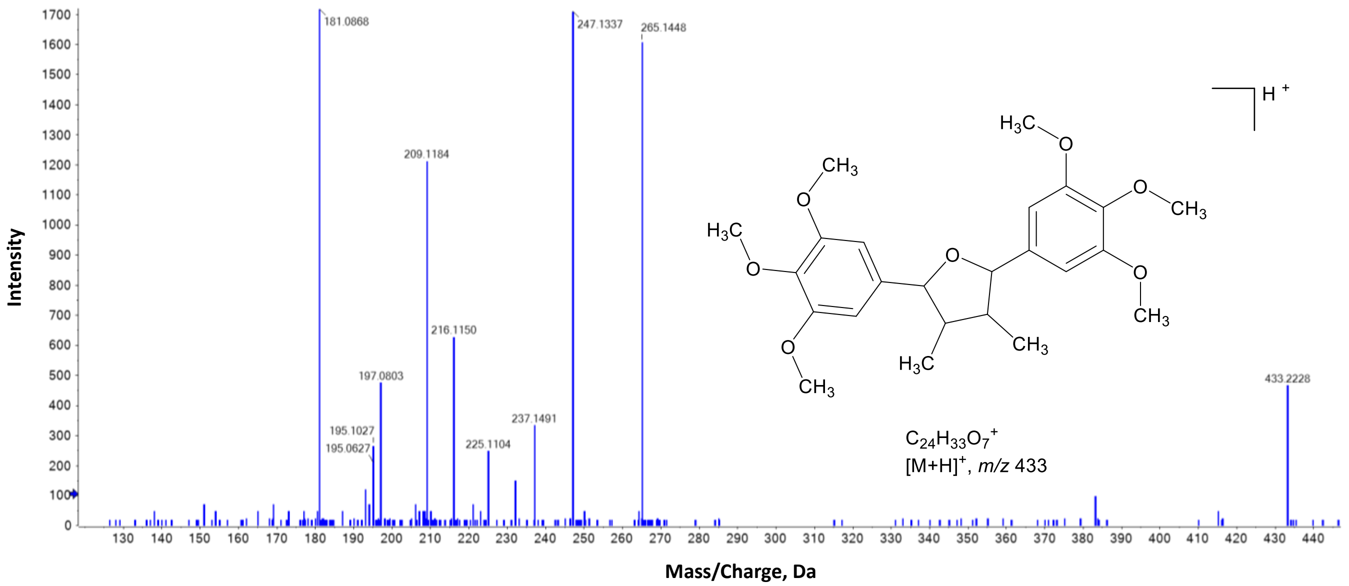

Figure S10_96: MS/MS spectrum of P96.

P97, Aristolactam BIII

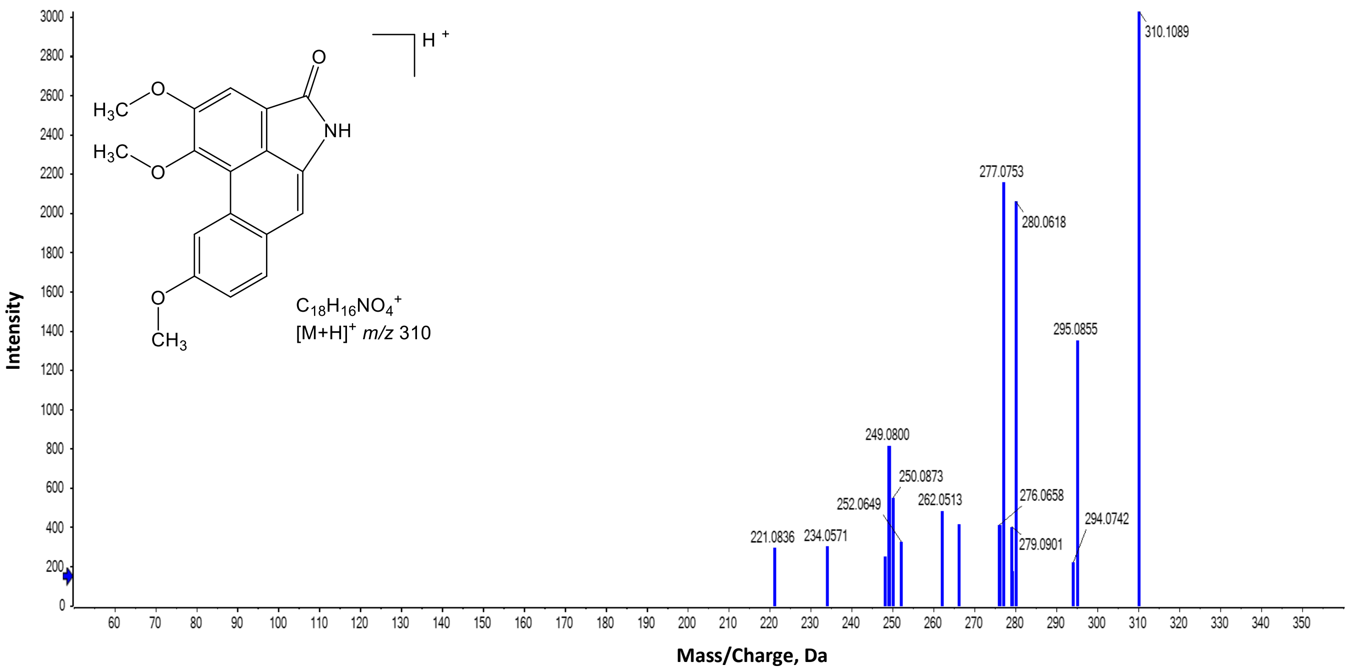

Figure S10_97: MS/MS spectrum of P97.

P98: $C_{18}H_{20}NO_3^+$, isomer of piperettyline (formula see **P92**)

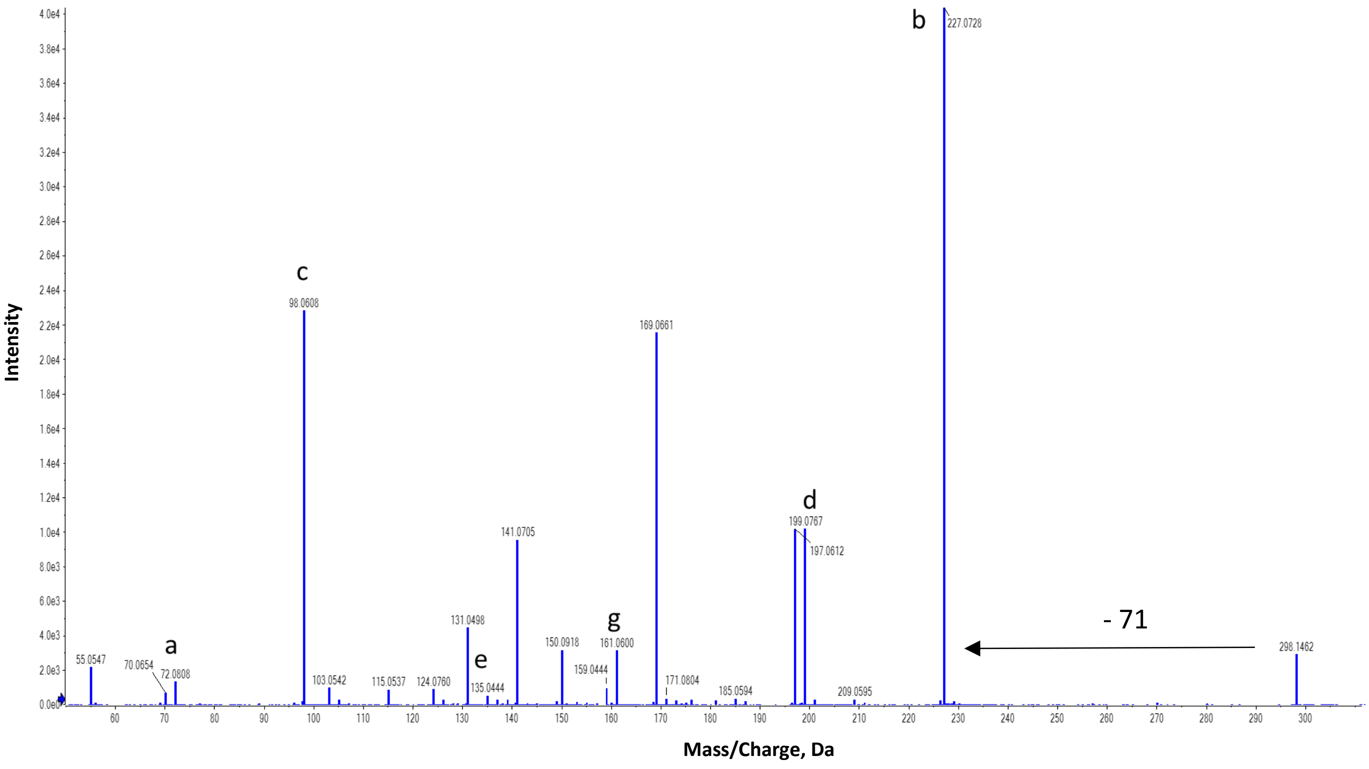

Figure S10_98: MS/MS spectrum of P98.

P99, Dehydronorglaucine

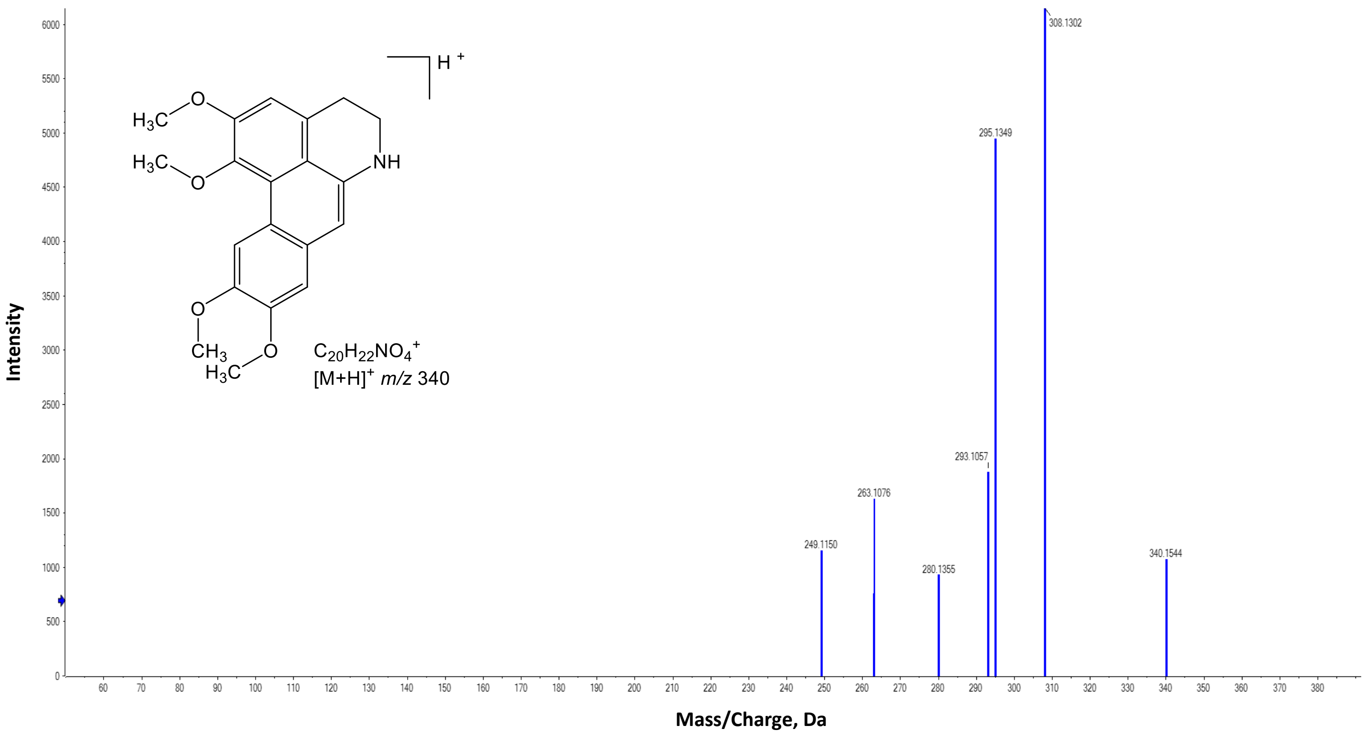

Figure S10_99: MS/MS spectrum of P99.

P100, *N*-Demethyl-*N*-formyldehydronuciferine

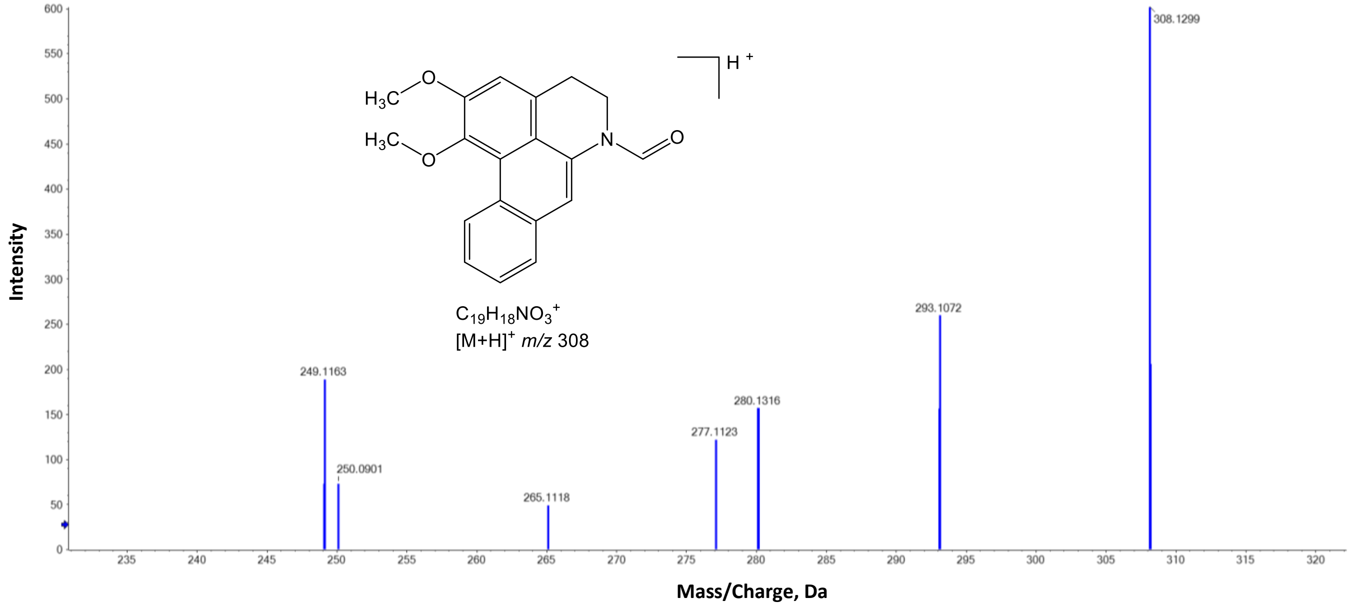

Figure S10_100: MS/MS spectrum of P100.

P101, Piperamide-C7:1

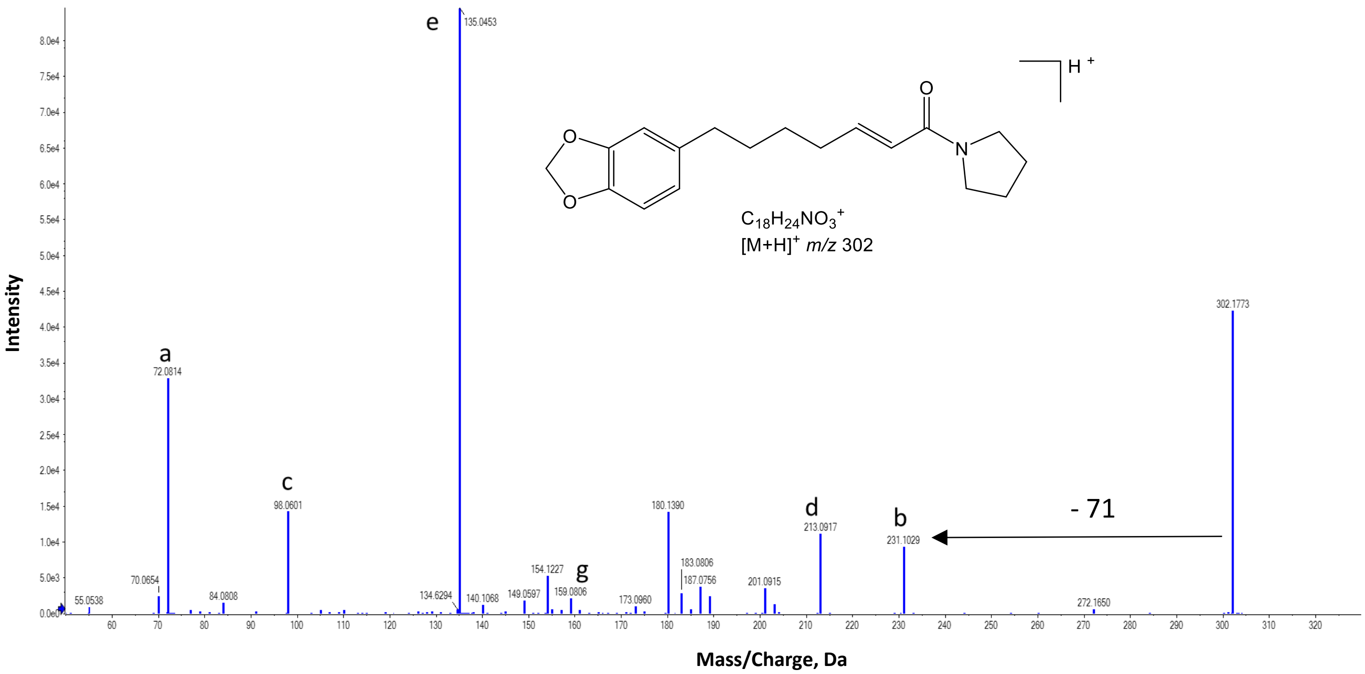

Figure S10_101: MS/MS spectrum of P101.

P102: $C_{18}H_{20}NO_3^+$, isomer of piperettyline (formula see **P92**)

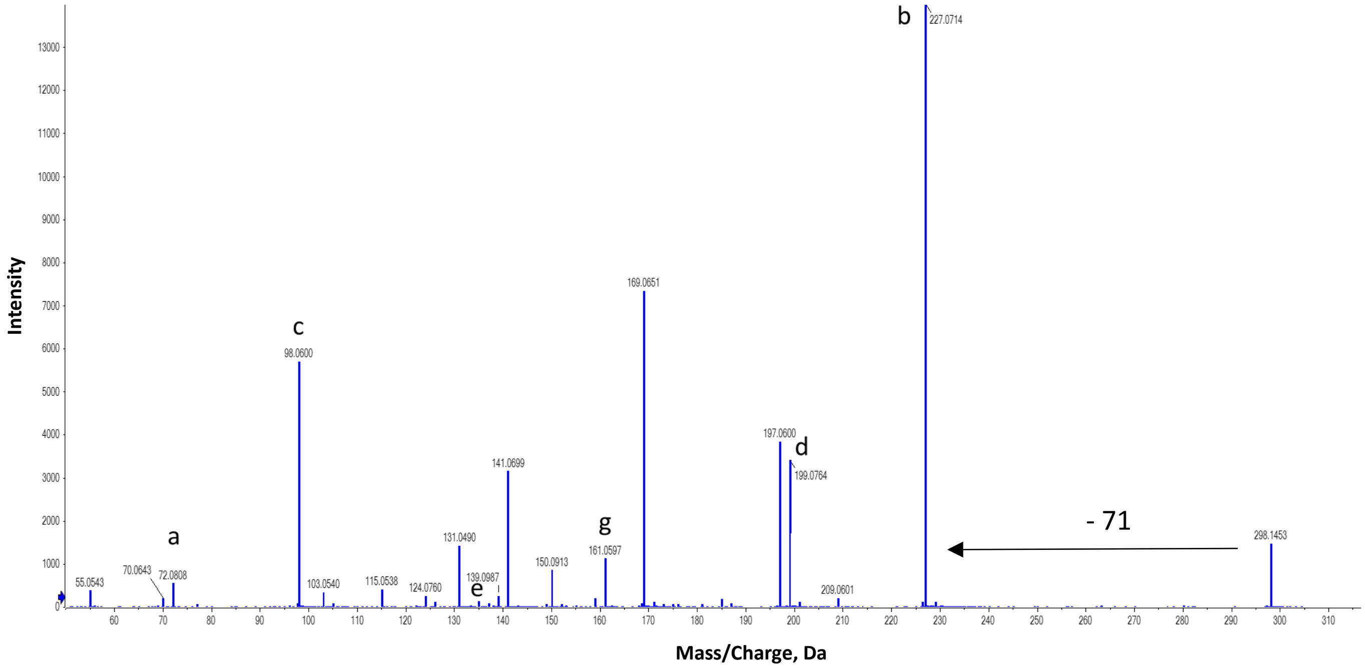

Figure S10_102: MS/MS spectrum of P102.

P103: $C_{18}H_{20}NO_3^+$, isomer of *N*-Demethyl-*N*-formyldehydronuciferine (formula see **P100**)

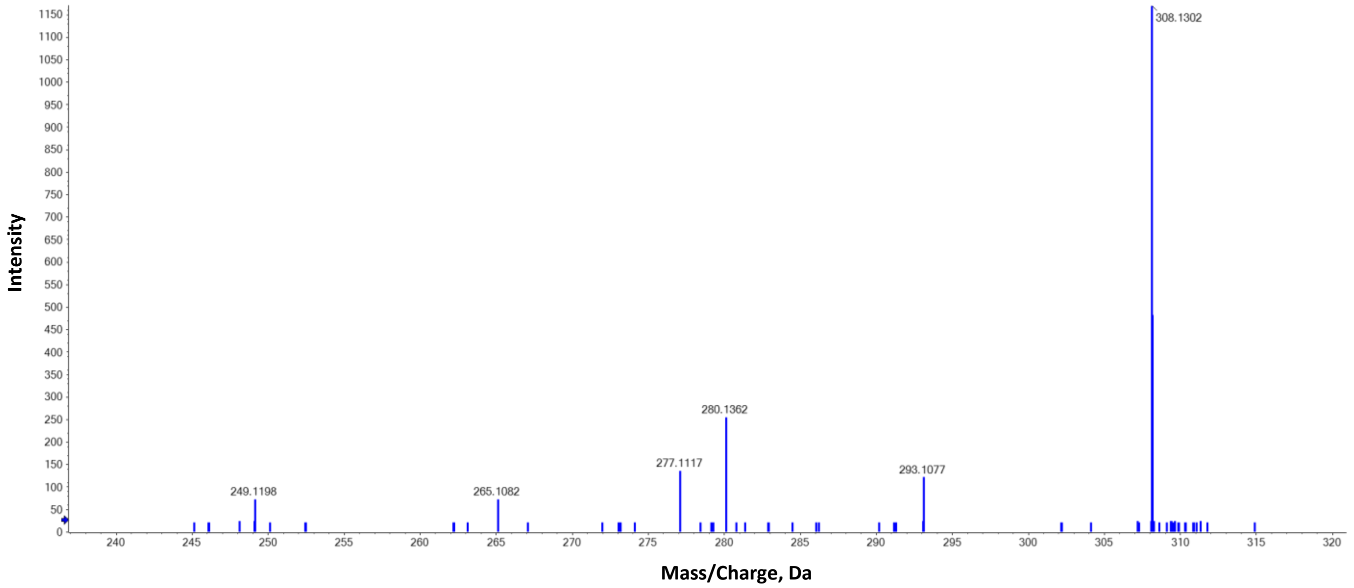

Figure S10_103: MS/MS spectrum of P103.

P104, 2,4-decadienoic acid p-hydroxyphenethylamide

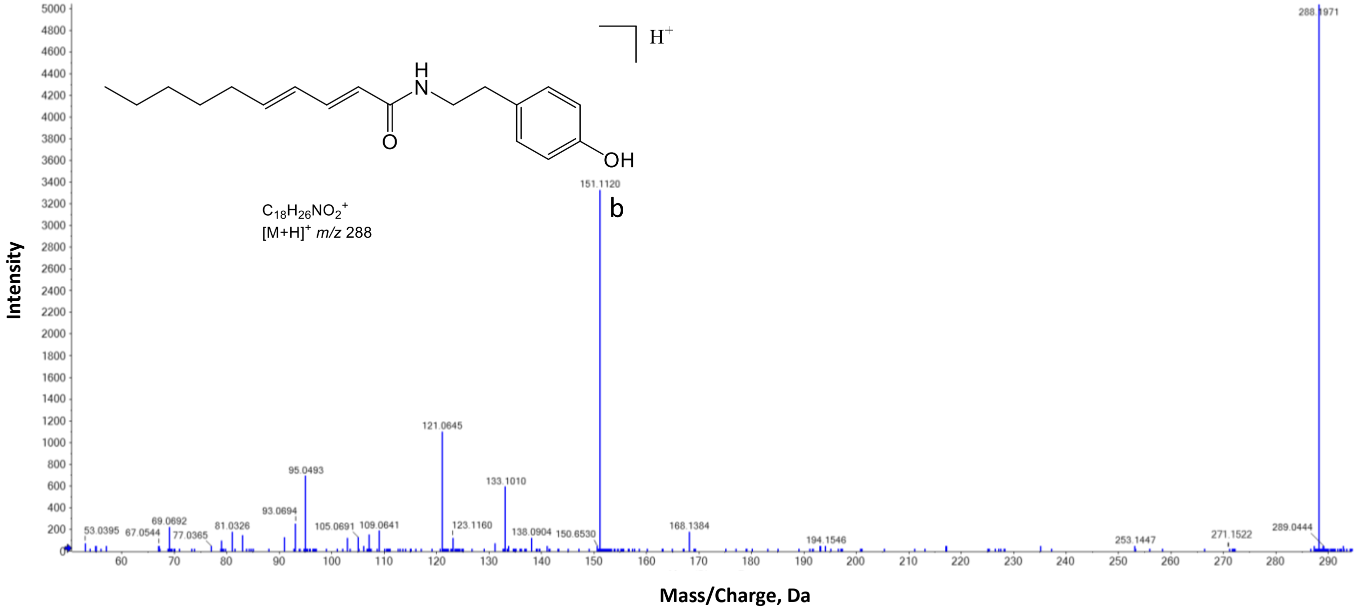

Figure S10_104: MS/MS spectrum of P104.

P105, Chingchengenamide A

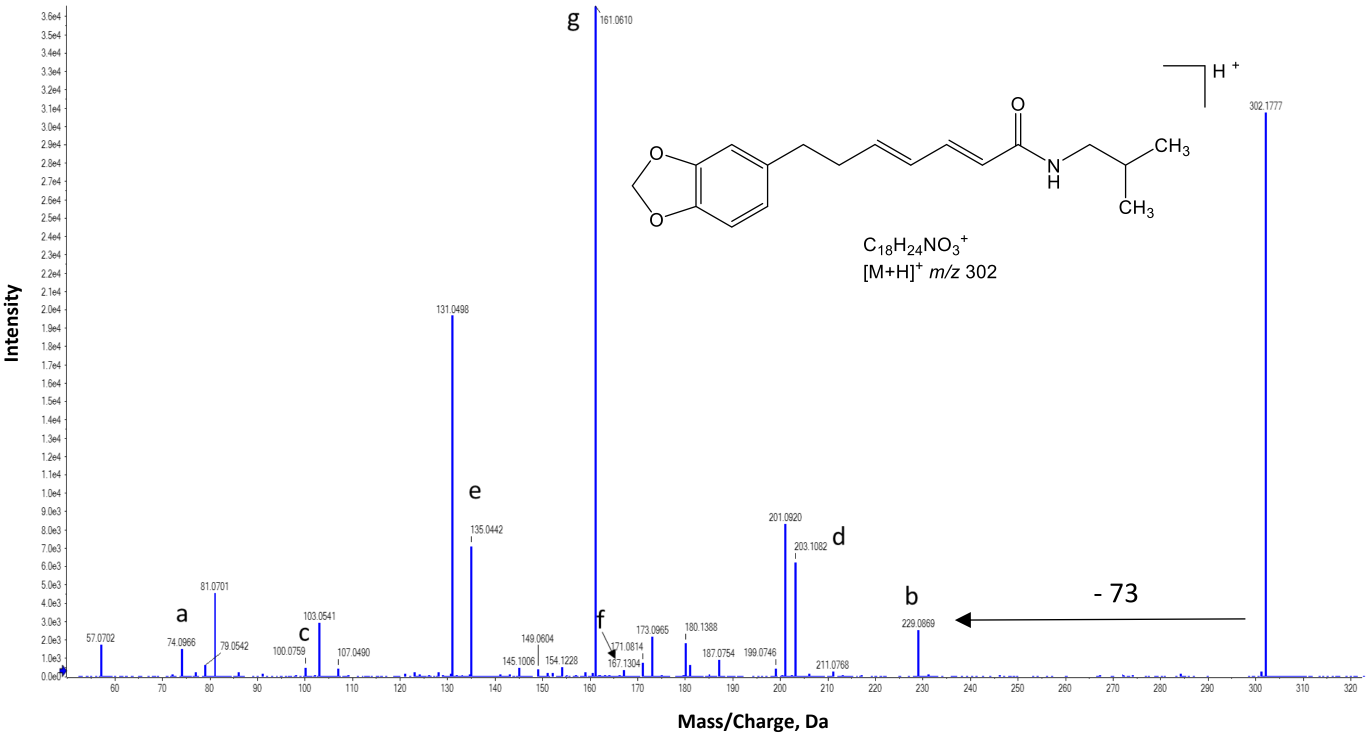

Figure S10_105: MS/MS spectrum of P105.

P106, 1-(Pyrrolidinyl)-2,4,6-decatrieneone

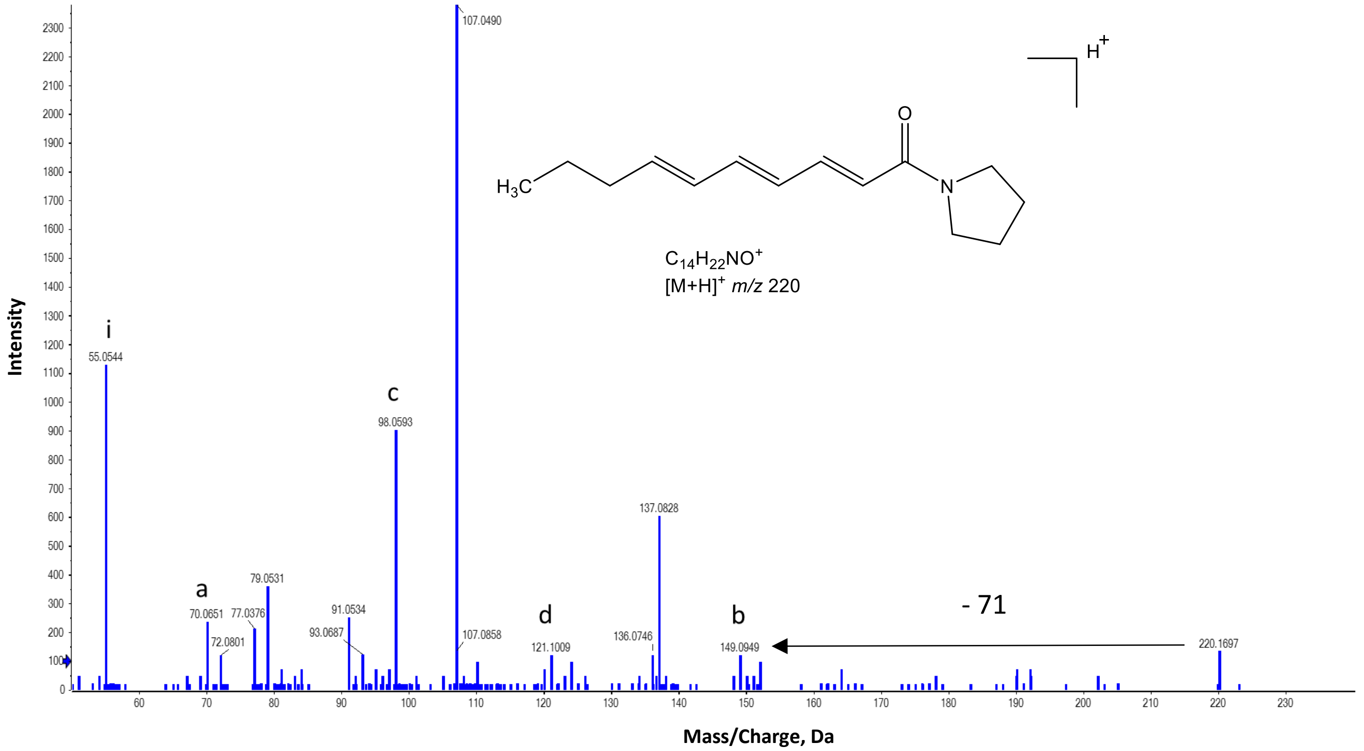

Figure S10_106: MS/MS spectrum of P106.

P107, 3,4-Dehydrofutoamide

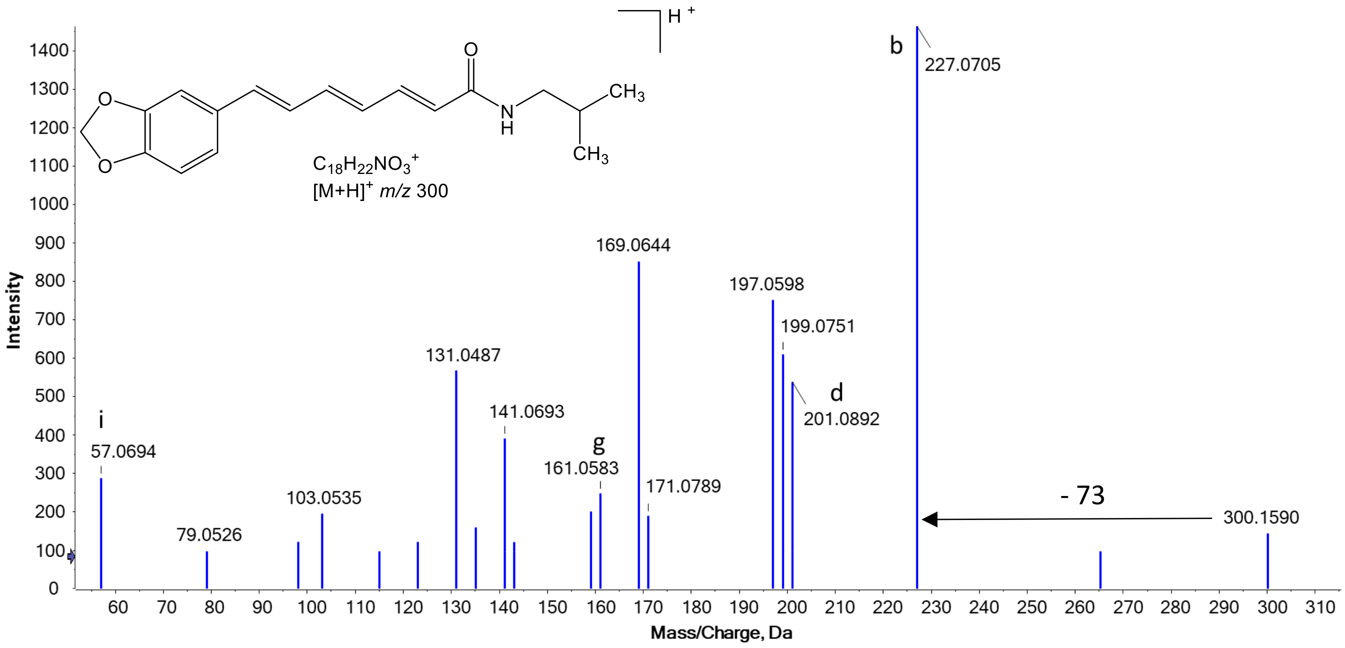

Figure S10_107: MS/MS spectrum of P107.

P108, Piperdardine

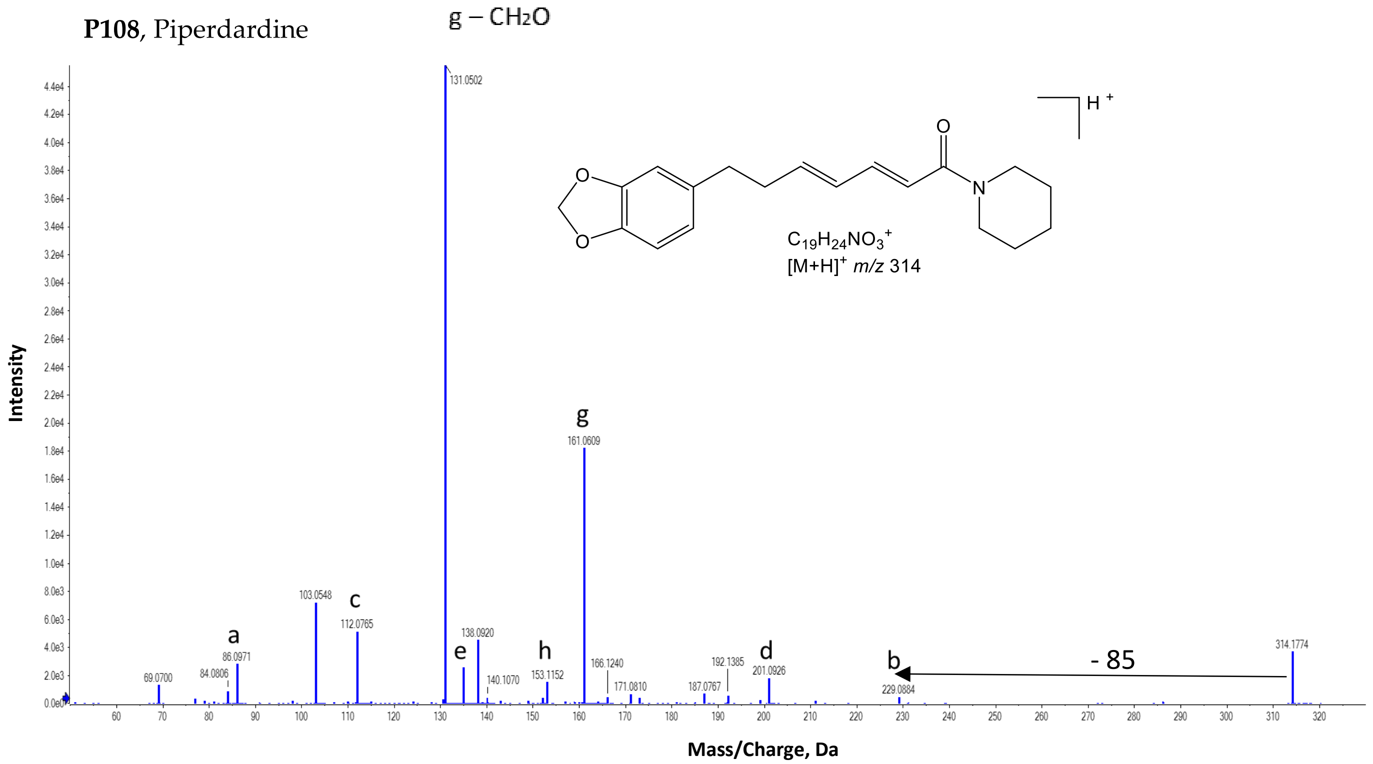

Figure S10_108: MS/MS spectrum of P108.

P109, Piperettine

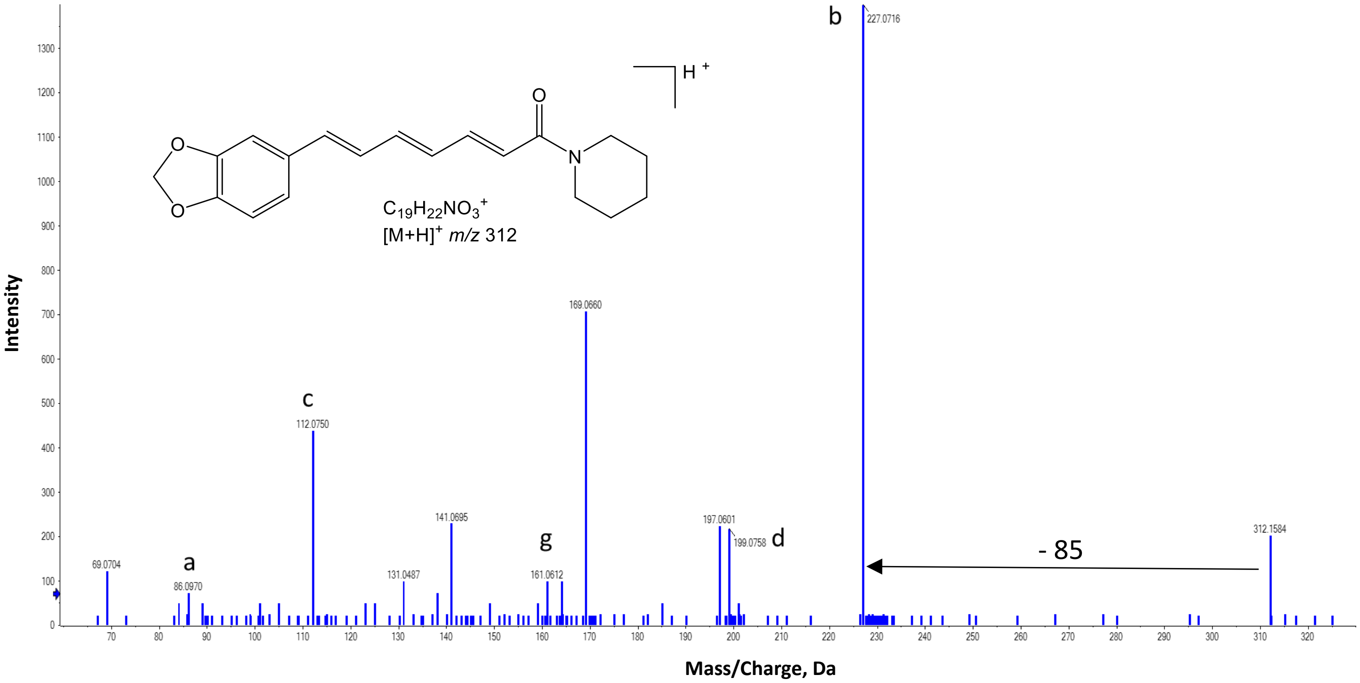

Figure S10_109: MS/MS spectrum of P109.

P110, 6,7-Dehydrobrachyamide B

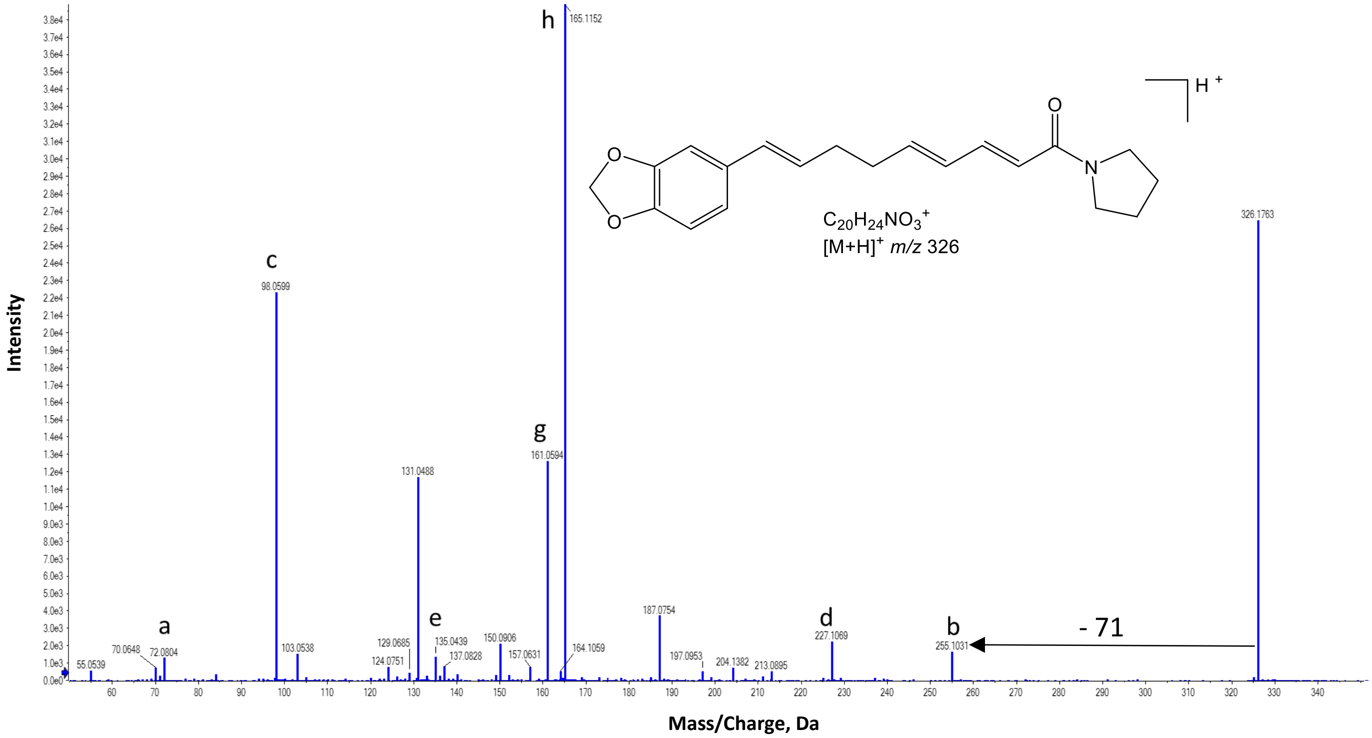

Figure S10_110: MS/MS spectrum of P110.

P111: $C_{19}H_{22}NO_3^+$, isomer of piperettine (formula see **P109**)

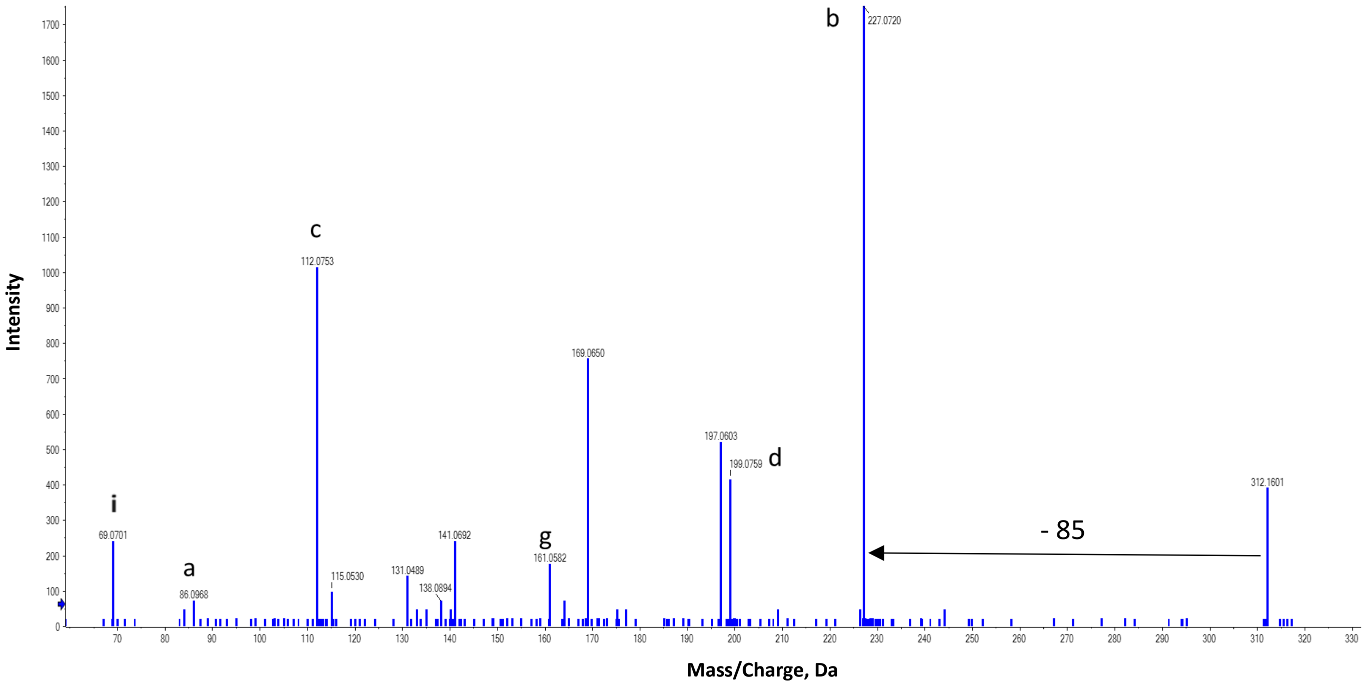

Figure S10_111: MS/MS spectrum of P111.

P112, Sarmentine

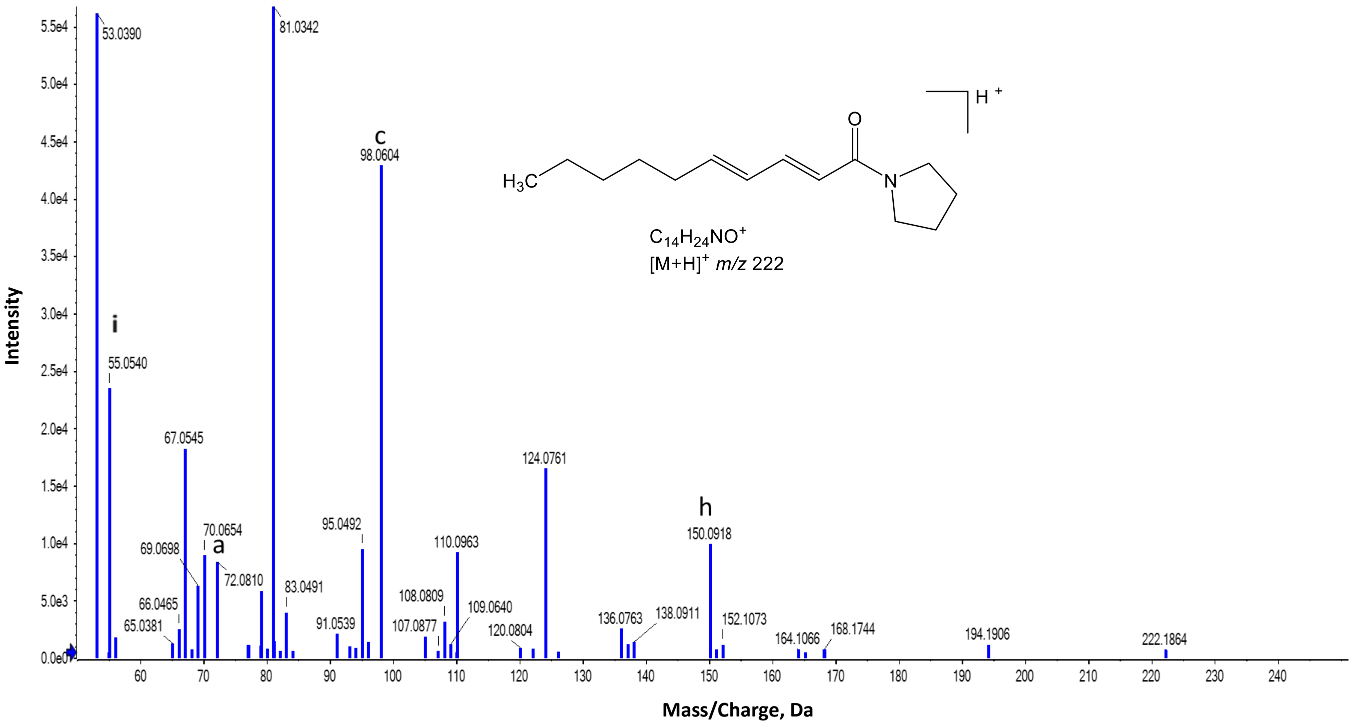

Figure S10_112: MS/MS spectrum of P112.

P113, Dehydroformouregine*

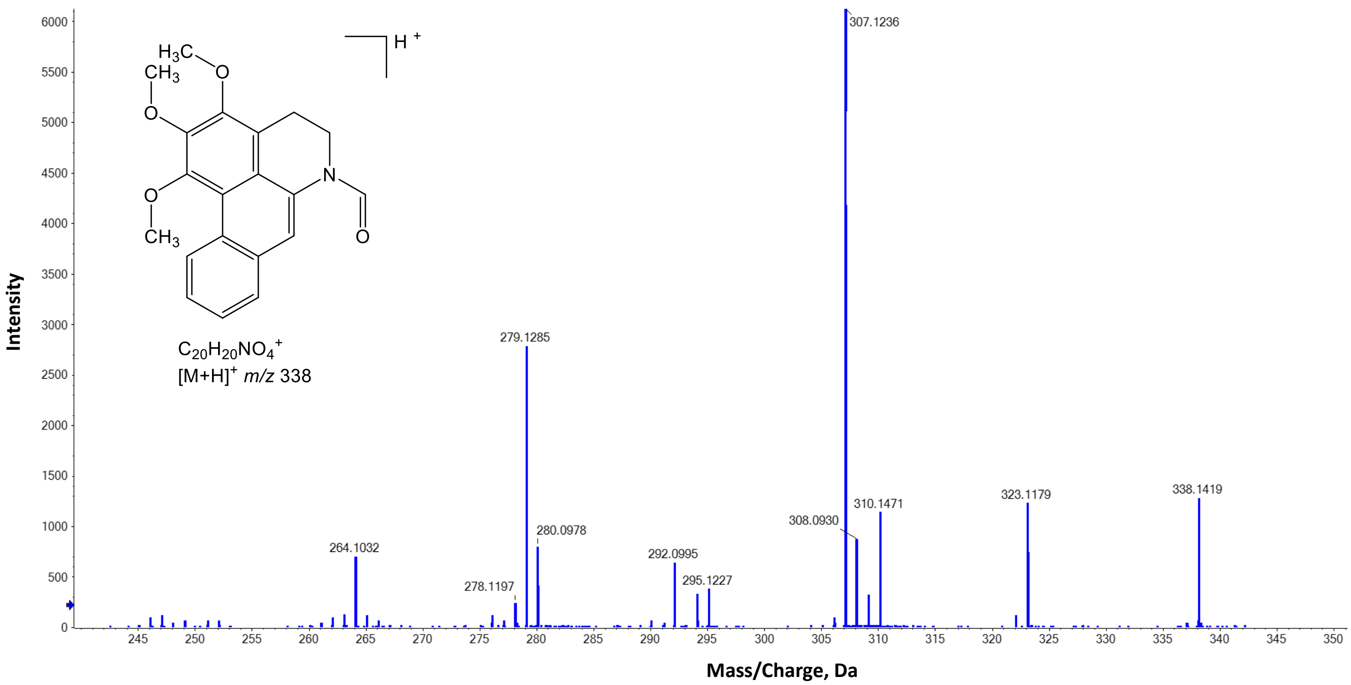

Figure S10_113: MS/MS spectrum of P113.

P114, Brachyamide B

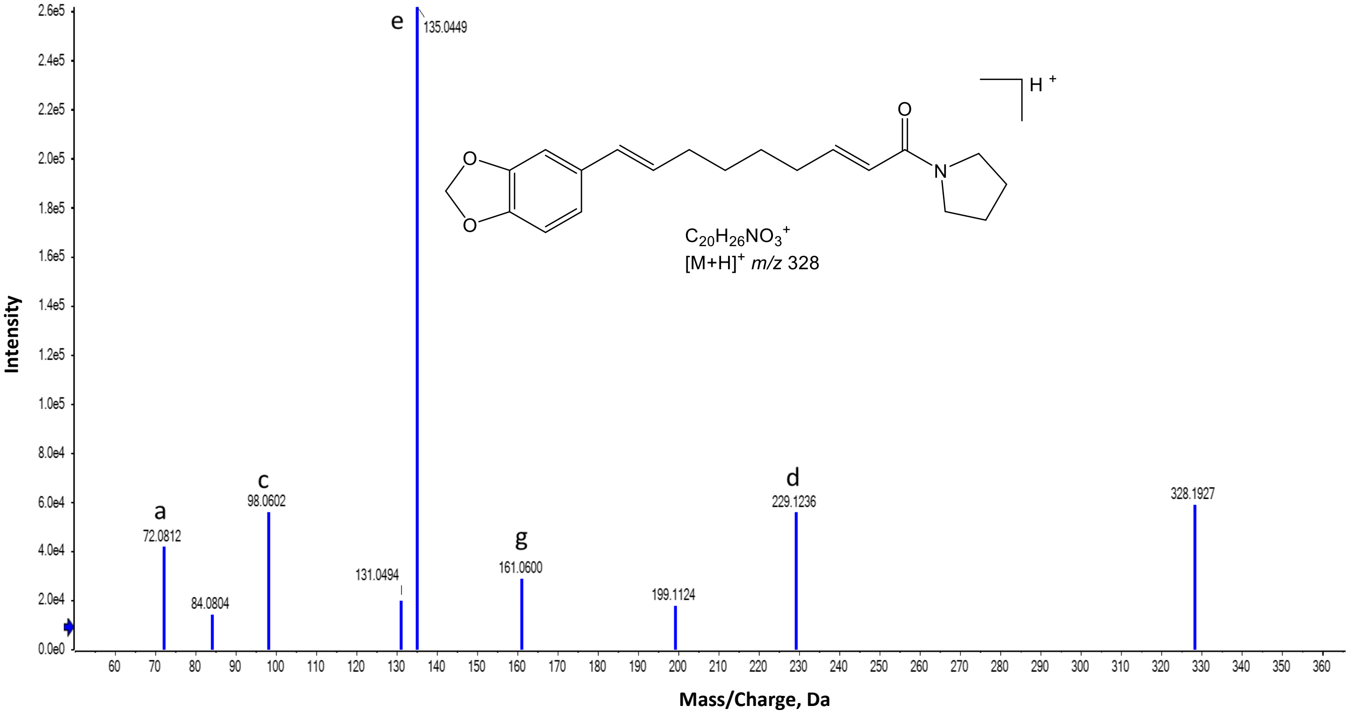

Figure S10_114: MS/MS spectrum of P114.

P115, *7-O,4′-O*-Dimethyl-apigenin*

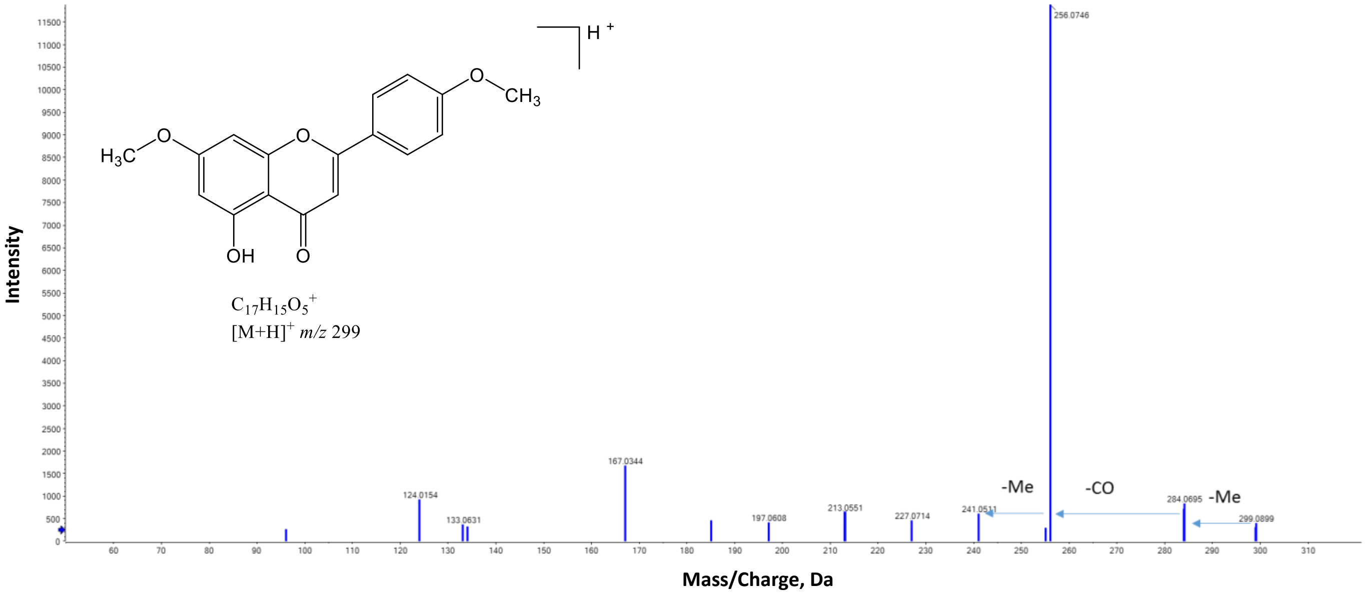

Figure S10_115: MS/MS spectrum of P115.

P116, Pellitorine

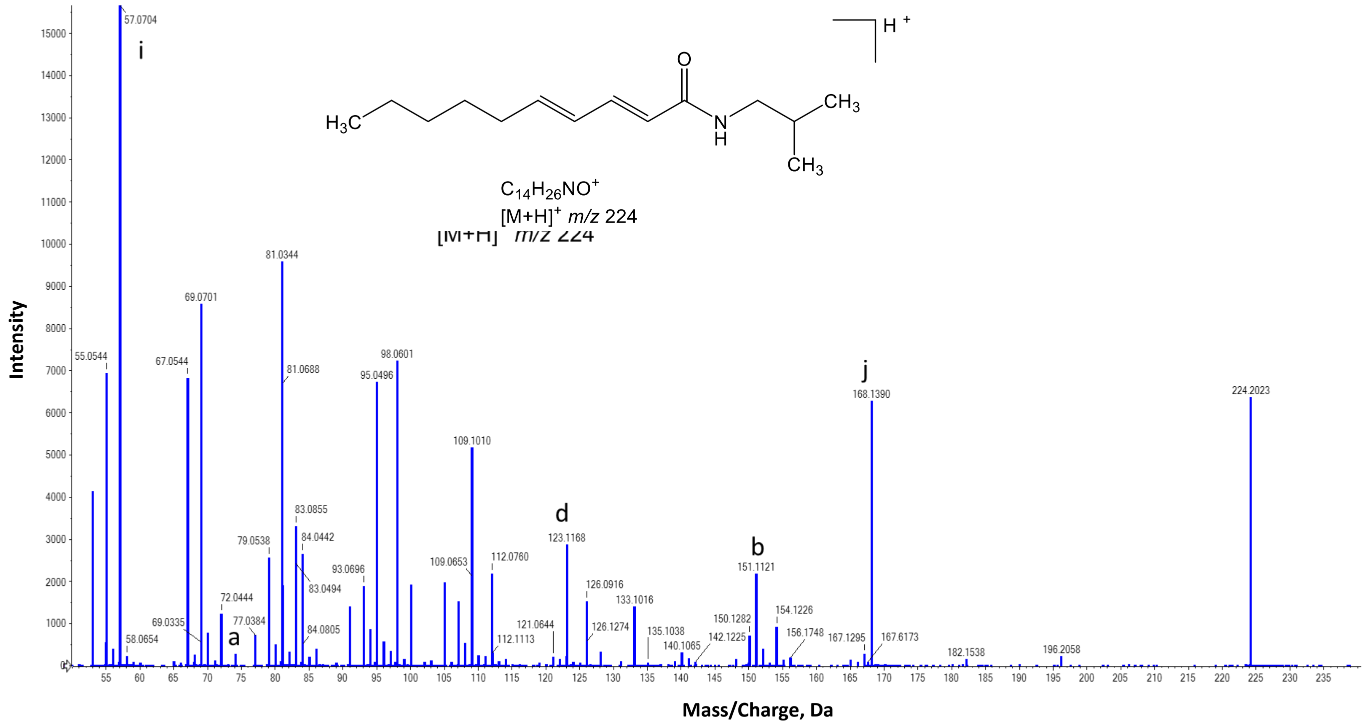

Figure S10_116: MS/MS spectrum of P116.

P117, Tricholein

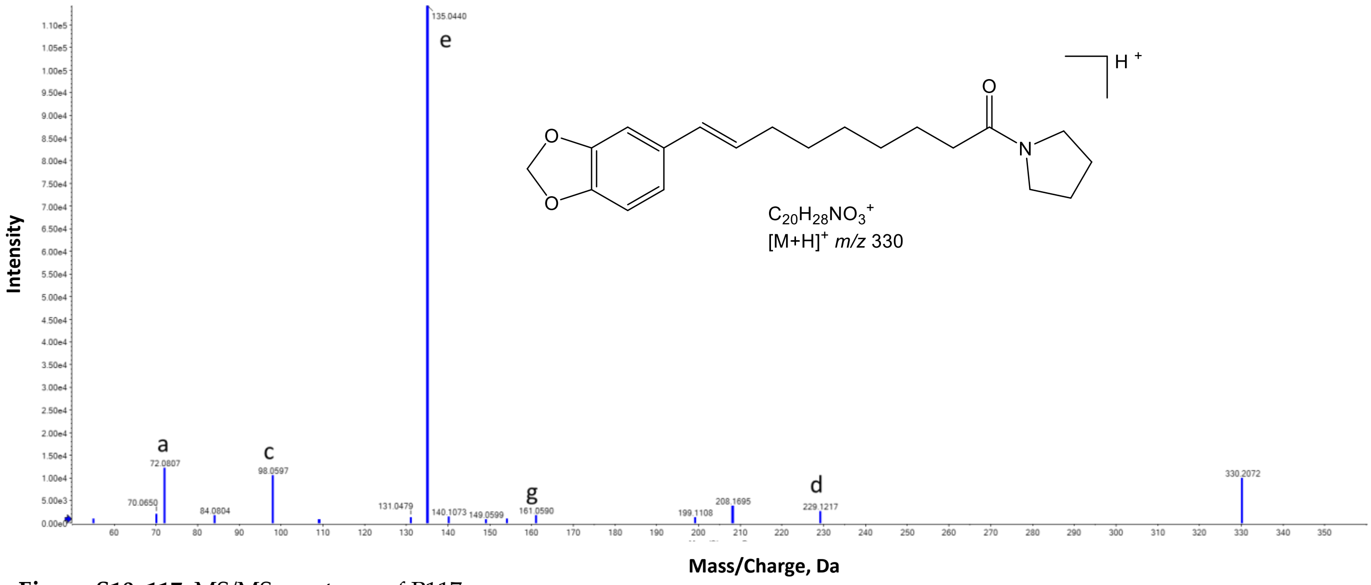

Figure S10_117: MS/MS spectrum of P117.

P118, 1-(Pyrrolidinyl-11-(3',4'-methylenedioxyphenyl)-2,4,8,10-undecatetraen-1-one

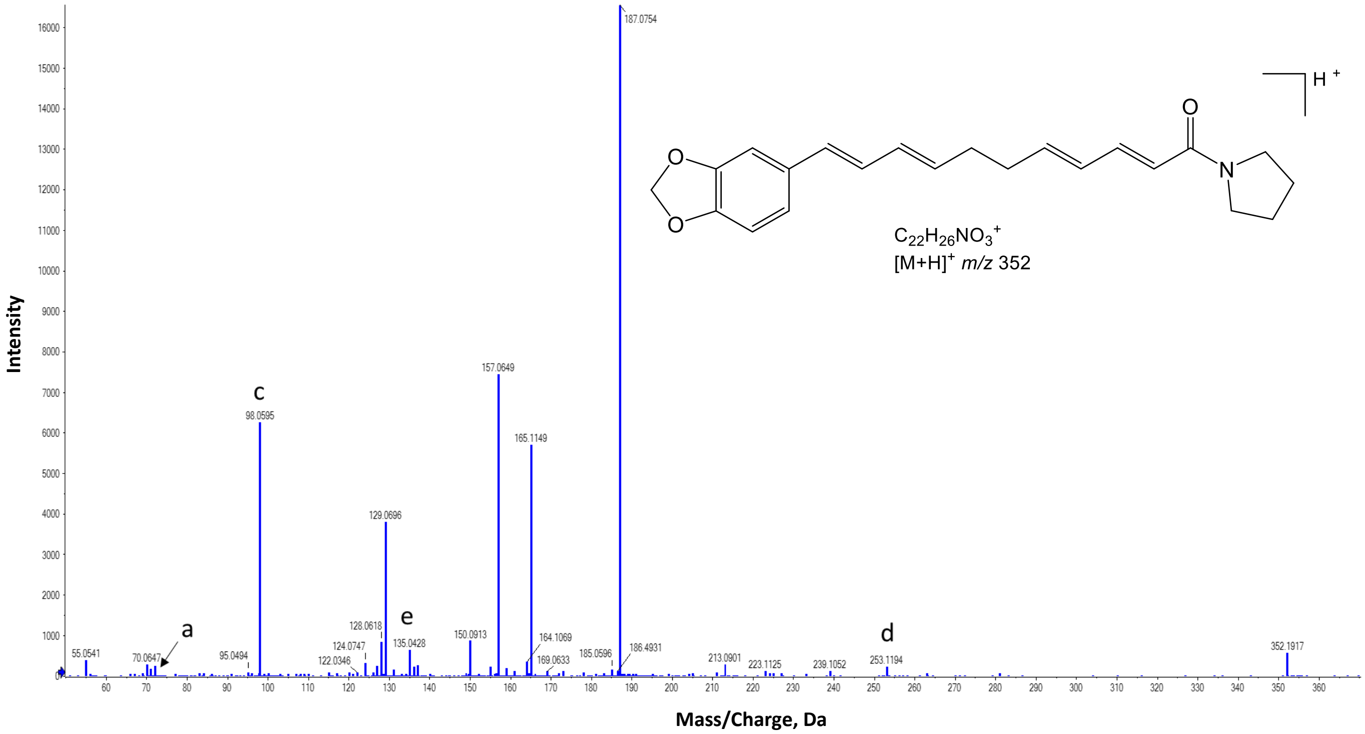

Figure S10_118: MS/MS spectrum of P118.

P119, Piperadione

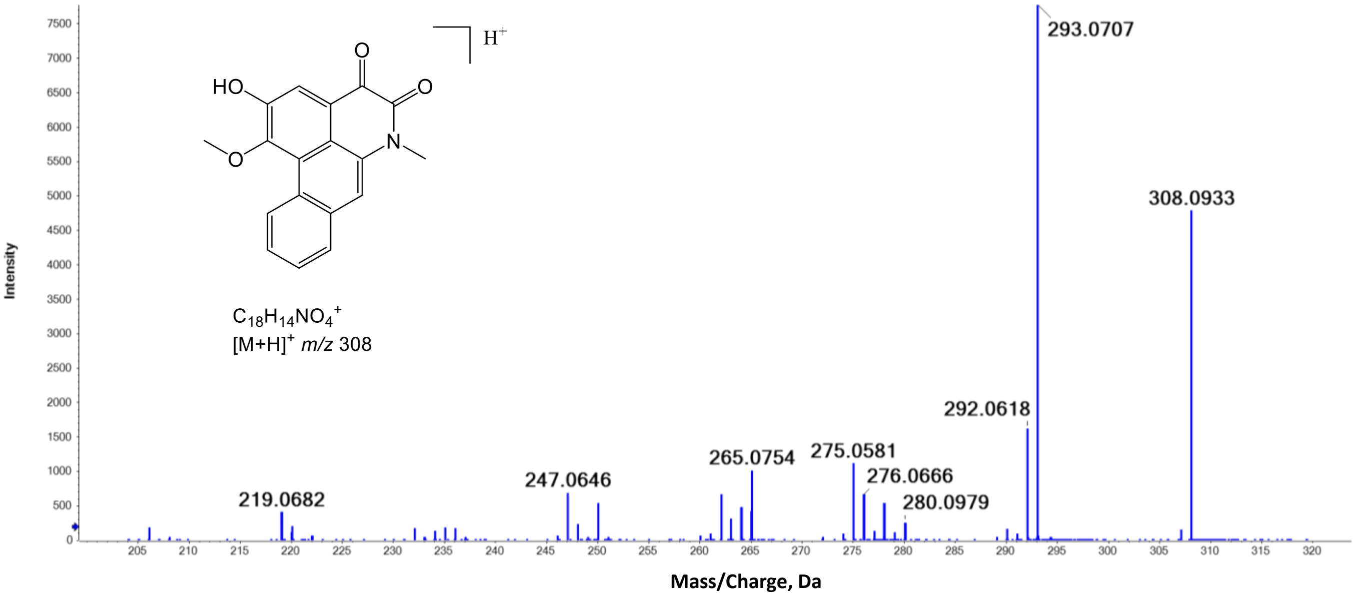

Figure S10_119: MS/MS spectrum of P119.

P120: $C_{21}H_{28}NO_3^+$, isomer of pipernonaline (formula see **P123**)

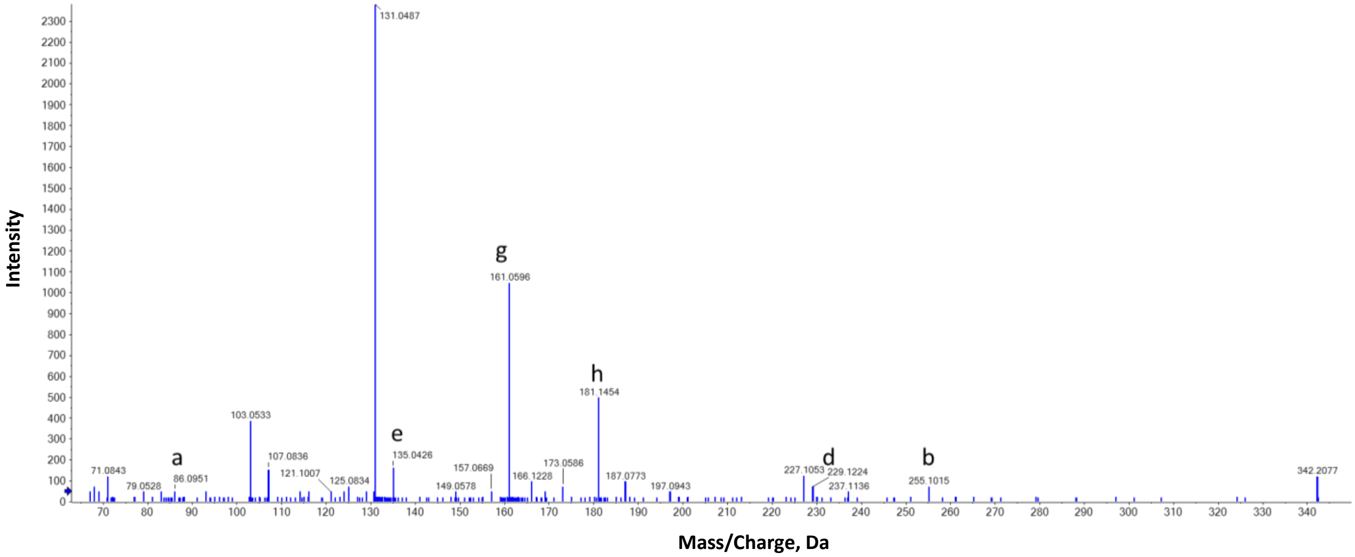

Figure S10_120: MS/MS spectrum of P120.

P121, 1-(Pyrrolidinyl-11-(3',4'-methylenedioxyphenyl)-2,4,10-undecatrien-1-one

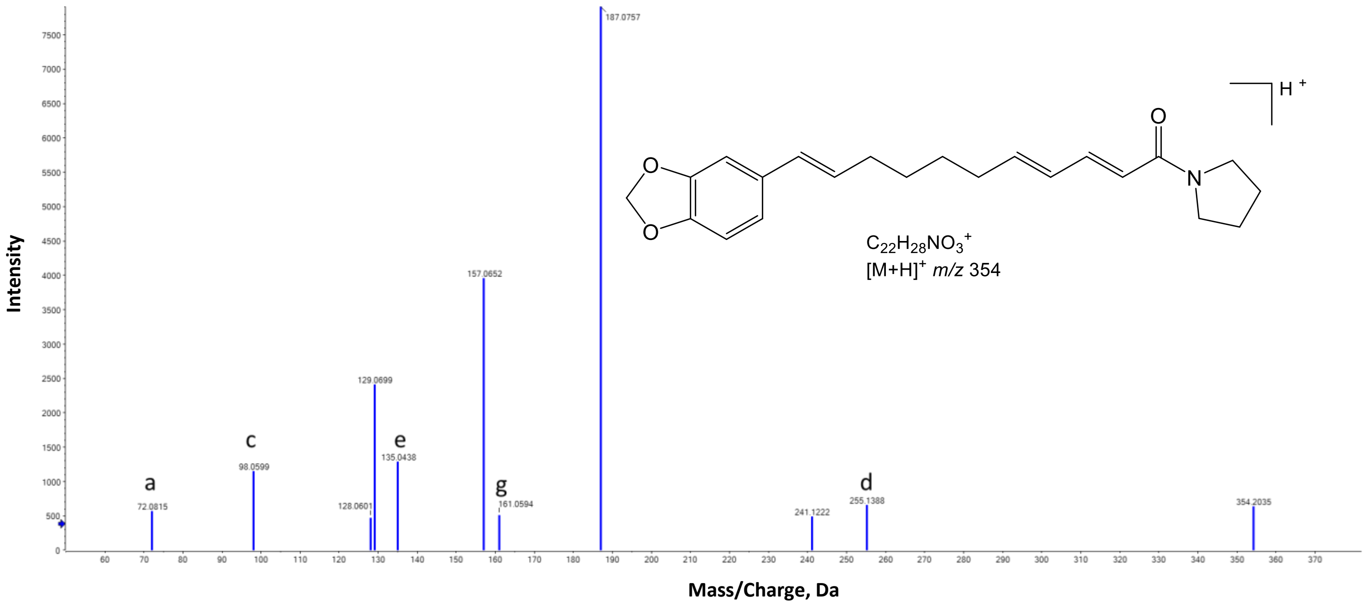

Figure S10_121: MS/MS spectrum of P121.

P122, Andamanicin*

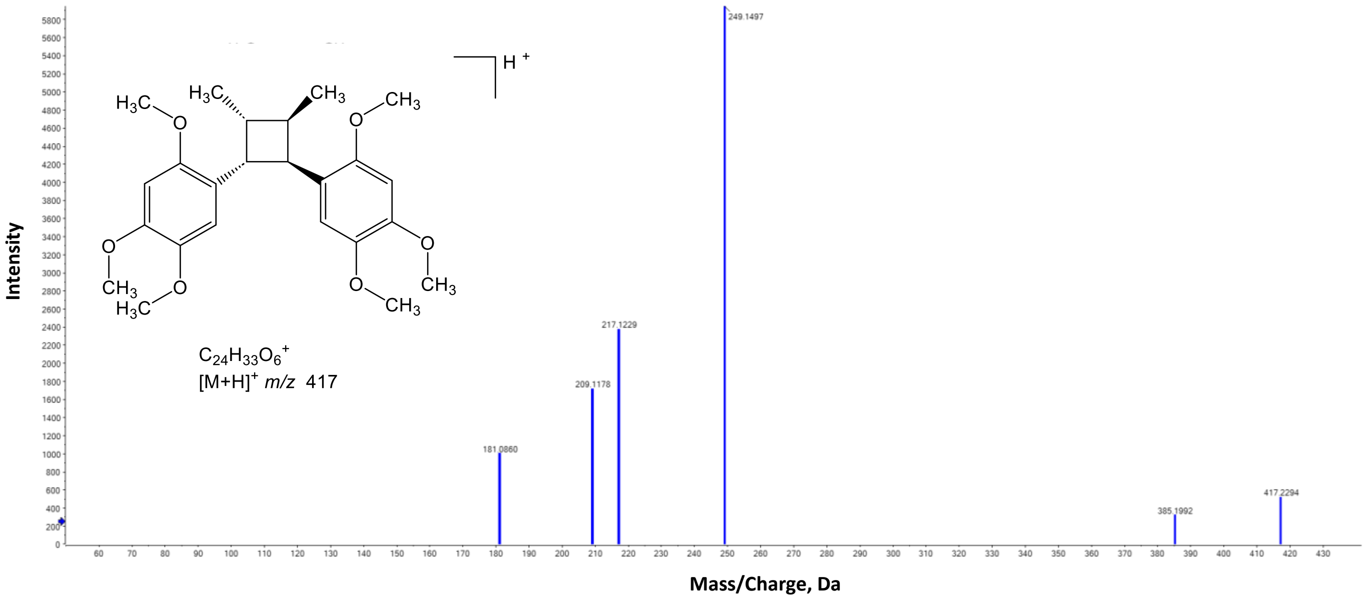

Figure S10_122: MS/MS spectrum of P122.

P123, Pipernonaline/ Pipernonatine

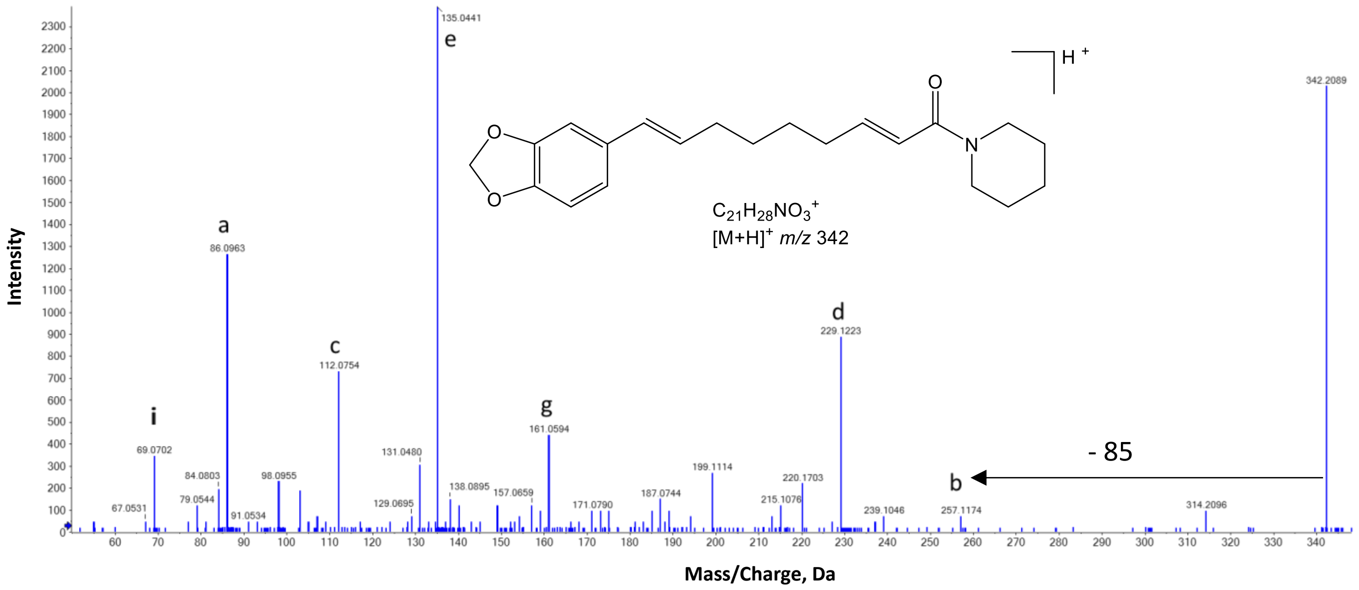

Figure S10_123: MS/MS spectrum of P123.

P124, Homopellitorine / N-2'-methylbutyl-decadienamide

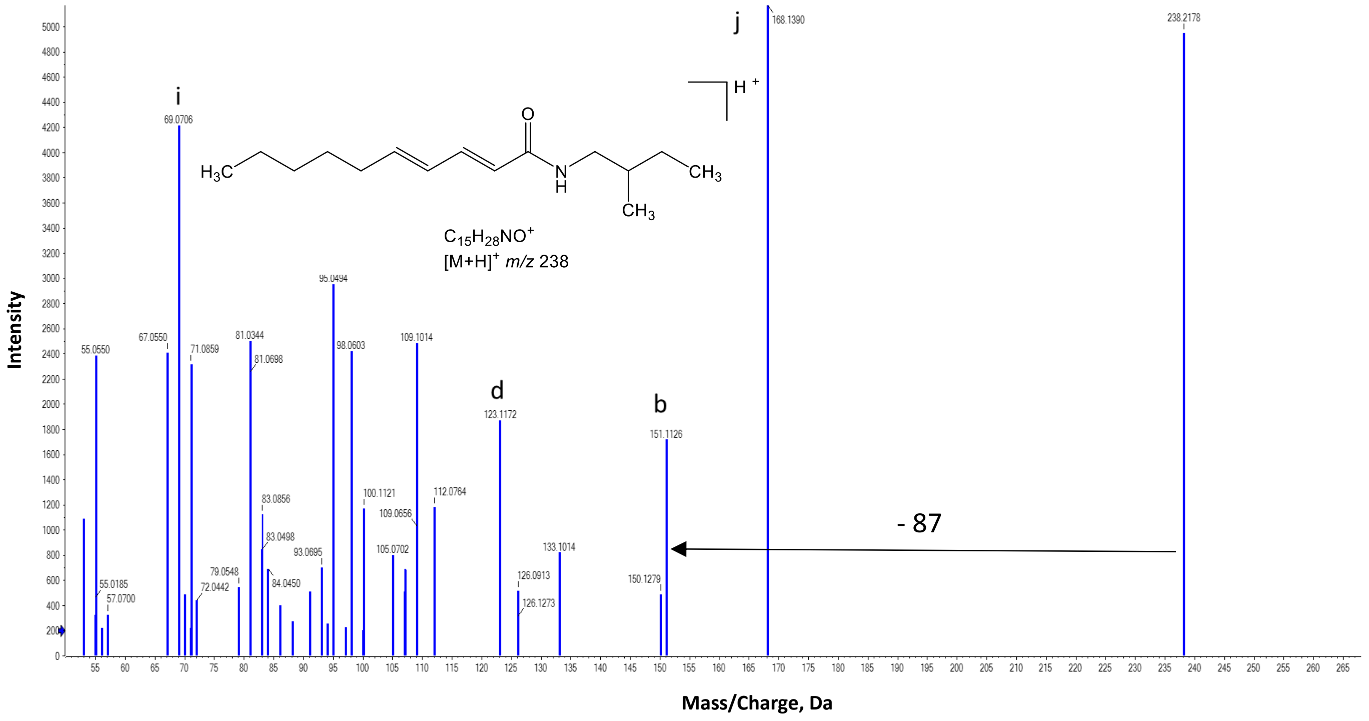

Figure S10_124: MS/MS spectrum of P124.

P125: $C_{21}H_{28}NO_3^+$, isomer of 1-(Pyrrolidinyl-11-(3',4'-methylenedioxyphenyl)-2,4,10-undecatrien-1-one (formula see **P121**)

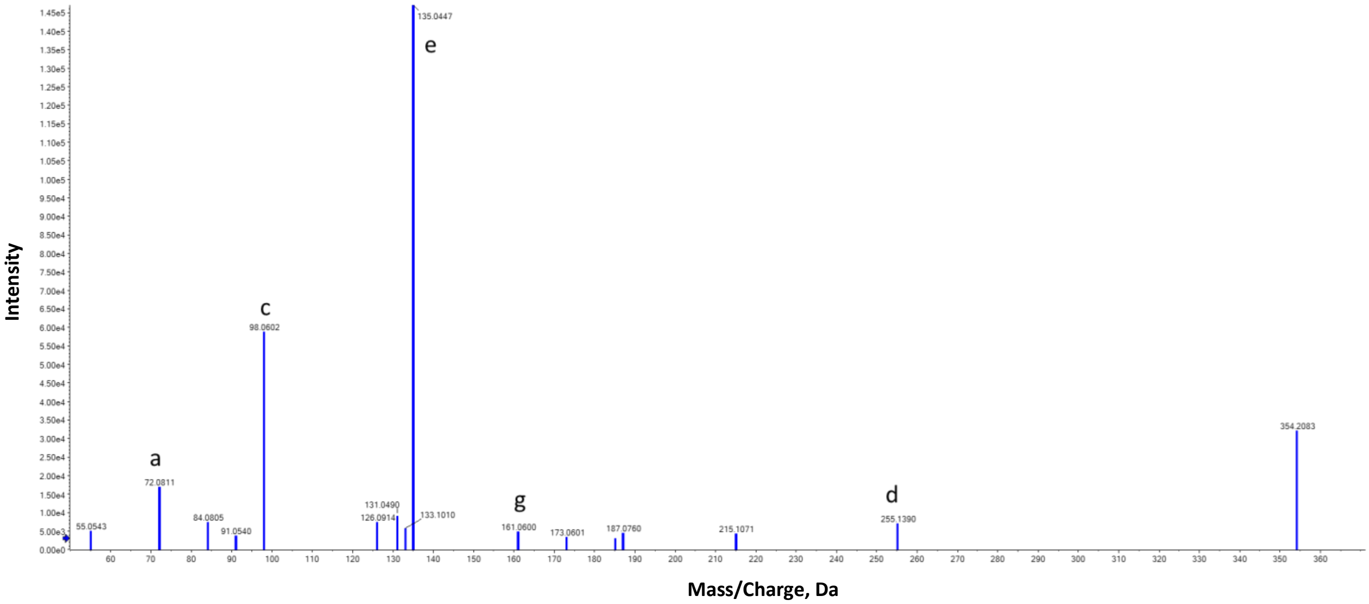

Figure S10_125: MS/MS spectrum of P125.

P126, Heterotropan

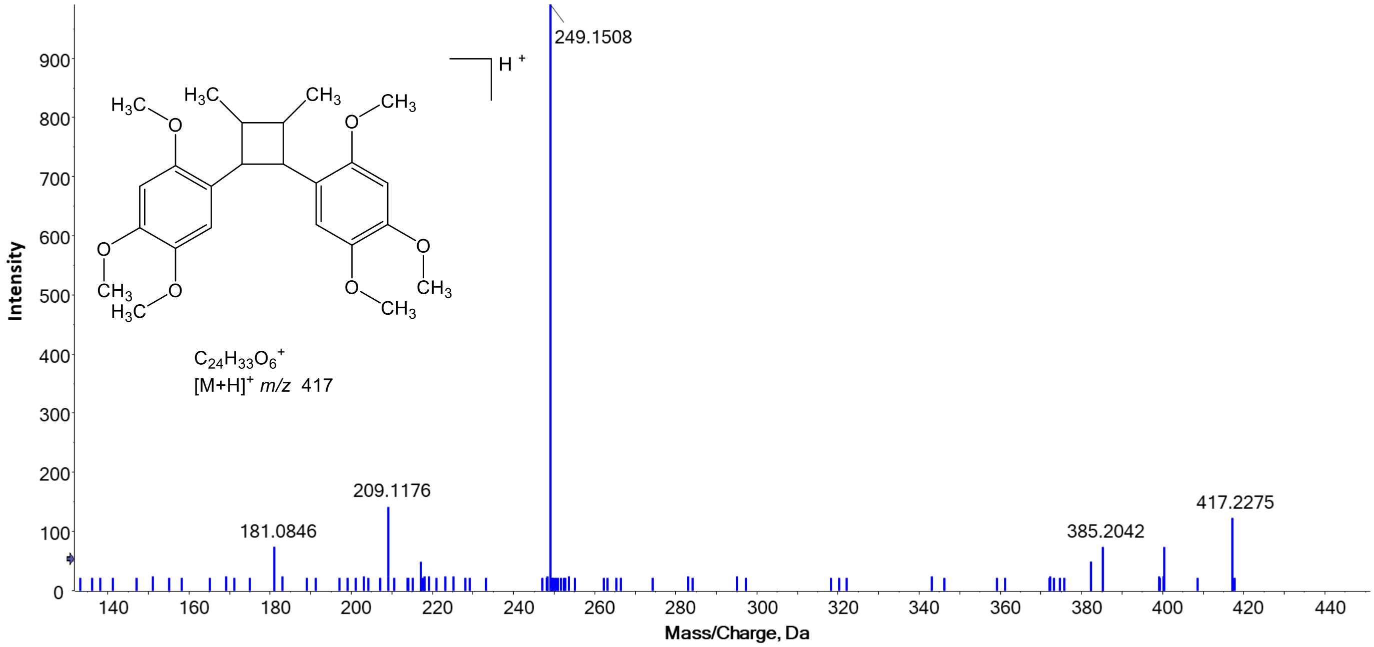

Figure S10_126: MS/MS spectrum of P126.

P127, Magnosalin*

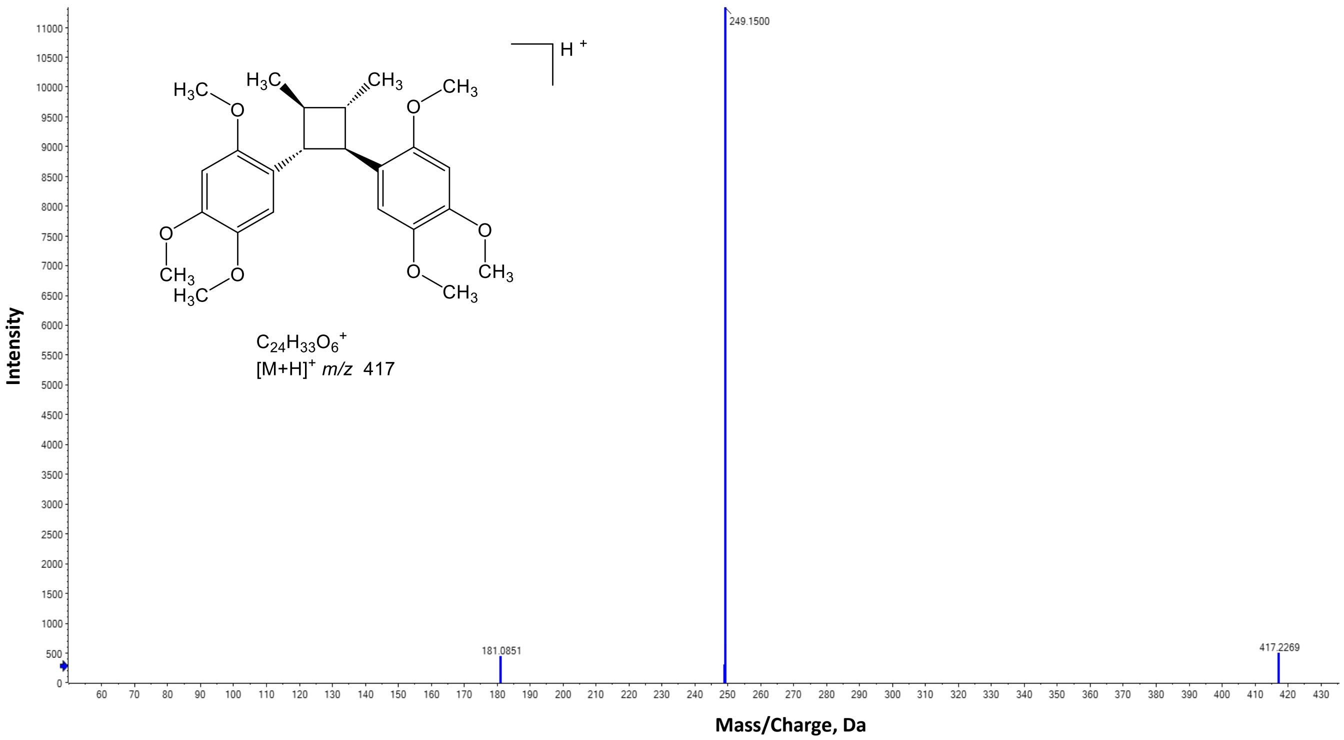

Figure S10_127: MS/MS spectrum of P127.

P128, Retrofractamide B

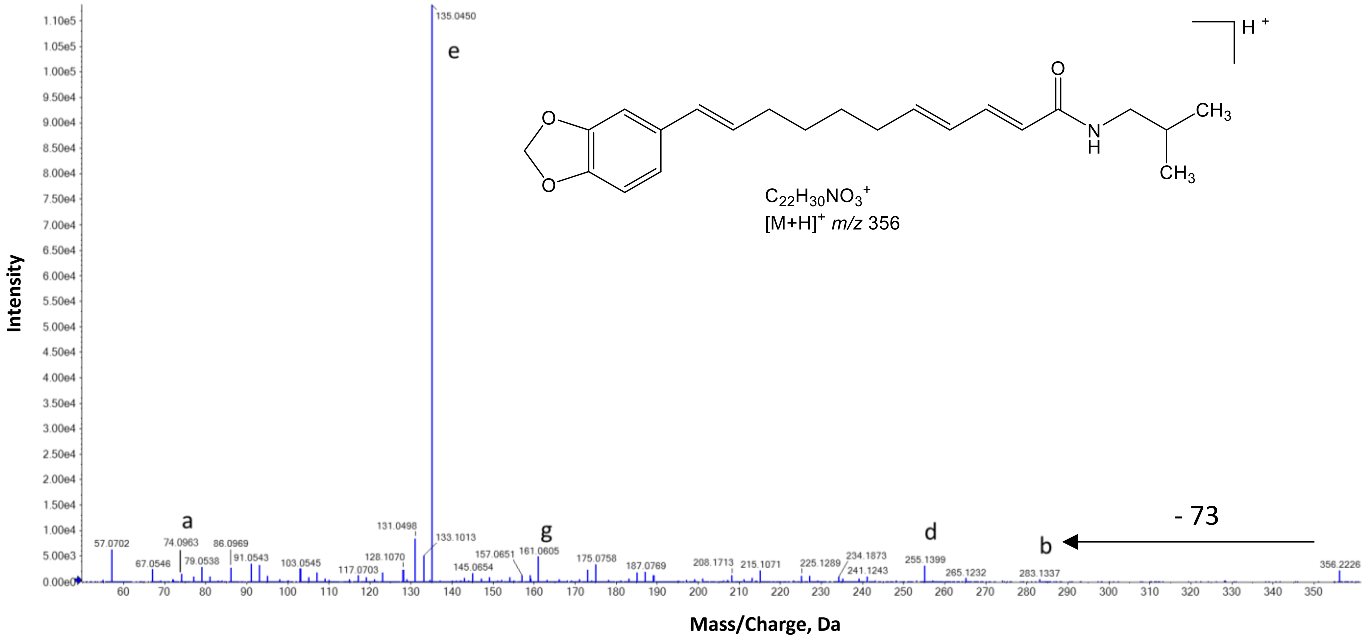

Figure S10_128: MS/MS spectrum of P128.

P129, 11-(3,4-methylenedioxyphenyl)-2,10-undecenoyl]pyrrolidine

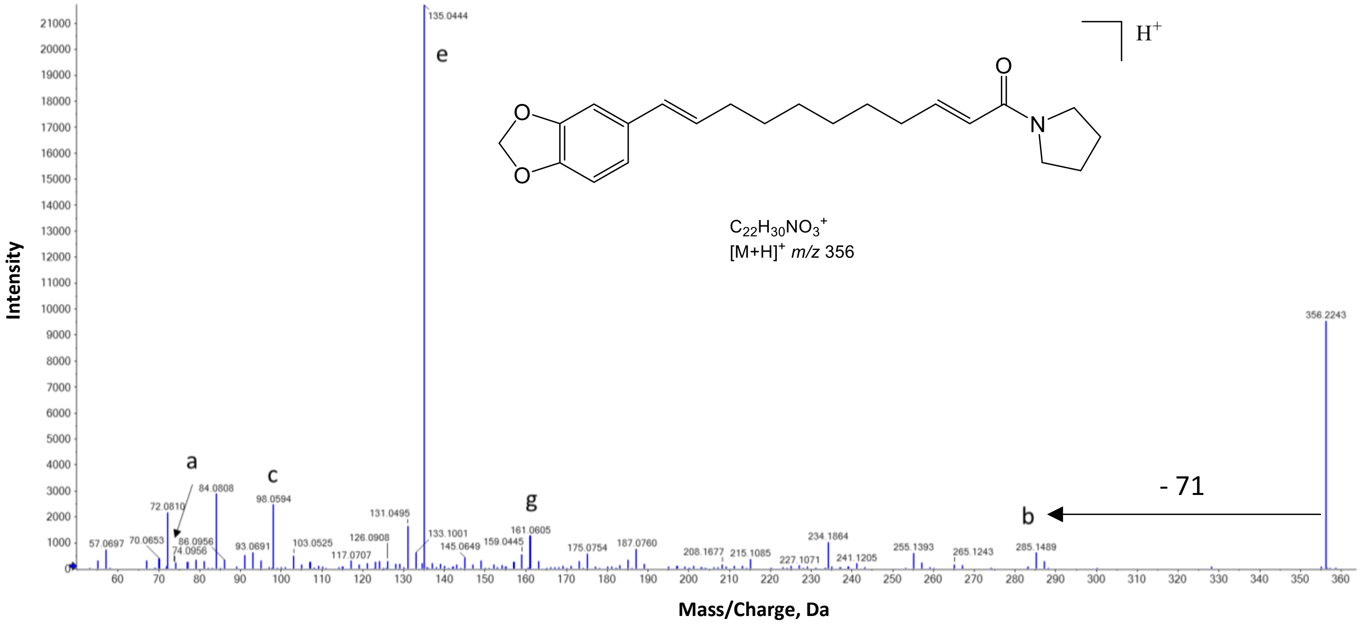

Figure S10_129: MS/MS spectrum of P129.

P130: $C_{12}H_{17}O_3^+$, isomer of *trans*-asarone (formula see **P95**)

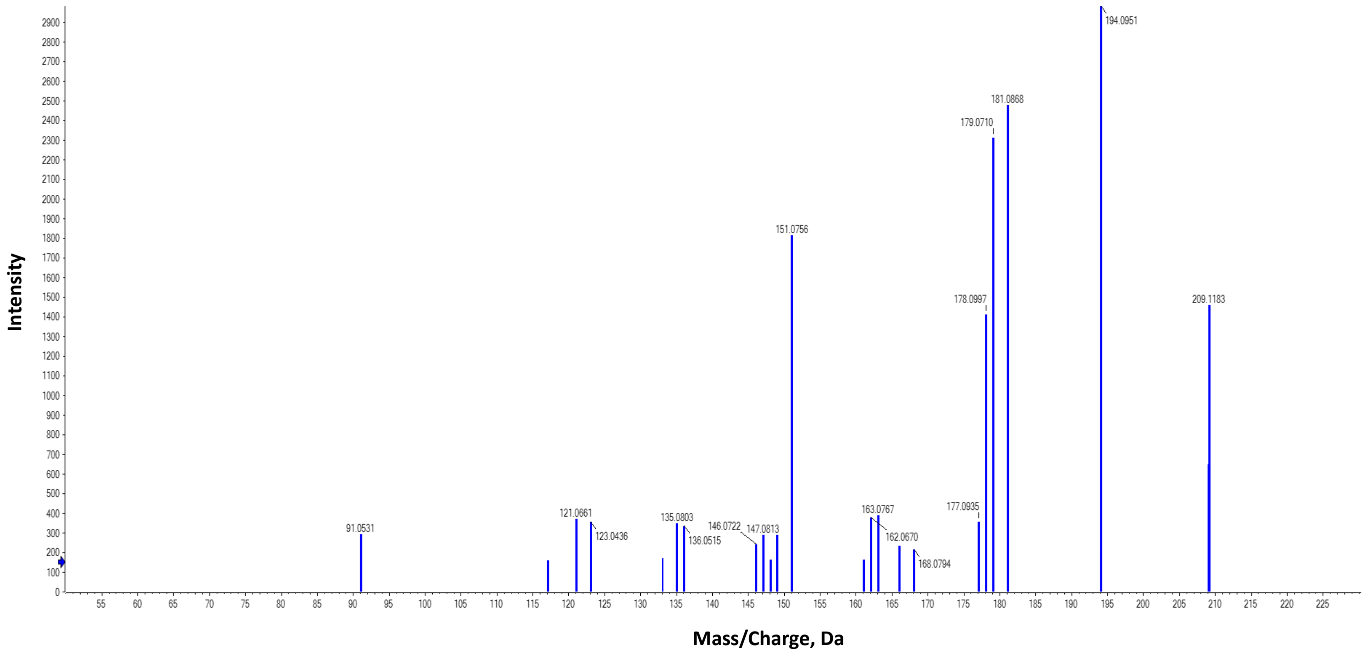

Figure S10_130: MS/MS spectrum of P130.

P131, 1-(Pyrrolidinyl-14-2,4,7,9-tetradecatetraen-1-one

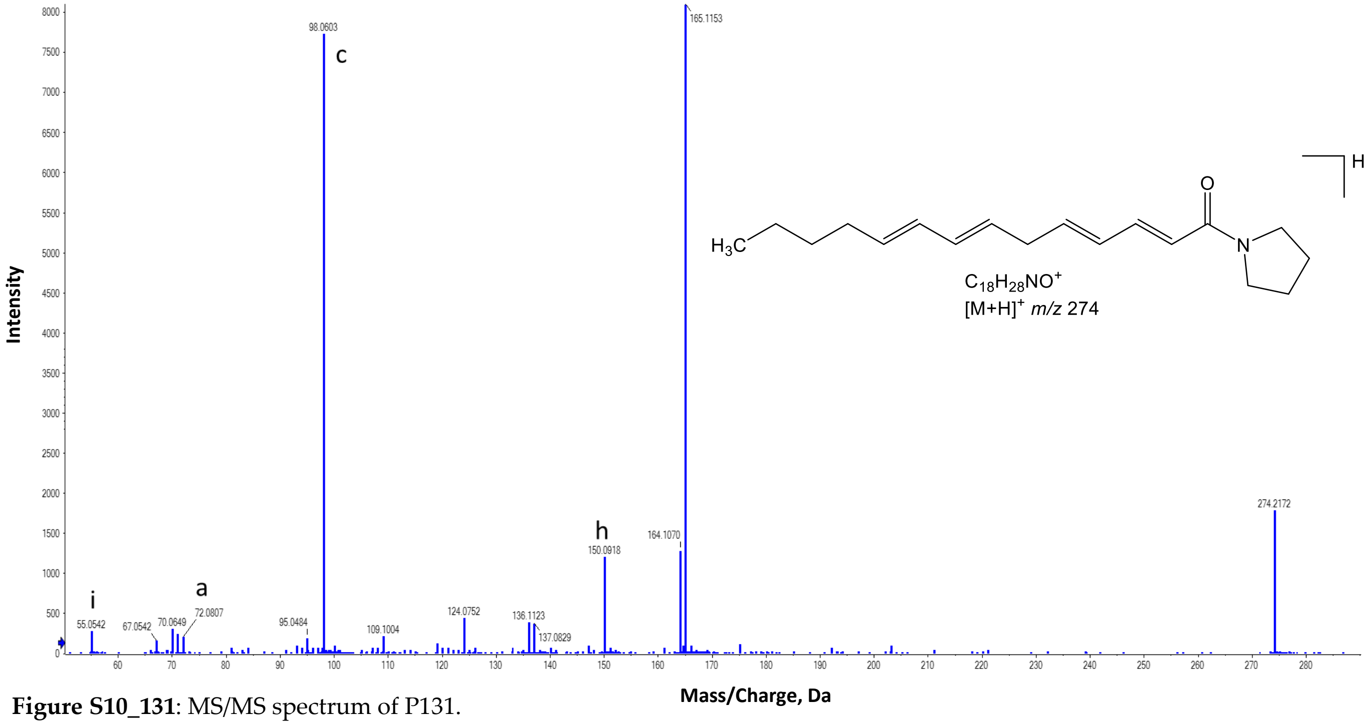

Figure S10_131: MS/MS spectrum of P131.

P132, 2,4-Dodecadienoyl pyrrolidine

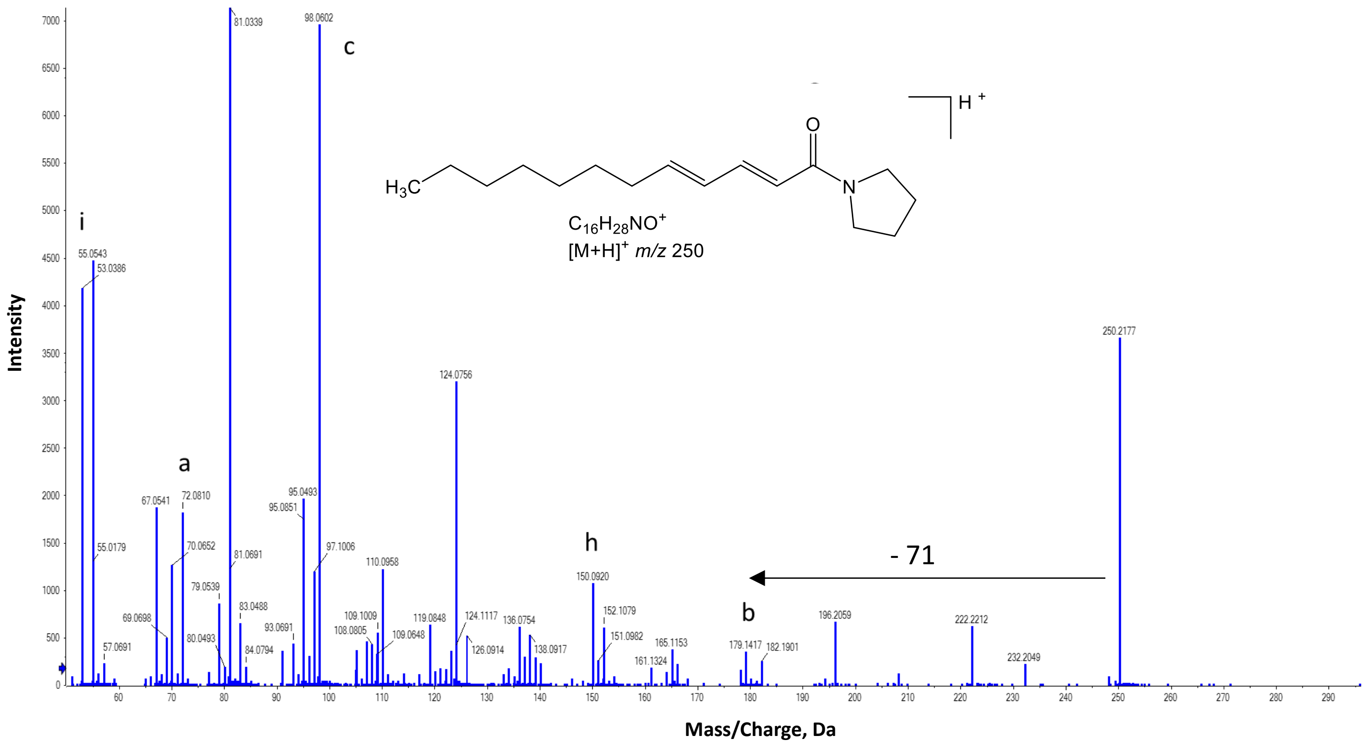

Figure S10_132: MS/MS spectrum of P132.

P133, Kalecide

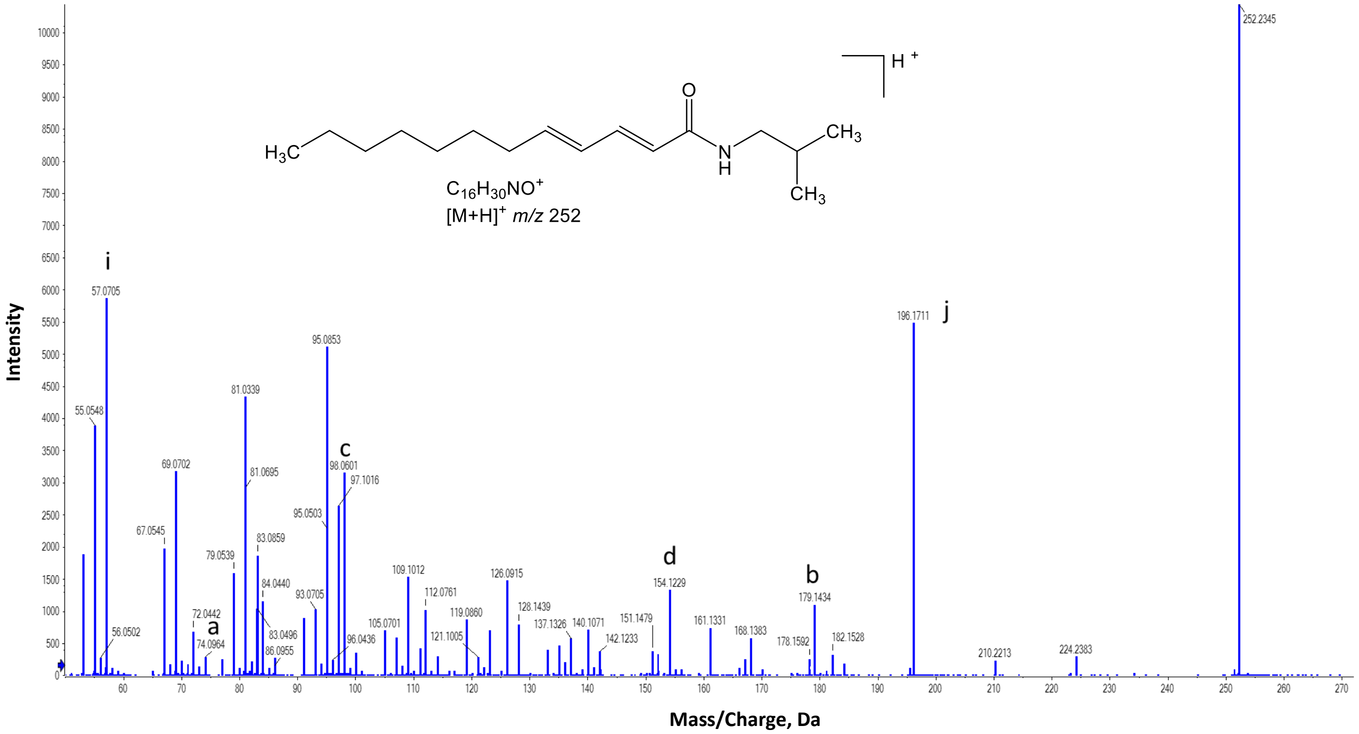

Figure S10_133: MS/MS spectrum of P133.

P134, Dehydroguineesine

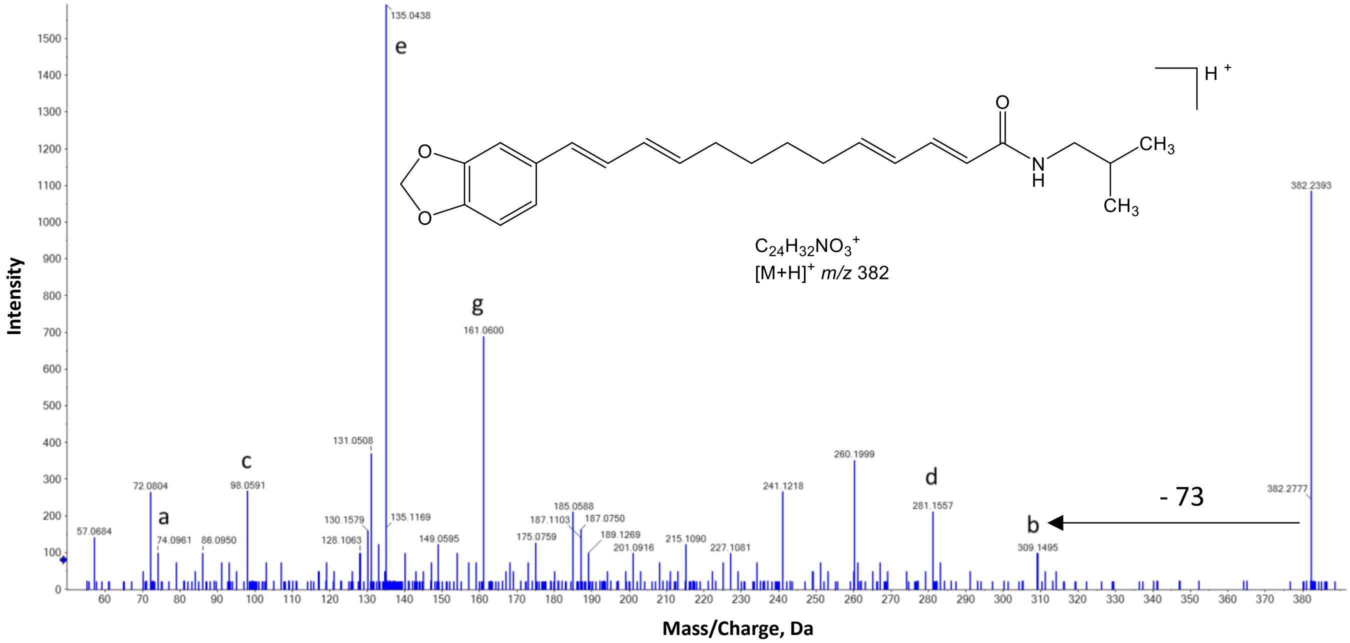

Figure S10_134: MS/MS spectrum of P134.

P135, Brachyamide A

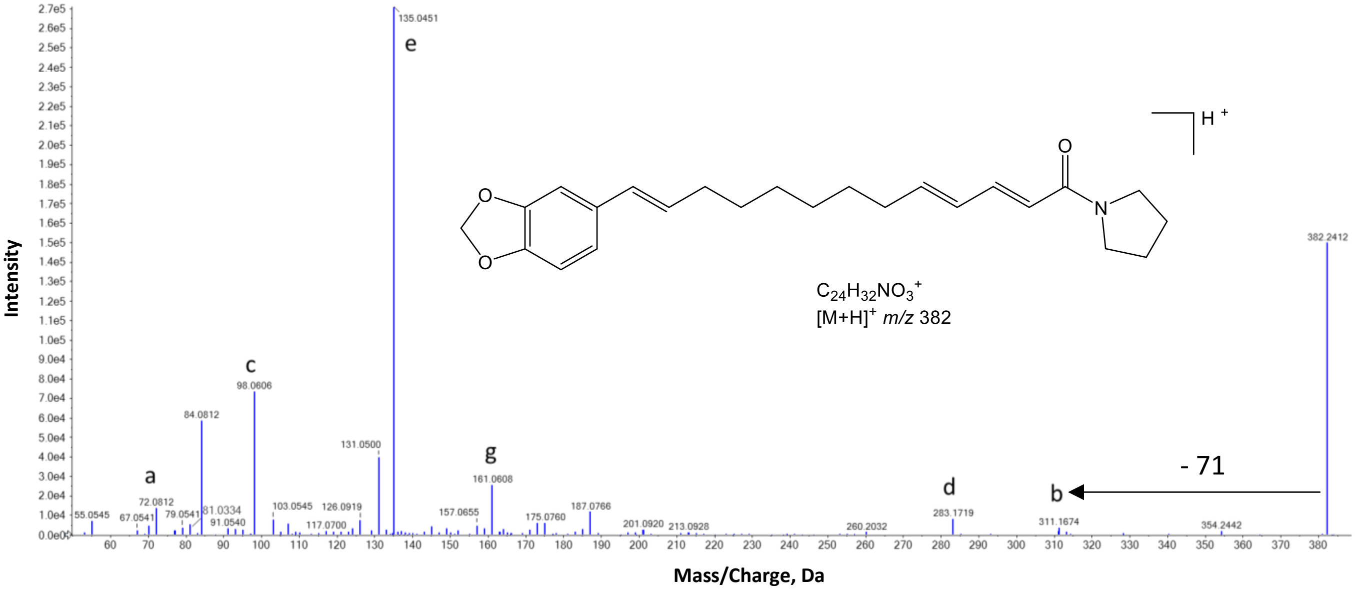

Figure S10_135: MS/MS spectrum of P135.

P136, Guineensine

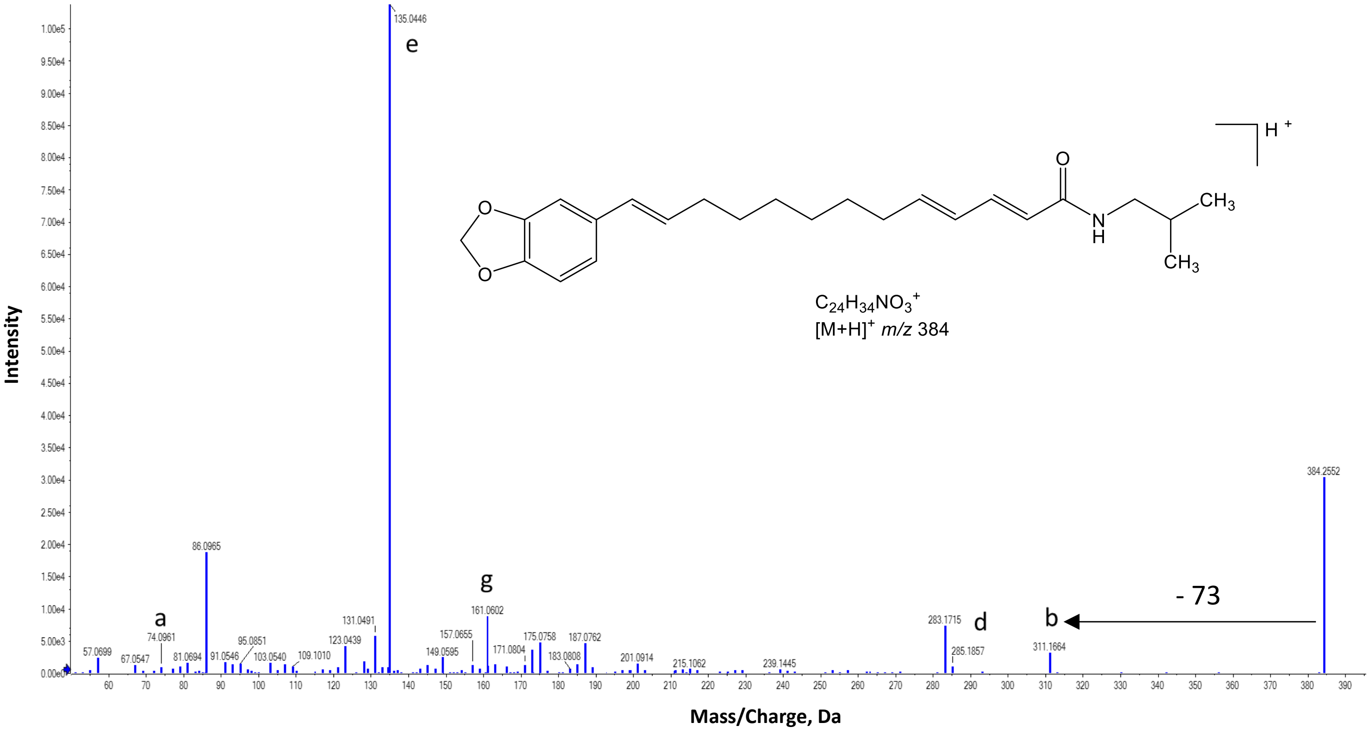

Figure S10_136: MS/MS spectrum of P136.

P137, Piperflaviflorine A

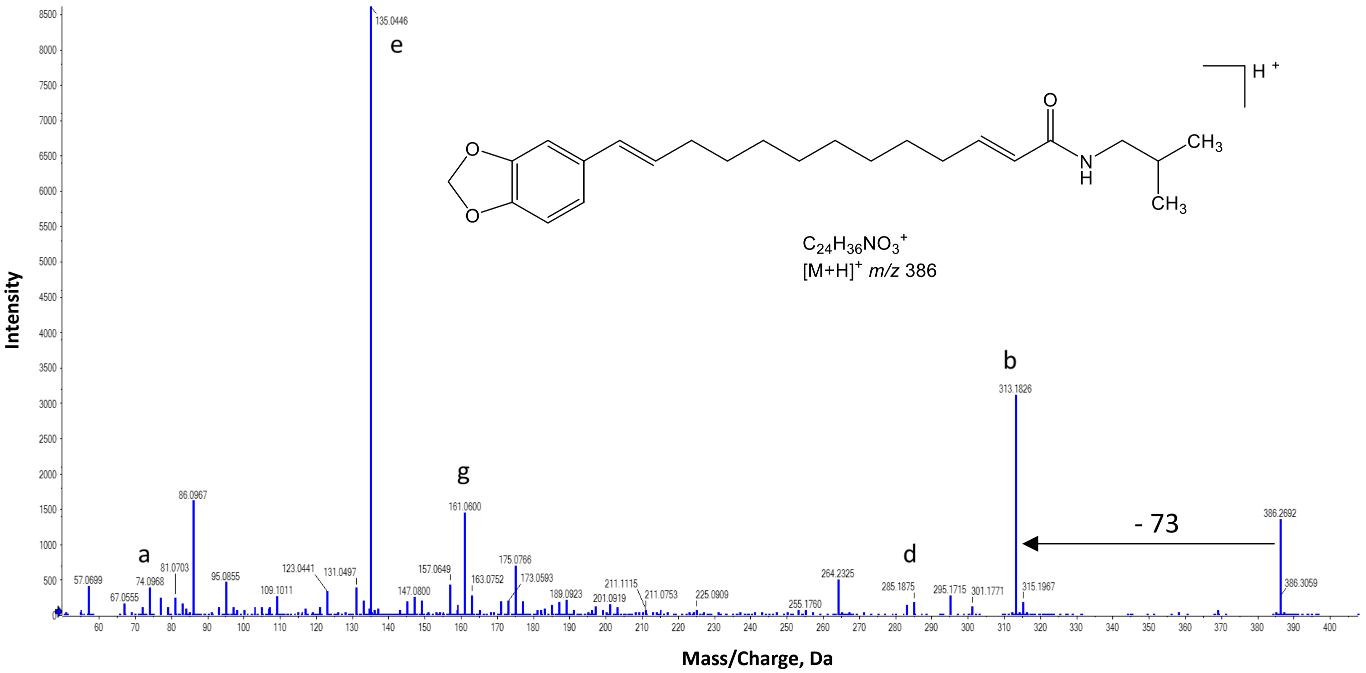

Figure S10_137: MS/MS spectrum of P137.

P138, Dihydrobrachyamide A

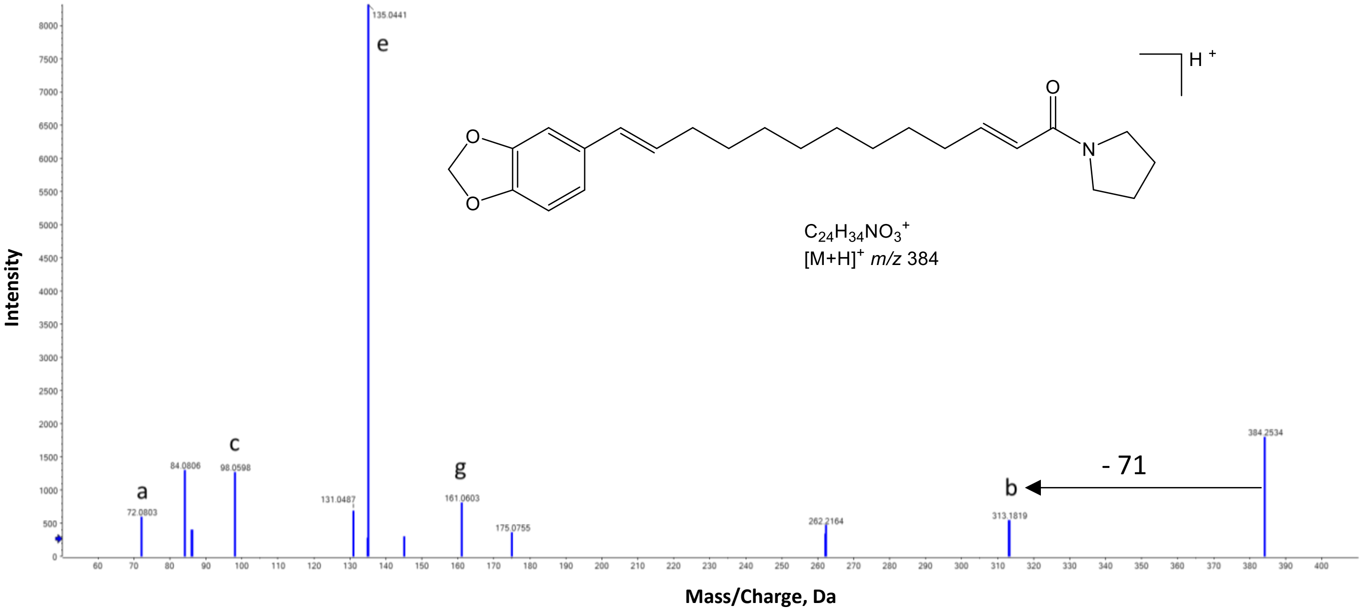

Figure S10_138: MS/MS spectrum of P138.

P139, Piperflaviflorine B

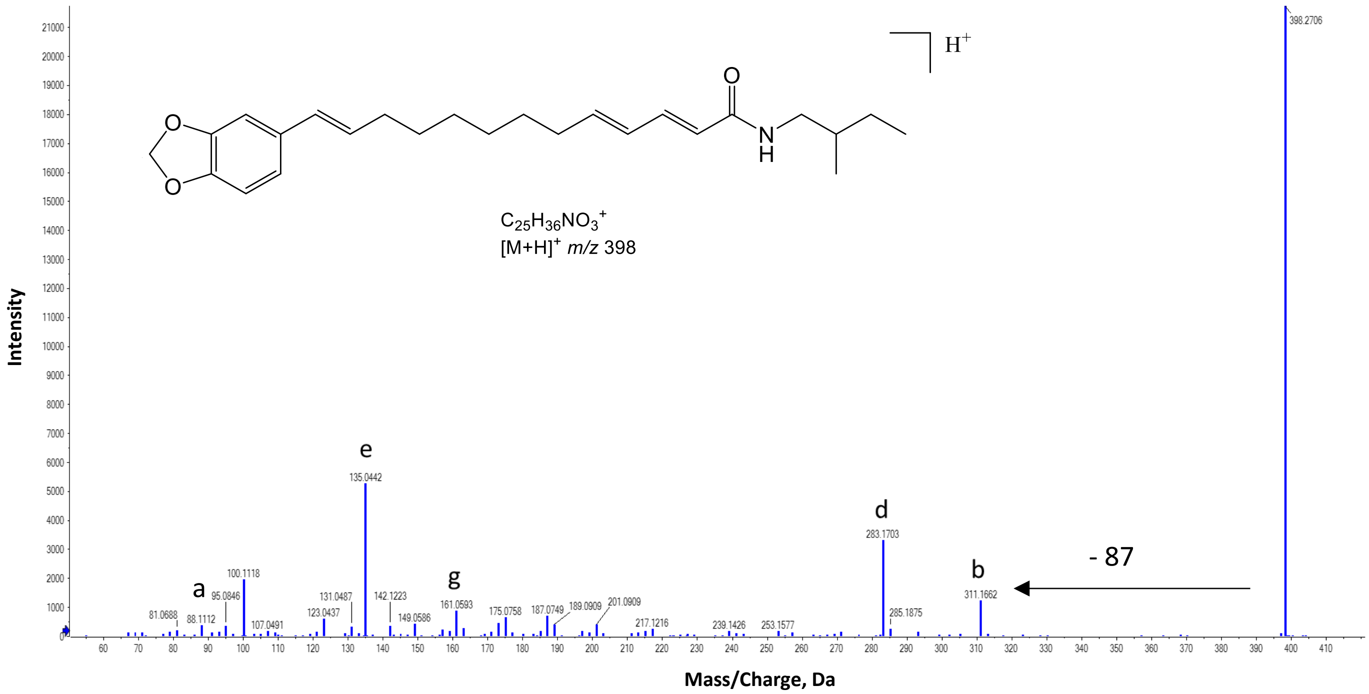

Figure S10_139: MS/MS spectrum of P139.

P140,1-(Pyrrolidinyl)-18-2,4,9,11,13- octadecatetraen-1-one

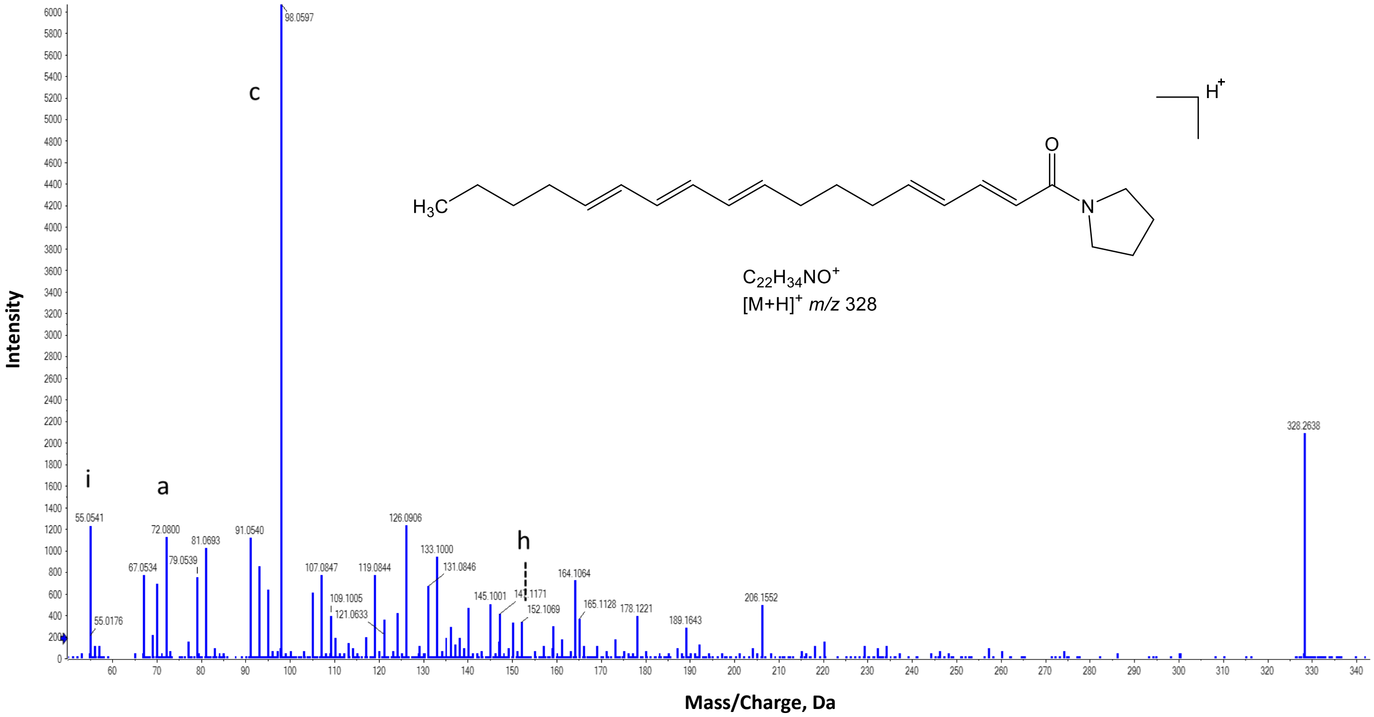

Figure S10_140: MS/MS spectrum of P140.

P141, Achilleaamide

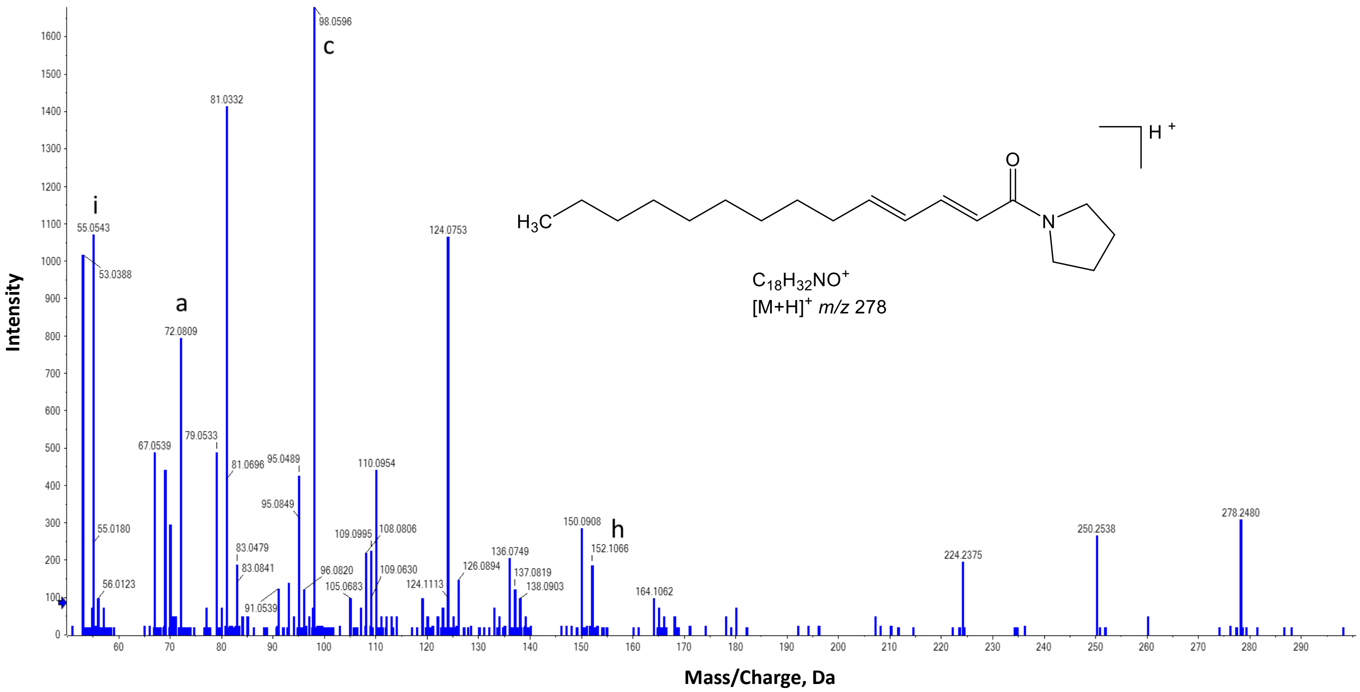

Figure S10_141: MS/MS spectrum of P141.

P142, 1-(Pyrrolidinyl)-18-2,4,11,13-octadecatetraen-1-one

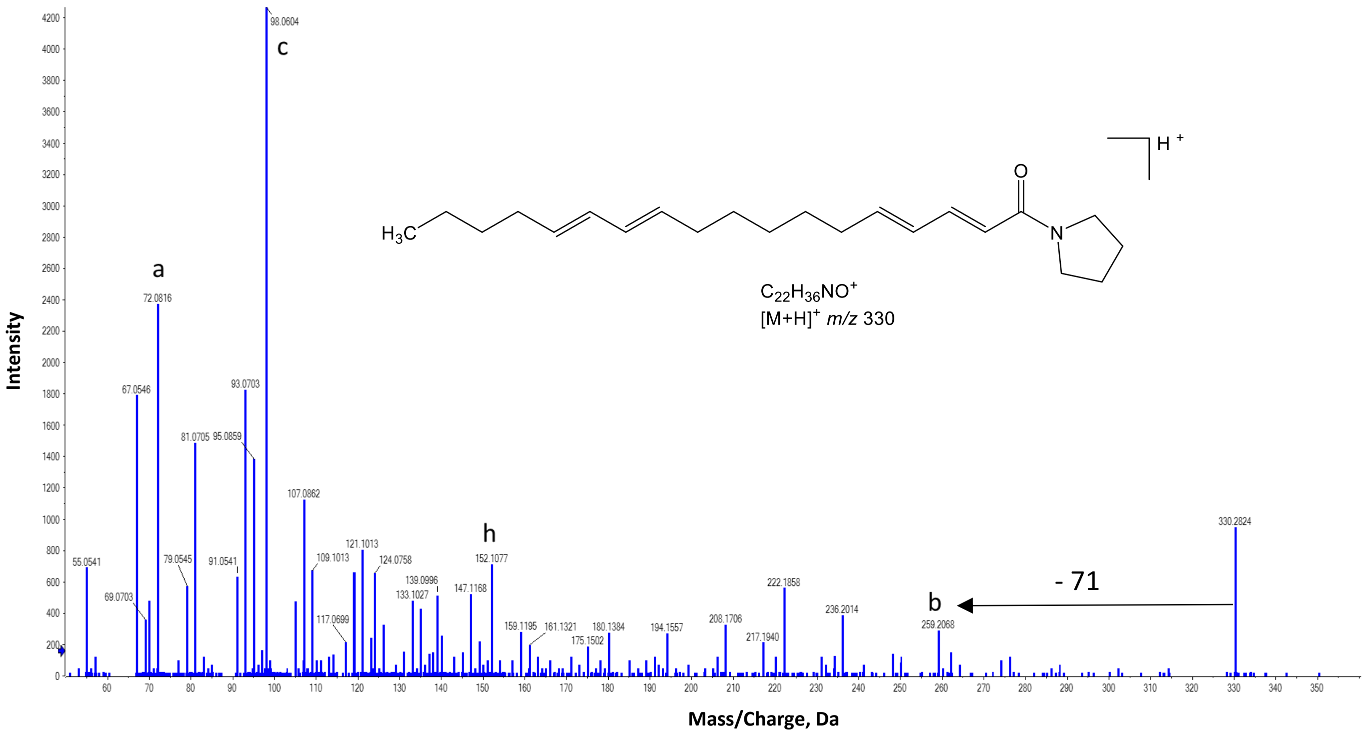

Figure S10_142: MS/MS spectrum of P142.

P143, Brachystamide B

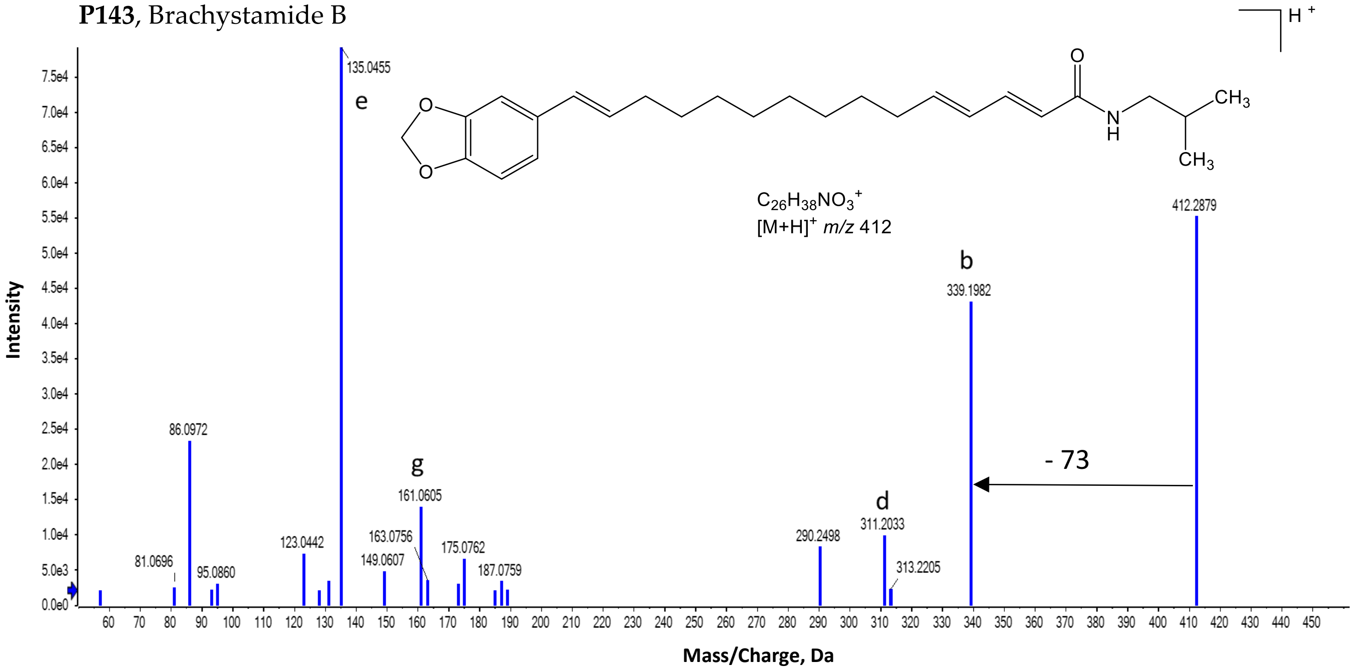

Figure S10_143: MS/MS spectrum of P143.

P144, 1-(Pyrrolidinyl)-15-(3',4'-methylenedioxyphenyl)-2,4,14-pentadecatrien-1-one

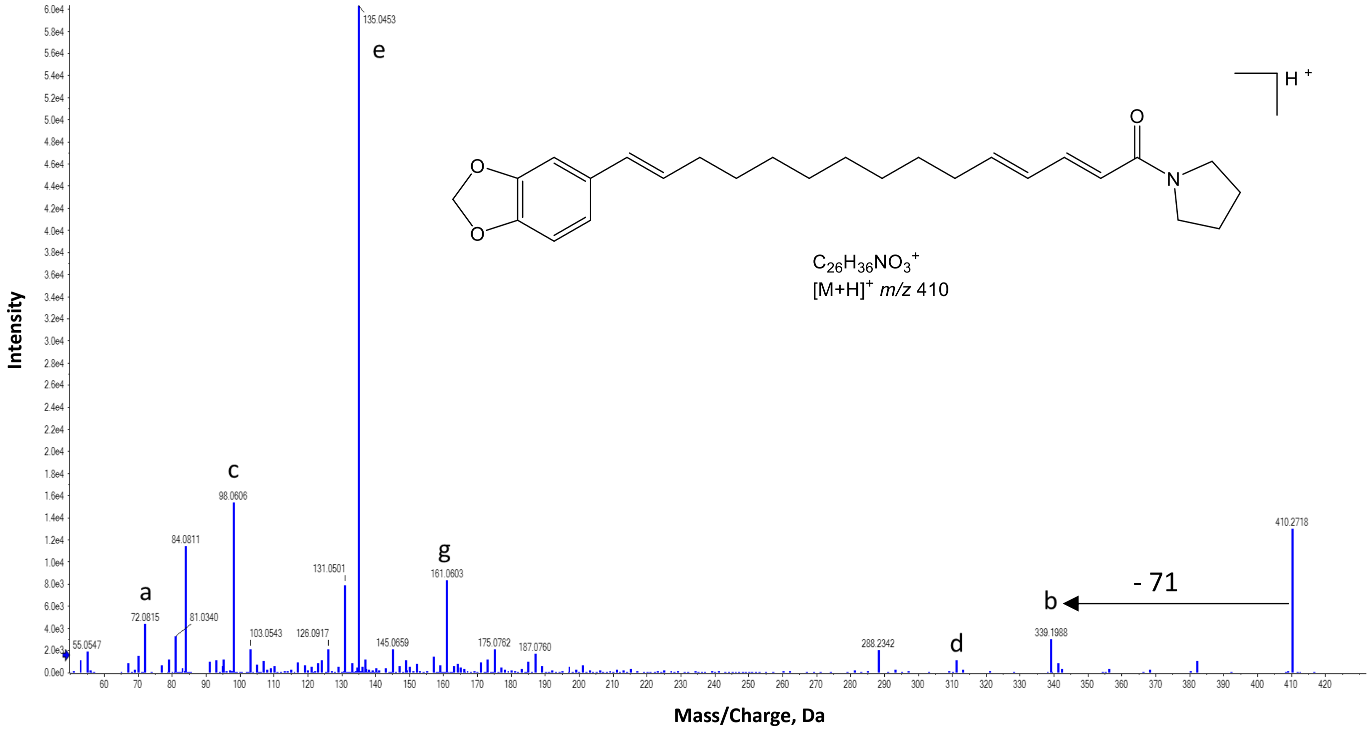

Figure S10_144: MS/MS spectrum of P144.

P145, *N*-Isobutyl- 2,4,10,12-octadecatetraenamide

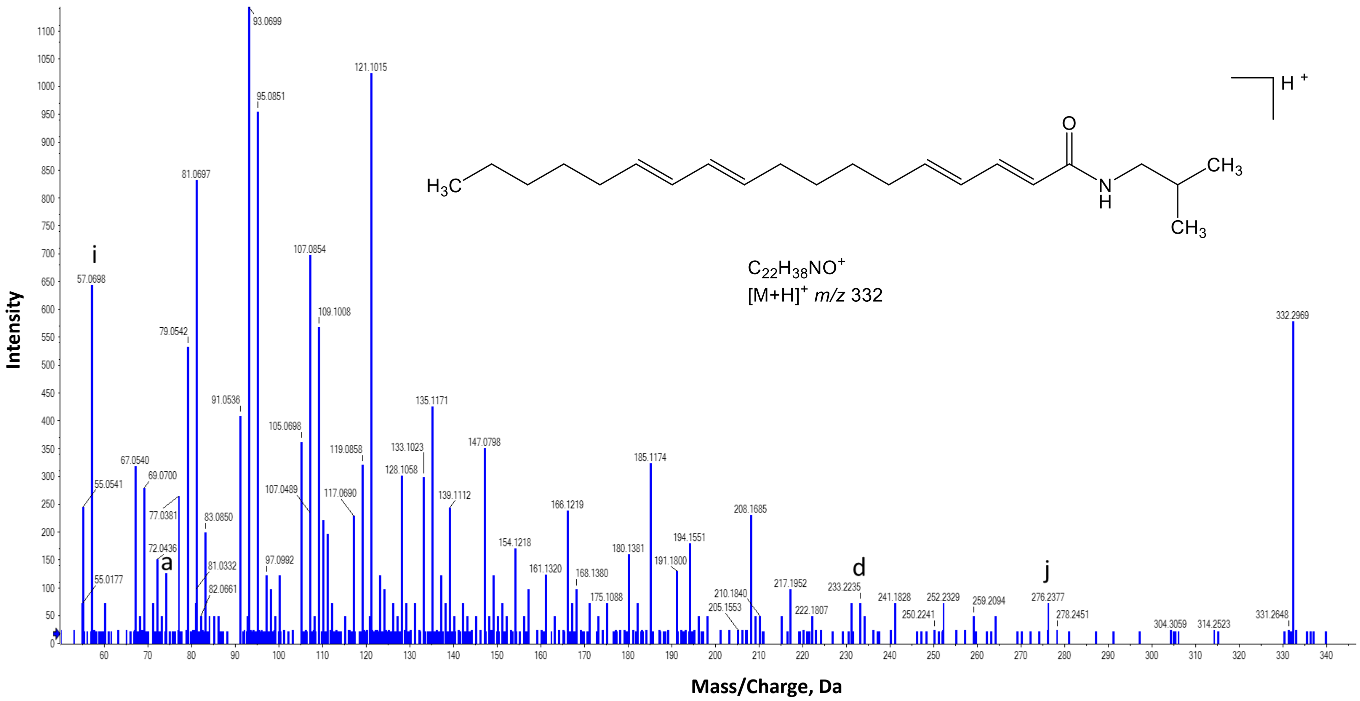

Figure S10_145: MS/MS spectrum of P1.

P146, 1-(Pyrrolidinyl)-15-(3',4'-methylenedioxyphenyl)-12,14-pentadecadien-1-one

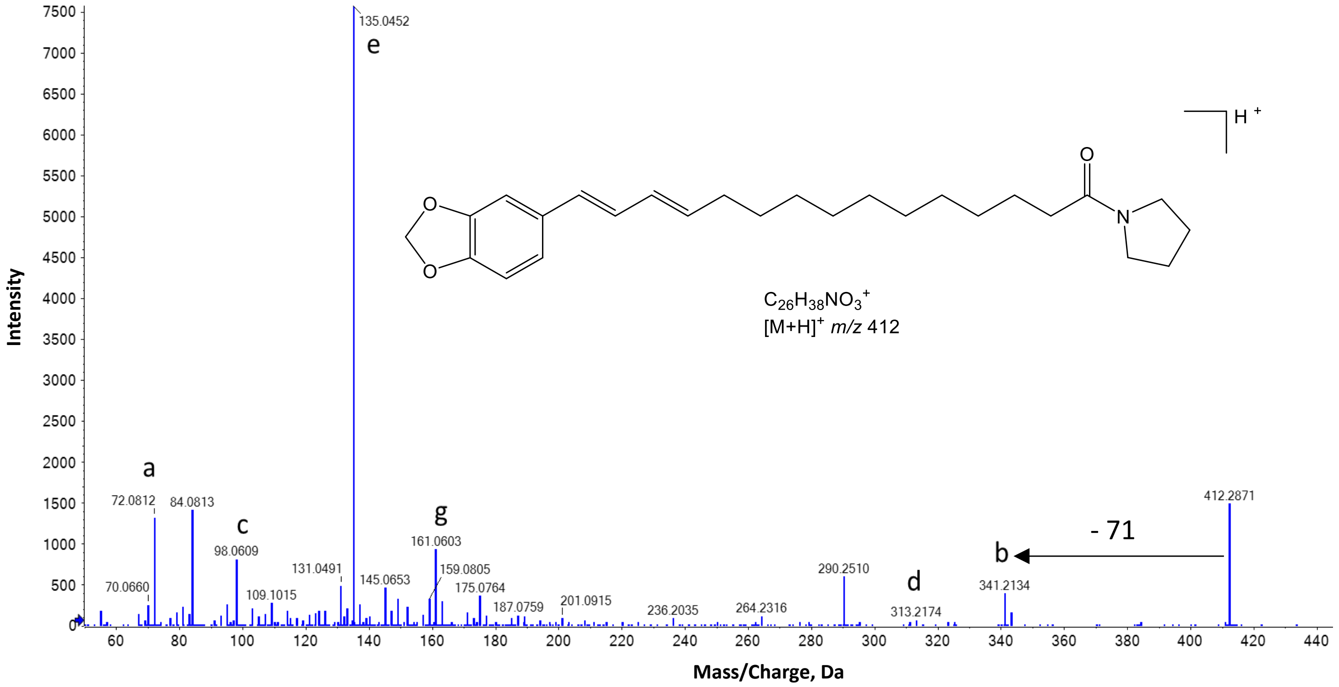

Figure S10_146: MS/MS spectrum of P146.

P147: $C_{21}H_{28}NO_3^+$, isomer of *N*-Isobutyl- 2,4,10,12-octadecatetraenmide (formula see **P145**)

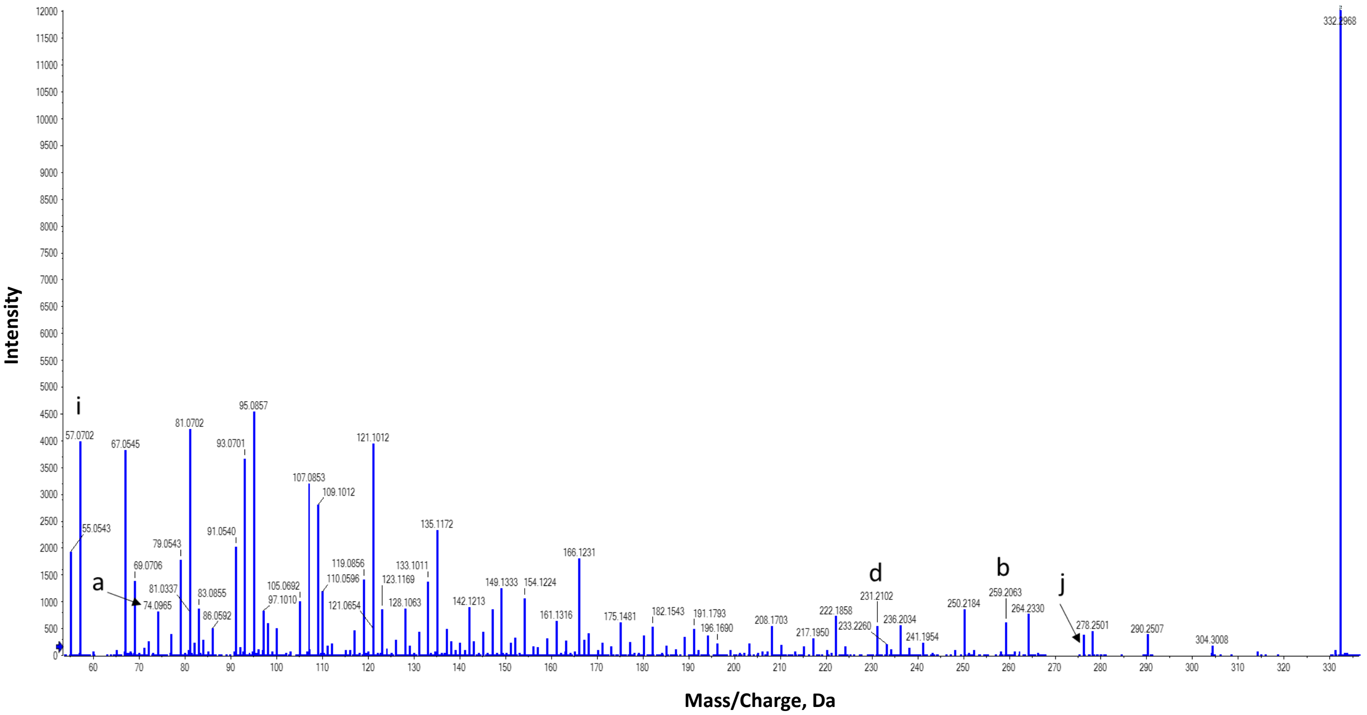

Figure S10_147: MS/MS spectrum of P147.

P148, 1-(Pyrrolidinyl)-2,4,10,12-octadecatetraenmide

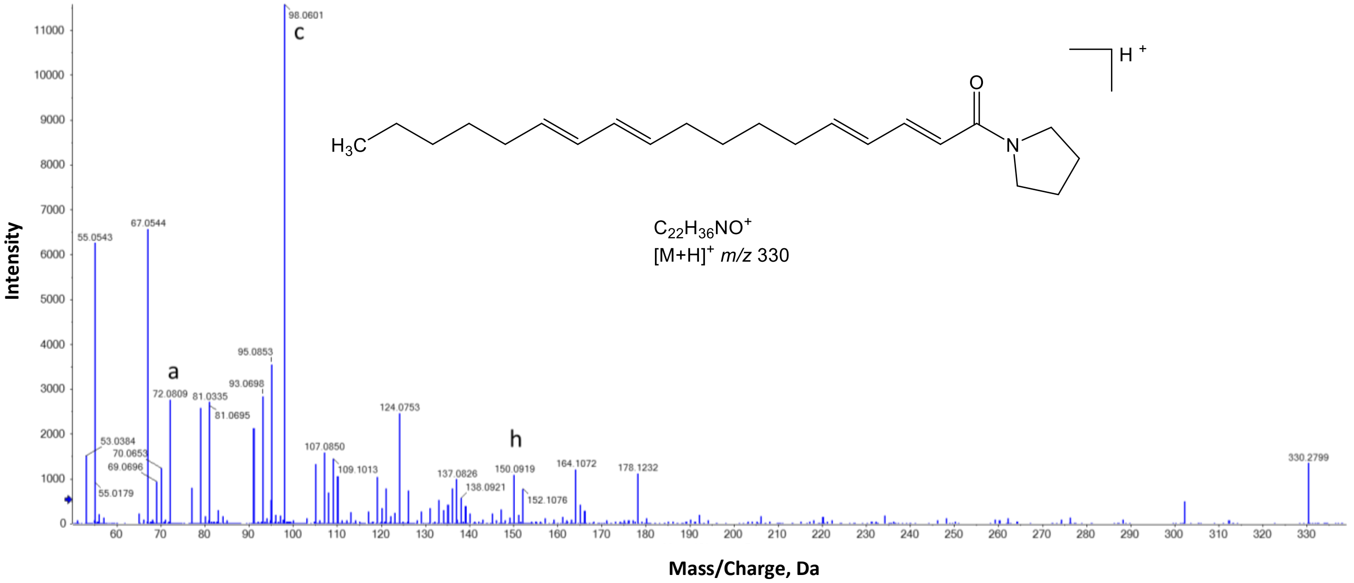

Figure S10_148: MS/MS spectrum of P148.

P149, 1-(Pyrrolidinyl)-18-2,4,11-octadecatrien-1-one

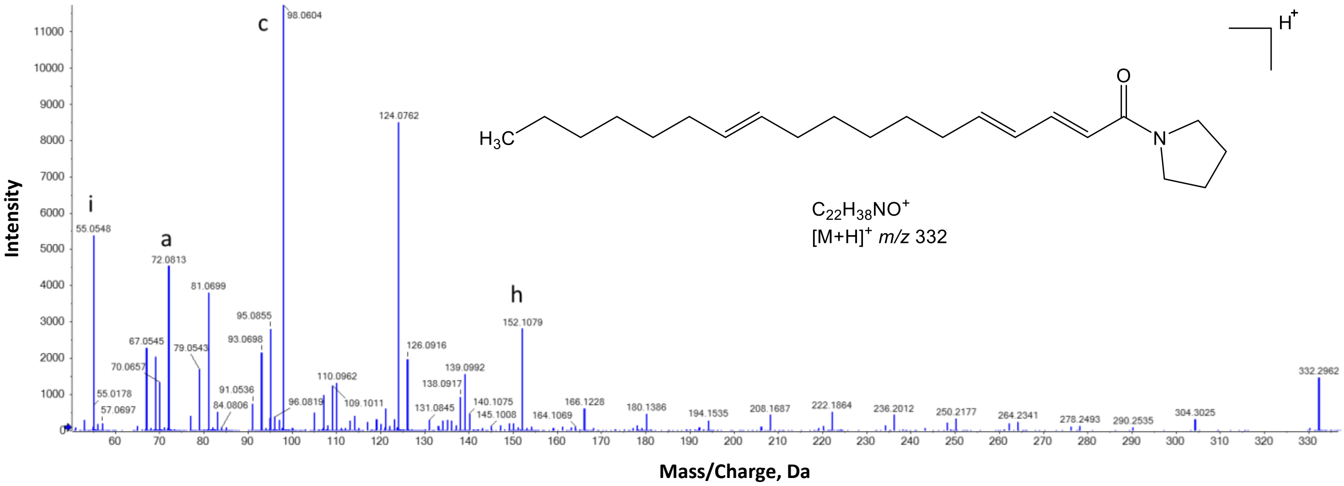

Figure S10_149: MS/MS spectrum of P149.

P150, Pipericine

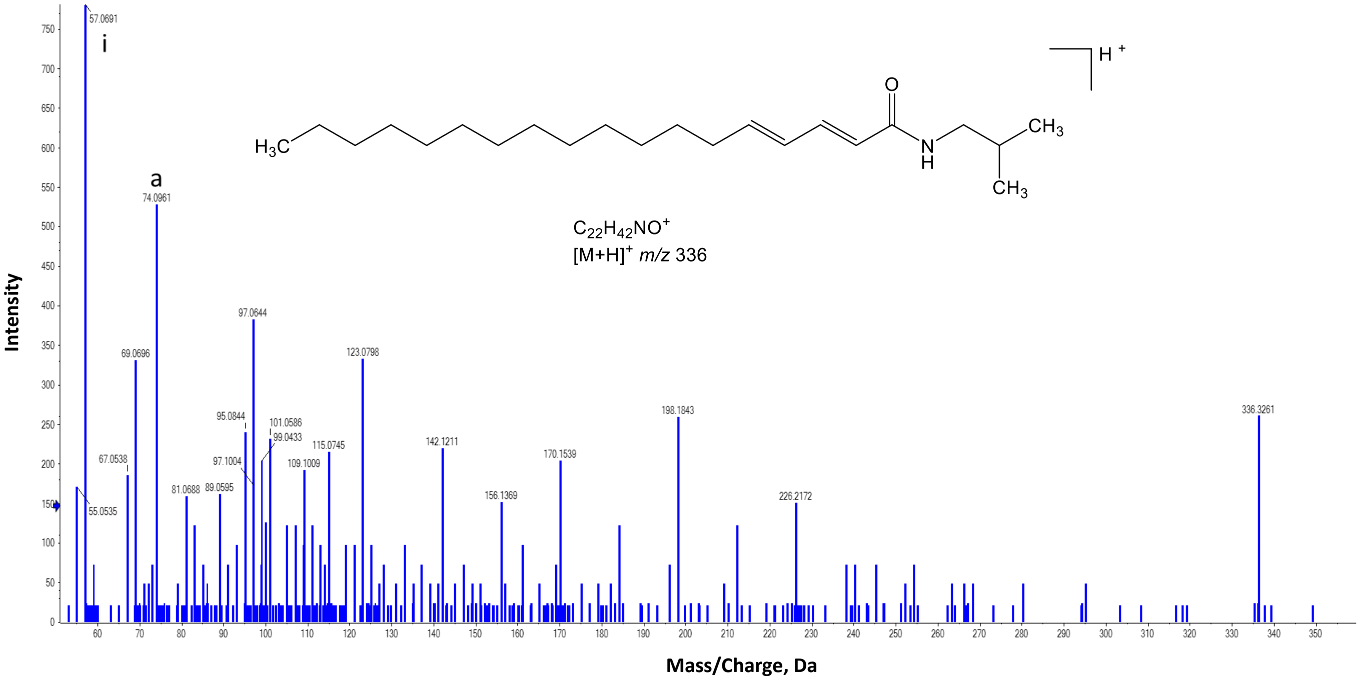

Figure S10_150: MS/MS spectrum of P150.

P151: $C_{21}H_{28}NO_3^+$, isomer of 1-(Piperidinyl)-2,4,11-heptadecatrien-1-one (formula see **P149**)

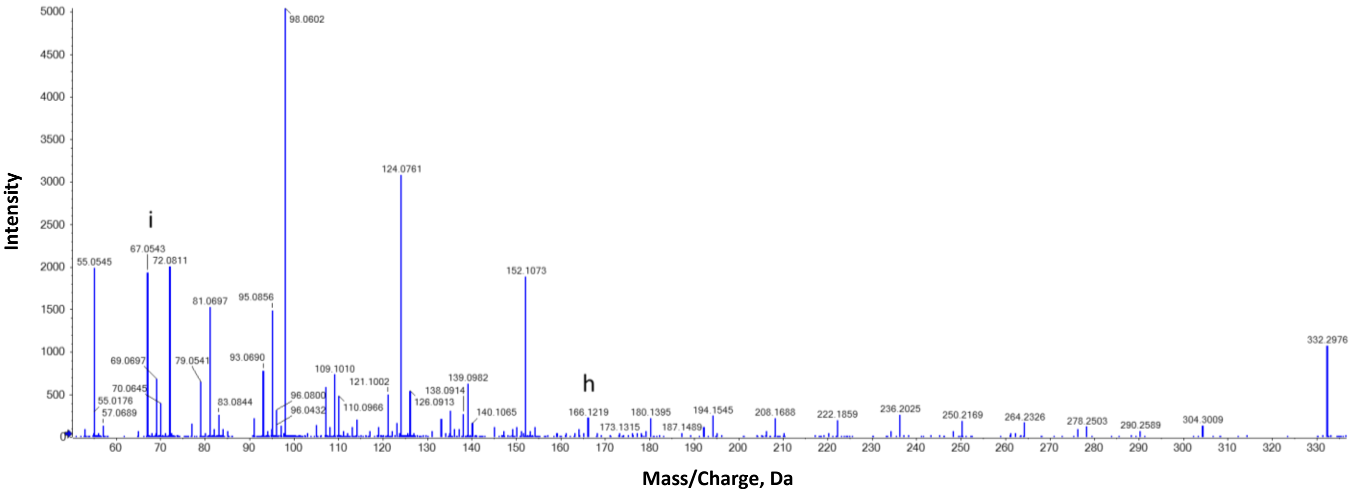

Figure S10_151: MS/MS spectrum of P151.

P152, 1-(Pyrrolidinyl)-2,4-hexadecadienmide

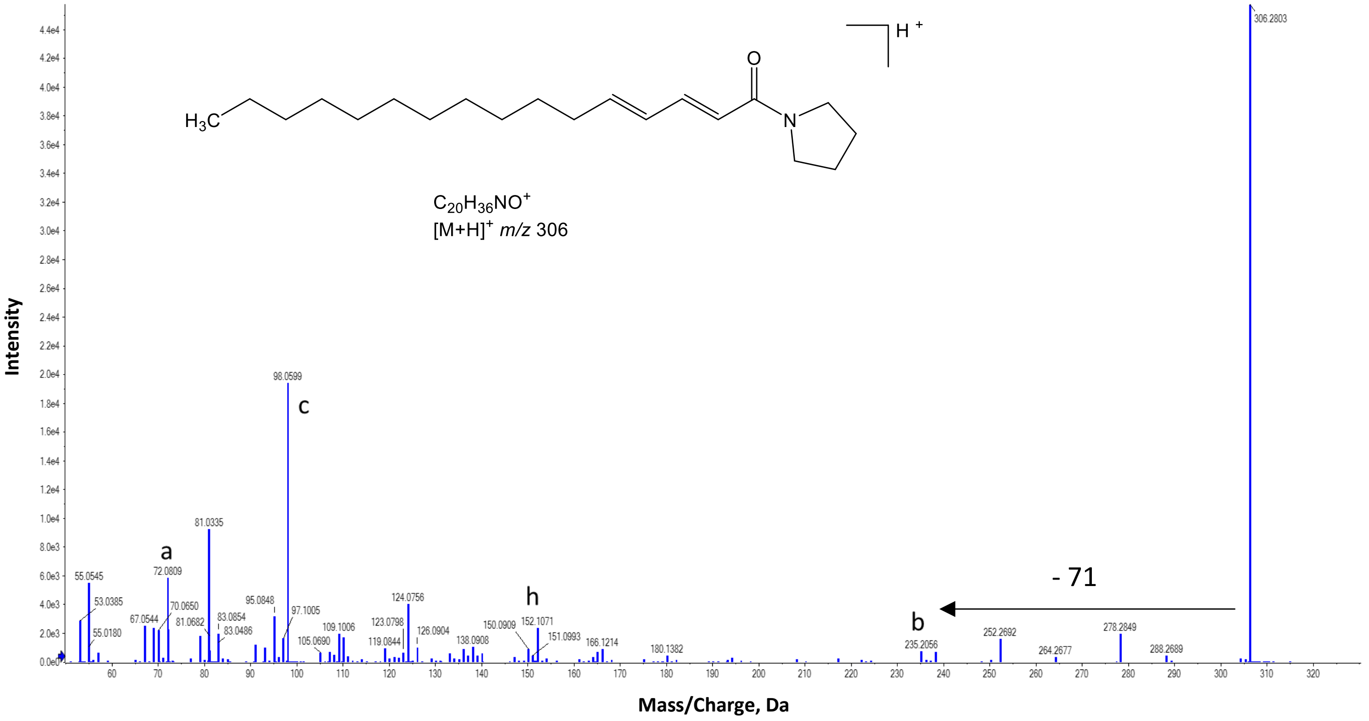

Figure S10_152: MS/MS spectrum of P152.

P153, *N*-Isobutyl-2,4,12- octadecatrienamide

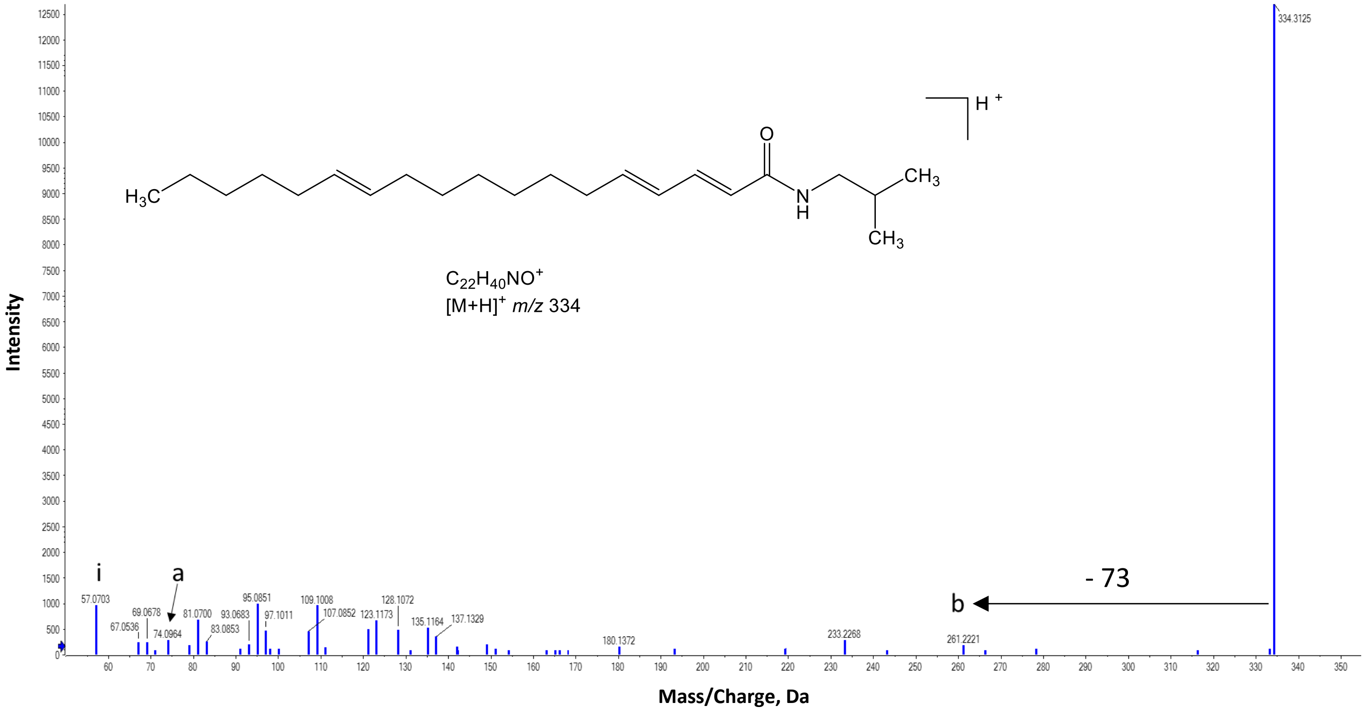

Figure S10_153: MS/MS spectrum of P153.

P154, 1-(Pyrrolidyl)-2,4,12-octadecatrienamide

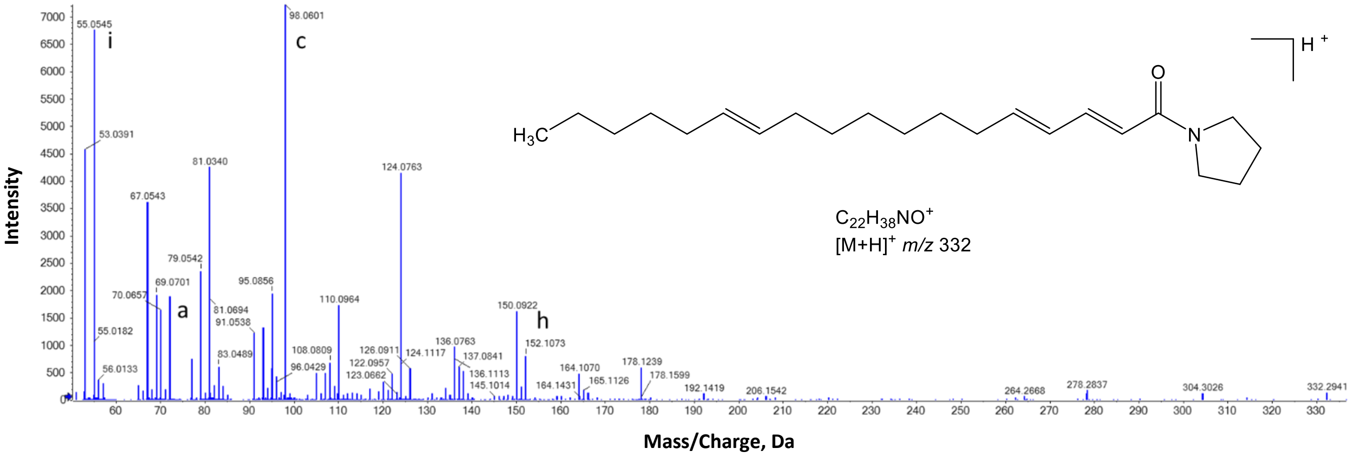

Figure S10_154: MS/MS spectrum of P154.
